# GABA_A_ receptor-mediated seizure liabilities: a mixed-methods screening approach

**DOI:** 10.1007/s10565-023-09803-y

**Published:** 2023-04-24

**Authors:** Konstantina Bampali, Filip Koniuszewski, Florian D. Vogel, Jure Fabjan, Christos Andronis, Eftychia Lekka, Vassilis Virvillis, Thomas Seidel, Annie Delaunois, Leandro Royer, Michael G. Rolf, Chiara Giuliano, Martin Traebert, Gautier Roussignol, Magali Fric-Bordat, Ludmilla Mazelin-Winum, Sharon D. Bryant, Thierry Langer, Margot Ernst

**Affiliations:** 1 Department of Pathobiology of the Nervous System, Center for Brain Research, Medical University Vienna, Spitalgasse 4, 1090 Vienna, Austria; 2 Biovista, 34 Rodopoleos Street, 16777 Athens, Greece; 3 Department of Pharmaceutical Sciences, Division of Pharmaceutical Chemistry, University of Vienna, Josef-Holaubek-Platz 2, 1090 Vienna, Austria; 4 UCB Biopharma SRL, Chemin du Foriest, Braine-L’Alleud, Belgium; 5 R&D Biopharmaceuticals, Astra Zeneca, Pepparedsleden 1, 431 83 Mölndal, Sweden; 6 R&D Biopharmaceuticals, Astra Zeneca, Fleming Building (B623), Babraham Research Park, Babraham, Cambridgeshire, CB22 3AT UK; 7 Novartis Institutes for Biomedical Research, Fabrikstrasse 2, CH-4056 Basel, Switzerland; 8 Sanofi R & D, Preclinical Safety, Montpellier, France; 9 Inte:Ligand GmbH, Mariahilferstrasse 74B/11, 1070 Vienna, Austria

**Keywords:** GABA_A_ receptor, Pharmacotoxicology, Allosteric sites, Amoxapine, Seizures

## Abstract

GABA_A_ receptors, members of the pentameric ligand-gated ion channel superfamily, are widely expressed in the central nervous system and mediate a broad range of pharmaco-toxicological effects including bidirectional changes to seizure threshold. Thus, detection of GABA_A_ receptor-mediated seizure liabilities is a big, partly unmet need in early preclinical drug development. This is in part due to the plethora of allosteric binding sites that are present on different subtypes of GABA_A_ receptors and the critical lack of screening methods that detect interactions with any of these sites. To improve in silico screening methods, we assembled an inventory of allosteric binding sites based on structural data. Pharmacophore models representing several of the binding sites were constructed. These models from the NeuroDeRisk IL Profiler were used for in silico screening of a compiled collection of drugs with known GABA_A_ receptor interactions to generate testable hypotheses. Amoxapine was one of the hits identified and subjected to an array of in vitro assays to examine molecular and cellular effects on neuronal excitability and in vivo locomotor pattern changes in zebrafish larvae. An additional level of analysis for our compound collection is provided by pharmacovigilance alerts using FAERS data. Inspired by the Adverse Outcome Pathway framework, we postulate several candidate pathways leading from specific binding sites to acute seizure induction. The whole workflow can be utilized for any compound collection and should inform about GABA_A_ receptor-mediated seizure risks more comprehensively compared to standard displacement screens, as it rests chiefly on functional data.

**Supplementary Information:**

The online version contains supplementary material available at 10.1007/s10565-023-09803-y.

## Introduction

γ-Aminobutyric acid type A receptors (GABA_A_Rs), GABA-gated pentameric anion channels, are known as important CNS drug targets in the treatment of various neuropsychiatric conditions (Korpi and Sinkkonen 2006; Sieghart and Savic 2018) and for the induction and maintenance of general anesthesia (Antkowiak 2015; Olsen and Li 2011). They are also a large, highly abundant, and complex source of adverse drug effects that are mediated by unwanted activity at some of the many subtypes of this receptor family. Such adverse reactions come from two categories: (i) unwanted on-target effects (such as memory impairments or ataxia) occurring after targeting GABA_A_Rs for medical purposes (such as in hypnotic medication in order to treat various forms of insomnia); (ii) off-target effects related to GABA_A_Rs exhibited by new drugs engineered to target other biomolecules. Many important insights into the mechanisms of GABA-mediated side effects come from research dealing with GABA_A_ receptor-targeting compounds. This contributes to a more complete understanding of the pathways that connect a given GABA_A_R targeting drug with the (wanted and unwanted) outcomes it elicits. The prediction and prevention of unwanted interactions with this large protein family in preclinical drug development (Bowes et al. 2012) is facilitated by such insights. This study is part of a consortial effort to derisk drug development in early preclinical stages, with particular emphasis on seizures and convulsions as adverse events (https://neuroderisk.eu/).

GABA_A_Rs are expressed by nearly all or all CNS neurons and thus are involved to some degree in almost every CNS function. This ubiquitous presence makes it rather challenging to disentangle specific contributions of individual entities to physiological events. The pharmacology and toxicology of GABA_A_R subtypes illustrates that a wide range of normal function is influenced by this target family, prominent examples being vigilance, seizure threshold, and EEG power bands (D'Hulst et al. 2009; Galanopoulou 2008). Another important distinction needs to be made between the effects induced by either single or chronic exposure to a substance interacting with GABA_A_Rs (Gravielle 2018). The GABA-ergic signaling system in the nervous system is well known for the high degree of plasticity with which it responds to chronic substance exposure (Gravielle 2018). Here, we focused on acute induction of seizures and convulsions as adverse effects (AEs) by compounds which influence the activity of GABA_A_Rs by directly binding and subsequent changing of channel activity. Seizure/convulsion AEs are known to potentially result from (functional) inhibition of GABA_A_Rs (Galanopoulou 2008; Meldrum and Rogawski 2007). Channel blockers and orthosteric antagonists are well known as seizurogenic toxins, and benzodiazepine site negative modulators (historically called benzodiazepine receptor inverse agonists), such as DMCM (methyl 6,7-dimethoxy-4-ethyl-β-carboline-3-carboxylate), are also known as seizurogenic. Not all receptor subtypes possess binding sites for benzodiazepines, and thus, it is of interest to identify the precise molecular players for different classes of toxins.

Each subtype of the GABA_A_ receptor family receptors is assembled in mammalian species as homo or heteropentamers drawn from a repertoire of 19 subunits (Olsen and Sieghart 2008; Sieghart 1995). This results in a large variety of receptor subtypes that display distinctive properties (Olsen and Sieghart 2008). As all members of the pentameric ligand-gated ion channel superfamily (Jaiteh et al. 2016), GABA_A_Rs are composed of five identical or homologous subunits. The subunits are glycoproteins with three domains. While the extracellular domains (ECDs) and transmembrane domains (TMDs) are highly conserved across the entire superfamily, the intracellular domains (ICDs) are much more variable (and do not exist, e.g., in bacterial pentameric ligand-gated ion channels) (Koniuszewski et al. 2022). Sequence similarity divides the mammalian paralogs into nine beta-like and ten alpha-like subunits, which branch further into the respective subunit classes. At this time, a recent surge of structural studies provides research now with the means to investigate the structural basis for unwanted drug effects at atom level detail and to examine binding sites of potentially seizurogenic compounds.

**Fig. 1:**
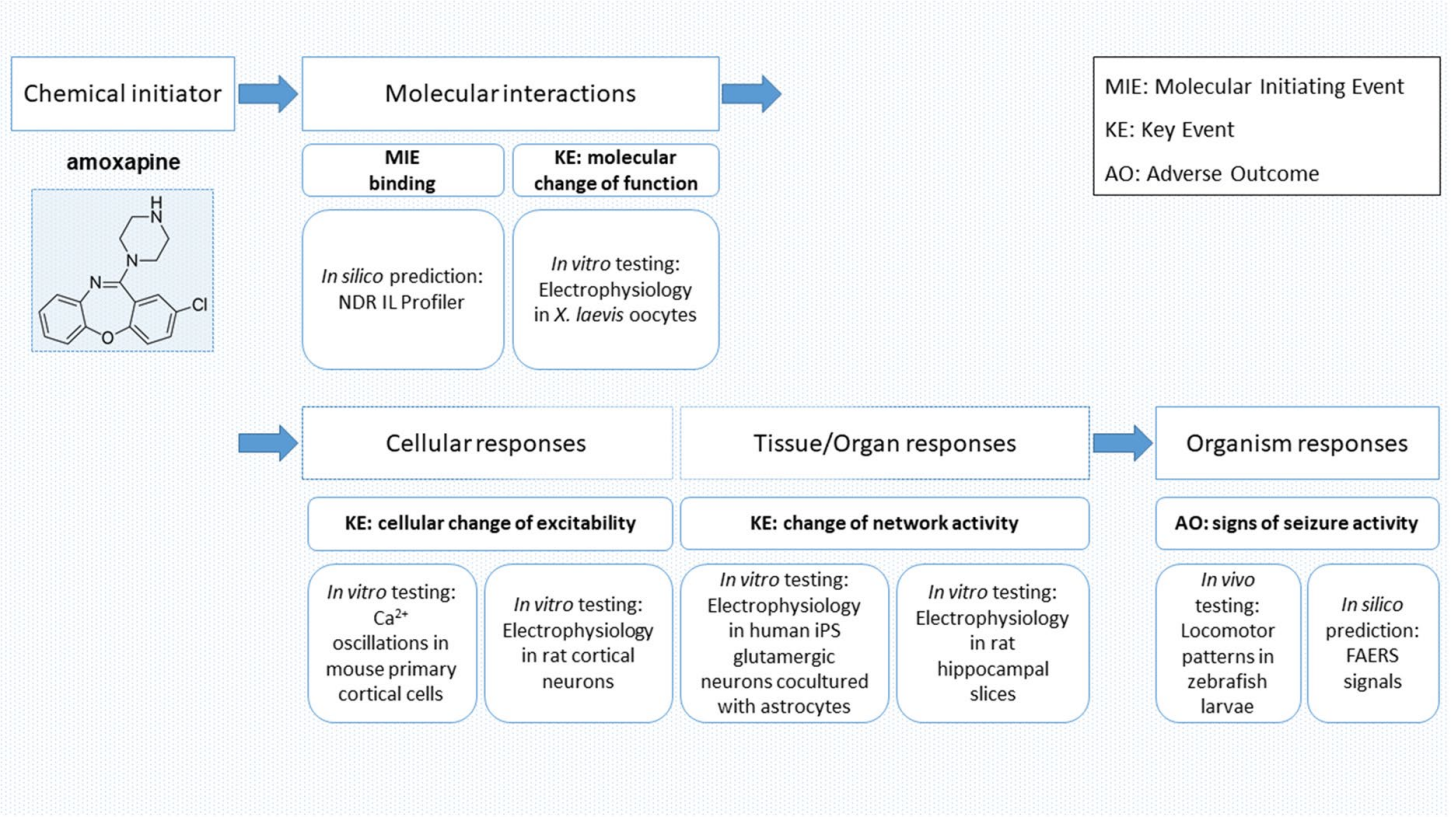
Workflow mapped to adverse outcome pathway (AOP) scales: Assays used for in silico, in vitro, and in vivo experiments to identify molecular initiating events (MIEs) and key events (KEs) related to structural alerts for identifying seizure risk adverse outcomes (AOs)

The “Adverse Outcome Pathway” (AOP) framework was established to reflect the mechanisms by which a stressor, which can be a small molecule, elicits a series of events leading to the observed adverse outcome (OECD 2013). The aim is to map key events, which can be detected and contain necessary parts of the mechanism, into standardized terminology and to use the AOP for the development of assays and biomarkers. For ligands that bind to the channel blocking (picrotoxin) site of GABA_A_Rs, the so-called AOP 10 has been described (https://aopwiki.org/aops/10). Several early events such as binding to the target, as well as late events such as changes in organ (brain) activity, can be predicted and tested by different methods. Such methods, employed in the NeuroDeRisk project (https://neuroderisk.eu/) to test the effects of small drug-like molecules and drugs for potential seizure liability, can be arranged in a workflow that follows the AOP framework, helping to unravel the different key players of the mechanisms leading to the adverse outcome and to optimize preclinical prediction tools and assays. Figure 1 provides the result of mapping experimental approaches to the AOP framework.

Thus, we employed in silico screening with the NeuroDeRisk IL Profiler V1.0 (https://docs.inteligand.com/ndr/il-profiler/) to generate hypotheses for drug-pocket interactions (molecular initiating events) and subsequently tested the hit compound amoxapine in an array of preclinical assays (Fig. 1). This substance has been noted in the past as a potential seizurogenic agent that interacts with GABA_A_Rs (Squires and Saederup 1993). We also mined a FAERS dataset to connect drugs with pharmacovigilance-derived seizure alerts. In total, we deduced several candidate AOPs from the findings and propose this framework to connect GABA_A_R targeting molecules with preclinical seizure alerts, providing testable hypotheses.

## Results

### Structural basis of GABA_A_R pharmacotoxicology

**Fig. 2:**
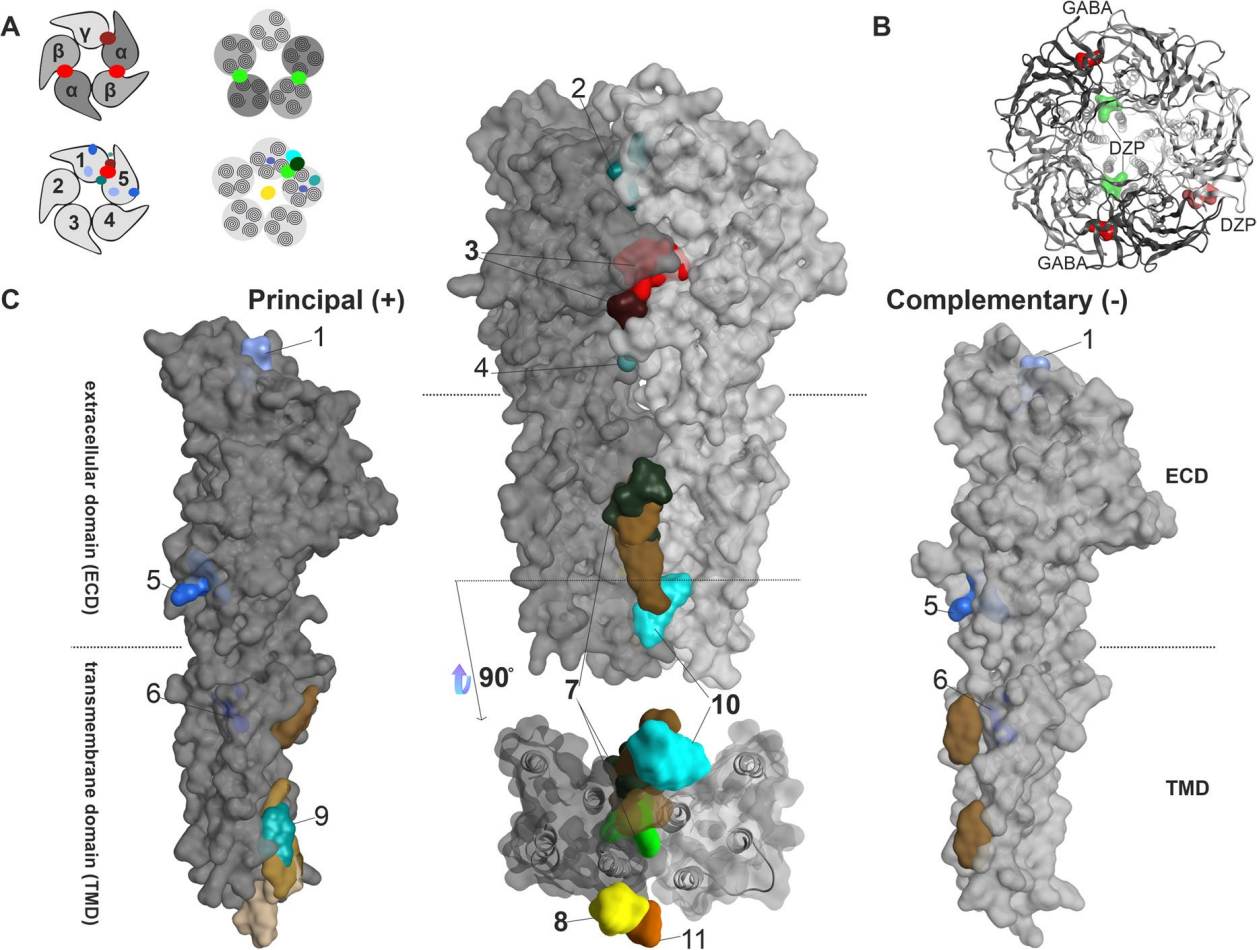
Summary of all identified and putative binding sites, for which structural evidence exists. **A** Top: representation of αβγ GABA_A_R pentamers. Below: representation of a generic pentamer. Representative intrasubunit binding sites and interfaces found on the ECD (left), TMD (right), as well as the channel pore (right) are depicted. Colors are matching the ones depicted in panels **B**, **C**. **B** Top view of an atomic model of an α1β3γ2 GABA_A_R (6HUP) in ribbon representation, with GABA and diazepam binding to the ECD, as well as the diazepam TMD binding site shown in surface filling representation. **C** All intrasubunit binding sites that can be found on the principal ( +) side in dark grey (left). Representation of all binding sites that occupy an interface between two subunits (middle). Representation of all intrasubunit binding sites that can be found on the complementary (-) side in light grey (right). The extracellular and transmembrane domains are marked as ECD and TMD on the individual subunit renderings, and dashed lines indicate the approximate localization of the lipid collar in the space filling renderings. Below the upright dimer, a view of the transmembrane domain binding sites as seen from the intracellular space is depicted. The surface maps of the principal and complementary sides that are depicted in this summary were obtained from 6HUP. All ligands are shown in space filling representation (representative ligands for each site: 1—“fragment” in sky blue, 2—AM-3607 in turquoise, 3—GABA/diazepam in red and ketamine in dark red, 4—Ba^2 +^ -atom in cadet grey, 5—chlorpromazine in ocean blue, 6—propofol in navy blue, 7—diazepam in light green and avermectin in dark green, 8—picrotoxin in yellow, 9—pregnenolone sulfate in light cyan, 10—alphaxolone in dark cyan, 11—memantine in orange. Brown sites represent lipid-associated sites (e.g., cholesterol in light and dark brown, PIP2 in sand). Sites 1, 5, 6, and 9 are intrasubunit-located, whereas sites 2, 3, 4, 7, and 10 are interface-located. Site 8 and site 11 are located within the channel pore. This summary of binding sites resulted from a superposition of atomic resolution structures of GABA_A_R and homologous proteins. The PDB files used, citations, as well the full description of the ligands occupying the binding sites can be found in Supplementary Table S1. Note that structural data for the intracellular domain is lacking

In order to fully describe a molecular initiating event (for toxins, usually a ligand binding at a target binding site), the binding site must be known. GABA_A_Rs contain multiple allosteric sites in addition to the orthosteric sites, a property shared with all members of the pLGIC family (Koniuszewski et al. 2022). Here, we compile the current knowledge on small molecule interaction sites on GABA_A_Rs based chiefly on structural and some indirect evidence. Figures 2A–C provide an overview on all positions of a generic subunit dimer where small molecule binding sites have been observed in experimental structures of GABA_A_Rs and homologous pLGICs.

At this time, the existing structural evidence largely confirms the localization of binding sites as proposed by biochemical and computational evidence and adds novel and highly interesting candidates for sites at which allosteric ligands exert pharmaco-toxicological effects. Figure 2C provides a cumulative overview of small molecule localizations as observed in GABA_A_R structures as well as some that were observed in homologous proteins (Supplementary Table S1, Supplementary Figures S1, S2). At subunit interfaces, several binding sites have been observed within the superfamily including the orthosteric site (3), a site for general anesthetics in the upper TMD (7), and a site for modulatory steroids in the lower TMD (10) as reviewed in detail recently (Koniuszewski et al. 2022). Sites which are localized in or on a single subunit include the so-called lipid-associated sites used by inhibitory steroids in the TMD (9). The binding site used by the tricyclic molecule chlorpromazine in a bacterial homolog’s ECD has been recently mapped to the α5 subunit of GABA_A_Rs (Bampali et al. 2022). Additional sites for endogenous molecules have been described and add to the overall complexity of allosteria (Supplementary Figures S1, S2).

Prediction of ligand binding at sites that mediate ortho- and allosteric effects on channel activity enables the in silico generation of early alerts for GABA_A_R-mediated toxicology. GABA-mediated currents can be reduced by orthosteric antagonism, as is the case for bicuculline, or by allosteric negative modulation, as exemplified by the seizurogenic benzodiazepine site ligand DMCM, and also by direct pore blockage by, e.g., picrotoxin (Fig, 2C). Importantly, owing to the existence of 19 subunits, binding sites can vary in their amino acid composition. In the implementation of the NeuroDeRisk IL Profiler V1.0 which was used for this study, models for the orthosteric sites, the high affinity benzodiazepine binding sites, the channel (picrotoxin) site, and the inhibitory neurosteroid site have been employed. The two latter are more highly conserved across the majority of the subunits (Koniuszewski et al. 2022), while the sites at the ECD interfaces for GABA and benzodiazepines show high variability.

**Fig. 3:**
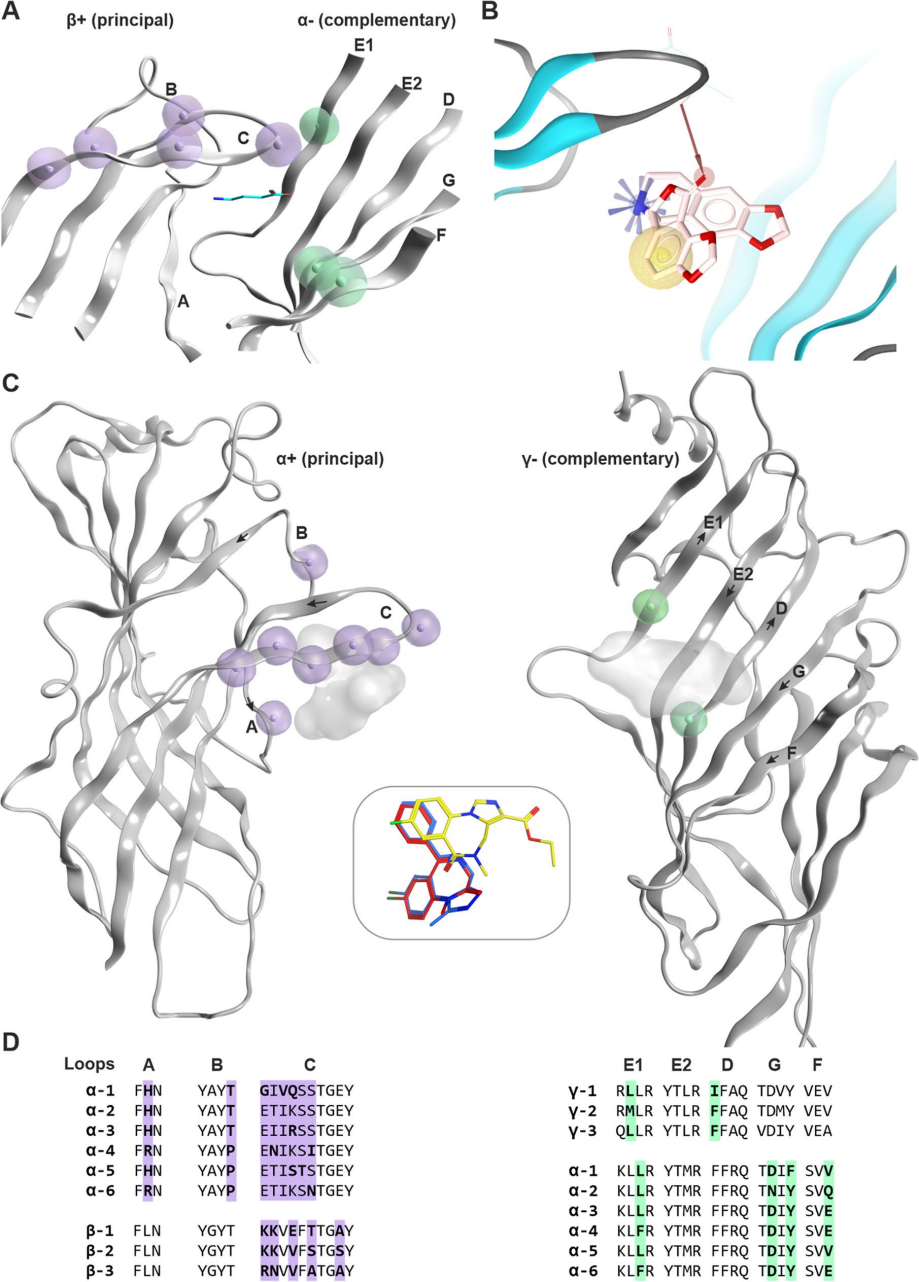
Known ECD interface binding sites for GABA and benzodiazepines: **A** GABA sites: β3 + / α1 − of 6HUJ showing in lilac residue positions that are different in the beta subunits (β1, β2, β3), as well as those that differ in the alpha subunits in light green. The GABA molecule is rendered in cyan sticks. More details are provided in Supplementary Figure S3. **B** Chemical feature interactions of the bicuculline bound 6HUK structure rendered with LigandScout 4.4 Expert. Yellow spheres, blue stars, and red vectors represent hydrophobic, positive ionizable, and hydrogen bond acceptor interactions, respectively. **C** Side view of the benzodiazepine binding site (α + /γ − interface) from a PDBeFold superposition of selected atomic resolution structures (PDB IDs: 6HUP—diazepam, 6HUO—alprazolam, 6D6T/6D6U—flumazenil). The subunits are rendered individually for more clarity, and the variable positions are highlighted as in panel **A** with lilac for the principal and light green for the complementary face, respectively. The insert box in the middle depicts the binding modes of diazepam (red), alprazolam (blue), and flumazenil (yellow). The corresponding ligands are displayed on the protein as shadows for orientation. The direction of the beta strands on the complementary face is indicated by arrows. **D** Partial alignment for the binding site forming segments matching panels **A** and **B**. More details on variable positions, including those that are found on segment F, are provided in Supplementary Figures S4 and S5)

Apart from the homologous binding sites on ρ-subunit homopentameric receptors, in most cases, the β and α subunits of GABA_A_R form the principal ( +) and complementary ( −) components of the GABA binding site, respectively. One of the most widespread receptor subtypes contains two α, two β, and one γ subunit, resulting in two GABA binding sites at the β + /α − interfaces (Bencsits et al. 1999; Sigel and Ernst 2018) (Fig. 3A). We recently found that clozapine and loxapine, as well as other structurally similar molecules, can inhibit α5β3γ2 receptors as orthosteric antagonists (Bampali et al. 2022). Some of these inhibit currents also of α1β3γ2 receptors.

The best-studied allosteric binding site in the ECD is the high affinity benzodiazepine binding site. Benzodiazepines are clinically important sedative/hypnotic and anxiolytic drugs that modulate GABA_A_Rs by acting as “positive allosteric modulators” (PAMs), while negative allosteric modulators (NAMs) acting at this site are known to elicit “inverse” effects such as anxiogenesis and/or seizures (Sieghart and Savic 2018). This distinct site from the orthosteric site is structurally homologous to the β + /α − agonist binding sites and is located at the α + /γ − interface of the extracellular domain (Fig. 3B and C). Models, such as NDR-IL-GABA-A-Flumazenil-6d6t and NDR-IL-GABA-A-PAM-Diazepam-6hup, in the NeuroDeRisk IL Profiler V1.0 (https://docs.inteligand.com/ndr/models/) were developed using some chemical feature interaction information derived from cryogenic electron microscopy (Cryo-EM) resolved diazepam and flumazenil GABA_A_R bound structures (PDB IDs: 6HUP (Masiulis et al. 2019) and 6D6T (Zhu et al. 2018), respectively. Since NAM effects at α1-containing benzodiazepine sites are seizurogenic (Crestani and Rudolph 2015), but not those at α5-containing benzodiazepine sites, subtype information is highly relevant for this class of sites as well (Fig. 3C, D). Differences between subtypes can be derived from the partial alignment shown in Fig. 3D and from Supplementary Figure S5.

Depending on the subunits that contribute, the ECD interfaces can form additional modulatory sites like the pyrazoloquinolinone (PQ) binding site at α + /β − (Xenia Simeone et al. 2017). Histamine is a ligand of the β3 + /β3 − interface (Sente et al. 2022). So far, no ligands are known for pockets at most of the other subunit interfaces (e.g., γ + /β −). For these sites, structural data still is incomplete, and thus, in silico models are vital for early preclinical drug derisking.

An additional concern for the informed selection of preclinical species and cellular systems for assays are diverging sequences in different organisms. Here, we consider the protein sequences of mammalian species which are in regular use as laboratory animals in (neuro-) pharmaco-toxicological research, organisms routinely or historically used for experimental binding studies, and of zebrafish, an increasingly popular organism in neuroscientific research (Supplementary Table S2). For the 19 mammalian subunits, it can be stated that those subunits with very high expression levels in the brain are highly conserved, while some of the subunits with highly regio-specific expression show much lower conservation (Supplementary Figures S6, S7, and S8). Subunits which are highly conserved among mammalian organisms also display rather high conservation across vertebrates, e.g., the α1 or γ2 subunits which are also highly similar in many fish species including zebrafish. Thus, toxicological screenings in zebrafish or zebrafish larvae will recapitulate effects well which are mediated by those highly conserved and widely expressed subunits. In contrast, effects which might be mediated by the most variable subunits, such as the mammalian epsilon subunit, may even diverge between laboratory rodents and primates, and cellular assay systems may reflect species-specific effects.

The individual subunits consist of more variable and more conserved segments. Interestingly, the signal peptides and the very N-terminal segments display very high variability (Supplementary Figures S6, S7), while the remainder of the ECD is more conserved with variable segments at the ligand binding domains, reflecting subunit-specific binding sites. The highest conservation across the species is seen for the TMDs, and the highest variability in the ICDs (Supplementary Figure S7). Full sequences are provided in Supplementary Figure S8, where details can be examined at the amino acid level.

### In silico profiling of drug structures for GABA_A_R pharmacotoxicology

In silico activity profiling is a well-established concept for multiple purposes, such as identification of molecular initiating events, and targets related to phenotypic data, repurposing existing drugs for new targets, addressing ligand selectivity issues, as well as prediction of adverse effects (Bryant and Langer 2013). In addition, 3D-pharmacophores have been validated as effective tools for in silico profiling, data mining, and medicinal chemistry decision-making (Langer 2010; Langer and Bryant 2008). The NeuroDeRisk IL Profiler is an innovative tool that incorporates 3D-pharmacophore technology from LigandScout (Wolber and Langer 2005) and activity profiling algorithms from Inte:Ligand to identify chemical structures for risk of neurotoxic adverse events (NeuroDeRisk IL Profiler V1.0. https://docs.inteligand.com/ndr/il-profiler). It was developed as part of the Horizon 2020 Innovative Medicines Initiative 2 (NeuroDeRisk 821,528) to cover prediction of risk for seizure (including GABA_A_R pharmacology), suicidality, and peripheral neuropathies. The tool is highly useful for red flagging chemical structures with risk early in development programs, supporting investigative research on adverse outcome pathways, and giving clues on molecular initiating events and adverse events.

We took advantage of the NeuroDeRisk IL Profiler V1.0 to profile 81 drugs (111 chemical structures, see “Methods” and Supplementary Table S3) selected from public databases against 8 GABA_A_R models. The development of the models is fully described in the publically available documentation (https://docs.inteligand.com/ndr/models/). The results involving models for GABA_A_-related positive allosteric modulation (PAM), similar to that of diazepam, and seizure risk (allosteric inhibition, channel blocking, orthosteric site antagonism) are shown in Table 1. None of the drug structures profiled hit the GABA_A_ orthosteric site agonist models, while flumazenil and brexanolone (neurosteroid) were correctly identified by the NDR-IL-Flumazenil-6d6t and NDR-IL-GABA-A-NSteroid-Pregnanolone-5o8f models, respectively (Supplementary Figure S9).

**Table 1:**
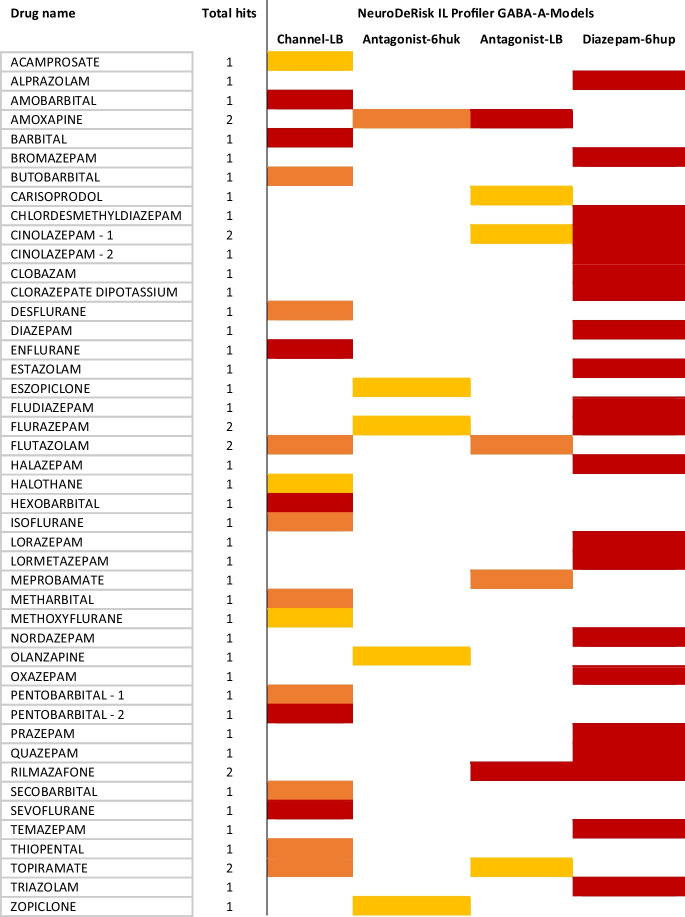
Results from in silico profiling of 81 drugs using selected GABA_A_R models in the NeuroDeRisk IL Profiler V1.0. Drugs that were hits for the orthosteric site antagonism, channel blocking, and positive allosteric modulation (PAM) diazepam models are included in Table 1 (see Supplementary Figure S9 for full profile). Red indicates a higher fit score to the model, orange and yellow have lower fit scores (fit scores as defined in the public documentation) while white was not a hit. All other results are in the Supplementary Figure S9

Benzodiazepine drugs in the dataset, including clobazam, were correctly identified by the NDR-IL-GABA-A-PAM-Diazepam-6hup model and were consistent with the anticipated PAM diazepam binding site (highest fit score by diazepam). Several other benzodiazepine drug chemical structures used as queries, such as flutazolam, ketazolam, medazepam, and mexazolam, were not flagged as hits by the NDR-IL-PAM-Diazepam-6hup model (Supplementary Figure S9). However, medazepam is a prodrug, exerting its GABA_A_R effects through the active metabolites diazepam, nordazepam, temazepam, and oxazepam, all of which were identified by the model (Table 1). Similarly, active metabolites of flutazolam, ketazolam, mexazolam, and rilmazafone (flurazepam, diazepam, chlorazepam, and 8-chloro-6-(2-chlorophenyl)- N,N-dimethyl-4H-1,2,4-triazolo(1,5-a) (1,4)benzodiazepine-2-carboxamide, respectively) were identified by the model as well.

Interestingly, flutazolam hit both the channel and antagonist models alerting potential seizure risk based on its chemical structure, while flurazepam, rilmazafone, and one stereoisomer of cinolazepam also hit models for GABA_A_R orthosteric site antagonism. Topiramate, an anticonvulsant drug acting on GABA_A_Rs but with an unidentified mode of action, had moderate and moderately low fit scores for the GABA_A_ channel and orthosteric antagonist models, respectively, while olanzapine, an antipsychotic drug, and the structurally similar antidepressant amoxapine also hit the GABA_A_ orthosteric antagonist models (Table 1). Some barbiturates (amobarbital, barbital, pentobarbital, and hexobarbital), anesthetics, and acamprosate (alcohol use disorder) hit to the GABA_A_ channel model indicating a potential link to GABA_A_R channel interaction (Table 1). Carisoprodol (musculoskeletal pain) and meprobamate (an active metabolite of carisoprodol) both hit the NDR-IL-GABA-A-gs-Antag-LB model, the latter with a higher fit score.

The highest scoring hit for the Antagonist-6huk model (NDR-IL-GABA-A-gs-Antag-6huk-3) was amoxapine, while the fit to the orthosteric site Antagonist-LB model (NDR-IL-GABA-A-gs-Antag-LB) scored even higher. Additionally, it was the only drug in the dataset that hit both orthosteric antagonist models, and not any other GABA_A_R models in the study.

Antagonists of the orthosteric GABA/bicuculline binding sites are known to be toxic and induce seizures, and thus, hits were not expected to be found among the selected test compounds which are all approved for use in humans. Nevertheless, the amoxapine drug package inserts and pharmacovigilance data indicated potential seizure risk though no indication of the mechanism of action or target. Moreover, the reduction of GABA-elicited currents as an off-target effect has been described previously for different related compounds, such as clozapine (Bampali et al. 2022). Based on the unique hit profile and our previous findings on the similar compound clozapine (Bampali et al. 2022), amoxapine was selected for a comprehensive experimental follow-up.

### Pharmacovigilance alerts for seizure/convulsion AEs of the compounds

**Fig. 4:**
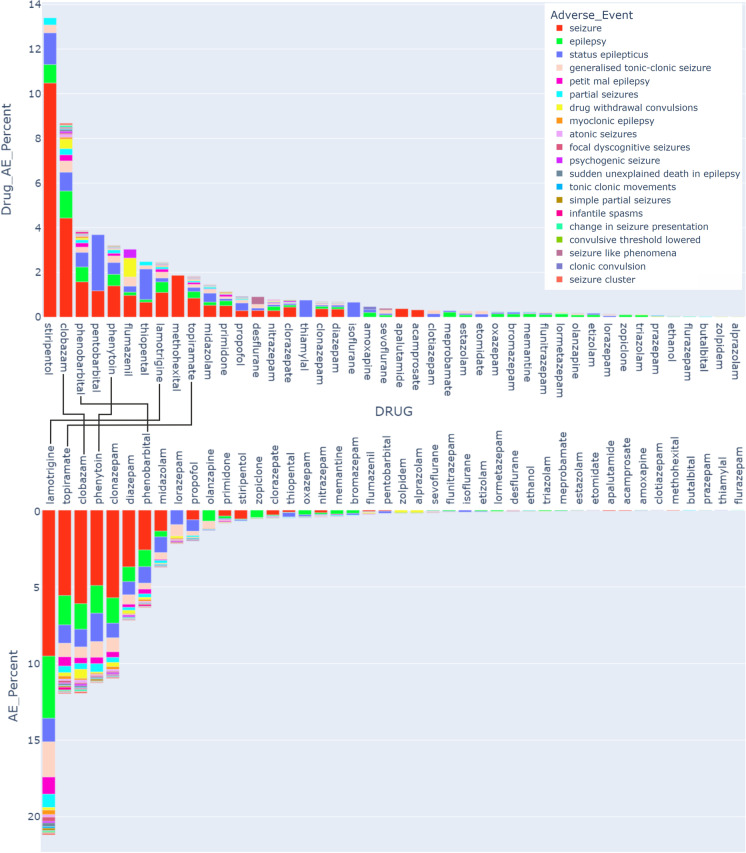
Pharmacovigilance data. The upper box plot displays the fraction (in %) of each AE for a specific drug from the total number of reports for this drug. The drugs are sorted by the total burden of the seizure/convulsion groups of AE, and the AEs in the legend are also sorted by the size of their contribution to the total AE count for these drugs. The lower box plot displays the fraction (in %) of the cumulative seizure AEs for each drug among the total reports in the seizures MedDRA category (46,285 total reports). Compounds which occur in the top 10 of both are connected. The total reports and the reports per AE per drug for the selected drugs and AEs are shown in Supplementary Table S4

In addition to the NeuroDeRisk IL Profiler in silico screen to predict GABA_A_R molecular initiating events, all 81 compounds were subjected to a data search in the FAERS database as described in the “Methods” section. We retrieved from the entire set of the used records all those which contain any of the MedDRA terms from the neurological seizure/convulsion group and filtered on the basis of the disproportionality analysis as described in the methods (Supplementary Figure S10). A total of 46,285 records reflecting seizure-type AEs for 41 of the screened drugs passed the statistical criteria (Supplementary Figures S11 and S12) and went into further analysis. Figure 4 displays the contribution of each AE to the total AEs of a given drug (Fig. 4, upper part) and the contribution of each drug to the total pool of seizure-category AEs in the entire dataset (Fig. 4, lower part). The top 20 AEs account for > 98% of the 46,285 records; only these are displayed in Fig. 4.

Of the total pool of drugs known to exert effects on GABA_A_Rs, 43% are associated with AEs from the seizures/convulsions category at the level of stringency used for the analysis. Of note, of the 43 benzodiazepines for which FAERS records were available, we found seizure/convulsion AE alerts for 18 of them, and two Z-drugs zopiclone and zolpidem, and the benzodiazepine antagonist flumazenil. The chemically atypical 1,5-benzodiazepine clobazam gives the strongest signal of the analyzed benzodiazepines. In the same vein, seizures were observed in two clinical trials (LundbeckLLC 2012, 2018).

This implies that the liability to induce seizure-category AEs is not uniform for benzodiazepine-type drugs. Comparing the rankings of the cumulative fraction of seizure/convulsion AEs within the drugs’ AE reports with the fraction a drug contributes to the total pool of the AEs in the analyzed dataset, five drugs appear in both top 10 lists (see connections between upper and lower box plots in Fig. 4).

Six of the compounds with seizure alerts are thought to exert their wanted effects chiefly by other targets, and their effects on GABA_A_Rs are considered an off-target effect, namely, phenytoin, lamotrigine, amoxapine, acamprosate, memantine, and olanzapine. The tricyclic compound amoxapine and the structurally related drug olanzapine, which were identified as potential orthosteric GABA_A_R antagonists using the NeuroDeRisk IL Profiler, both appeared in the top 30 compounds for our FAERS-based alert profile. Thus, amoxapine was red-flagged by both the NeuroDeRisk IL Profiler and by pharmacovigilance data analysis and was therefore selected as the test compound for the experimental assays.

### In vitro and in vivo testing of the selected hit—amoxapine

**Fig. 5:**
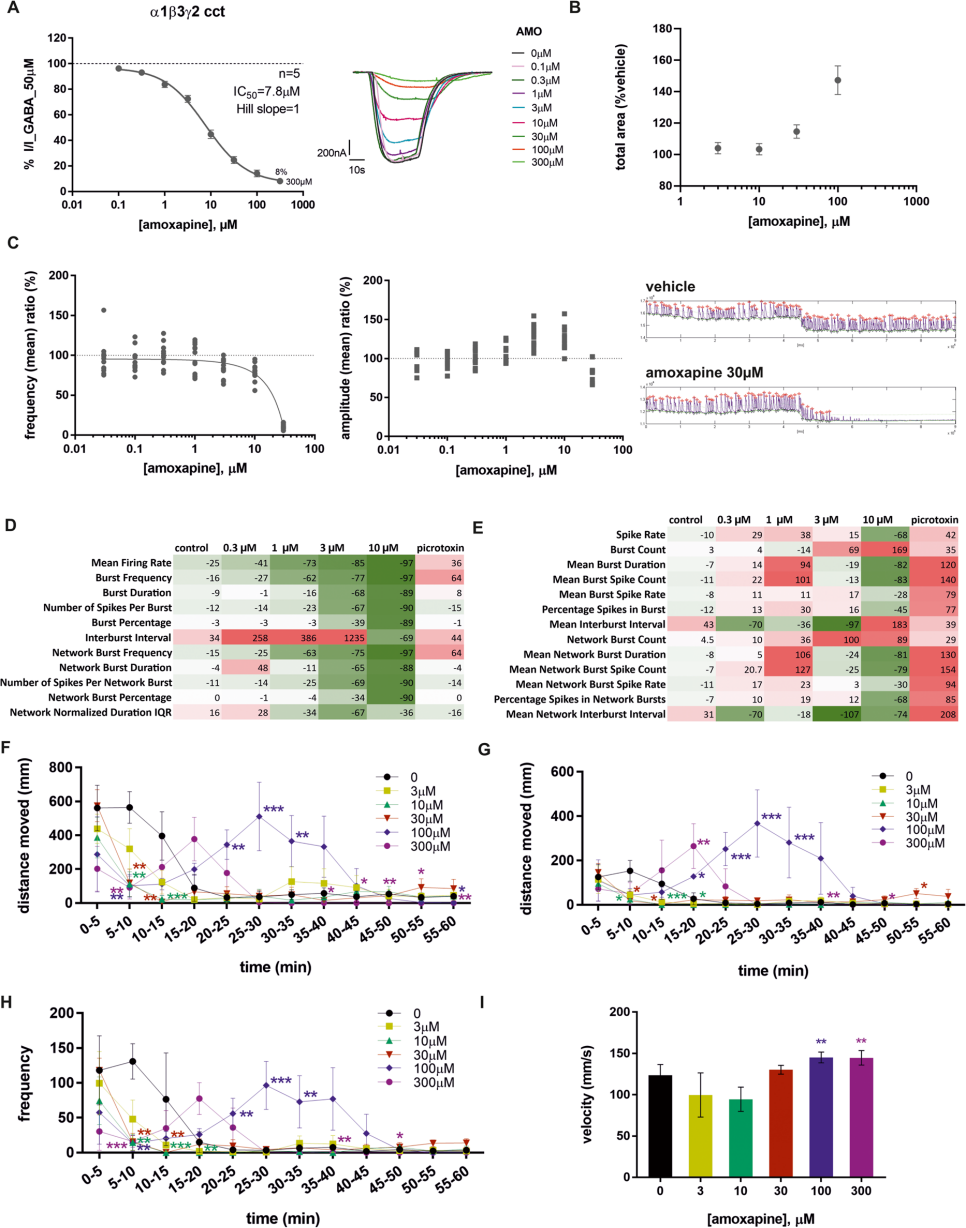
Amoxapine results from different assays. **A** Dose–response curve of amoxapine derived from TEVC recordings in *X. laevis* oocytes expressing α1β3γ2 concatenated GABA_A_R. Representative traces depicted on the right. Data depict mean ± SEM. **B** Mean (± SEM) concentration–response data summary of amoxapine (3, 10, 30, and 100 μM) effects on CA1 population spike area in rat hippocampal brain slices. Statistical testing was run on raw concentration data (dose: F_(4,36)_ = 22.85, *p* < .001; Dunnett’s post hoc: amoxapine 30 μM vs veh, *p* < .05; amoxapine 100 μM vs veh, *p* < .001). **C** Calcium oscillation evaluation showing the average of the amoxapine concentration effect on frequency then on amplitude (values depict % of vehicle ratio) and a sample traces recordings for vehicle and amoxapine for the highest amoxapine concentration, namely, 30 µM. **D** Microelectrode array (MEA) recordings from rat cortical neurons. **E** Microelectrode array (MEA) recordings with human iPS glutamatergic neurons in co-culture with human astrocytes. The values in the heat maps (**D**, **E**) are presented as maximum percentage of change vs baseline after a treatment duration of 1 h (**D**) or 10 min (**E**). Each value represents the mean of 3 (**D**) or 5 (**E**) wells. A red color shows an increase of the respective endpoint, while a green color shows a decrease of the respective endpoint value compared to baseline (0%). The intensity of the colors indicates the magnitude of the effects as shown below. **F**–**I** Effect of amoxapine treatment on zebrafish larvae locomotor measurements during 1-h exposure period using video-tracking system (*n* = 8 larvae/group): **F** Total distance moved in mm (TDM), median ± median absolute deviation (mad); *p ≤ 0.05, **p ≤ 0.01, and ***p ≤ 0.001 versus control group (2-way ANOVA type with repeated measure on factor time); **G** total distance moved at high velocity(> 20 mm/s) (TDMH) in mm median ± mad; *p ≤ 0.05, **p ≤ 0.01, and ***p ≤ 0.001 versus control group (2-way ANOVA type with repeated measure on factor time); **H** frequency at high velocity (> 20 mm/s) (FH), median ± mad; *p ≤ 0.05, **p ≤ 0.01, and ***p ≤ 0.001 versus control group (2-way ANOVA type with repeated measure on factor time); **I** maximal velocity (MV) in mm/s, median ± mad; **p ≤ 0.01 versus control group (Dunnett’s test after transformation on rank)

Amoxapine was first tested on recombinantly expressed α1β3γ2 GABA_A_R in *Xenopus laevis* oocytes, as this subtype is highly expressed in the brain. We observed a dose-dependent inhibition of GABA-induced current, which is nearly complete at 300 μM (Fig. 5A).

The effects of amoxapine (3, 10, 30, and 100 μM) were also tested on hippocampal CA1 population spike area, in a total of 11 slices from three animals (Fig. 5B). Application of increasing concentrations of the compound was associated with the appearance of multiple population spikes that resulted in an increase in the population spike (PS) area over a range of stimulus voltages. Quantitatively, this resulted in a concentration-dependent increase in PS area that was statistically significant at the higher test concentrations, as was for the control compound bicuculline (Supplementary Figure S13). PS area in the presence of 30 and 100 μM amoxapine (expressed as % control) was 115 ± 4.30% and 147 ± 9.15% of control, respectively.

Calcium oscillation measurements in mouse cortical neurons were also performed. The frequency of the calcium oscillation represents the network synchronicity activation. This frequency presents a pattern type, where changes upon compound exposure should correlate with the excitability of the network. The amplitude represents the power of the network that could be linked to cell number recruitment in the synchronized network, as well as cell calcium intensity of cellular response. Amoxapine had a global inhibitory effect with a unidirectional inhibitory effect on frequency but a dual effect on amplitude (first increasing at 3 and 10 µM, then decreasing and finally lost at 30 µM) of calcium oscillations in mouse cortical neurons (Fig. 5C).

Amoxapine was additionally tested in rat cortical neurons (Fig. 5D) and human iPSC neuron astrocyte co-culture (Fig. 5E) using a microelectrode array (MEA). In rat cortical primary cells, there was a clear dose-dependent decrease in parameters reflecting neuronal activity (mean firing rate, burst frequency, burst duration, number of spikes per burst, burst percentage, and interburst interval). Accordingly, parameters reflecting the network activity were also modulated by increasing concentrations of amoxapine, suggesting a perturbation in the excitation-inhibition balance of the network. These results are in qualitative agreement with the calcium oscillations observations in mouse cortical neurons. That is, the frequency of network synchronous activity (calcium oscillations and network bursts) is strongly downregulated by increasing amoxapine concentrations.

In hiPSC neurons, at low concentrations (< 10 µM), the drug increased neuronal activity, as seen by the increased spike rate and mean burst duration (Fig. 5E) indicating a seizurogenic pattern. At 0.3 and 1 μM, the magnitude of the positive values increased, while at 3 μM a, few endpoints start to decrease, while at 10 μM, most of the measured endpoints strongly decreased which could be indicative of an overall decrease of neuronal activity.

Additionally, amoxapine was also tested in vivo in zebrafish larvae from 3 to 300 μM (Fig. 5F–I). At the end of the 1-h video recording, mortality was only observed after treatment with amoxapine at 300 μM (2/8 animals) (Supplementary Table S5). Behavioral alterations were observed from 30 μM (erratic movements in 3/8 animals at 30 μM, 8/8 animals at 100 and 300 μM) (Supplementary Table S5). Moreover, when compared to the control group, statistically significant increases in all locomotor parameters automatically measured (TDM, FH, TDMH, and MV) were observed from 100 μM. In conclusion, under our experimental conditions, amoxapine was considered to have a convulsant activity in 7 dpf zebrafish larvae from 30 μM and to be toxic at 300 μM.

## Discussion

In this study, we examined known and tentative connections of GABA_A_R ligands with potential seizure liabilities in humans. One goal was to test a pipeline aimed at functional testing, which was performed with amoxapine. An additional goal was to align our workflow with the adverse outcome pathway (AOP) framework and to propose new AOPs that link GABA_A_R ligands with seizure-category AOs.

In order to use in silico methods for the prediction of molecular interactions at the scale of binding at GABA_A_Rs, we first analyzed structural data to generate a binding site inventory. Most structural knowledge has been accumulated for the highly conserved and highly expressed receptor subtypes, chiefly α1βγ2. Seizurogenic and convulsant drug effects have clearly been connected to this receptor population (Crestani and Rudolph 2015); thus, binding sites featured by this receptor subtypes were of major interest. For a few additional receptor subtypes, recent structure data is available (Miller et al. 2017; Sente et al. 2022) and will be useful in future studies to consider their impact as well. This study focused on the major subtype and its binding sites that might mediate seizure/convulsion AEs. To facilitate future use of this comprehensive structural analysis, we complemented it with a species comparison of all 19 mammalian subunits and the corresponding zebrafish sequences. This data can guide the selection of constructs and preclinical species to minimize the use of diverging binding sites for receptor subtypes of interest in preclinical assays. The so-called loop F region of several subunits features species differences (Supplementary Figure S5). The γ1 subunit, as an important example, is highly expressed in human amygdala and considered to be a target for anxiety disorders. It displays considerable species variation in the binding site forming segments of the ECD (Supplementary Figure S5). The binding site inventory, together with the subunit sequence data, can be used for downstream purposes such as the construction of pharmacophore models, or alternatively direct docking screens into structural models.

The NeuroDeRisk IL Profiler GABA_A_ models were used to profile 81 drugs to predict GABA_A_R molecular initiating events and supported the selection of amoxapine for experimental studies due to hitting two GABA_A_ antagonist seizure risk models. Experimental results of this study indicated that amoxapine shows high seizurogenic and convulsant potential. The NeuroDeRisk IL Profiler V1.0 was robust in that it correctly classified benzodiazepine drugs including their active metabolites, flumazenil, and the neurosteroid brexanolone. In addition, the GABA_A_ agonist models did not retrieve any of the 81 drugs, and none were expected to have orthosteric agonist activity at GABA_A_Rs. In terms of seizure risk involving GABA_A_ channel and orthosteric antagonist predictions, 27 drugs, including barbiturates and volatile anesthetics, hit at least one of the NeuroDeRisk IL Profiler GABA_A_ seizure risk models. While the risk of seizure has been reported in the drug package inserts and pharmacovigilance FAERS alerts indicated the potential seizure risk of these drugs, the mechanisms of action for seizure induction are not clear. Therefore, the NeuroDeRisk IL Profiler is a useful tool for nominating drugs and investigational compounds for experimental studies to further investigate AEs and related GABA_A_R pharmacology, as demonstrated with the experimental follow-up on amoxapine in this study. At the time the study was performed, the NeuroDeRisk IL Profiler V1.0 (2020) did not have models for barbiturate nor Z-drug GABA_A_ allosteric sites, which have been developed subsequently for V2.0 along with seizure/convulsions risk models involving other targets. Moreover, recent cryo-EM structural data (Zhu et al. 2022) will support further development of DMCM convulsant models related to GABA_A_R pharmacology and seizure/convulsions risk. This pilot study was limited in many regards: Existing structural data, coverage of binding sites, and the level of theory of the in silico parts are among the limitations. Thus, there remains an unmet need to establish a full inventory of confirmed binding sites in a comprehensive panel of receptor subtypes—a big task to be tackled by future research. Related proteins and homology modeling potentially bridge the gap for binding sites of high scientific interest (Bampali et al. 2022; Koniuszewski et al. 2022).

To obtain more information about the seizure risk of the 81 drugs, pharmacovigilance data was utilized to search for seizure/convulsion AE alerts and to correlate with the findings from the molecular initiating event (ligand binding) screens. Pharmacovigilance data is generally considered to be a useful source for alerts and testable hypotheses, in spite of the inherent limitation (Jeetu and Anusha 2010). Here, we used a dataset from the FAERS to analyze the associations of our test set of drugs with the AEs that can be reported on the basis of the MedDRA seizure/convulsion category. For drugs that were never approved in the USA, there are no or few records and thus no results (e.g., flutazolam, rilmafazone). For the majority of the tested compounds, a sufficient number of records is available and thus enables the use of appropriate statistical methods. This analysis not only results in an alert signal for amoxapine, but suggests that of the GABA_A_R targeting compounds that we analyzed, 43% are associated with seizure-category AEs and thus 57% are not, irrespective of ATC codes or chemotypes. The atypical benzodiazepine clobazam bears an ATC code as an anticonvulsant and yet appears to be firmly connected with seizure induction as shown in clinical trials (LundbeckLLC 2012, 2018).

Amoxapine was “red-flagged” by the NeuroDeRisk IL Profiler and the FAERS analysis. Thus, preclinical assays that cover the molecular, cellular, tissue, and organism scales were employed to investigate its effects on GABA_A_R-mediated inhibitory transmission and on neuronal firing patterns. To assess its predicted antagonistic properties, GABA-elicited currents were examined in recombinantly expressed α1β3γ2 receptors in the presence of amoxapine, and near complete inhibition was observed. Moreover, most results from the in vitro and in vivo assays presented in Fig. 5 are consistent with seizurogenic properties of amoxapine, except calcium oscillation and MEA data in rodent cortical neurons, which showed a decrease in neuronal activity. This different effect could be explained by species differences or cell type reactivity and/or differences in the experimental design of the assays. The MEA recordings in human iPSC neurons display a bicuculline-like pattern (Supplementary Table S6). The effects of amoxapine on zebrafish larvae are also consistent with seizure-like behavioral alterations. Together, these findings suggest that orthosteric antagonism of GABA_A_R contributes to the known seizure liability of this compound (Pisani et al. 1999; Squires and Saederup 1993). However, since amoxapine is a highly promiscuous molecule, contributions from other targets and pathways are entirely possible, in particular at higher dose levels. Given that individual cellular models including cultured cells display an increase in excitability in response to amoxapine, it is unlikely that this is an indirect effect, e.g., triggered by its action on catecholamine transporters, but rather indicative of an effect mediated by membrane proteins expressed by all tested cell types. This renders the inhibitory effect at GABA_A_Rs a likely candidate, at least at high brain levels of the lipophilic molecule (therapeutic concentrations 500 μg/mL (Boutelle 1980)).

Intriguingly, we get strong FAERS alerts for a number of compounds which are said to enhance GABA-mediated inhibitory currents (PAMs), and not only for those thought to act as functional antagonists or channel blockers. In fact, PAMs are overrepresented in the drug collection we tested, because they are widely used as tranquilizers, anxiolytics, and general anesthetics. Thus, FAERS data suggests that seizure liabilities are not only mediated by functional inhibitors of GABA_A_Rs, but also by compounds that enhance GABA-mediated inhibition. To provide an integrated view of these seemingly contradictory mechanisms, we frame effects induced by modulators in terms of the putative pathways that lead to the acute seizure induction (Perucca et al. 1998). It is important to distinguish between ortho and allosteric functional effects. For many ligands, it is unclear whether functional agonism/ antagonism is ortho- or allosterically mediated.

The induction of seizures by orthosteric antagonists (such as bicuculline) and channel blockers (such as picrotoxin) is a well-accepted phenomenon. However, the complete mechanism that links binding of the “inhibitors” bicuculline, picrotoxin, or other known GABA_A_R targeting convulsants to the seizure type that occurs in an organism is still far from fully clear. At a coarse grain scale, they are understood: Compounds that reduce GABA elicited currents and thus reduce inhibitory signals in the CNS lead to an increase in excitability. This straightforward mechanism is reflected in the “AOP 10” of the adverse outcome pathway model (https://aopwiki.org/wiki/index.php/Aop:10).

**Fig. 6:**
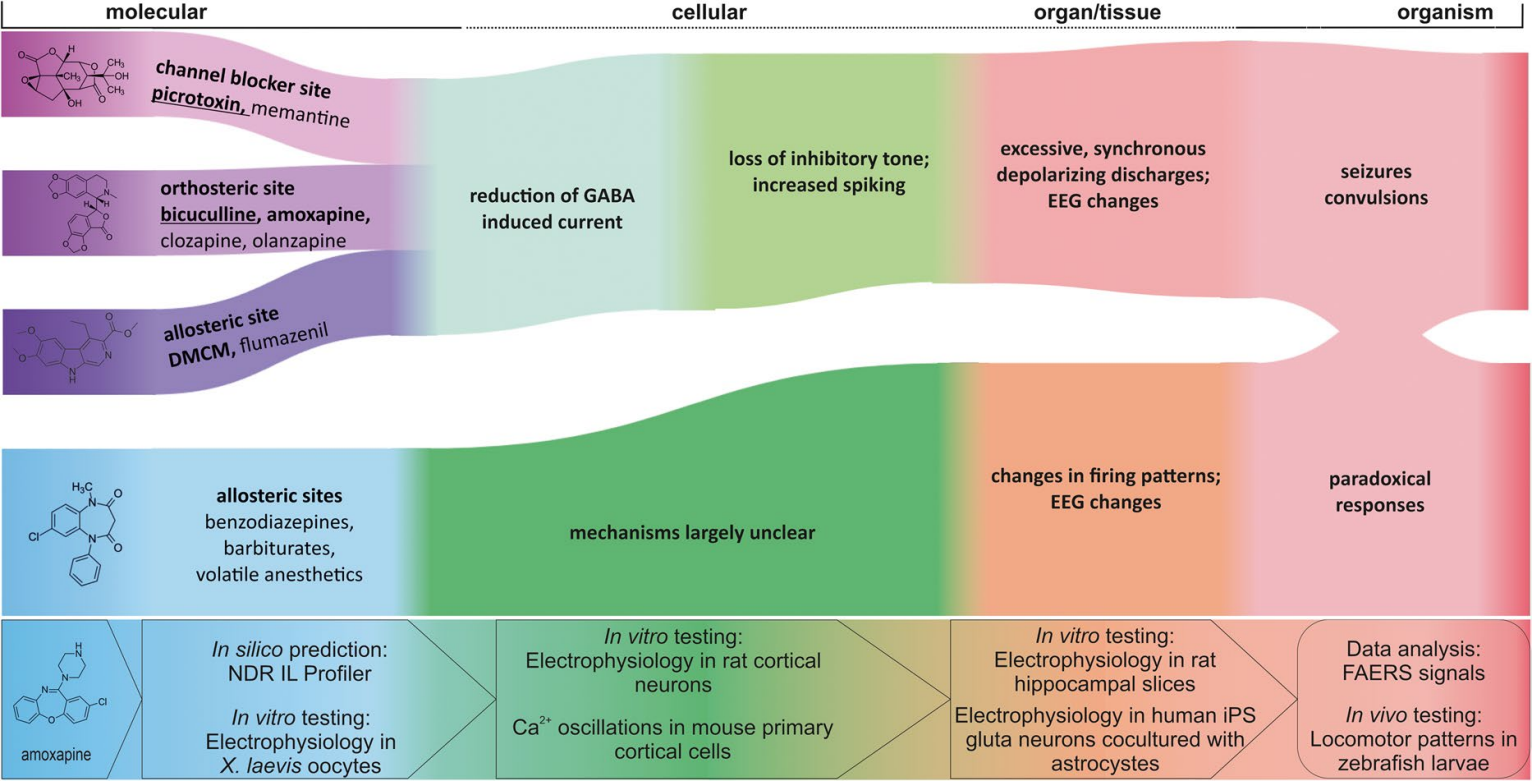
Candidate AOPs for several of the compounds we investigated in this study. The left set of events in blue/ purple hues represent the binding of ligands to their respective binding sites. Picrotoxin, bicuculline, DMCM, clobazam, and amoxapine are rendered in 2D as examples for the different binding sites and for the experimental pipeline. Different allosteric sites can mediate functional agonism and antagonism as well as NAM and PAM effects, which induce typically a change in GABA-elicited current. The proposal reflects a coarse grain model which requires further details to generate complete AOPs. Here, green hues indicate the late molecular and the cellular scales at which the ligand binding leads to changes in inhibitory transmission and then to changes in neuronal firing patterns. The red hues represent the organ and organism scales, at which changed neuronal firing patterns impact on network activity and thus on EEG and ultimately lead to organism responses such as seizures, convulsions, or paradoxical responses such as agitation that are often observed for GABA_A_R targeting “tranquilizers.” The assays that were used in this study at the molecular, cellular, tissue, and organism scales are integrated at the bottom of the graph. Ligand examples for each pathway are boldfaced and underlined for agents with known seizurogenic properties, boldfaced for strong candidates (meeting at least two criteria), and in standard font for the remaining examples

This AOP framework is helpful to structure known and candidate pathways and events that lead to drug-induced AEs and to optimize tools for their prediction and detection. Here, we propose candidates for additional AOPs that reflect distinct molecular initiating events or distinct mechanisms (Fig. 6). The proposed AOPs reflect the distinct molecular initiating events and are based on at least one of the following criteria: previous literature (DMCM, clozapine), results of the NeuroDeRisk IL Profiler screen (amoxapine, olanzapine), FAERS associations (amoxapine, olanzapine, PAM-type ligands such as some benzodiazepines and barbiturates). For amoxapine, our whole pipeline of in silico and experimental data strongly supports the proposed AOP as an orthosteric antagonist. Intriguingly, the FAERS search we conducted results in a seizures/convulsions alert signal albeit of moderate strength for amoxapine, but also for olanzapine. Consistent with its structural similarity with amoxapine, it also was a hit for the GABA_A_-antagonist model of the NeuroDeRisk IL Profiler. We thus place both compounds on a list of candidates for a “bicuculline-like” mechanism of seizurogenicity (Fig. 6).

Mechanistically much less clear is the acute seizurogenic effect elicited by antiepileptic compounds. This phenomenon is often attributed to the spectrum of paradoxical response to drugs which should be CNS depressant (tranquilizers, antiepileptics). At this time, to our best knowledge, it is completely unclear which molecular and cellular events drive them, but they are consistently also observed in preclinical species including zebrafish (McCarroll et al. 2019). Concerning possible mechanisms, it is known that compounds which are PAMs in some receptor subtypes can be NAMs in other subtypes (Sieghart and Savic 2018; Treven et al. 2018). This might explain the observation that they can be anticonvulsant under certain circumstances and convulsant in others. Disinhibition of target neurons under the control of interneurons may be another phenomenon that drives acute seizure induction by positive modulators. As only approximately half of the PAM-type compounds we examined here are connected with a seizure alert in the FAERS data, the question arises which differences in their molecular targets (i.e., GABA_A_R subtypes and/or additional targets) or mechanism of action drive this difference. Given the many receptor subtypes and the lack of functional data on these, this is an area of big unmet scientific need.

For future comprehensive models and the further refinement of toolchains for preclinical compound testing, some recommendations can be made guided by the AOP philosophy: At the molecular scale, a distinction between binding of a molecule to specific binding sites of defined receptor subtypes (the so-called molecular initiating event or MIE) and the changes in molecular function that is elicited by the binding should be made. The binding can be predicted if reliable structural or pharmacophore models are available and is commonly tested by standard displacement assays. High affinity ligands suitable for such assays are critically lacking for most allosteric binding sites of GABA_A_Rs, underscoring the need for in silico screening methods. New compounds should be subjected to comprehensive in silico screens for all allosteric sites that have been described. In this study, a single subtype was considered, and in future efforts, individual subtypes would need to be implemented.

For subsequent experimental testing of the molecular consequences of ligand binding to receptor activity, functional studies can be employed. Such methods (typically classical electrophysiology or voltage-dependent dye-based techniques) are sensitive to changes in channel function induced by ligands or by changes in GABA-elicited channel function. Here, the disadvantage is that the readout gives no indication of the binding sites and that functionally silent ligands also exist. Thus, multiple assays need to be combined in different ways if GABA_A_R-mediated activity should be ruled out with high confidence for a given compound. Functional testing is to be preferred due to the lack of radioligands for most of the allosteric sites depicted in Fig. 2 (Delaunois et al. 2020).

Functional changes such as an antagonist or NAM-induced reduction of GABA currents, or the PAM-induced increases in GABA currents, induce acute cellular effects that include loss or gain of neuronal inhibition, respectively, and thus in turn change in the spiking rate of affected neuron populations. These acute cellular effects elicited by a compound can be tested with a variety of assays that probe changes in neuronal excitability directly or indirectly. Larger numbers of compounds need to be tested to evaluate the assays we used here for their performance.

The AOP candidates presented in Fig. 6 intend to describe only acute seizure induction by compounds which reduce GABA-mediated ionotropic inhibition as the main, or contributing, mode of action, or with an unclear link between molecular effects and the seizure outcome. They do not reflect pathways that lead to seizures from prolonged exposure to the drug or to drug-induced changes in seizure threshold that lead to withdrawal seizures. The proposed preclinical pipeline of pharmacophore screening and functional assays is in fact designed for the prediction of acute GABA_A_R-mediated seizure liabilities, and it should work robustly for “inhibitory” effects. A big unmet need in preclinical research is the pharmacological profiling of compounds at the different GABA_A_ receptor subtypes. The observation that certain subtypes occur in extrasynaptic localization and chiefly mediate tonic inhibition has been made historically after the approval of many of the drugs that are described as PAM-type tranqulizers and anesthetics. Their role in toxicological effects is largely unclear. It has been noted that PAM-type compounds can accelerate desensitization, which may lead to a net reduction of charge transfer despite an enhanced but transient peak current (Kang et al. 2020; Liao et al. 2019). Thus, extrasynaptic receptors may actually contribute to the so-called paradoxical effects that are seen in susceptible individuals.

As we emphasized, the AOPs proposed here are very coarse-grained and lack detailed information on receptor subtypes. The experimental assays are intended to probe potential markers for the key events that connect the drug to the seizure, as depicted in Fig. 6. For the case of amoxapine, the majority of the tested assays generated a robust alert; thus, the pipeline can be benchmarked for more compounds that exert net inhibitory effects. For the seizure liabilities that are associated with positive modulators, new assays likely need to be developed to understand the molecular and cellular drivers.

There are thus several major unmet needs to be addressed by future research: One is the careful profiling of compounds in appropriate in vitro assays to capture functional effects more comprehensively. Recombinant studies are limited by the lacking knowledge on existing subtypes and the uncertainties concerning the subunit assembly rules in overexpressing cells versus physiological cells, chiefly neurons, and glia cells. Test systems that take advantage of diversely differentiated neuron- and glia-like cells based on hIPSCs might be a viable option. They do express multiple subtypes and thus cannot provide a molecular pharmacology—but the integrated responses to toxicants are likely to provide excellent alerts as they are a better approximation of the physiological cell response compared to overexpressing cells with isolated subtypes.

Another very important in vivo factor that is well described in the literature, but poorly integrated into preclinical toxicological workflows, are the neuroplastic adaptations of the mammalian brain to disease and drug exposure. It is known that epileptic seizures induce changes in expression in neurons (Brooks-Kayal et al. 1998), and thus, patients will experience dynamic changes to drug responses with disease progression. In the same vein, drugs themselves induce transcriptome changes, such as the well-known loss of high affinity benzodiazepine sites in the mammalian brain that occurs in response to benzodiazepine exposure. Thus, both therapeutic and unwanted responses to a drug change in the brain due to neuronal plasticity of multiple sources. This can potentially be captured by appropriate preclinical models and may require mammalian laboratory models for the calibration of appropriate cellular and organoid models that eventually should reduce and replace animal models.

## Conclusion

Structural studies are beginning to break new ground by providing 3D models which, in synergy with ligand-based in silico screening, could be very powerful to provide inexpensive “early alerts.” Substances which hit GABA_A_R-based pharmacophore models can be subjected to in vitro assays that test molecular change of function rather than binding to avoid the blind spots of allosteric sites with no corresponding radioligand. Cellular systems for in vitro assays can potentially be optimized to reflect species differences and to avoid a “rodent bias” in functional studies. Hybrid methods can take advantage of 3D structures and in vitro data to identify candidate sites, design experiments, and refine toxicophores. Ever faster computers enable in silico screening using a range of algorithms. Clinical data can be used in AI-supported analysis tools to detect unexpected connections between drugs and outcomes and between protein families and outcomes. Future efforts should be dedicated to the construction and testing of more detailed AOPs that can inform about receptor subtype contributions to drug effects.

## Methods

### Compound selection

Small molecules which target GABA_A_Rs were selected from four publicly available sources (Drugbank (https://go.drugbank.com/), Wikipedia (https://en.wikipedia.org/), Wikidata (WD; https://www.wikidata.org), and OpenTargets (https://www.targetvalidation.org/), without the intention to generate a complete list of all GABA_A_R targeting drugs, which would be beyond the scope. The selection was performed as follows:

Data extraction of Drugbank data was performed with a python script that retrieves all drugs from each GABA_A_R subunit record, identified by the encoding gene name (example: https://www.drugbank.ca/polypeptides/P14867 for GABRA1). Likewise, for OpenTargets, all drugs associated with any of the 19 subunits were retrieved via the encoding gene names.

Extraction of WikiData was done by the use of the 19 subunits with the Wikidata Query Service with a SPARQL query, and benzodiazepines from Wikipedia were obtained from (https://en.wikipedia.org/wiki/List_of_benzodiazepines, accessed on 09.07.2020).

All drugs and their SMILES codes are listed in Supplementary Table S3.

### In silico methods

#### Analysis of protein sequence and structural data

Sequences listed in the Supplementary Table S7 were acquired from either UniProt or NCBI Protein databases. Next, MUSCLE (Edgar 2004) was used to align the sequences per subunit isoform. Gap opening penalty was set to 2 and gap extension penalty to 0.2. The alignments were imported into the Python v3.9.7 (Rossum and Drake 2009) environment by using the biopython v1.78 package (Cock et al. 2009). Next, all non-human sequences were compared to their human orthologs by using a custom substitution matrix. The matrix was constructed by taking the blosum90 substitution matrix. The values for insertions and deletions were set to − 0.5 and the value for a gap in both sequences to 0. The rest of the values were normalized linearly to a range between 0 and 1. Using this matrix, every position on the non-human sequences was translated into a substitution score. To plot these scores, the alignment position had to be defined relative to the *x*-axis. As two graphs were generated, the sequences were divided into ECD and non-ECD parts. The alignment position numbers were created for each part differently. For ECD first the numbers of the longest sequence (GABRR1) were created. All other sequences had the second conserved cysteine in the cys-loop fixed to the same number as the longest one (GABRR1) (Supplementary Figure S6, vertical line). For all non-ECD sequences, the alignment position number starts at 1 (thus, at the beginning of the TMD). The annotated sequence segments were generated separately for the ECD and non-ECD parts as follows: The ECD segments (“loops”) A-G were used as in Supplementary Figure S4 for the human sequences, which is in line with the literature. For the TMD, the four membrane-spanning domains were used. All the selected segments were converted into the corresponding alignment positions. The position of annotations for both parts of the sequences was corrected separately based on the generation of the alignment position number described above. Substitution score sequences and annotations were plotted using matplotlib (Hunter 2007).

All experimental structures have been taken from the protein data bank (www.rcsb.org). Superposition and analysis of pocket candidates in the entire cys-loop family: PDBefold (http://www.ebi.ac.uk/msd-srv/ssm/) and MOE (http://www.chemcomp.com) were used to analyze structures. All molecular visualizations were done in MOE.

#### 3D-chemical structure dataset preparation and in silico profiling

The molecular structures of the 81 identified drugs were downloaded from the DrugBank website (Wishart et al. 2018) (https://go.drugbank.com/) as SD-files containing 2D atom coordinates. Stereoisomers were elaborated since some drug formulations have racemic mixtures. The individual drug SD-files were concatenated to a single file for processing by the software Flipper (Flipper 3.1.1.2: OpenEye Scientific Software, Santa Fe, NM. https://docs.eyesopen.com/applications/omega/flipper.html) (version 3.1.1.2, with default settings for all non-mandatory options) in order to enumerate all possible stereoisomers of drugs having one or more undefined stereocenters. The resulting output SD-file contained a total of 111 structures which were then subjected to the program OMEGA (Hawkins et al. 2010) (http://www.eyesopen.com, version 3.1.1.2, with default settings for all non-mandatory options) to generate a single low-energy 3D structure for each defined stereoisomer.

In silico 3D-profiling of 111 chemical structures was performed using the NeuroDeRisk IL Profiler V1.0 (Inte:Ligand, GmbH, Vienna, Austria; https://docs.inteligand.com/ndr/il-profiler/) on an Apple MacMini 3.2 GHz 6-core Intel Core i7 with macOS v.11.2.3 operating system using the bundled NeuroDeRisk Toolbox KNIME Analytics platform installation V1.0 (Inte:Ligand GmbH, Vienna, Austria). A maximum of 200 conformations of each query chemical structure were generated using iCon (Poli et al. 2018) as implemented in NeuroDeRisk IL Profiler V1.0. Default settings for conformation generation were used [Best; Max pool size: 4000; Timeout: 600 s; Max fragment build time (30 s); RMS threshold: 0.8 Angstroms; Energy Window: 20 kcal/mol; Enumerate rings, Enumerate nitrogens, Torsion drive, Generate coordinates from CT and Include input conf. were toggled on]. The following 8 GABA_A_R models were selected for the in silico profiling experiments: NDR-IL-GABA-A-Channel-LB-5, NDR-IL-GABA-A-gs-Agonist-6huj-4, NDR-IL-GABA-A-gs-Agonist-LB, NDR-IL-GABA-A-gs-Antagonist-6huk-3, NDR-IL-GABA-A-gs-Antagonist-LB, NDR-IL-GABA-A-Flumazenil-6d6t, NDR-IL-GABA-A-NSteroid-Pregnanolone-5o8f (NAM-steroid-8o8f), and NDR-IL-GABA-A-PAM-Diazepam-6hup. Profiling was done using the algorithms idbgen and iscreen from LigandScout (Wolber et al. 2006; Wolber and Langer 2005) (LigandScout V4.4 Expert. http://www.inteligand.com/ligandscout/) as implemented in the NeuroDeRisk IL Profiler V1.0. Explanations of the model names and models in the NeuroDeRisk IL Profiler are described in the documentation (https://docs.inteligand.com/ndr/models/). Screening settings included Retrieval mode: Stop after first matching conformation, Check exclusion volumes was toggled on while include non-matching molecules and Include non-matching pharmacophores were toggled off for the results shown in Table 1, and all options were toggled on for the Supplementary Figure S9.

#### Analysis of pharmacovigilance data

In order to retrieve FAERS records of adverse events that reflect seizures and convulsions, the list of possible reporting terms from the Medical Dictionary for Regulatory Activities (MedDRA) for the System Organ Class “Nervous system disorders” was used (raw results are provided as Supplementary Table S4). The list of seizure-AEs contains all the descendants of the MedDRA class SEIZURES (incl. subtypes):

(https://bioportal.bioontology.org/ontologies/MEDDRA?p=classes&conceptid=http%3A%2F%2Fpurl.bioontology.org%2Fontology%2FMEDDRA%2F10039911) accessed on 16.08.2022.

The data set for the FAERS analysis was used from Khaleel et al. (2022) which contains the FAERS data from Q1 2004 until Q3 2021. It is a curated data set in which also a disproportionality analysis was performed. From this data set, we extracted all records for the drugs from our drug list (81 drugs) and the MedDRA terms as defined above.

All records meeting commonly used criteria, specifically proportional reporting ratio (PRR) ≥ 2, the lower boundary of the CI 95% of the information component (IC025) had to be positive, and at least 5 reports in the dataset were used for further analysis. Except for the number of reports, these criteria correspond to those used in Andronis et al. (2020).

Visualization of the pharmacovigilance data was done using Python 3.10 with the plotly 5.5.0 and pandas 1.4.0 libraries. In Fig. 4, the percentages of the bar charts were calculated as follows:

For the upper bar plot:

Drug AE % = Drug AE reports / Drug total reports * 100.

For the lower bar plot:

AE % = Drug AE reports / Total reports for seizures incl subtypes (In total 46,285 reports) * 100.where drug AE reports is the total number of reports for a given drug/AE combination that pass the statistical criteria. For visualization of the bar chart (Fig. 4), we used only the adverse events that cover approximately 98% of all seizure-related terms (Supplementary Figure S11). In the Supplementary Figure S11, the pie chart was calculated as followed:

Pie chart AE % = Total reports for each AE / Total reports for seizures incl subtypes (In total 46,285 reports) * 100.

Supplementary Figure S10 shows the workflow of the FAERS analysis.

### Experimental methods

#### Two-electrode voltage clamp (TEVC) recordings in *Xenopus laevis* oocytes

Stock solution and buffers were prepared as described in Simeone et al. (2017). For the electrophysiological experiments, GABA was dissolved in NDE buffer [96 mM NaCl, 5 mM HEPES–NaOH (pH 7.5), 2 mM KCl, 1 mM MgCl_2_, 1.8 mM CaCl_2_]. Amoxapine was dissolved in DMSO with a stock concentration of 30 mM.

In order to generate mRNA, all constructs were linearized, transcribed, and purified as described previously (Simeone et al. 2017). For the microinjection, the RNA of the α1β3γ2 concatenated receptor combination was mixed at a 1:1 ratio. The approach used for subunit concatenation of α1β3γ2 GABA_A_R has been described previously (Simeone et al. 2019). The dual (γ2β3) and triple (α1β3α1) constructs were injected at a ratio of 1:1, with a final concentration of 70 ng/μl.

Healthy defolliculated oocytes were injected with an aqueous solution of mRNA with a Nanoject II (Drummond). The injected oocytes were incubated at 18 °C (ND96 + antibiotic) for 3–4 days before recording. Electrophysiological recordings were performed as specified in Simeone et al. (2017)*.* GABA concentration amounting to ~ 30% of maximum GABA currents was used, namely, 50 μM GABA. To ensure the incorporation of the γ2 subunit, diazepam was applied at the end of each measurement (~ 200% at 1 µM). All recordings were performed at room temperature at a holding potential of − 60 mV using a Dagan TEV-200A two-electrode voltage clamp (Dagan Corporation) and a Turbo Tec-03X npi amplifier.

#### In vitro electrophysiological experiments using the multi-electrode (MEA) technology and human iPS glutamatergic neurons in co-culture with astrocytes differentiated from human iPS cells

All cells were delivered by Fujifilm Cellular Dynamics Inc. FCDI, 525 Science Dr. Madison, WI 53,711. For all the experiments described below, ICell Gluta Neurons (lots 103,311, 104,925, and 105,990) and ICell Astrocytes (lots 105,152, 105,993, and 104,345) were used. Neurons were tested between 14 and 21 days in vitro. Amoxapine (Sigma-Aldrich) was diluted in DMSO to reach a final DMSO concentration of 0.1% DMSO in the medium and was tested at 0.3, 1, 3, and 10 µM. Picrotoxin 10 μM was used as a positive control. The effects of amoxapine are measured in 4 wells per concentration. The consumables used were the following: 50% PEI solution (Sigma-Aldrich), borate buffer 20 × (Thermo Fisher Scientific), Laminin (Sigma-Aldrich), BrainPhys Neuronal Medium (STEMCELL Technologies), N2 Supplement 100 × (Thermo Fisher Scientific), Penicillin–Streptomycin, 100 × (Bioconcept), iCell Neural Supplement B, M1029 Lot: 103,721, 105,466, iCell Nervous System Supplement, Lot: 105,169, 105,937, 104,455, Multichannelsystems 96-well Multiwell Plates for Multiwell Systems (96W700/100F-288).

All wells were coated with 0.07% PEI solution the day before plating the cells. For this, 80 µl PEI solution was added to the wells of a 96-well plate and incubated for 1 h at 37 °C. Thereafter, the PEI solution was removed, and the wells were rinsed twice with 200 µl D-PBS -/- and twice with 200 µl sterile Omnipure water. The plates were air-dried overnight.

Media were prepared as follows: Complete BrainPhys Medium for 100 mL: 95 mL BrainPhys Neuronal Medium, 2 mL iCell Nervous supplement B, 1 mL iCell Nervous supplement, 1 mL N-2 Supplement, 1 mL Penicillin–streptomycin, 100 uL Laminin. Dotting medium for 1 mL: 100 µl Laminin + 900 µl compl. Medium. Fill-up medium (for 1 96-well plate): 775 µl Laminin + 24 mL compl. medium.

Thawing and plating was performed by transfer of cells in a 50 mL tube (1 for the Astrocytes, 1 for the GlutaNeurons) and addition of 1 mL complete BrainPhys medium 1 drop/2 s, add 8 mL complete BrainPhys medium 1 drop/second, centrifugation at 400 g 5 min. Adding of dotting medium: for 240,000 cells/8 µL (Gluta Neurons). Adding of dotting medium: for 27,000 cells/3 µL (Astrocytes). Cell suspensions: GlutaNeurons und Astrocytes mixed 102,000 GlutaNeurons/5 µL and 13,800 Astrocytes/5 µL- ratio ~ 12% 0.5 µL cell suspension/well added directly to the electrode area of the wells. Incubated for ~ 1 h at 37 °C, 5% CO2, and > 90% humidity, add the filling medium (200 µL). Medium change every other day for 14–21 days.

Regarding data acquisition, all recordings were executed with a Multiwell-MEA System (Multichannelsystems, MCS). The MEA system was pre-warmed to 37 °C, and plates were covered with 5% CO2 during the whole measurement. 96-well plates were placed into the device, and the cells were left to rest for 10 min. The electrical activity of the co-cultures was measured with a sampling rate of 20,000 Hz for 5 min intervals before compound addition and 2, 10, and 60 min after compound addition. The following filters were used: High-Pass: 2nd Order Butterworth filter (10 Hz) and Low Pass: 2nd Order Butterworth filter (3500 Hz). A burst was defined to be > 50 ms with > 10 spikes.

The experimental endpoints analyzed were spike count, spike rate, burst count, mean burst duration, mean burst spike count, mean burst spike rate, percentage spikes in bursts, mean interburst interval, network burst count, mean network burst duration, mean network burst spike count, mean network burst spike rate, percentage spikes in network burst, and mean network interburst interval.

All analysis was done with Multichannel systems Multiwell Analyzer software Version 2.0. Raw data were exported for further analysis in Excel. The values measured by each electrode were normalized with predose values of the same electrode, and the percentage of change was calculated for the 5 min intervals at 2 min, 10 min, and 60 min after compound administration. All responses were corrected for the time-matched vehicle control values. The maximum effect during the time period of 60 min is reported.

#### Calcium oscillations in mouse primary cortical cells

For all the experiments described below, C57BL/6 J mice were used. Neonates from 0–1 day old were used. On the day of plating, mice were euthanized, then the brain was extracted for microdissection. Cortex was extracted under a binocular microscope.

After tissue extraction, cells were harvested under the biological safety cabinet. For enzymatic dissociation, tissues were washed with neurobasal medium (2–8 °C) and resuspended in 6 mL Neurobasal then incubated with 300 µL of trypsin (10 ×) and 150 µL of DNase (400 KU/mL). After 2–5 min of incubation at 37 °C, then 2 washes with Neurobasal + 1% Bovine calf serum (BCS) (37 °C). Mechanical dissociation: 3 mL addition of NBC + 1% BCS (37 °C) then 10 gentles up and down aspiration with P1000 for dissociation.

For cell plating, after 8 min centrifugation, was done at 1000 rpm. Supernatant was removed and the cells were resuspended with Neurobasal medium with N2 and B27 + 1% BCS (37 °C) and counted. Cells were plated in Greiner polylysine 96 microplate black/clear bottom at high density (80,000 cell/well). After 30 s centrifugations at 1000 rpm, the plates were placed in an incubator at 37 °C, 5% CO_2_, and 80% humidity.

The culture medium was renewed 24 h after cell plating and then every 2–3 days with a visual inspection of cells under the microscope.

 A vehicle free of protein was used to avoid protein binding in the medium. This means that amoxapine was tested at their nearest of their free fraction, but there were some solubility limitations. This limitation was tested by the solubility assay in the vehicle that is an HBSS medium with 0.1% of DMSO final.

At neuronal maturity 16 days in vitro + / − 1 days, the medium is removed, and cells are incubated with a specific loading medium (100 µl/well). This medium is composed of HBSS medium, a saline solution containing 2 mM of Mg^2 +^ . In this solution, a calcium probe (Cal520 ATTBioquest®) is added (1/200^th^) with Powerload (Thermofisher®) and Probenecid (Thermofisher®) to facilitate probe entry and maintenance in the cells, for 1 h at 37 °C, in 5% CO_2_. Plates are then washed 3 times with HBSS medium containing 0.1 mM of Mg^2 +^ . Plates are incubated for 10 min at 37 °C, in 5% CO_2_ between each wash.

Meanwhile, a plate containing amoxapine diluted in HBSS containing 0.1 mM Mg^2 +^ and 0.1% DMSO (Sigma-Aldrich®) is prepared. A range of concentrations is tested for amoxapine and vehicle (corresponding to HBSS containing 0.1 mM Mg^2 +^ and 0.1% DMSO) and positive reference (4 Aminopyridine or 4-AP at 30 µM) are also added on this “product plate.” The results for 4-AP are 146% for frequency and 95% for amplitude.

For fluorescence detection, µCell FDSS instrument (Hamamatsu®) is used, a kinetic plate reader with an integrated dispensing head and imaging-based detector. Ca^2 +^ flux oscillations variations are detected live for 15 min, the injection starting at 7 min 30 s after the beginning of the recording. During this recording, two parameters are measured in each well: oscillation frequencies and amplitudes (before and after compound injection). Data output for frequency and amplitude are obtained with Waveanalysis® software from Hamamatsu© and are given in Reference Fluorescence Unit (RFU) and used for data analysis.

Raw data from µCell FDSS are processed with Waveanalysis® software from Hamamatsu© in combination with a template of product plate scheme. The software enables counting the number of peaks detected and their height. Data output of oscillations frequency and amplitude for each well after compound injection are reported as ratio values, i.e., normalized to 100% in comparison to values before injection (initial state) per well and versus vehicle control. Data obtained for the test article at different concentrations can be used for EC50 estimation using speed HMTS, a software designed by Sanofi with the main objective of drawing dose–response fitting for screening throughput activities. EC50 can be estimated for each parameter and for each compound with several tested concentrations.

#### Electrophysiological recordings from rat hippocampal brain slices

Activity in rat hippocampal brain slices was assessed as previously described (Easter et al. 2007). Briefly, Han Wistar rats (6–12 weeks, male or female) were anesthetized with halothane and killed by cervical dislocation. The brain was quickly removed and placed in ice-cold artificial cerebrospinal fluid (aCSF). Parasaggital hippocampal brain slices were prepared and mounted in a Slicemaster multi-slice recording system (Stopps et al. 2004). For each slice, a bipolar stimulating electrode (Scientifica UK Ltd) was placed in the CA2 stratum radiatum and the Schaffer collateral pathway stimulated at 30 s intervals using constant current pulses (0.03 ms duration) of varying amplitudes. Evoked PS were recorded using a borosilicate glass microelectrode placed within the CA1 cell body layer. Clampex (Axon Instruments, version 9.2) was used to control the amplitude and frequency of the voltage stimulus and to record the evoked response at the recording electrode. The assay has been pharmacologically validated as shown in Easter et al. (2007) and in the supplementary information with bicuculline (Supplementary Figure S13). Data were expressed as % difference versus vehicle. For statistical testing, raw concentration data were fit with a linear mixed effects model in R using the package lme4 (version 1.1–31). Models were specified with a fixed effect of dose, and a random effect of slice nested within the animal to account for the hierarchial structure of the experiment. On occasion, there were multiple technical replicates per slice (i.e., a single slice was tested twice). Where this occurred, the data across the replicates was summarized as a mean value per slice. Where there was a significant main effect of dose as indicated by analysis of variance using Sattherwaite’s method for degrees of freedom estimation, a Dunnett’s posthoc analysis was carried out to compare all doses to vehicle using the R package emmeans (version 1.8.3). A significance threshold of *p* < 0.05 was used throughout. Model fit was evaluated via visualization of the residuals. For the bicuculline data, there was clear non-linearity in the residuals, and the raw data were log transformed to improve model fit.

#### In vitro functional assessment using microelectrode arrays (MEA) in rat cortical neurons

The experimental protocol was similar to the previous publication (Bradley et al. 2018). Cryopreserved rat cortical neurons (co-culture of neurons and astrocytes from QBM Biosciences) were thawed and slowly diluted with neurobasal medium supplemented with B27, L-glutamine, and penicillin–streptomycin (NB/B27). Cells were plated onto the MEA 48-well plates resulting in 75,000 cells per well. The plates were maintained in a humidified incubator at 37 °C with 5% CO_2_ for 14–18 days with medium (NB/B27) changes 3 times a week before experimental procedures were performed. For treatment, 250 μL of the medium was removed from each well, dispensed into the corresponding wells of a sterile 48-well plate, and mixed with vehicle (DMSO 0.2%), positive control, or amoxapine at four concentrations in triplicate. The formulations were carefully added back to the corresponding wells of the MEA and incubated for 1 h at 37 °C. Picrotoxin (PTZ) 10 μM was used as the positive control.

All recordings were obtained with Axion Biosystems Maestro MEA system.

Prior to baseline recordings, an assessment of activity was performed for each well of the 48-well plate by observing spontaneous spike activity. Wells with no or sparse activity were eliminated from the experiment. Typically, firing rates of 20 spikes/second (well averages) or more are acceptable.

After a 3-min equilibration time, baseline recordings of approximately 15 min were obtained immediately before the addition of treatment compounds and controls. Following a 1-h incubation at 37 °C with compounds, another 15-min recording was obtained after a 3-min equilibration time. Electrodes with 100 or more spikes over the 15-min post-treatment period (~ 7 spikes/min) were determined to be “active,” and only wells with 5 or more active electrodes were used in the final analysis. If a treated well fell below the activity threshold due to compound effect, only spike count was determined and reported. All other parameters were not calculated.

The following 12 endpoints, calculated using custom MATLAB (MathWorks, Natick, MA) scripts, were reported: firing rate (spikes/second), burst rate (bursts/sec), number of spikes in burst, percent isolated spikes, coefficient of variation (CV) of the inter-spike intervals (ISI) (indicator of the burstiness of the spike train), normalized burst duration IQR (indicator of burst duration regularity), burst duration (s), interburst interval (s), mean of ISI-distance (synchrony endpoint), normalized mean absolute deviation (MAD) burst spike number (indicator of statistical dispersion of the spikes in bursts), median/mean ISI (indicator of spike organization within bursts/burst deterioration), and median ISI.

Firing rate and burst rate reflect neuronal activity, while the other parameters reflect network burst structure and organization.

Data were expressed as % difference versus vehicle. Statistical analysis was conducted using GraphPad Prism 9.2. For each parameter, an ordinary one-way ANOVA was performed to determine if there was a difference among the means of the different groups. A Tukey post hoc multiple comparison was then conducted to assess statistical significance between the control group and each of the dose levels. A heatmap was built for each tested drug, to visualize the magnitude of change in each parameter.

#### Locomotor patterns in zebrafish larvae

This model was adapted from an experiment published by Berghmans et al. (2007). The experiments were performed in an Association for Assessment and Accreditation of laboratory Animal Care (AAALAC)-approved animal facility at approximately 28 °C on a 14-h/10-h light/dark cycle. For all the experiments described below, zebrafish larvae of AB-strain were used. At 6 days post-fertilization (dpf), the larvae were received at the laboratory and were maintained in an E3 medium (13.7 mM NaCl, 0.537 mM KCl, 0.025 mM Na_2_HPO_4_, 0.044 mM KH_2_PO_4_, 1.3 mM CaCl_2_, 1.0 mM MgSO_4_, 2 mM HEPES buffer pH = 7.25). On the test day (7 dpf), larvae were individually placed in a 48-well multiplate containing 450 µL of E3 medium for a few hours.

Locomotor pattern assay: before testing, baseline locomotion was recorded. The plate was subjected to a 10-min baseline assessment period to ensure that each animal was correctly tracked and that there was no abnormal swimming behavior before treatment.

Then each larva was exposed to, by adding 50 µL, amoxapine (3, 10, 30, 100, and 300 μM) or vehicle (final concentration in the well 0.5% DMSO in E3 medium). For each solution, pH was adjusted between 7.0 and 7.4.

Then, larvae were video monitored, and their movements were traced for 1 h (using DanioVision, EthoVision XT 14.0, Noldus). Locomotor parameters collected were analyzed by using automated tracking (quantitative analysis). Abnormal swimming behavior as circling or erratic movements were quantified by a visual scoring of the occurrence of behavioral alterations videorecording (qualitative analysis) (Supplementary Table S5).

For the quantitative analysis, the locomotor parameters collected were the total distance moved (TDM in mm), the total distance moved at high velocity (TDMH > 20 mm/s), the maximal velocity (MV in mm/s), and the frequency at high velocity (FH). A 1-h global analysis was done for each parameter, and a kinetic analysis (1 h divided into a 5-min period) was added for TDM, TDMH, and FH.

For all parameters TDM, TDMH, FH, and MV, the median and the median absolute deviation (mad) were calculated. The level of statistical significance was *p* ≤ 0.05.

A decrease in the locomotor parameters was not considered in this model evaluating only proconvulsant activity.

For the analysis of the 5-min periods, for each compound, a statistical analysis was performed on TDM, TDMH, and FH for each concentration level of amoxapine versus the control group using an ANOVA-TYPE test. A complementary analysis was performed at a fixed time followed by a test difference of each dose level versus the control group (contrast analysis with a Bonferroni-Holm correction), when the complementary analysis reached 10%.

For the analysis of the 1-h period, for MV, TDM_1-hour, TDMH_1-hour, and FH_1-hour, an ANOVA test after rank transformation followed by a Dunnett test was performed to compare the treated groups with the control group.

### Supplementary Information

Below is the link to the electronic supplementary material.

Supplementary file1 (PDF 19.7 MB)

## Data Availability

The data that support the findings of this study are available from the corresponding author upon reasonable request.

## Abbreviations

AAALAC: Association for assessment and accreditation of laboratory animal care
AO: Adverse outcome
AOP: Adverse outcome pathway
AE: Adverse event
Cryo-EM: Cryogenic electron microscopy
CV: Coefficient of variation
DI: Diazepam insensitive
DMSO: Dimethylsulfoxide
DS: Diazepam sensitive
DPF: Days post fertilization
EC: Effective concentration
ECD: Extracellular domain
FAERS: FDA adverse event reporting system
FH: Frequency at high velocity
GABA: γ-Aminobutyric acid
GABAARs: γ-Aminobutyric acid type A receptors
GABRR: γ-Aminobutyric acid type A receptor gene
HBSS: Hank’s balanced salt solution
hiPSC: Human induced pluripotent stem cells
ICD: Intracellular domain
IC025: Information component
INDEL: Insertion/deletion
IQR: Normalized burst duration
ISI: Inter-spike interval
KE: Key events
L(Ant): Ligand antagonist
LB: Ligand based
L(ChB): Ligand channel blocker
L(NAM): Ligand negative allosteric modulator
L(PAM): Ligand positive allosteric modulator
MAD: Mean absolute deviation
MEA: Multielectrode array/microelectrode array
MedDRA: Medical dictionary for regulatory activities
MIE: Molecular initiating events
MV: Maximal velocity
NAM: Negative allosteric modulator
PAM: Positive allosteric modulator
PBS: Phosphate buffer saline
PDB: Protein database
PEI: Polyethyleneimine
PIP2: Phosphatidylinositol-4,5-bisphosphate
PDB ID: Protein database identifier
pLGIC: Pentameric ligand-gated ion channel
PQ: Pyrazoloquinolinone
PRR: Proportional reporting ratio
PS: Population spike
PTZ: Picrotoxin
RPM: Rotations per minute
SF: Supplementary figure
SMILES: Simplified molecular input line entry system
SI: Supplementary information
TDM: Total distance moved
TDMH: Total distance moved at high velocity
TMD: Transmembrane domain
TEVC: Two-electrode voltage clamp
WD: Wikidata
ZI: Zolpidem insensitive
ZS: Zolpidem sensitive
( +): Principal site of the interface
(-): Complementary site of the interface
4-AP: 4-Aminopyridine
