# Supplementary material for: GABAA receptor-mediated seizure liabilities: a mixed-methods screening approach
Source: Cell Biol Toxicol. 2023 Apr 24;39(6):2793–819. doi: 10.1007/s10565-023-09803-y (PMC10693519; doi:10.1007/s10565-023-09803-y)
Supplement: Supplementary file 1 — Supplementary file1 (PDF 19.7 MB) [file 10565_2023_9803_MOESM1_ESM.pdf]

# Supplementary Information

## $GABA_A$ receptor-mediated seizure liabilities: A mixed-methods screening approach

Konstantina Bampali (1), Filip Koniuszewski✉ (1), Florian D. Vogel (1), Jure Fabjan (1), Christos Andronis (2), Eftychia Lekka (2), Vassilis Virvillis (2), Thomas Seidel (3), Annie Delaunois (4), Leandro Royer (4), Michael G. Rolf (5), Chiara Giuliano (6), Martin Traebert (7), Gautier Roussignol (8), Magali Fric-Bordat (8), Ludmilla Mazelin-Winum (8), Sharon D. Bryant (9), Thierry Langer (3), Margot Ernst✉* (1)

(1) Department of Pathobiology of the Nervous System, Center for Brain Research, Medical University Vienna, Spitalgasse 4, 1090 Vienna, Austria

(2) Biovista, 34 Rodopoleos Street, 16777, Athens, Greece

(3) Department of Pharmaceutical Sciences, Division of Pharmaceutical Chemistry, University of Vienna, Josef-Holaubek-Platz 2, 1090 Vienna, Austria

(4) UCB Biopharma SRL, Chemin du Foriest, Braine-l'Alleud, Belgium

(5) Astra Zeneca, R&D Biopharmaceuticals, Pepparedsleden 1, 431 83 Mölndal, Sweden

(6) Astra Zeneca, R&D Biopharmaceuticals, Fleming Building (B623), Babraham Research Park, Babraham, Cambridgeshire CB22 3AT, United Kingdom

(7) Novartis Institutes for Biomedical Research, Fabrikstrasse 2, CH-4056, Basel, Switzerland

(8) Preclinical safety, Sanofi R & D, Montpellier, France

(9) Inte:Ligand GmbH, Mariahilferstrasse 74B/11 1070 Vienna, Austria

*Corresponding author – main contact: margot.ernst@meduniwien.ac.at, phone number: +43 1 40160 34065

✉These authors are shared corresponding authors

| Nr | PDB ID | Protein | Ligand | Subunits | Puthenkalam et al., 2016 | References |
|---|---|---|---|---|---|---|
| 1 | 5AFJ | AChBP | fragment 1 | α7 | 1 | Spurny et al., 2015 |
| 2 | 5VDH * | Glycine | AM-3607 | α3+α3- | - | Huang et al., 2017 |
| | 6HJX * | ELIC | 2-Ethanesulfonic acid | | - | Henault et al., 2019 |
| 3 | 6HUP | GABA-A | GABA | β3+α1- | Site 1 | Masiulis et al., 2019 |
| | | | Diazepam | α1+γ2- | | |
| | 6HUO | | GABA | β3+α1- | | |
| | | | Alprazolam | α1+γ2- | | |
| | 6HUK * | | Bicuculline methochloride | β3+α1- | | |
| | 6A96 | | GABA | β3+α5- | | Liu et al., 2018 |
| | 6DW0, 6DW1 | | GABA | β1+α1- | | Phulera et al., 2018 |
| | 6DW0, 6DW1 | | GABA | α1+β1- | | |
| | 6D6T, 6D6U | | GABA | β2+α1- | | Zhu et al., 2018 |
| | | | Flumazenil | α1+γ2- | | |
| | 4COF | | Benzamidine | β3+β3- | | Miller and Aricescu., 2014 |
| | 6QFA | | Histamine | β3+β3- | | Uchanski et al., 2021 |
| | 7PC0 | | alpha-Cobratoxin | β3+α1- | | Kasaragod et al., 2022 |
| | 7QN9, 7QN8, 7QNC | | histamine | β3+β3- | | Sente et al., 2022 |
| | 7QND, 7QNC | | gaboxadol | δ+β3- | | Sente et al., 2022 |
| | 7QNB | | GABA | β3+γ2- | | Sente et al., 2022 |
| | 7QNE | | Ro15-1413 | α1+γ2- | | Sente et al., 2022 |
| | 4TWF, 4TWD | ELIC | memantine, bromomemantine | | | Ulens et al., 2014 |
| | 4F8H * | GLIC | Ketamine | | Site 2 | Pan et al., 2012a |
| 4 | 2YN6 * | ELIC | Ba-Atom | | Site 3 | Zimmermann et al., 2012 |
| 5 | 5LG3 * | ELIC | Chlorpromazine | β1- | Site 5 | Nys et al., 2016 |
| | 5LID | | Bromopromazine | | | |
| | 3ZKR | | Br- atom | | | Spurny et al., 2013 |
| 6 | 5MUR * | GLIC | Propofol | | Site 8 | Sauguet et al., 2018 |
| | 3P4W * | GLIC | Desflurane | | Site 8 | Nury et al., 2011 |
| L | 6D6T, 6D6U * | GABA-A | Cholesterol | β2+ | - | Zhu et al., 2018 |
| 7 | 6HUP * | GABA-A | Diazepam | β3+α1- | Site 7 | Masiulis et al., 2019 |
| | 5MVM | GLIC | Propofol | | | Fourati et al., 2018 |
| | 4HFE | GLIC | Ethanol | | | Sauget et al., 2013 |
| | 6X3V | GABA-A | Etomidate | β2+α1- | | Kim et al., 2020 |
| | 6X3T | GABA-A | Propofol | | | |
| | 6X3W | GABA-A | Phenobarbital | | | |
| | 6X3X | GABA-A | Diazepam | β2+α1-, γ2+β2- | | |
| | 5VDH * | alpha-3 Glycine | Avermectin | α3+α3- | | Huang et al., 2017 |
| L | 6D6T, 6D6U * | GABA-A | Cholesterol | β2+α1- | | Zhu et al., 2018 |
| L | 6D6T * | GABA-A | Cholesterol | α1- | - | Zhu et al., 2018 |
| L | 6D6T | GABA-A | Cholesterol | α1+β2- | - | Zhu et al., 2018 |
| | 5OSA * | GLIC-GABAAR alpha-1 chimera | Cholesterol Hemisuccinate | α1+α1- | - | Laverty et al., 2017 |

| | | | | | | |
|---|---|---|---|---|---|---|
| L | 6D6T, 6D6U * | GABA-A | Cholesterol | β2+α1- | Site 7 | Zhu et al., 2018 |
| L | 6D6T * | GABA-A | Cholesterol | α1- | | Zhu et al., 2018 |
| L | 6D6T | GABA-A | Cholesterol | α1+β2- | | Zhu et al., 2018 |
| | 5OSA * | GLIC-GABAAR alpha-1 chimera | Cholesterol Hemisuccinate | α1+α1- | | Laverty et al., 2017 |
| 8 | 6HUG, 6HUJ, 6X40 * | GABA-A | Picrotoxin | αβαγβ | - | Masiulis et al., 2019, Kim et al., 2020 |
| | 5MUO * | GLIC | Propofol | | - | Sauget et al., 2018 |
| L | 6D6T,6D6U * | GABA-A | Cholesterol * | β2+ | Site 10 | Zhu et al., 2018 |
| | | | | α1+ | | |
| 9 | 5OSC * | GLIC-GABAAR alpha-1 chimera | Pregnenolone Sulfate * | α1+ | | Laverty et al., 2017 |
| L | 3EAM * | GLIC | Lipid * | | | Bocquet et al., 2008 |
| 10 | 6CDU * | chimeric human alpha-1 GABAA | Alphaxalone * | α1+α1- | overlap with Site 9 | Chen et al., 2018 |
| | 5O8F * | chimeric beta-3 alpha-5 GABAA | Pregnenolone * | α5β3 | | Miller et al., 2017 |
| | 5OSB * | GLIC-GABAAR alpha-1 chimera | THDOC * | α1+α1- | | Laverty et al., 2017 |
| L | 6D6T * | GABA-A | Cholesterol * | β2- | Site 10 | Zhu et al., 2018 |
| L | 6D6T, 6D6U * | GABA-A | Cholesterol * | α1+γ2- | - | Zhu et al., 2018 |
| L | 6I53 * | GABA-A | PIP2 * | α1+ | overlap with site 10 | Laverty et al., 2019 |
| | 6HUO, 6HUK, 6HUJ, 6HUG, 6HUP | | | | | Masiulis et al., 2019 |
| 11 | 4TWD, 4TWF | ELIC | memantine, bromomemantine | | - | Ulens et al., 2014 |

**Supplementary Table S1: Overview of binding sites of $GABA_A$ receptors, including homologous proteins as displayed in Figure 2**. Binding sites were compared to Puthenkalam et al. and assigned to the new binding site nomenclature (Bocquet *et al.*, 2009; Nury *et al.*, 2011; Pan *et al.*, 2012; Zimmermann *et al.*, 2012; Sauguet *et al.*, 2013; Spurny *et al.*, 2013; Miller *et al.*, 2014; Ulens *et al.*, 2014; Spurny *et al.*, 2015; Nys *et al.*, 2016; Huang *et al.*, 2017; Laverty *et al.*, 2017; Miller *et al.*, 2017; Chen *et al.*, 2018; Fourati *et al.*, 2018; Liu *et al.*, 2018; Phulera *et al.*, 2018; Zhu *et al.*, 2018; Hénault *et al.*, 2019; Masiulis *et al.*, 2019; Kim *et al.*, 2020; Uchański *et al.*, 2021; Kasaragod *et al.*, 2022; Sente *et al.*, 2022; Zhu *et al.*, 2022). Numbers correspond to Figure 2, “L” stands for “lipid or cholesterol derived ligand”.

A

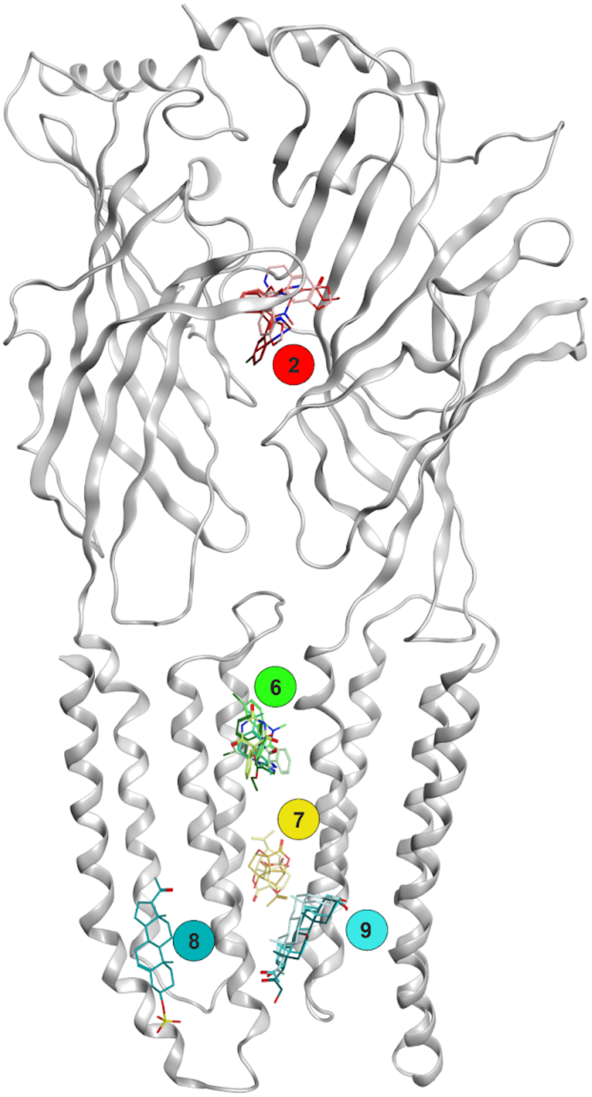


B

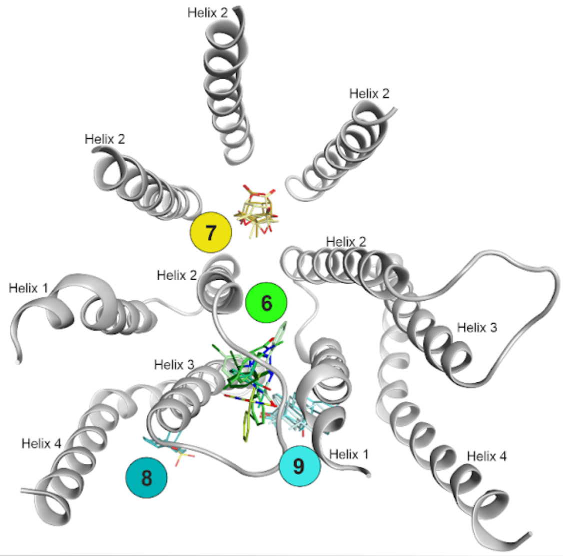


C

| | Ligand |
|---|---|
| 2 | GABA, Diazepam, Alprazolam, Flumazenil, Bicuculline methochloride, histamine, α-Cobratoxin |
| 6 | Diazepam, Propofol, Etomidate, Phenobarbital |
| 7 | Picrotoxin |
| 8 | Pregnenolone Sulfate, PIP2, lipid |
| 9 | Alphaxalone, Pregnenolone, THDOC |

**Supplementary Figure S1: Binding sites found in GABA$_A$ receptor atomic resolution structures. (a)** Side view and (**b**) top view of a superposition of the PDB files shown in (**c**). The ligands are represented in stick representation (GABA in red, diazepam in red for the ECD binding site (2), bicuculline methochloride in pink, flumazenil in red, diazepam in the TMD binding site (6) in light green, etomidate in green, phenobarbital in yellow-green, propofol in dark green, picrotoxin in yellow, alphaxalone in light cyan, THDOC in cyan, pregnanolone in dark cyan, pregnenolone sulfate in cyan). Binding sites are coloured and numbered according to their localization in the protein. Sites 2,6 and 9 are interface-located, whereas sites 8-9 are intrasubunit-located. Site 7 is located within the channel pore. (**c**) Table detailing all binding sites and their respective ligands. The corresponding binding site identification numbers reported in Puthenkalam *et. al.* are also depicted (Puthenkalam *et al.*, 2016).

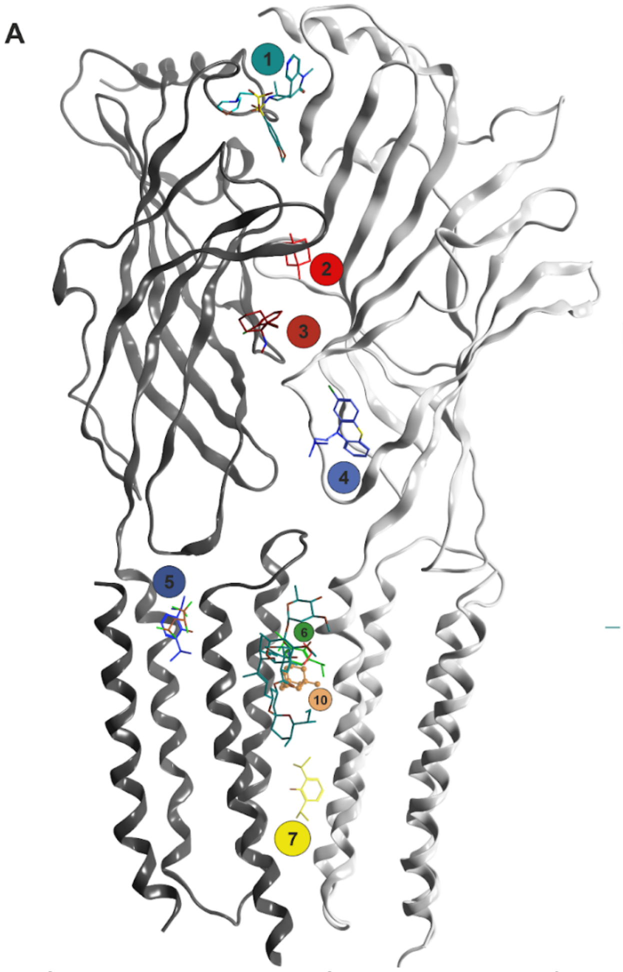


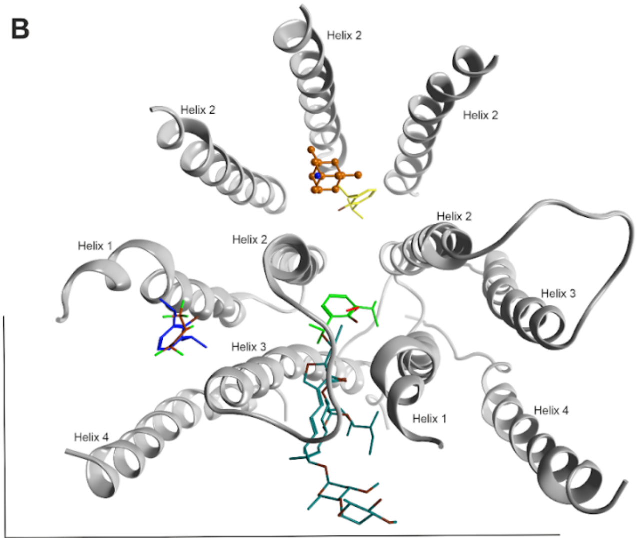


C

| | Ligand |
|---|---|
| 1 | AM-3607, 2-Ethanesulfonic acid |
| 2 | Memantine, Bromomemantine |
| 3 | Ketamine |
| 4 | Chlorpromazine, Bromopromazine |
| 5 | Propofol, Desflurane |
| 6 | Avermectin, Ethanol, Propofol |
| 7 | Propofol |
| 10 | Memantine, Bromomemantine |

**Supplementary Figure S2. Binding sites found in homologous $GABA_A$ receptor atomic resolution structures. (a)** Side view and (**b**) top view of a superposition of the protein data bank identities (PDB IDs) shown in (**c**). Two subunits of 6HUP are shown in grey ribbon representation. The ligands are represented in stick representation (AM-3607 in cyan, memantine in the ECD site (2) in red, ketamine in dark red, chlorpromazine in blue, propofol in dark blue, avermectin in green, propofol in the channel blocker site (7), memantine in the channel blocker site (10) in orange). Binding sites are coloured and numbered according to their localization in the protein. Sites 1-3 and 6 are interface-located, whereas sites 4, 5 are intrasubunit-located. Site 7 and 10 is located within the channel pore. (**c**) Table detailing all binding sites and their respective ligands and PDB IDs. The corresponding binding site identification numbers reported in Puthenkalam *et. al.* are also depicted (Puthenkalam *et al.*, 2016). ECD: extracellular domain, TMD: transmembrane domain.

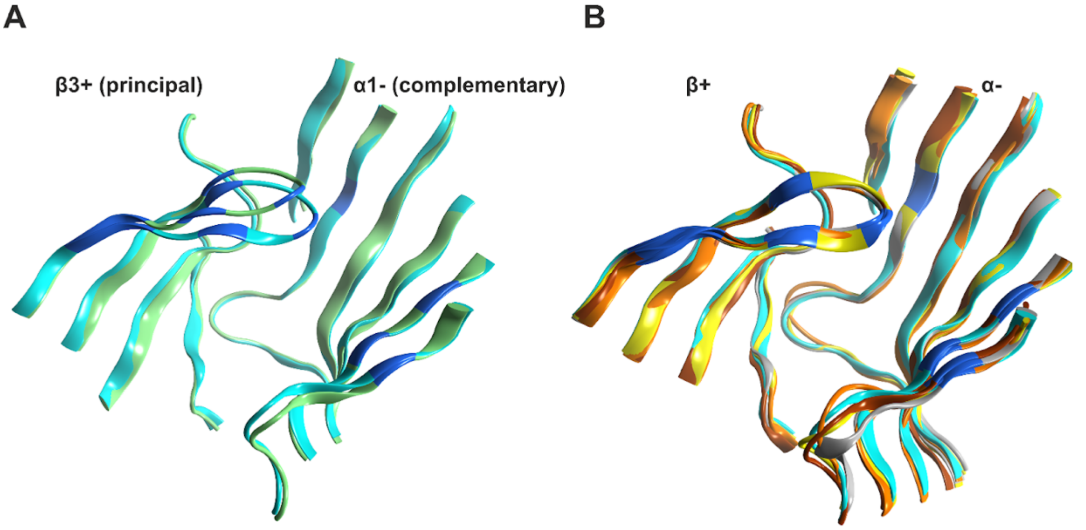


**Supplementary Figure S3:** GABA binding site (A) Comparison of the bicuculline bound state (6HUK in green) and GABA/picrotoxin bound state (6HUJ in cyan). Highlighted blue residues are showing the different residues in the b1, b2 and b3 subunits as in Figure 3A and 3D. (B) Superposition of b1 (6DW0 in orange), b2 (6D6T in yellow) and b3 (6HUJ in cyan, 7QNA in grey, 6A96 in brown) principal site and a1 (6HUJ, 6DW0, 6D6T), a4 (7QNA) and a5 (6A96) complementary site.

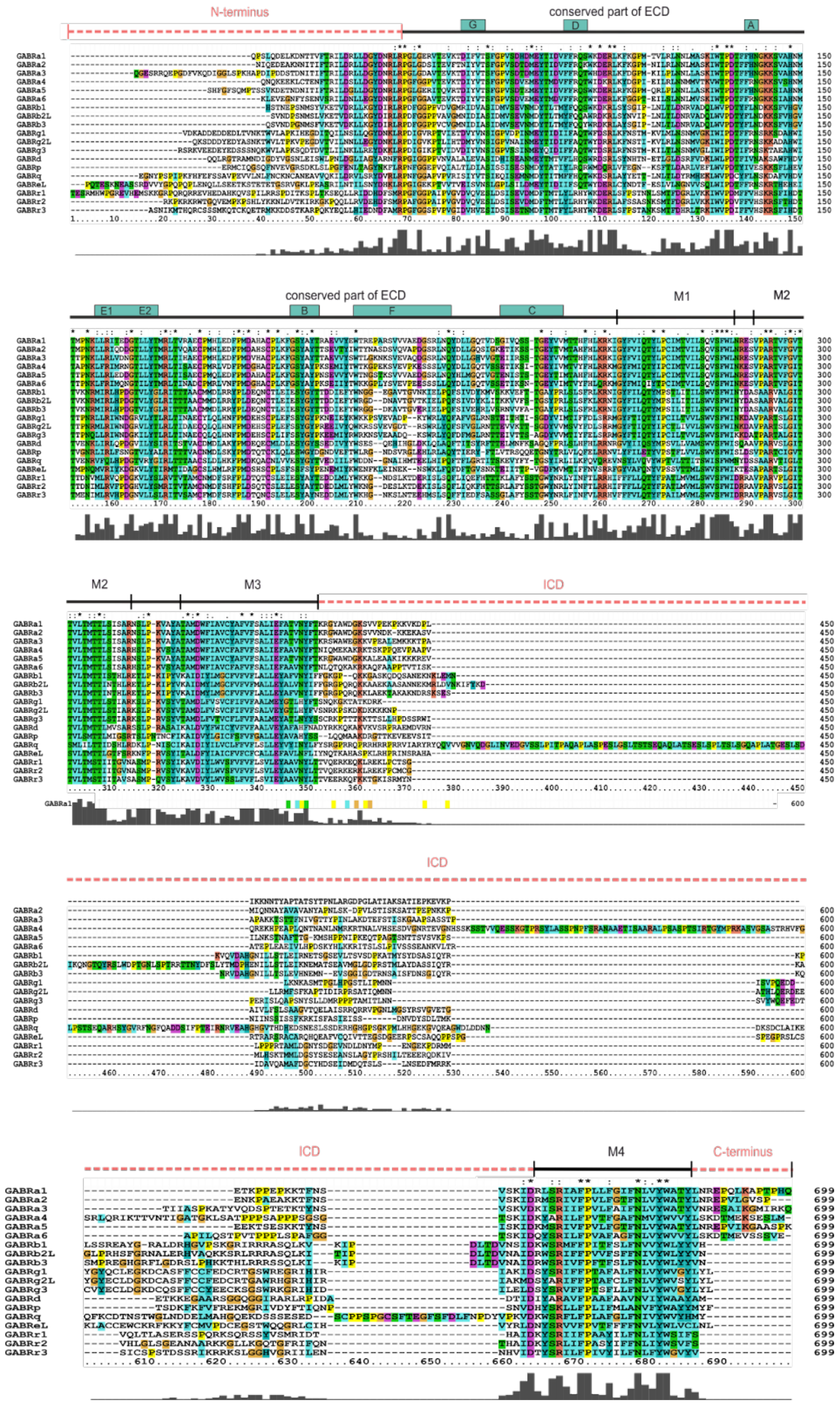


**Supplementary Figure S4:** Sequence alignment of the 19 human subunits with annotated segments A-G, M1-M4.

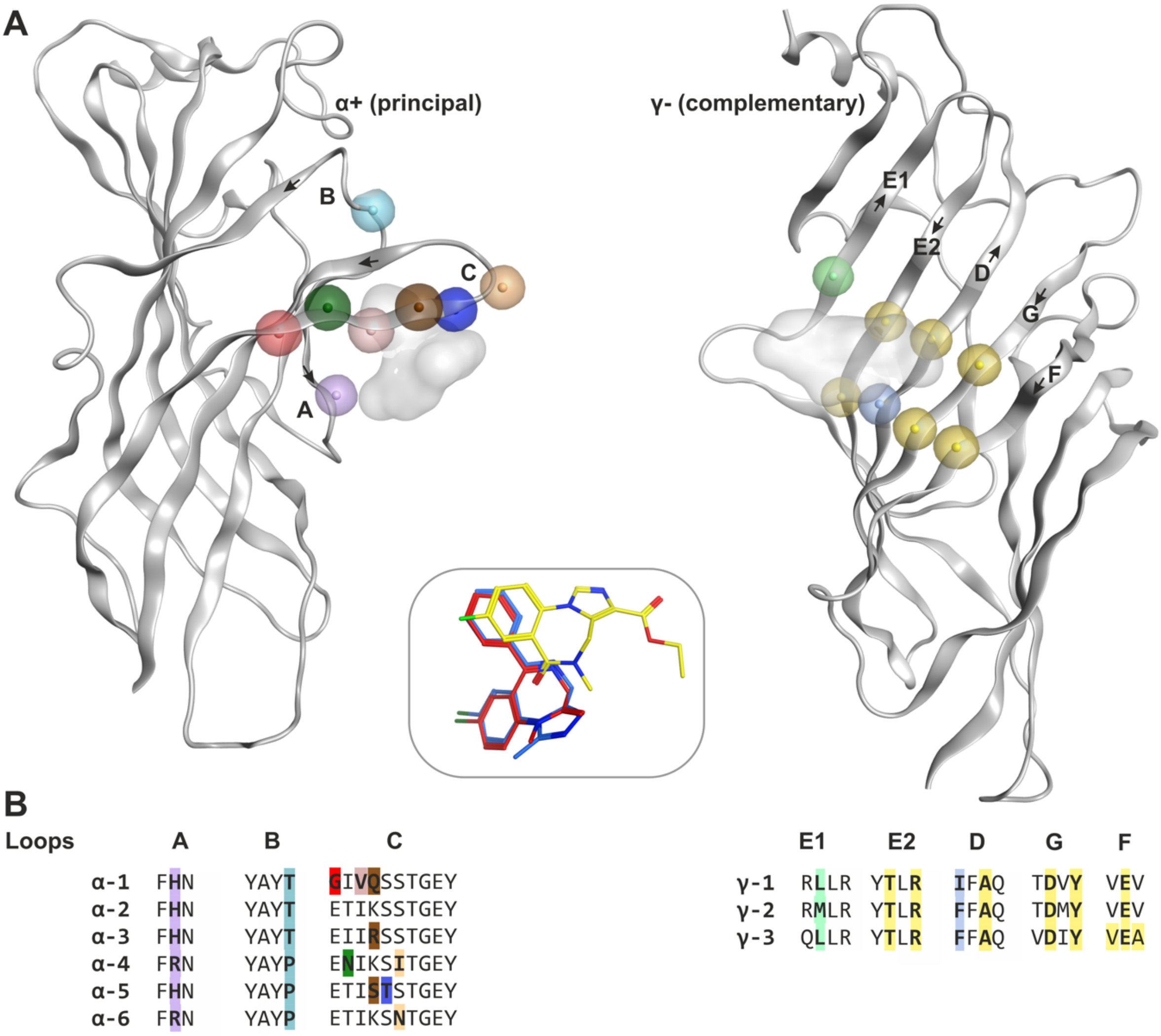


**Supplementary Figure S5:** ECD interface high affinity binding site for benzodiazepines: (A) Side view of the benzodiazepine binding site (α+/γ− interface) from a PDBeFold superposition of selected atomic resolution structures (PDB IDs: 6HUP – diazepam, 6HUO – alprazolam, 6D6T/ 6D6U – flumazenil). The subunits are displayed individually to view also the deeper parts of the pocket. The direction of the beta strands is indicated by arrows on the ribbon. Ligands are displayed as shadows on the protein, for visualization purposes diazepam and alprazolam on the principal (+) side and flumazenil on the complementary (-) side. The insert box in the middle depicts the binding modes of diazepam (red), alprazolam (blue) and flumazenil (yellow). The coloured spheres on the ribbon rendering identify the variable positions, color matched with panel (B), on the so-called "loops" (segments) A-G. In loop A, highlighted in purple, are the amino acid differences between α4,6 and α1,2,3,5 subunits that render the former diazepam-insensitive (DI) and the latter diazepam-sensitive (DS). In loop B, highlighted in light blue, is the amino acid difference between α4,5,6 and α1,2,3 subunits that render the former zolpidem-insensitive (ZI) and the latter zolpidem-sensitive (ZS). Several differences are observed in loop C: Amino acid positions which are unique for any isoform are highlighted in different colors: in red and pink the unique amino acids of the α1 subunit; in brown the amino acids which are unique for the α1, α3 and α5 subunits, respectively; in dark blue the unique amino acid of the α5 subunit and in light orange the amino acids unique to α4 and 6 subunits, respectively. On the complementary side of the pocket (γ−) the binding site contributing amino acids are highlighted: in yellow all the positions that are conserved in all three γ isoforms; in cyan the unique amino acid in the γ1 subunit and in light green the unique amino acid in the γ2 subunit. More variable positions are found on segment F (Supplementary Figure S4). (B) provides the partial alignment for the binding site forming segments, more loop F information is in S4.

| Scientific name of species | Species |
|---|---|
| Homo sapiens | Human |
| Rattus norvegicus | Rat |
| Mus musculus | Mouse |
| Pan paniscus | Bonobo, historically called : pygmy chimpanzee |
| Pan troglodytes | Common chimpanzee |
| Mustela putorius furo | Ferret |
| Macaca mulatta | Rhesus macaque |
| Canis lupus familiaris | Domestic dog |
| Danio rerio | Zebrafish |
| Bos taurus | Cattle |

**Supplementary Table S2:** Names of species that were used in this study for protein sequence comparisons.

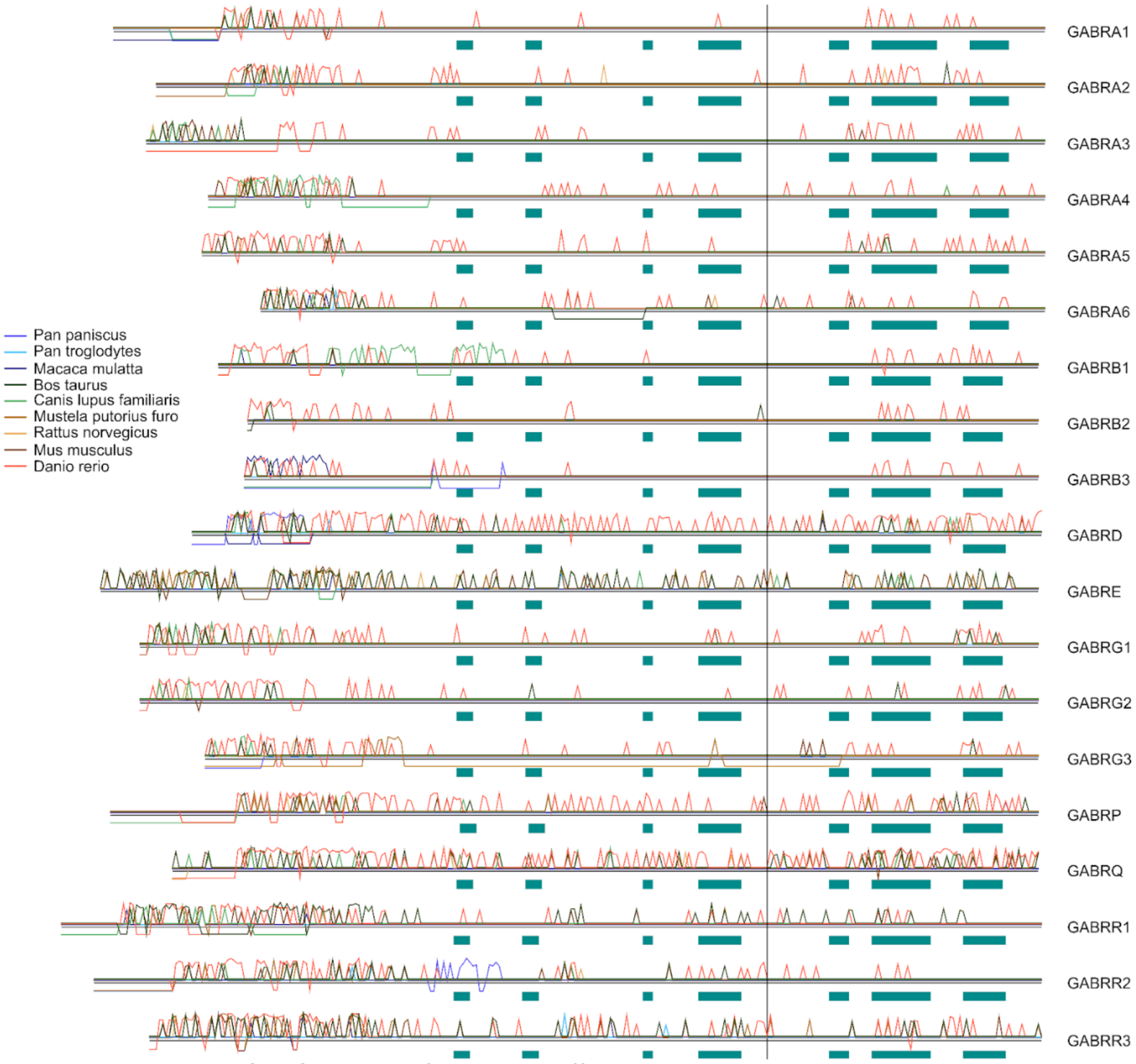


**Supplementary Figure S6: Overview of pairwise differences between the human subunits and nine orthologs in the ECD.** Substitution scores are plotted for pairwise alignments between human sequences and their orthologs as described in the methods. The colors reflect the different species as indicated in the legend. Full alignments and the correspondences for Danio rerio (which lacks an epsilon subunit) are in Supplementary Figure S8. The upwards spikes correlate with the (normalized) substitution score for amino acid exchanges from the blosum90 matrix, and downwards deflections indicate insertions/deletions (INDELs). The score graphs are aligned along the cys-loop, the second cysteine is represented by the vertical bar. The binding site forming segments G, D, A, E, B, F and C (from N- to C-terminal) are indicated by cyan bars below the graph for each subunit.

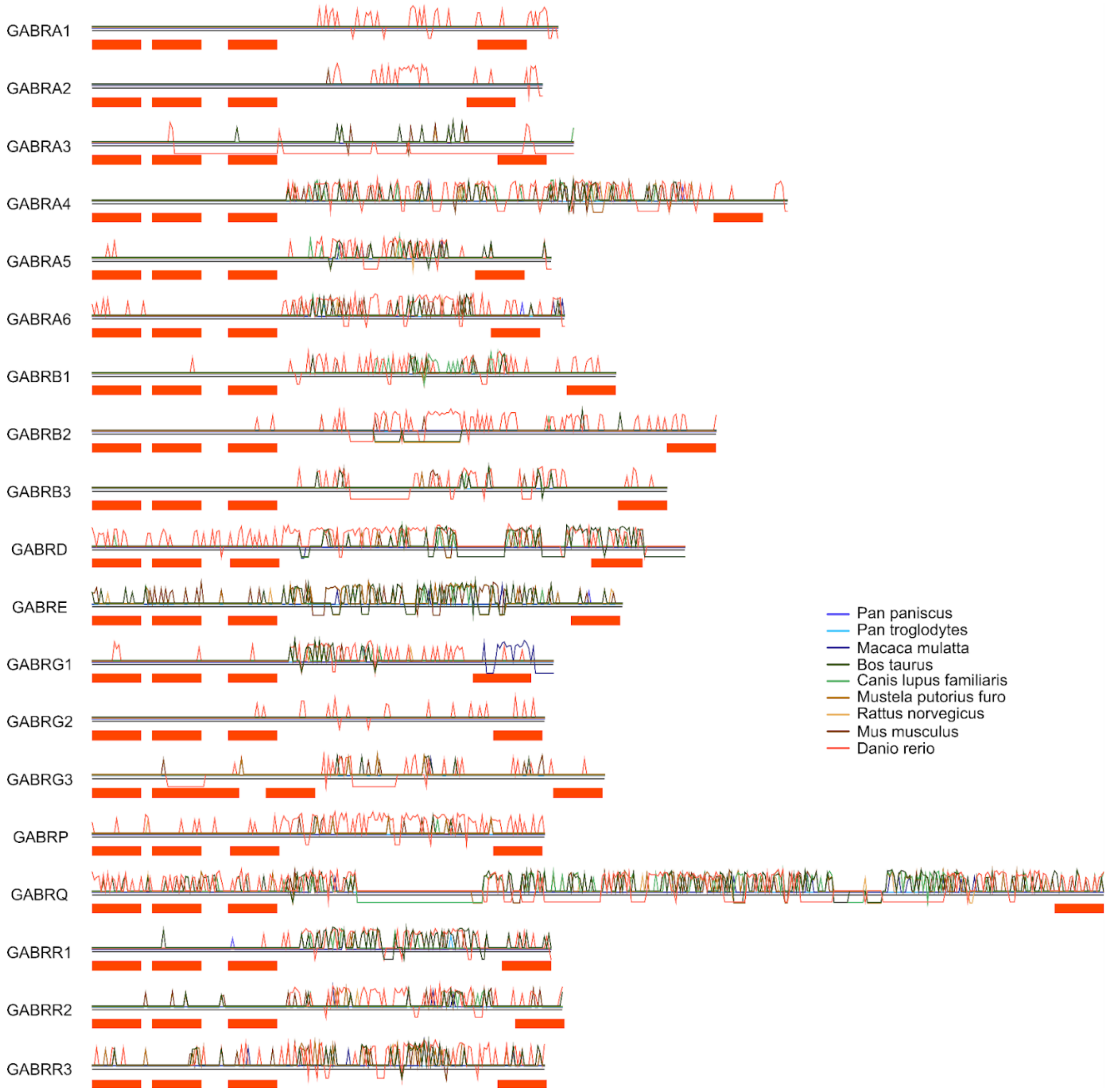


**Supplementary Figure S7: Overview of pairwise differences between the human subunits and nine orthologs in the TMD and ICD.** Substitution scores are plotted for pairwise alignments between human sequences and their orthologs as described in the methods. The colors reflect the different species as indicated in the legend. Full alignments and the correspondences for Danio rerio (which lacks an epsilon subunit) are in Supplementary Figure S8. The upwards spikes correlate with the (normalized) substitution score for amino acid exchanges from the blosum90 matrix, and downwards deflections indicate INDELs. The score graphs are aligned at the beginning of TM1, the four TM segments are indicated by red bars below each score graph.

**The next pages provide per subunit alignments from up to 10 species as Supplementary Figure S8**

```
                                                                   *  . * :  ** *  :* *  * *:* .* :  ** ************ ****************
P14867|GABRA1_Homo_sapiens                 ---------------------------------MRKSPGLSDCLWAWILLLST--LTGRSYGQPSLQDELKDNTTVFTRILDRLLDGYDNRLRPGLGERV   65
A0A2R9AU65|GABRA1_Pan_paniscus             ------------------MVQVSIASNELLQPAMRKSPGLSDCLWAWILLLST--LTGRSYGQPSLQDELKDNTTVFTRILDRLLDGYDNRLRPGLGERV   80
A0A2I3SDV7|GABRA1_Pan_troglodytes          ------------------MVQVSIASNELLQPAMRKSPGLSDCLWAWILLLST--LTGRSYGQPSLQDELKDNTTVFTRILDRLLDGYDNRLRPGLGERV   80
A0A1D5Q406|GABRA1_Macaca_mulatta           MFLNGEMEGKVENGWSRFPLLPMRCHIPLLQPAMRKSPGLSDYLWAWILLLST--LTGRSYGQPSLQDELKDNTTVFTRILDRLLDGYDNRLRPGLGERV   98
M3YP62|GABRA1_Mustela_putorius_furo        ---------------------------------MKKSPGFSEYLWAWTLLLST--LTGRSYGQPSLQDELKDNTTVFTRILDRLLDGYDNRLRPGLGERV   65
P62813|GABRA1_Rattus_norvegicus            ---------------------------------MKKSRGLSDYLWAWTLILST--LSGRSYGQPS-QDELKDNTTVFTRILDRLLDGYDNRLRPGLGERV   64
P62812|GABRA1_Mus_musculus                 ---------------------------------MKKSRGLSDYLWAWTLILST--LSGRSYGQPS-QDELKDNTTVFTRILDRLLDGYDNRLRPGLGERV   64
E2RSP8|GABRA1_Canis_lupus_familiaris       ------------------MVQVSIASNELHKPAMKKSLGLSDYLWAWTLLLST--LIGRSYGQPSLQDELKDNTTVFTRILDRLLDGYDNRLRPGLGERV   80
Q08BJ3|GABRA1_Danio_rerio                  --------------------------------MMWGGRG-AAWLWIWACLLVTNALAGKSSSQNA--DEQKDNTTVFTRILDSLLDGYDNRLRPGLGERV   65
P08219|GABRA1_Bos_taurus                   ---------------------------------MKKSPGLSDYLWAWTLFLST--LTGRSYGQPSLQDELKDNTTVFTRILDRLLDGYDNRLRPGLGERV   65
                                           1.......10........20........30........40........50........60........70........80........90.......100

                                           ***********:*****************************:**********************************************:*************
P14867|GABRA1_Homo_sapiens                 TEVKTDIFVTSFGPVSDHDMEYTIDVFFRQSWKDERLKFKGPMTVLRLNNLMASKIWTPDTFFHNGKKSVAHNMTMPNKLLRITEDGTLLYTMRLTVRAE  165
A0A2R9AU65|GABRA1_Pan_paniscus             TEVKTDIFVTSFGPVSDHDMEYTIDVFFRQSWKDERLKFKGPMTVLRLNNLMASKIWTPDTFFHNGKKSVAHNMTMPNKLLRITEDGTLLYTMRLTVRAE  180
A0A2I3SDV7|GABRA1_Pan_troglodytes          TEVKTDIFVTSFGPVSDHDMEYTIDVFFRQSWKDERLKFKGPMTVLRLNNLMASKIWTPDTFFHNGKKSVAHNMTMPNKLLRITEDGTLLYTMRLTVRAE  180
A0A1D5Q406|GABRA1_Macaca_mulatta           TEVKTDIFVTSFGPVSDHDMEYTIDVFFRQSWKDERLKFKGPMTVLRLNNLMASKIWTPDTFFHNGKKSVAHNMTMPNKLLRITEDGTLLYTMRLTVRAE  198
M3YP62|GABRA1_Mustela_putorius_furo        TEVKTDIFVTSFGPVSDHDMEYTIDVFFRQSWKDERLKFKGPMTVLRLNNLMASKIWTPDTFFHNGKKSVAHNMTMPNKLLRITEDGTLLYTMRLTVRAE  165
P62813|GABRA1_Rattus_norvegicus            TEVKTDIFVTSFGPVSDHDMEYTIDVFFRQSWKDERLKFKGPMTVLRLNNLMASKIWTPDTFFHNGKKSVAHNMTMPNKLLRITEDGTLLYTMRLTVRAE  164
P62812|GABRA1_Mus_musculus                 TEVKTDIFVTSFGPVSDHDMEYTIDVFFRQSWKDERLKFKGPMTVLRLNNLMASKIWTPDTFFHNGKKSVAHNMTMPNKLLRITEDGTLLYTMRLTVRAE  164
E2RSP8|GABRA1_Canis_lupus_familiaris       TEVKTDIFVTSFGPVSDHDMEYTIDVFFRQSWKDERLKFKGPMTVLRLNNLMASKIWTPDTFFHNGKKSVAHNMTMPNKLLRITEDGTLLYTMRLTVRAE  180
Q08BJ3|GABRA1_Danio_rerio                  TEVKTDIFVTSIGPVSDHDMEYTIDVFFRQSWKDERLKFKGPMAVLRLNNLMASKIWTPDTFFHNGKKSVAHNMTMPNKLLRITEEGTLLYTMRLTVRAE  165
P08219|GABRA1_Bos_taurus                   TEVKTDIFVTSFGPVSDHDMEYTIDVFFRQSWKDERLKFKGPMTVLRLNNLMASKIWTPDTFFHNGKKSVAHNMTMPNKLLRITEDGTLLYTMRLTVRAE  165
                                           .......110.......120.......130.......140.......150.......160.......170.......180.......190.......200

                                           ****************************** ***  .*:*****:*********:**:****:*****************************************
P14867|GABRA1_Homo_sapiens                 CPMHLEDFPMDAHACPLKFGSYAYTRAEVVYEWTREPARSVVVAEDGSRLNQYDLLGQTVDSGIVQSSTGEYVVMTTHFHLKRKIGYFVIQTYLPCIMTV  265
A0A2R9AU65|GABRA1_Pan_paniscus             CPMHLEDFPMDAHACPLKFGSYAYTRAEVVYEWTREPARSVVVAEDGSRLNQYDLLGQTVDSGIVQSSTGEYVVMTTHFHLKRKIGYFVIQTYLPCIMTV  280
A0A2I3SDV7|GABRA1_Pan_troglodytes          CPMHLEDFPMDAHACPLKFGSYAYTRAEVVYEWTREPARSVVVAEDGSRLNQYDLLGQTVDSGIVQSSTGEYVVMTTHFHLKRKIGYFVIQTYLPCIMTV  280
A0A1D5Q406|GABRA1_Macaca_mulatta           CPMHLEDFPMDAHACPLKFGSYAYTRAEVVYEWTREPARSVVVAEDGSRLNQYDLLGQTVDSGIVQSSTGEYVVMTTHFHLKRKIGYFVIQTYLPCIMTV  298
M3YP62|GABRA1_Mustela_putorius_furo        CPMHLEDFPMDAHACPLKFGSYAYTRAEVVYEWTREPARSVVVAEDGSRLNQYDLLGQTVDSGIVQSSTGEYVVMTTHFHLKRKIGYFVIQTYLPCIMTV  265
P62813|GABRA1_Rattus_norvegicus            CPMHLEDFPMDAHACPLKFGSYAYTRAEVVYEWTREPARSVVVAEDGSRLNQYDLLGQTVDSGIVQSSTGEYVVMTTHFHLKRKIGYFVIQTYLPCIMTV  264
P62812|GABRA1_Mus_musculus                 CPMHLEDFPMDAHACPLKFGSYAYTRAEVVYEWTREPARSVVVAEDGSRLNQYDLLGQTVDSGIVQSSTGEYVVMTTHFHLKRKIGYFVIQTYLPCIMTV  264
E2RSP8|GABRA1_Canis_lupus_familiaris       CPMHLEDFPMDAHACPLKFGSYAYTRAEVVYEWTREPARSVVVAEDGSRLNQYDLLGQTVDSGIVQSSTGEYVVMTTHFHLKRKIGYFVIQTYLPCIMTV  280
Q08BJ3|GABRA1_Danio_rerio                  CPMHLEDFPMDAHACPLKFGSYAYTRAEVVYVWTRGAAQSVVVADDGSRLNQYDLMGQSVDSGVVQSSTGEYVVMTTHFHLKRKIGYFVIQTYLPCIMTV  265
P08219|GABRA1_Bos_taurus                   CPMHLEDFPMDAHACPLKFGSYAYTRAEVVYEWTREPARSVVVAEDGSRLNQYDLLGQTVDSGIVQSSTGEYVVMTTHFHLKRKIGYFVIQTYLPCIMTV  265
                                           .......210.......220.......230.......240.......250.......260.......270.......280.......290.......300

                                           ************************************************************************************ ** *:.*:***
P14867|GABRA1_Homo_sapiens                 ILSQVSFWLNRESVPARTVFGVTTVLTMTTLSISARNSLPKVAYATAMDWFIAVCYAFVFSALIEFATVNYFTKRGYAWDGKSVVPEKPKKVKDPLIKKN  365
A0A2R9AU65|GABRA1_Pan_paniscus             ILSQVSFWLNRESVPARTVFGVTTVLTMTTLSISARNSLPKVAYATAMDWFIAVCYAFVFSALIEFATVNYFTKRGYAWDGKSVVPEKPKKVKDPLIKKN  380
A0A2I3SDV7|GABRA1_Pan_troglodytes          ILSQVSFWLNRESVPARTVFGVTTVLTMTTLSISARNSLPKVAYATAMDWFIAVCYAFVFSALIEFATVNYFTKRGYAWDGKSVVPEKPKKVKDPLIKKN  380
A0A1D5Q406|GABRA1_Macaca_mulatta           ILSQVSFWLNRESVPARTVFGVTTVLTMTTLSISARNSLPKVAYATAMDWFIAVCYAFVFSALIEFATVNYFTKRGYAWDGKSVVPEKPKKVKDPLIKKN  398
M3YP62|GABRA1_Mustela_putorius_furo        ILSQVSFWLNRESVPARTVFGVTTVLTMTTLSISARNSLPKVAYATAMDWFIAVCYAFVFSALIEFATVNYFTKRGYAWDGKSVVPEKPKKVKDPLIKKN  365
P62813|GABRA1_Rattus_norvegicus            ILSQVSFWLNRESVPARTVFGVTTVLTMTTLSISARNSLPKVAYATAMDWFIAVCYAFVFSALIEFATVNYFTKRGYAWDGKSVVPEKPKKVKDPLIKKN  364
P62812|GABRA1_Mus_musculus                 ILSQVSFWLNRESVPARTVFGVTTVLTMTTLSISARNSLPKVAYATAMDWFIAVCYAFVFSALIEFATVNYFTKRGYAWDGKSVVPEKPKKVKDPLIKKN  364
E2RSP8|GABRA1_Canis_lupus_familiaris       ILSQVSFWLNRESVPARTVFGVTTVLTMTTLSISARNSLPKVAYATAMDWFIAVCYAFVFSALIEFATVNYFTKRGYAWDGKSVVPEKPKKVKDPLIKKN  380
Q08BJ3|GABRA1_Danio_rerio                  ILSQVSFWLNRESVPARTVFGVTTVLTMTTLSISARNSLPKVAYATAMDWFIAVCYAFVFSALIEFATVNYFTKRGYAWDGKSVVPEKQKKKKESLLKKN  365
P08219|GABRA1_Bos_taurus                   ILSQVSFWLNRESVPARTVFGVTTVLTMTTLSISARNSLPKVAYATAMDWFIAVCYAFVFSALIEFATVNYFTKRGYAWDGKSVVPEKPKKVKDPLIKKN  365
                                           .......310.......320.......330.......340.......350.......360.......370.......380.......390.......400

                                           *** *.***:::**:** ***********.  *.* *  *:.****.************::********* ***********::*:*:. .  **
P14867|GABRA1_Homo_sapiens                 NTY-APTATSYTPNLARGDPGLATIAKSATIEPKEVK--PETKPPEPKKTFNSVSKIDRLSRIAFPLLFGIFNLVYWATYLNREPQLKAPT--PHQ  456
A0A2R9AU65|GABRA1_Pan_paniscus             NTY-APTATSYTPNLARGDPGLATIAKSATIEPKEVK--PETKPPEPKKTFNSVSKIDRLSRIAFPLLFGIFNLVYWATYLNREPQLKAPT--PHQ  471
A0A2I3SDV7|GABRA1_Pan_troglodytes          NTY-APTATSYTPNLARGDPGLATIAKSATIEPKEVK--PETKPPEPKKTFNSVSKIDRLSRIAFPLLFGIFNLVYWATYLNREPQLKAPT--PHQ  471
A0A1D5Q406|GABRA1_Macaca_mulatta           NTY-APTATSYTPNLARGDPGLATIAKSATIEPKEVK--PETKPPEPKKTFNSVSKIDRLSRIAFPLLFGIFNLVYWATYLNREPQLKAPT--PHQ  489
M3YP62|GABRA1_Mustela_putorius_furo        NTY-APTATSYTPNLARGDPGLATIAKSATIEPKEVK--PETKPPEPKKTFNSVSKIDRLSRIAFPLLFGIFNLVYWATYLNREPQLKAPT--PHQ  456
P62813|GABRA1_Rattus_norvegicus            NTY-APTATSYTPNLARGDPGLATIAKSATIEPKEVK--PETKPPEPKKTFNSVSKIDRLSRIAFPLLFGIFNLVYWATYLNREPQLKAPT--PHQ  455
P62812|GABRA1_Mus_musculus                 NTY-APTATSYTPNLARGDPGLATIAKSATIEPKEVK--PETKPPEPKKTFNSVSKIDRLSRIAFPLLFGIFNLVYWATYLNREPQLKAPT--PHQ  455
E2RSP8|GABRA1_Canis_lupus_familiaris       NTY-APTATSYTPNLARGDPGLATIAKSATIEPKEVK--PETKPPEPKKTFNSVSKIDRLSRIAFPLLFGIFNLVYWATYLNREPQLKAPT--PHQ  471
Q08BJ3|GABRA1_Danio_rerio                  NTYTAATATAFAPNIAR-DPGLATIAKSAPPPPTEPKEEPKPKPPEAKKTFNSVSKIDRIARIAFPLLFGTFNLVYWATYLNKKPKLQGMNLQPH-  459
P08219|GABRA1_Bos_taurus                   NTY-APTATSYTPNLARGDPGLATIAKSATIEPKEVK--PETKPPEPKKTFNSVSKIDRLSRIAFPLLFGIFNLVYWATYLNREPQLKAPT--PHQ  456
                                           .......410.......420.......430.......440.......450.......460.......470.......480.......490......
```

```
                                                                        .   *::.: *  .        .*  *****:********************** :*** *:*******
P47869|GABRA2_Homo_sapiens               ----------------------MKTKLNIYNMQFLLFVFLVWDPARLVLANIQEDEAKNNITIFTRILDRLLDGYDNRLRPGLGDSITEVFTNIYVTSFG  78
XP_003816076.1|GABRA2_Pan_paniscus       ----------------------MKTKLNIYNMQFLLFVFLVWDPARLVLANIQEDEAKNNITIFTRILDRLLDGYDNRLRPGLGDSITEVFTNIYVTSFG  78
M3Y0V9|GABRA2_Mustela_putorius_furo      MVQLNVILSVFYFSCMALQRAAMKTKLNTYNVQLLLVFLVWDPARLVLANIQEDEAKNNITIFTRILDRLLDGYDNRLRPGLGDSITEVFTNIYVTSFG  100
P23576|GABRA2_Rattus_norvegicus          ----------------------MRTKLSTCNVWFPLLVLLVWNPARLVLANIQEDEAKNNITIFTRILDRLLDGYDNRLRPGLGDSITEVFTNIYVTSFG  78
P26048|GABRA2_Mus_musculus               ----------------------MKTKLSTCNVWSLLVLLVWDPVRLVLANIQEDEAKNNITIFTRILDRLLDGYDNRLRPGLGDSITEVFTNIYVTSFG  78
A0A5F4CYJ7|GABRA2_Canis_lupus_familiaris -------------------------------MRLLLLVVLLWDPARLVLANIQEDEAKNNITIFTRILDRLLDGYDNRLRPGLGDSITEVFTNIYVTSFG  69
H2QPE5|GABRA2_Pan_troglodytes            ----------------------MKTKLNIYNMQFLLFVFLVWDPARLVLANIQEDEAKNNITIFTRILDRLLDGYDNRLRPGLGDSITEVFTNIYVTSFG  78
E7F635|GABRA2a_Danio_rerio               ---------------------MILRRGHSHGFLIHLFLLVLW-RACSEAEVSGSDAFKNNITLFTRILDRLLDGYDNRLRPGLGDRVTEVKTDIYVTSFG  78
XP_028703913.1|GABRA2_Macaca_mulatta     ----------------------MKTKLNIYNMQLLLFVFLVWDPARLVLANIQEDEAKNNITIFTRILDRLLDGYDNRLRPGLGDSITEVFTNIYVTSFG  78
P10063|GABRA2_Bos_taurus                 ----------------------MKTKLNSSNMQLLLFVFLAWDPARLVLANIQEDEAKNNITIFTRILDRLLDGYDNRLRPGLGDSITEVFTNIYVTSFG  78
                                         1.......10........20........30........40........50........60........70........80........90.......100

                                         **********:******:*.******:********** ******************************** ::************************* *
P47869|GABRA2_Homo_sapiens               PVSDTDMEYTIDVFFRQKWKDERLKFKGPMNILRLNNLMASKIWTPDTFFHNGKKSVAHNMTMPNKLLRIQDDGTLLYTMRLTVQAECPMHLEDFPMDAH  178
XP_003816076.1|GABRA2_Pan_paniscus       PVSDTDMEYTIDVFFRQKWKDERLKFKGPMNILRLNNLMASKIWTPDTFFHNGKKSVAHNMTMPNKLLRIQDDGTLLYTMRLTVQAECPMHLEDFPMDAH  178
M3Y0V9|GABRA2_Mustela_putorius_furo      PVSDTDMEYTIDVFFRQKWKDERLKFKGPMNILRLNNLMASKIWTPDTFFHNGKKSVAHNMTMPNKLLRIQDDGTLLYTMRLTVQAECPMHLEDFPMDAH  200
P23576|GABRA2_Rattus_norvegicus          PVSDTDMEYTIDVFFRQKWKDERLKFKGPMNILRLNNSMASKIWTPDTFFHNGKKSVAHNMTMPNKLLRIQDDGTLLYTMRLTVQAECPMHLEDFPMDAH  178
P26048|GABRA2_Mus_musculus               PVSDTDMEYTIDVFFRQKWKDERLKFKGPMNILRLNNLMASKIWTPDTFFHNGKKSVAHNMTMPNKLLRIQDDGTLLYTMRLTVQAECPMHLEDFPMDAH  178
A0A5F4CYJ7|GABRA2_Canis_lupus_familiaris PVSDTDMEYTIDVFFRQKWKDERLKFKGPMNILRLNNLMASKIWTPDTFFHNGKKSVAHNMTMPNKLLRIQDDGTLLYTMRLTVQAECPMHLEDFPMDAH  169
H2QPE5|GABRA2_Pan_troglodytes            PVSDTDMEYTIDVFFRQKWKDERLKFKGPMNILRLNNLMASKIWTPDTFFHNGKKSVAHNMTMPNKLLRIQDDGTLLYTMRLTVQAECPMHLEDFPMDAH  178
E7F635|GABRA2a_Danio_rerio               PVSDTDMEYTVDVFFRQRWTDERLKFHGPMNILRLNNLMASKIWTPDTFFHNGKKSVAHNMTMPNKLLRIMENGTLLYTMRLTVQAECPMHLEDFPMDFH  178
XP_028703913.1|GABRA2_Macaca_mulatta     PVSDTDMEYTIDVFFRQKWKDERLKFKGPMNILRLNNLMASKIWTPDTFFHNGKKSVAHNMTMPNKLLRIQDDGTLLYTMRLTVQAECPMHLEDFPMDAH  178
P10063|GABRA2_Bos_taurus                 PVSDTDMEYTIDVFFRQKWKDERLKFKGPMNILRLNNLMASKIWTPDTFFHNGKKSVAHNMTMPNKLLRIQDDGTLLYTMRLTVQAECPMHLEDFPMDAH  178
                                         .......110.......120.......130.......140.......150.......160.......170.......180.......190.......200

                                         ************ :****:** *.: **:************* **::*****************************************************
P47869|GABRA2_Homo_sapiens               SCPLKFGSYAYTTSEVTYIWTYNASDSVQVAPDGSRLNQYDLLGQSIGKETIKSSTGEYTVMTAHFHLKRKIGYFVIQTYLPCIMTVILSQVSFWLNRES  278
XP_003816076.1|GABRA2_Pan_paniscus       SCPLKFGSYAYTTSEVTYIWTYNASDSVQVAPDGSRLNQYDLLGQSIGKETIKSSTGEYTVMTAHFHLKRKIGYFVIQTYLPCIMTVILSQVSFWLNRES  278
M3Y0V9|GABRA2_Mustela_putorius_furo      SCPLKFGSYAYTTSEVTYIWTYNASDSVQVAPDGSRLNQYDLLGQSIGKETIKSSTGEYTVMTAHFHLKRKIGYFVIQTYLPCIMTVILSQVSFWLNRES  300
P23576|GABRA2_Rattus_norvegicus          SCPLKFGSYAYTTSEVTYIWTYNPSDSVQVAPDGSRLNQYDLLGQSIGKETIKSSTGEYTVMTAHFHLKRKIGYFVIQTYLPCIMTVILSQVSFWLNRES  278
P26048|GABRA2_Mus_musculus               SCPLKFGSYAYTTSEVTYIWTYNASDSVQVAPDGSRLNQYDLLGQSIGKETIKSSTGEYTVMTAHFHLKRKIGYFVIQTYLPCIMTVILSQVSFWLNRES  278
A0A5F4CYJ7|GABRA2_Canis_lupus_familiaris SCPLKFGSYAYTTSEVTYIWTYNASDSVQVAPDGSRLNQYDLLGQSIGKETIKSSTGEYTVMTAHFHLKRKIGYFVIQTYLPCIMTVILSQVSFWLNRES  269
H2QPE5|GABRA2_Pan_troglodytes            SCPLKFGSYAYTTSEVTYIWTYNASDSVQVAPDGSRLNQYDLLGQSIGKETIKSSTGEYTVMTAHFHLKRKIGYFVIQTYLPCIMTVILSQVSFWLNRES  278
E7F635|GABRA2a_Danio_rerio               SCPLKFGSYAYTLTEVTYVWTRNATLSVEVAPDGSRLNQYDLMGQTVGKETIKSSTGEYTVMTAHFHLKRKIGYFVIQTYLPCIMTVILSQVSFWLNRES  278
XP_028703913.1|GABRA2_Macaca_mulatta     SCPLKFGSYAYTTSEVTYIWTYNASDSVQVAPDGSRLNQYDLLGQSIGKETIKSSTGEYTVMTAHFHLKRKIGYFVIQTYLPCIMTVILSQVSFWLNRES  278
P10063|GABRA2_Bos_taurus                 SCPLKFGSYAYTTSEVTYIWTYNASDSVQVAPDGSRLNQYDLPGQSIGKETIKSSTGEYTVMTAHFHLKRKIGYFVIQTYLPCIMTVILSQVSFWLNRES  278
                                         .......210.......220.......230.......240.......250.......260.......270.......280.......290.......300

                                         **********************************************************************:*******  *** :*************::
P47869|GABRA2_Homo_sapiens               VPARTVFGVTTVLTMTTLSISARNSLPKVAYATAMDWFIAVCYAFVFSALIEFATVNYFTKRGWAWDGKSVVNDKKKEKASVMIQNNAYAVAVANYAPNL  378
XP_003816076.1|GABRA2_Pan_paniscus       VPARTVFGVTTVLTMTTLSISARNSLPKVAYATAMDWFIAVCYAFVFSALIEFATVNYFTKRGWAWDGKSVVNDKKKEKASVMIQNNAYAVAVANYAPNL  378
M3Y0V9|GABRA2_Mustela_putorius_furo      VPARTVFGVTTVLTMTTLSISARNSLPKVAYATAMDWFIAVCYAFVFSALIEFATVNYFTKRGWAWDGKSVVNDKKKEKASVMIQNNAYAVAVANYAPNL  400
P23576|GABRA2_Rattus_norvegicus          VPARTVFGVTTVLTMTTLSISARNSLPKVAYATAMDWFIAVCYAFVFSALIEFATVNYFTKRGWAWDGKSVVNDKKKEKGSVMIQNNAYAVAVANYAPNL  378
P26048|GABRA2_Mus_musculus               VPARTVFGVTTVLTMTTLSISARNSLPKVAYATAMDWFIAVCYAFVFSALIEFATVNYFTKRGWAWDGKSVVNDKKKEKGSVMIQNNAYAVAVANYAPNL  378
A0A5F4CYJ7|GABRA2_Canis_lupus_familiaris VPARTVFGVTTVLTMTTLSISARNSLPKVAYATAMDWFIAVCYAFVFSALIEFATVNYFTKRGWAWDGKSVVNDKKKEKASVMIQNNAYAVAVANYAPNL  369
H2QPE5|GABRA2_Pan_troglodytes            VPARTVFGVTTVLTMTTLSISARNSLPKVAYATAMDWFIAVCYAFVFSALIEFATVNYFTKRGWAWDGKSVVNDKKKEKASVMIQNNAYAVAVANYAPNL  378
E7F635|GABRA2a_Danio_rerio               VPARTVFGVTTVLTMTTLSISARNSLPKVAYATAMDWFIAVCYAFVFSALIEFATVNYFTKRGWAWDGKSIVNDKKKE-TSVMKKNNAYAVAVANYAPHI  377
XP_028703913.1|GABRA2_Macaca_mulatta     VPARTVFGVTTVLTMTTLSISARNSLPKVAYATAMDWFIAVCYAFVFSALIEFATVNYFTKRGWAWDGKSVVNDKKKEKASVMIQNNAYAVAVANYAPNL  378
P10063|GABRA2_Bos_taurus                 VPARTVFGVTTVLTMTTLSISARNSLPKVAYATAMDWFIAVCYAFVFSALIEFATVNYFTKRGWAWDGKSVVNDKKKEKASVMIQNNAYAVAVANYAPNL  378
                                         .......310.......320.......330.......340.......350.......360.......370.......380.......390.......400

                                         :**..*.*:**** ****. *.:  . *:****************:******:***************: .: *
P47869|GABRA2_Homo_sapiens               SKDPVLSTISKSATTPEPNKKPENKPAEAKKTFNSVSKIDRMSRIVFPVLFGTFNLVYWATYLNREPVL-GVSP--  451
XP_003816076.1|GABRA2_Pan_paniscus       SKDPVLSTISKSATTPEPNKKPENKPAEAKKTFNSVSKIDRMSRIVFPVLFGTFNLVYWATYLNREPVL-GVSP--  451
M3Y0V9|GABRA2_Mustela_putorius_furo      SKDPVLSTISKSATTPEPNKKPENKPAEAKKTFNSVSKIDRMSRIVFPVLFGTFNLVYWATYLNREPVL-GVSP--  473
P23576|GABRA2_Rattus_norvegicus          SKDPVLSTISKSATTPEPNKKPENKPAEAKKTFNSVSKIDRMSRIVFPVLFGTFNLVYWATYLNREPVL-GVSP--  451
P26048|GABRA2_Mus_musculus               SKDPVLSTISKSATTPEPNKKPENKPAEAKKTFNSVSKIDRMSRIVFPVLFGTFNLVYWATYLNREPVL-GVSP--  451
A0A5F4CYJ7|GABRA2_Canis_lupus_familiaris SKDPVLSTISKSATTPEPNKKPENKPAEAKKTFNSVSKIDRMSRIVFPVLFGTFNLVYWATYLNREPVL-GVSP--  442
H2QPE5|GABRA2_Pan_troglodytes            SKDPVLSTISKSATTPEPNKKPENKPAEAKKTFNSVSKIDRMSRIVFPVLFGTFNLVYWATYLNREPVL-GVSP--  451
E7F635|GABRA2a_Danio_rerio               AKDSALPTVSKSA-TPEPS-KAQPVAKETKKTFNSVSKIDRMSRIIFPVLFGSFNLVYWATYLNREPVIKNMVPSP  451
XP_028703913.1|GABRA2_Macaca_mulatta     SKDPVLSTISKSATTPEPNKKPENKPAEAKKTFNSVSKIDRMSRIVFPVLFGTFNLVYWATYLNREPVL-GVSP--  451
P10063|GABRA2_Bos_taurus                 SKDPVLSTISKSATTPEPNKKPENKPAEAKKTFNSVSKIDRMSRIVFPVLFGTFNLVYWATYLNREPVL-GVSP--  451
                                         .......410.......420.......430.......440.......450.......460.......470.....
```

```
                                                                              :: : *    *   *****.**************************::****:*:****
P34903|GABRA3_Homo_sapiens           MIITQTSHCYMTSLGILFLINILPGTTGQGESRRQEPGDFVKQDIGGLSPKHAPDIPDDSTDNITIFTRILDRLLDGYDNRLRPGLGDAVTEVKTDIYVT  100
A0A2R9AHX2|GABRA3_Pan_paniscus       MIITQTSHCYMTSLGILFLINILPGTTGQGESRRQEPGDFVKQDIGGLSPKHAPDIPDDSTDNITIFTRILDRLLDGYDNRLRPGLGDAVTEVKTDIYVT  100
H2QZ88|GABRA3_Pan_troglodytes        MIITQTSHCYMTSLGILFLINILPGTTGQGESRRQEPGDFVKQDIGGLSPKHAPDIPDDSTDNITIFTRILDRLLDGYDNRLRPGLGDAVTEVKTDIYVT  100
F7G8R5|GABRA3_Macaca_mulatta         MIITQMSHYYMTSLGILFLINILPGTTGQGESRRQEPGDFVKQDIGGLSPKHAPDIPDDSTDNITIFTRILDRLLDGYDNRLRPGLGDAVTEVKTDIYVT  100
M3YJX4|GABRA3_Mustela_putorius_furo  MIVTQMSHFYTTSLGFLFLINIFPGITGQGESRRQEPGDFVKQDIGGLSPKHAPDIPDDSTDNITIFTRILDRLLDGYDNRLRPGLGDAVTEVKTDIYVT  100
P20236|GABRA3_Rattus_norvegicus      MITTQMWHFYVTRVGLLLLISILPGTTGQGESRRQEPGDFVKQDIGGLSPKHAPDIPDDSTDNITIFTRILDRLLDGYDNRLRPGLGDAVTEVKTDIYVT  100
P26049|GABRA3_Mus_musculus           MIITQMWHFYVTRVVLLLLISILPGTTSQGESRRQEPGDFVKQDIGGLSPKHAPDIPDDSTDNITIFTRILDRLLDGYDNRLRPGLGDAVTEVKTDIYVT  100
E2QVJ7|GABRA3_Canis_lupus_familiaris MIVTQMSHFYTIILGLLFLINIFPGTTGQGESRRQEPGDFVKQDIGGLSPKHAPDIPDDSTDNITIFTRILDRLLDGYDNRLRPGLGDAVTEVKTDIYVT  100
A0A2R8QC15|GABRA3_Danio_rerio        -----------------------------------------HRAVTG----HREPIPDDSKDNITIFTRILDRLLDGYDNRLRPGLGESVTEVRTNIYVT   55
P10064|GABRA3_Bos_taurus             MIITQMSQFYMAGLGLLFLINILPGTTGQVESRRQEPGDFVKQDIGGLSPKHAPDIPDDSTDNITIFTRILDRLLDGYDNRLRPGLGDAVTEVKTDIYVT  100
                                     1.......10........20........30........40........50........60........70........80........90.......100

                                     ********************:*:**********::*****************************************************************
P34903|GABRA3_Homo_sapiens           SFGPVSDTDMEYTIDVFFRQTWHDERLKFDGPMKILPLNNLLASKIWTPDTFFHNGKKSVAHNMTTPNKLLRLVDNGTLLYTMRLTIHAECPMHLEDFPM  200
A0A2R9AHX2|GABRA3_Pan_paniscus       SFGPVSDTDMEYTIDVFFRQTWHDERLKFDGPMKILPLNNLLASKIWTPDTFFHNGKKSVAHNMTTPNKLLRLVDNGTLLYTMRLTIHAECPMHLEDFPM  200
H2QZ88|GABRA3_Pan_troglodytes        SFGPVSDTDMEYTIDVFFRQTWHDERLKFDGPMKILPLNNLLASKIWTPDTFFHNGKKSVAHNMTTPNKLLRLVDNGTLLYTMRLTIHAECPMHLEDFPM  200
F7G8R5|GABRA3_Macaca_mulatta         SFGPVSDTDMEYTIDVFFRQTWHDERLKFDGPMKILPLNNLLASKIWTPDTFFHNGKKSVAHNMTTPNKLLRLVDNGTLLYTMRLTIHAECPMHLEDFPM  200
M3YJX4|GABRA3_Mustela_putorius_furo  SFGPVSDTDMEYTIDVFFRQTWHDERLKFDGPMKILPLNNLLASKIWTPDTFFHNGKKSVAHNMTTPNKLLRLVDNGTLLYTMRLTIHAECPMHLEDFPM  200
P20236|GABRA3_Rattus_norvegicus      SFGPVSDTDMEYTIDVFFRQTWHDERLKFDGPMKILPLNNLLASKIWTPDTFFHNGKKSVAHNMTTPNKLLRLVDNGTLLYTMRLTIHAECPMHLEDFPM  200
P26049|GABRA3_Mus_musculus           SFGPVSDTDMEYTIDVFFRQTWHDERLKFDGPMKILPLNNLLASKIWTPDTFFHNGKKSVAHNMTTPNKLLRLVDNGTLLYTMRLTIHAECPMHLEDFPM  200
E2QVJ7|GABRA3_Canis_lupus_familiaris SFGPVSDTDMEYTIDVFFRQTWHDERLKFDGPMKILPLNNLLASKIWTPDTFFHNGKKSVAHNMTTPNKLLRLVDNGTLLYTMRLTIHAECPMHLEDFPM  200
A0A2R8QC15|GABRA3_Danio_rerio        SFGPVSDTDMEYTIDVFFRQSWRDERLKFDGPMQVLPLNNLLASKIWTPDTFFHNGKKSVAHNMTTPNKLLRLVDNGTLLYTMRLTIHAECPMHLEDFPM  155
P10064|GABRA3_Bos_taurus             SFGPVSDTDMEYTIDVFFRQTWHDERLKFDGPMKILPLNNLLASKIWTPDTFFHNGKKSVAHNMTTPNKLLRLVDNGTLLYTMRLTIHAECPMHLEDFPM  200
                                     .......110.......120.......130.......140.......150.......160.......170.......180.......190.......200

                                     *.*************. **:* ** ..:***.** **************:*.* * ***********:********************************
P34903|GABRA3_Homo_sapiens           DVHACPLKFGSYAYTTAEVVYSWTLGKNKSVEVAQDGSRLNQYDLLGHVVGTEIIRSSTGEYVVMTTHFHLKRKIGYFVIQTYLPCIMTVILSQVSFWLN  300
A0A2R9AHX2|GABRA3_Pan_paniscus       DVHACPLKFGSYAYTTAEVVYSWTLGKNKSVEVAQDGSRLNQYDLLGHVVGTEIIRSSTGEYVVMTTHFHLKRKIGYFVIQTYLPCIMTVILSQVSFWLN  300
H2QZ88|GABRA3_Pan_troglodytes        DVHACPLKFGSYAYTTAEVVYSWTLGKNKSVEVAQDGSRLNQYDLLGHVVGTEIIRSSTGEYVVMTTHFHLKRKIGYFVIQTYLPCIMTVILSQVSFWLN  300
F7G8R5|GABRA3_Macaca_mulatta         DVHACPLKFGSYAYTTAEVVYSWTLGKNKSVEVAQDGSRLNQYDLLGHVVGTEIIRSSTGEYVVMTTHFHLKRKIGYFVIQTYLPCIMTVILSQVSFWLN  300
M3YJX4|GABRA3_Mustela_putorius_furo  DVHACPLKFGSYAYTTAEVVYSWTLGKNKSVEVAQDGSRLNQYDLLGHVVGTEIIRSSTGEYVVMTTHFHLKRKIGYFVIQTYLPCIMTVILSQVSFWLN  300
P20236|GABRA3_Rattus_norvegicus      DVHACPLKFGSYAYTKAEVIYSWTLGKNKSVEVAQDGSRLNQYDLLGHVVGTEIIRSSTGEYVVMTTHFHLKRKIGYFVIQTYLPCIMTVILSQVSFWLN  300
P26049|GABRA3_Mus_musculus           DVHACPLKFGSYAYTKAEVIYSWTLGKNKSVEVAQDGSRLNQYDLLGHVVGTEIIRSSTGEYVVMTTHFHLKRKIGYFVIQTYLPCIMTVILSQVSFWLN  300
E2QVJ7|GABRA3_Canis_lupus_familiaris DVHACPLKFGSYAYTTAEVVYSWTLGKNKSVEVAQDGSRLNQYDLLGHVVGTEIIRSSTGEYVVMTTHFHLKRKIGYFVIQTYLPCIMTVILSQVSFWLN  300
A0A2R8QC15|GABRA3_Danio_rerio        DAHACPLKFGSYAYTNNEVVYIWTADDEKSVSVAPDGSRLNQYDLLGHVIGKETISSSTGEYVVMTTYFHLKRKIGYFVIQTYLPCIMTVILSQVSFWLN  255
P10064|GABRA3_Bos_taurus             DVHACPLKFGSYAYTTAEVVYSWTLGKNKSVEVAQDGSRLNQYDLLGHVVGTEIIRSSTGEYVVMTTHFHLKRKIGYFVIQTYLPCIMTVILSQVSFWLN  300
                                     .......210.......220.......230.......240.......250.......260.......270.......280.......290.......300

                                     *********** :                                               :*
P34903|GABRA3_Homo_sapiens           RESVPARTVFGVTTVLTMTTLSISARNSLPKVAYATAMDWFIAVCYAFVFSALIEFATVNYFTKRSWAWEGKKVPEALEMKKKTPAAPAKKTSTTFNIVG  400
A0A2R9AHX2|GABRA3_Pan_paniscus       RESVPARTVFGVTTVLTMTTLSISARNSLPKVAYATAMDWFIAVCYAFVFSALIEFATVNYFTKRSWAWEGKKVPEALEMKKKTPAAPAKKTSTTFNIVG  400
H2QZ88|GABRA3_Pan_troglodytes        RESVPARTVFGVTTVLTMTTLSISARNSLPKVAYATAMDWFIAVCYAFVFSALIEFATVNYFTKRSWAWEGKKVPEALEMKKKTPAAPAKKTSTTFNIVG  400
F7G8R5|GABRA3_Macaca_mulatta         RESVPARTVFGVTTVLTMTTLSISARNSLPKVAYATAMDWFIAVCYAFVFSALIEFATVNYFTKRSWAWEGKKVPEALEMKKKTPAAPAKKTSTTFNIVG  400
M3YJX4|GABRA3_Mustela_putorius_furo  RESVPARTVFGVTTVLTMTTLSISARNSLPKVAYATAMDWFIAVCYAFVFSALIEFATVNYFTKRSWAWEGKKVPEALEMKKKTPAAPTKKTSTTFNIVG  400
P20236|GABRA3_Rattus_norvegicus      RESVPARTVFGVTTVLTMTTLSISARNSLPKVAYATAMDWFIAVCYAFVFSALIEFATVNYFTKRSWAWEGKKVPEALEMKKKTPAAPTKKTSTTFNIVG  400
P26049|GABRA3_Mus_musculus           RESVPARTVFGVTTVLTMTTLSISARNSLPKVAYATAMDWFIAVCYAFVFSALIEFATVNYFTKRSWAWEGKKVPEALEMKKKTPAAPTKK-NTTFNIVG  399
E2QVJ7|GABRA3_Canis_lupus_familiaris RESVPARTVFGVTTVLTMTTLSISARNSLPKVAYATAMDWFIAVCYAFVFSALIEFATVNYFTKRSWAWEGKKVPEALEMKKKTPAAPTKKTSTTFNIVG  400
A0A2R8QC15|GABRA3_Danio_rerio        RESVPARTVFGKS-----------------------------------------------FF--------------------------------------  270
P10064|GABRA3_Bos_taurus             RESVPARTVFGVTTVLTMTTLSISARNSLPKVAYATAMDWFMAVCYAFVFSALIEFATVNYFTKRSWAWEGKKVPEALEMKKKTPAVPTKKTSTTFNIVG  400
                                     .......310.......320.......330.......340.......350.......360.......370.......380.......390.......400

                                       **                                                                  * :**
P34903|GABRA3_Homo_sapiens           TTYPINLAKDTEFSTISK-GAAPSASSTPTIIASPKATYVQDSPTETKTYNSVSKVDKISRIIFPVLFAIFNLVYWATYVNRESAIKGMIRKQ  492
A0A2R9AHX2|GABRA3_Pan_paniscus       TTYPINLAKDTEFSTISK-GAAPSASSTPTIIASPKATYVQDSPTETKTYNSVSKVDKISRIIFPVLFAIFNLVYWATYVNRESAIKGMIRKQ  492
H2QZ88|GABRA3_Pan_troglodytes        TTYPINLAKDTEFSTISK-GAAPSASSTPTIIASPKATYVQDSPTETKTYNSVSKVDKISRIIFPVLFAIFNLVYWATYVNRESAIKGMIRKQ  492
F7G8R5|GABRA3_Macaca_mulatta         TTYPINLAKDTEFSTISK-GAAPSASSTPTIIASPKATYVQDSPTETKTYNSVSKVDKISRIIFPVLFAIFNLVYWATYVNRESAIKGMIRKQ  492
M3YJX4|GABRA3_Mustela_putorius_furo  TTYPINLAKDTEFSTISK-GAAPSASSTPTIIASPKTTYVQDIPTETKTYNSVSKVDKISRIIFPVLFAIFNLVYWATYVNRESAIKGMIRKH  492
P20236|GABRA3_Rattus_norvegicus      TTYPINLAKDTEFSTISKAAAAPSASSTPTVIASPKTTYVQDSPAETKTYNSVSKVDKISRIIFPVLFAIFNLVYWATYVNRESAIKGMIRKQ  493
P26049|GABRA3_Mus_musculus           TTYPINLAKDTEFSTISKSAAAPSASSTPTAIASPKATYVQDSPAETKTYNSVSKVDKISRIIFPVLFAIFNLVYWATYVNRESAIKGMIRKQ  492
E2QVJ7|GABRA3_Canis_lupus_familiaris TTYPINLAKDTEFSTISK-GAAPSASSTPTIIASPKTTYVQDIPTETKTYNSVSKVDKISRIIFPVLFAIFNLVYWATYVNRESAIKGMIRKH  492
A0A2R8QC15|GABRA3_Danio_rerio        --YP------------------------------------------------------------------FAVVY------------------  277
P10064|GABRA3_Bos_taurus             TTYPINLAKDTEFSAISK-GAAPSTSSTPTIIASPKTTCVQDIPTETKTYNSVSKVDKISRIIFPVLFAIFNLVYWATYVNRESAIKGMIRKQ  492
                                     .......410.......420.......430.......440.......450.......460.......470.......480.......490...
```

```
                                                .        *  .*.:.  :   . :  : :                           ********************************
P48169|GABRA4_Homo_sapiens             MVSAKKVPAIALSAGVSFALLRFLCLAVCLNESPGQNQKEEKLCTENFTRILDSLLDGYDNRLRPGFGGPVTEVKTDIYVTSFGPVSDVEMEYTMDVFFR  100
A0A2R9C6M3|GABRA4_Pan_paniscus         MVSAKKVPAIALSAGVSFALLRFLCLAVCLNESPGQNQKEEKLCTENFTRILDSLLDGYDNRLRPGFGGPVTEVKTDIYVTSFGPVSDVEMEYTMDVFFR  100
H2QPE7|GABRA4_Pan_troglodytes          MVSAKKVPAIALSAGVSFALLRFLCLAVCLNESPGQNQKEEKLCTENFTRILDSLLDGYDNRLRPGFGGPVTEVKTDIYVTSFGPVSDVEMEYTMDVFFR  100
F6YLN4|GABRA4_Macaca_mulatta           MVSAKKVPAIALSAGVSFALLRFLCLAVCLNESPGQNQKEEKLCTENFTRILDSLLDGYDNRLRPGFGGPVTEVKTDIYVTSFGPVSDVEMEYTMDVFFR  100
M3Y0X5|GABRA4_Mustela_putorius_furo    MVSAKKVPAIATSSRVSFALLHFLCLAACLNESPGQNQKEEKLCPENFTRILDSLLDGYDNRLRPGFGGPVTEVKTDIYVTSFGPVSDVEMEYTMDVFFR  100
P28471|GABRA4_Rattus_norvegicus        MVSVQKVPAIVLCSGVSLALLHVLCLATCLNESPGQNSKDEKLCPENFTRILDSLLDGYDNRLRPGFGGPVTEVKTDIYVTSFGPVSDVEMEYTMDVFFR  100
Q9D6F4|GABRA4_Mus_musculus             MVSVQKVPAIALCSGVSLALLHFLCLAACLNESPGQNSKDEKLCPENFTRILDSLLDGYDNRLRPGFGGPVTEVKTDIYVTSFGPVSDVEMEYTMDVFFR  100
F1P9P9|GABRA4_Canis_lupus_familiaris   ---------MVMTTGCALDLGGLLSLVSHV--LPLEKKRGK---------------------------GPVTEVKTDIYVTSFGPVSDVEMEYTMDVFFR   62
Q568M9|GABRA4_Danio_rerio              MVSAKKEMVTAMYPTSISTLLYFLCVAACIKRISGQIRKDEKLYPENFTRILDRLLDGYDNRLRPGFGGPVTEVKTDIYVTSFGPVSDVEMEYTMDVFFR  100
P20237|GABRA4_Bos_taurus               MVSAKKVPAIAMSFGVSFALLHFLCLAACLNESPGQNQKEEKLCPENFTRILDSLLDGYDNRLRPGFGGPVTEVKTDIYVTSFGPVSDVEMEYTMDVFFR  100
                                       1.......10........20........30........40........50........60........70........80........90.......100

                                       ***:*:**:*:**:*******:***************:**:*******:*****:*******************:******:***********:**
P48169|GABRA4_Homo_sapiens             QTWIDKRLKYDGPIEILRLNNMMVTKVWTPDTFFRNGKKSVSHNMTAPNKLFRIMRNGTILYTMRLTISAECPMRLVDFPMDGHACPLKFGSYAYPKSEM  200
A0A2R9C6M3|GABRA4_Pan_paniscus         QTWIDKRLKYDGPIEILRLNNMMVTKVWTPDTFFRNGKKSVSHNMTAPNKLFRIMRNGTILYTMRLTISAECPMRLVDFPMDGHACPLKFGSYAYPKSEM  200
H2QPE7|GABRA4_Pan_troglodytes          QTWIDKRLKYDGPIEILRLNNMMVTKVWTPDTFFRNGKKSVSHNMTAPNKLFRIMRNGTILYTMRLTISAECPMRLVDFPMDGHACPLKFGSYAYPKSEM  200
F6YLN4|GABRA4_Macaca_mulatta           QTWIDKRLKYDGPIEILRLNNMMVTKVWTPDTFFRNGKKSVSHNMTAPNKLFRIMRNGTILYTMRLTISAECPMRLVDFPMDGHACPLKFGSYAYPKSEM  200
M3Y0X5|GABRA4_Mustela_putorius_furo    QTWIDKRLKYDGPIEILRLNNMMVTKVWTPDTFFRNGKKSVSHNMTAPNKLFRIMRNGTILYTMRLTISAECPMRLVDFPMDGHACPLKFGSYAYPKSEM  200
P28471|GABRA4_Rattus_norvegicus        QTWIDKRLKYDGPIEILRLNNMMVTKVWTPDTFFRNGKKSVSHNMTAPNKLFRIMRNGTILYTMRLTISAECPMRLVDFPMDGHACPLKFGSYAYPKSEM  200
Q9D6F4|GABRA4_Mus_musculus             QTWIDKRLKYDGPIEILRLNNMMVTKVWTPDTFFRNGKKSVSHNMTAPNKLFRIMRNGTILYTMRLTISAECPMRLVDFPMDGHACPLKFGSYAYPKSEM  200
F1P9P9|GABRA4_Canis_lupus_familiaris   QTWIDKRLKYDGPIEILRLNNMMVTKVWTPDTFFRNGKKSVSHNMTAPNKLFRIMRNGTILYTMRLTISAECPMRLVDFPMDGHACPLKFGSYAYPKSEM  162
Q568M9|GABRA4_Danio_rerio              QTWVDRRLRYEGPVEILRLNNLMVTKVWTPDTFFRNGKRSVAHNMTAPNRLFRIMKNGTILYTMRLTISAECPMRLVNFPMDGHSCPLKFGSYAYPKTEM  200
P20237|GABRA4_Bos_taurus               QTWIDKRLKYDGPIEILRLNNMMVTKVWTPDTFFRNGKKSVSHNMTAPNKLFRIMRNGTILYTMRLTISAECPMRLVDFPMDGHACPLKFGSYAYPKSEM  200
                                       .......110.......120.......130.......140.......150.......160.......170.......180.......190.......200

                                       ********:.***** ***********:********:*******:********:*******************************************
P48169|GABRA4_Homo_sapiens             IYTWTKGPEKSVEVPKESSSLVQYDLIGQTVSSETIKSITGEYIVMTVYFHLRRKMGYFMIQTYIPCIMTVILSQVSFWINKESVPARTVFGITTVLTMT  300
A0A2R9C6M3|GABRA4_Pan_paniscus         IYTWTKGPEKSVEVPKESSSLVQYDLIGQTVSSETIKSITGEYIVMTVYFHLRRKMGYFMIQTYIPCIMTVILSQVSFWINKESVPARTVFGITTVLTMT  300
H2QPE7|GABRA4_Pan_troglodytes          IYTWTKGPEKSVEVPKESSSLVQYDLIGQTVSSETIKSITGEYIVMTVYFHLRRKMGYFMIQTYIPCIMTVILSQVSFWINKESVPARTVFGITTVLTMT  300
F6YLN4|GABRA4_Macaca_mulatta           IYTWTKGPEKSVEVPKESSSLVQYDLIGQTVSSETIKSITGEYIVMTVYFHLRRKMGYFMIQTYIPCIMTVILSQVSFWINKESVPARTVFGITTVLTMT  300
M3Y0X5|GABRA4_Mustela_putorius_furo    IYTWTKGPEKSVEVPKESSSLVQYDLVGQTVSSETIKSITGEYVVMTVYFHLRRKMGYFMIQTYIPCIMTVILSQVSFWINKESVPARTVFGITTVLTMT  300
P28471|GABRA4_Rattus_norvegicus        IYTWTKGPEKSVEVPKESSSLVQYDLIGQTVSSETIKSITGEYIVMTVYFHLRRKMGYFMIQTYIPCIMTVILSQVSFWINKESVPARTVFGITTVLTMT  300
Q9D6F4|GABRA4_Mus_musculus             IYTWTKGPEKSVEVPKESSSLVQYDLIGQTVSSETIKSITGEYIVMTVYFHLRRKMGYFMIQTYIPCIMTVILSQVSFWINKESVPARTVFGITTVLTMT  300
F1P9P9|GABRA4_Canis_lupus_familiaris   IYTWTKGPEKSVEVPKESSSLVQYDLVGQTVSSETIKSITGEYIVMTVYFHLRRKMGYFMIQTYIPCIMTVILSQVSFWINKESVPARTVFGITTVLTMT  262
Q568M9|GABRA4_Danio_rerio              IYTWTKGPQHSVEVPPESSSLVQYDLIGQTVSSETVKSITGEYVVMTVYFHLRKKMGYFMIQTYIPCIMTVILSQVSFWINKESVPARTVFGITTVLTMT  300
P20237|GABRA4_Bos_taurus               IYTWTKGPEKSVEVPKESSSLVQYDLIGQTVSSETIKSITGEYIVMTVYFHLRRKMGYFMIQTYIPCIMTVILSQVSFWINKESVPARTVFGITTVLTMT  300
                                       .......210.......220.......230.......240.......250.......260.......270.......280.......290.......300

                                       ********************************************* *   :**:* :*.    .. :.. ::* .*  * **:.:* .:***.*: :  ::
P48169|GABRA4_Homo_sapiens             TLSISARHSLPKVSYATAMDWFIAVCFAFVFSALIEFAAVNYFTNIQMEKAKRKTSKPPQEVPA-APVQREKHPEAPL-QNTNANLNMRKRTNALV--HS  396
A0A2R9C6M3|GABRA4_Pan_paniscus         TLSISARHSLPKVSYATAMDWFIAVCFAFVFSALIEFAAVNYFTNIQMEKAKRKTSKPPQEVPA-APVQREKHPEAPL-QNTNANLNMRKRTNALV--HS  396
H2QPE7|GABRA4_Pan_troglodytes          TLSISARHSLPKVSYATAMDWFIAVCFAFVFSALIEFAAVNYFTNIQMEKAKRKTSKPPQEVPA-APVQREKHPEAPL-QNTNANLNMRKRTNALV--HS  396
F6YLN4|GABRA4_Macaca_mulatta           TLSISARHSLPKVSYATAMDWFIAVCFAFVFSALIEFAAVNYFTNIQMEKAKRKTSKPPQEVPA-APVQREKHPEAPL-QNTNANLNMRKRTNASV--HS  396
M3Y0X5|GABRA4_Mustela_putorius_furo    TLSISARHSLPKVSYATAMDWFIAVCFAFVFSALIEFAAVNYFTNVQMAKAKRKSSKALQEIPA-APVQREKPPEAPL-QNTNANLNMRKRTNASV--HS  396
P28471|GABRA4_Rattus_norvegicus        TLSISARHSLPKVSYATAMDWFIAVCFAFVFSALIEFAAVNYFTNIQMQKAKKKISKPPPEVPA-APVLKEKHTETSL-QNTHANLNMRKRTNALV--HS  396
Q9D6F4|GABRA4_Mus_musculus             TLSISARHSLPKVSYATAMDWFIAVCFAFVFSALIEFAAVNYFTNIQMQKAKKKISKPPPEVPA-APVLKEKHTETSL-QNTHANLNMRKRTNALV--HS  396
F1P9P9|GABRA4_Canis_lupus_familiaris   TLSISARHSLPKVSYATAMDWFIAVCFAFVFSALIEFAAVNYFTNIQMEKAKRKSSKALQEIPA-APVQREKHPEAPL-QNTNANLNMRKRTNASV--HS  358
Q568M9|GABRA4_Danio_rerio              TLSISARHSLPKVSYATAMDWFIAVCFAFVFSALIEFAAVNYFTNAQAERAKRKQAKAAAAASVKSSTGKNKDTEEVLQQNSDTNGNLRKRVNSNIQPQA  400
P20237|GABRA4_Bos_taurus               TLSISARHSLPKVSYATAMDWFIAVCFAFVFSALIEFAAVNYFTNVQMEKAKRKTSKAPQEISA-APVLREKHPETPL-QNTNANLSMRKRANALV--HS  396
                                       .......310.......320.......330.......340.......350.......360.......370.......380.......390.......400

                                       **.   ** .     *  *** : . * **      : :::     **.. *:.*** : * *::    .*  ..:                 *. :  :*.:* .
P48169|GABRA4_Homo_sapiens             ESDVGNRTEV----GNHSSKSSTVVQESSKGTPRSYLAS----SPNPFSRANAAETIS-AARALPSASPTS--IRTGYMPRKASVGSASTRHVFGSRLQR  485
A0A2R9C6M3|GABRA4_Pan_paniscus         ESDVGNRTEV----GNHSSKSSTVVQESSKGTPRSYLAS----SPNPFSRANAAETIS-AARALPSASPTS--IRTGYMPRKASVGSASTRHVFGSRLQR  485
H2QPE7|GABRA4_Pan_troglodytes          ESDVGNRTEV----GNHSSKSSTVVQESSKGTPRSYLAS----SPNPFSRANAAETIS-AARALPSASPTS--IRTGYMPRKASVGSASTRHVFGSRLQR  485
F6YLN4|GABRA4_Macaca_mulatta           ESDVGNRTVV----GNHSSKSSTVVQESSKGTPRSYLAS----SPNPFSRANAAETIS-AARALPSASPAS--SRTGYVSRKASIASASTRHVFGSRLQR  485
M3Y0X5|GABRA4_Mustela_putorius_furo    ESDAGHRTEV----GNHSSKSSSVTQGSSEATSQSYLAS----SPNPFSHPNAAETIS-AARAPLSAPPSTP-GRTGYVSQK-----ASTHRVFGSRLER  481
P28471|GABRA4_Rattus_norvegicus        ESDVNSRTEV----GNHSSK-TTAAQESSETTPKAHLAS----SPNPFSRANAAETISAAARGLSSAASPS---PHGTL-QPAPLRSASARPAFGARLGR  483
Q9D6F4|GABRA4_Mus_musculus             ESDVKSRTEV----GNHSSK-TSAVQESSEATPKAHLAS----SPNPFSRANAAETMSAAARGLSSAASPS---PHGTL-RPASLGSASTRPAFGSRLGR  483
F1P9P9|GABRA4_Canis_lupus_familiaris   ESDVGNRTEV----GNHSSKSSTVIQGSSEATPQSYLAA----SPNPFSHANAAETIS-AARAPLSAPSSTP-SRTGYVSQQASVGSASTHRVFGSRLER  448
Q568M9|GABRA4_Danio_rerio              ESKKTQRTEASSKSGASSSSKPTLTSQSSSTSEGTSSFSSQSRLSPSSASQSNAAASSSSASKTTPPAPPSTPTVPDKPSPGTTPAGSAPLHHLLGPKLEN  500
P20237|GABRA4_Bos_taurus               ESDVGSRTDV----GNHSSKSSTVVQGSSEATPQSYLAS----SPNPFSRANAAETIS-AARAIPSALPSTP-SRTGYVPRQVPVGSASTQHVFGSRLQR  486
                                       .......410.......420.......430.......440.......450.......460.......470.......480.......490.......500
```

```
                                     **          : .* : . .:.*..* *:****:********:******************* . :
P48169|GABRA4_Homo_sapiens           IKTTVNTIGATGKLSA-TPPPSAPPPSGSGTSKIDKYARILFPVTFGAFNMVYWVVYLSKDTMEKSESLM   554
A0A2R9C6M3|GABRA4_Pan_paniscus       IKTTVNTIGATGKLSA-TPPPSTPPPSGSGTSKIDKYARILFPVTFGAFNMVYWVVYLSKDTMEKSESLM   554
H2QPE7|GABRA4_Pan_troglodytes        IKTTVNTIGATGKLSA-TPPPSAPPPSGSGTSKIDKYARILFPVTFGAFNMVYWVVYLSKDTMEKSESLM   554
F6YLN4|GABRA4_Macaca_mulatta         IKTTVNTIGATGKLSA-TPPPSAPPPSGSGTSKIDKYARILFPVTFGAFNMVYWVVYLSKDTMEKSESLM   554
M3Y0X5|GABRA4_Mustela_putorius_furo  IKTTVNTTGAPGKLSATTPPPPAPPPPGSGTSKIDKYARILFPVTFGAFNMVYWVVYLSKDTMEKSESLM   551
P28471|GABRA4_Rattus_norvegicus      IKTTVNTTGVPGNVSA-TPPPSAPPPSGSGTSKIDKYARILFPVTFGAFNMVYWVVYLSKDTMEKSESLM   552
Q9D6F4|GABRA4_Mus_musculus           IKTTVNTTGAAGNVSA-TPPPPAPPPSGSGTSKIDKYARILFPVTFGAFNMVYWVVYLSKDTMEKSESLM   552
F1P9P9|GABRA4_Canis_lupus_familiaris IKTTVNTIGASGKLSA-TTPPSAPPPSGSGTSKIDKYARILFPVTFGAFNMVYWVVYLSKDTMEKSESLM   517
Q568M9|GABRA4_Danio_rerio            IK----------KKEAKAQPQPAAPATG-GASKIDEYARILFPVSFGAFNMVYWVVYLSKDTMEAKGA--   557
P20237|GABRA4_Bos_taurus             IKTTVNSIGTSGKLSA-TTTPSAPPPSGSGTSKIDKYARILFPVTFGAFNMVYWVVYLSKDTMEKSESLM   555
                                     .......510.......520.......530.......540.......550.......560.......570
```

```
                                      *. *  .   * :.* :  : *.*:.*:.::* .*.: :  * :****:*********************::*:::*:*:*******************
P31644|GABRA5_Homo_sapiens            MDNGMFSGFIMIKNLLLFCISMNLSSHFGFSQMPTSSVKD-ETNDNITIFTRILDGLLDGYDNRLRPGLGERITQVRTDIYVTSFGPVSDTEMEYTIDVF   99
A0A2R9BFW8|GABRA5_Pan_paniscus        MDNGMFSGFIMIKNLLLFCISMNLSSHFGFSQMPTSSVKD-ETNDNITIFTRILDGLLDGYDNRLRPGLGERITQVRTDIYVTSFGPVSDTEMEYTIDVF   99
H2Q919|GABRA5_Pan_troglodytes         MDNGMFSGFIMIKNLLLFCISMNLSSHFGFSQMPTSSVKD-ETNDNITIFTRILDGLLDGYDNRLRPGLGERITQVRTDIYVTSFGPVSDTEMEYTIDVF   99
G7MW76|GABRA5_Macaca_mulatta          MDNGMFSGFIMIKNLLLFCISMNLSSHFGFSQMPTSSVKD-ETNDNITIFTRILDGLLDGYDNRLRPGLGERITQVRTDIYVTSFGPVSDTEMEYTIDVF   99
M3XNJ0|GABRA5_Mustela_putorius_furo   MDNGMFSSFIMIKNLLLFCISMNLASHFGFSQMPTSSVKD-ETNDNITIFTRILDGLLDGYDNRLRPGLGERITQVRTDIYVTSFGPVSDTEMEYTIDVF   99
P19969|GABRA5_Rattus_norvegicus       MDNGMLSRFIMTKTLLVFCISMTLSSHFGFSQMPTSSVQD-ETNDNITIFTRILDGLLDGYDNRLRPGLGERITQVRTDIYVTSFGPVSDTEMEYTIDVF   99
Q8BHJ7|GABRA5_Mus_musculus            MDNGMLSRFIMTQTLLVFCISMTLSSHFGFSQMPTSSVQD-ETNDNITIFTRILDGLLDGYDNRLRPGLGERITQVRTDIYVTSFGPVSDTEMEYTIDVF   99
E2RG38|GABRA5_Canis_lupus_familiaris  MDNGMFSSFIMIKNLLLFCISMNLASHFGFAQMPTSSVKD-ETNDNITIFTRILDGLLDGYDNRLRPGLGERITQVRTDIYVTSFGPVSDTEMEYTIDVF   99
E9QE70|GABRA5_Danio_rerio             MGYGPHCSGKM-RSLWVGVLFMTLTCHLSLSQSTTGTPKESELNDNITVFTRILDGLLDGYDNRLRPGLGEKVTEIKTNIFVTSFGPVSDTEMEYTIDVF   99
Q08E50|GABRA5_Bos_taurus              MDNGMFSSFIMIKNLLLFCISMNLASHFGFSQMPTSSVKA-ETDDNITIFTRILDGLLDGYDNRLRPGLGERITQVRTDIYVTSFGPVSDTEMEYTIDVF   99
                                      1.......10........20........30........40........50........60........70........80........90.......100

                                      ********** *****: *********:******** ********************:*************************************** *
P31644|GABRA5_Homo_sapiens            FRQSWKDERLRFKGPMQRLPLNNLLASKIWTPDTFFHNGKKSIAHNMTTPNKLLRLEDDGTLLYTMRLTISAECPMQLEDFPMDAHACPLKFGSYAYPNS   199
A0A2R9BFW8|GABRA5_Pan_paniscus        FRQSWKDERLRFKGPMQRLPLNNLLASKIWTPDTFFHNGKKSIAHNMTTPNKLLRLEDDGTLLYTMRLTISAECPMQLEDFPMDAHACPLKFGSYAYPNS   199
H2Q919|GABRA5_Pan_troglodytes         FRQSWKDERLRFKGPMQRLPLNNLLASKIWTPDTFFHNGKKSIAHNMTTPNKLLRLEDDGTLLYTMRLTISAECPMQLEDFPMDAHACPLKFGSYAYPNS   199
G7MW76|GABRA5_Macaca_mulatta          FRQSWKDERLRFKGPMQRLPLNNLLASKIWTPDTFFHNGKKSIAHNMTTPNKLLRLEDDGTLLYTMRLTISAECPMQLEDFPMDAHACPLKFGSYAYPNS   199
M3XNJ0|GABRA5_Mustela_putorius_furo   FRQSWKDERLRFKGPMQRLPLNNLLASKIWTPDTFFHNGKKSIAHNMTTPNKLLRLEDDGTLLYTMRLTISAECPMQLEDFPMDAHACPLKFGSYAYPNS   199
P19969|GABRA5_Rattus_norvegicus       FRQSWKDERLRFKGPMQRLPLNNLLASKIWTPDTFFHNGKKSIAHNMTTPNKLLRLEDDGTLLYTMRLTISAECPMQLEDFPMDAHACPLKFGSYAYPNS   199
Q8BHJ7|GABRA5_Mus_musculus            FRQSWKDERLRFKGPMQRLPLNNLLASKIWTPDTFFHNGKKSIAHNMTTPNKLLRLEDDGTLLYTMRLTISAECPMQLEDFPMDAHACPLKFGSYAYPNS   199
E2RG38|GABRA5_Canis_lupus_familiaris  FRQSWKDERLRFKGPMQRLPLNNLLASKIWTPDTFFHNGKKSIAHNMTTPNKLLRLEDDGTLLYTMRLTISAECPMQLEDFPMDAHACPLKFGSYAYPNS   199
E9QE70|GABRA5_Danio_rerio             FRQSWKDERLCFKGPMEMLPLNNLLASNIWTPDTFFLNGKKSIAHNMTTPNKLLRLKDDGTLLYTMRLTISAECPMQLEDFPMDAHACPLKFGSYAYPIS   199
Q08E50|GABRA5_Bos_taurus              FRQSWKDERLRFKGPMQRLPLNNLLASKIWTPDTFFHNGKKSIAHNMTTPNKLLRLEDDGTLLYTMRLTISAECPMQLEDFPMDAHACPLKFGSYAYPNS   199
                                      .......110.......120.......130.......140.......150.......160.......170.......180.......190.......200

                                      **:* **:*. *******:*********:*:*.***:**** *:**:* ***:*************:**:*********************************
P31644|GABRA5_Homo_sapiens            EVVYVWTNGSTKSVVVAEDGSRLNQYHLMGQTVGTENISTSTGEYTIMTAHFHLKRKIGYFVIQTYLPCIMTVILSQVSFWLNRESVPARTVFGVTTVLT   299
A0A2R9BFW8|GABRA5_Pan_paniscus        EVVYVWTNGSTKSVVVAEDGSRLNQYHLMGQTVGTENISTSTGEYTIMTAHFHLKRKIGYFVIQTYLPCIMTVILSQVSFWLNRESVPARTVFGVTTVLT   299
H2Q919|GABRA5_Pan_troglodytes         EVVYVWTNGSTKSVVVAEDGSRLNQYHLMGQTVGTENISTSTGEYTIMTAHFHLKRKIGYFVIQTYLPCIMTVILSQVSFWLNRESVPARTVFGVTTVLT   299
G7MW76|GABRA5_Macaca_mulatta          EVVYVWTNGSTKSVVVAEDGSRLNQYHLMGQTVGTENISTSTGEYTIMTAHFHLKRKIGYFVIQTYLPCIMTVILSQVSFWLNRESVPARTVFGVTTVLT   299
M3XNJ0|GABRA5_Mustela_putorius_furo   EVVYVWTNGTTKSVVVAEDGSRLNQYHLMGQTVGTENISTSTGEYTVMTAHFHLKRKIGYFVIQTYLPCIMTVILSQVSFWLNRESVPARTVFGVTTVLT   299
P19969|GABRA5_Rattus_norvegicus       EVVYVWTNGSTKSVVVAEDGSRLNQYHLMGQTVGTENISTSTGEYTIMTAHFHLKRKIGYFVIQTYLPCIMTVILSQVSFWLNRESVPARTVFGVTTVLT   299
Q8BHJ7|GABRA5_Mus_musculus            EVVYVWTNGSTKSVVVAEDGSRLNQYHLMGQTVGTENISTSTGEYTIMTAHFHLKRKIGYFVIQTYLPCIMTVILSQVSFWLNRESVPARTVFGVTTVLT   299
E2RG38|GABRA5_Canis_lupus_familiaris  EVVYVWTNGTTKSVVVAEDGSRLNQYHLMGQTVGTENISTSTGEYTIMTAHFHLKRKIGYFVIQTYLPCIMTVILSQVSFWLNRESVPARTVFGVTTVLT   299
E9QE70|GABRA5_Danio_rerio             EVVYKWTKGPGKSVVVAEEGSRLNQYHLIGHTAGTEDISTSRGQYTVMMAHFYLKRKIGYFVIQTYMPCFMTVILSQVSFWLNRESVPARTVFGVTTVLT   299
Q08E50|GABRA5_Bos_taurus              EVIYVWTNGTAKSVVVAEDGSRLNQYHLMGQTVGTENISTSTGEYTIMTAHFHLKRKIGYFVIQTYLPCIMTVILSQVSFWLNRESVPARTVFGVTTVLT   299
                                      .......210.......220.......230.......240.......250.......260.......270.......280.......290.......300

                                      *******************************************.******** ** : ***   : :.*..* :*:      ** *:   .
P31644|GABRA5_Homo_sapiens            MTTLSISARNSLPKVAYATAMDWFIAVCYAFVFSALIEFATVNYFTKRGWAWDGKKALEAAKIKKK-REVILNKSTNAFTTGKMSHPPNIPKEQTPAGTS   398
A0A2R9BFW8|GABRA5_Pan_paniscus        MTTLSISARNSLPKVAYATAMDWFIAVCYAFVFSALIEFATVNYFTKRGWAWDGKKALEAAKIKKK-REVILNKSTNAFTTGKMSHPPNIPKEQTPAGTS   398
H2Q919|GABRA5_Pan_troglodytes         MTTLSISARNSLPKVAYATAMDWFIAVCYAFVFSALIEFATVNYFTKRGWAWDGKKALEAAKIKKK-REVILNKSTNAFTTGKMSHPPNIPKEQTPAGTS   398
G7MW76|GABRA5_Macaca_mulatta          MTTLSISARNSLPKVAYATAMDWFIAVCYAFVFSALIEFATVNYFTKRGWAWDGKKALEAAKIKKK-REVMLNKSTNAFTAGKMSHPPNIPKEQTPAGTS   398
M3XNJ0|GABRA5_Mustela_putorius_furo   MTTLSISARNSLPKVAYATAMDWFIAVCYAFVFSALIEFATVNYFTKRGWAWDGKKASEAAKVKKKERELILNKSTNAYTTGKMTHPPNIPKEQTPAGTS   399
P19969|GABRA5_Rattus_norvegicus       MTTLSISARNSLPKVAYATAMDWFIAVCYAFVFSALIEFATVNYFTKRGWAWDGKKALEAAKIKKKERELILNKSTNAFTTGKLTHPPNIPKEQLPGGTG   399
Q8BHJ7|GABRA5_Mus_musculus            MTTLSISARNSLPKVAYATAMDWFIAVCYAFVFSALIEFATVNYFTKRGWAWDGKKALEAAKIKKKERELILNKSTNAFTTGKLTHPPNIPKEQPPAGTA   399
E2RG38|GABRA5_Canis_lupus_familiaris  MTTLSISARNSLPKVAYATAMDWFIAVCYAFVFSALIEFATVNYFTKRGWAWDGKKASEAAKVKKKERELILNKSTNAYTTGKMTHPPNIPKEQTPAVTS   399
E9QE70|GABRA5_Danio_rerio             MTTLSISARNSLPKVAYATAMDWFIAVCYAFVFSALIEFATVNYFTKRSWAWDGKKALEAQQPKKK-DPLALSKKPNNFTA-------NINKDAAISTIS   391
Q08E50|GABRA5_Bos_taurus              MTTLSISARNSLPKVAYATAMDWFIAVCYAFVFSALIEFATVNYFTKRGWAWDGKKALEAAKIKKKERELTINKSTNAYTTGKMTHPPNIPKEQTPAGTT   399
                                      .......310.......320.......330.......340.......350.......360.......370.......380.......390.......400

                                      *:  :..::. * *  :.: ******:*********:**:*********************.
P31644|GABRA5_Homo_sapiens            NTT-SVSVKPSEEK-TSESKKTYNSISKIDKMSRIVFPVLFGTFNLVYWATYLNREPVIKGAASPK   462
A0A2R9BFW8|GABRA5_Pan_paniscus        NTT-SVSVKPSEEK-TSESKKTYNSISKIDKMSRIVFPVLFGTFNLVYWATYLNREPVIKGAASPK   462
H2Q919|GABRA5_Pan_troglodytes         NTT-SVSVKPSEEK-TSESKKTYNSISKIDKMSRIVFPVLFGTFNLVYWATYLNREPVIKGAASPK   462
G7MW76|GABRA5_Macaca_mulatta          NTT-SVSVKPSEEK-TPESKKTYNSISKIDKMSRIVFPVLFGTFNLVYWATYLNREPVIKGAASPK   462
M3XNJ0|GABRA5_Mustela_putorius_furo   NAS-SASVKPSEEK-TSENKKTYNSISKIDKMSRIVFPVLFGTFNLVYWATYLNREPVIKGAASPK   463
P19969|GABRA5_Rattus_norvegicus       NAVGTASIRASEEK-TSESKKTYNSISKIDKMSRIVFPILFGTFNLVYWATYLNREPVIKGATSPK   464
Q8BHJ7|GABRA5_Mus_musculus            NAP-TVSIKASEEK-TAESKKTYNSISKIDKMSRIVFPILFGTFNLVYWATYLNREPVIKGATSPK   463
E2RG38|GABRA5_Canis_lupus_familiaris  NAS-SASVKPSEEK-TSENKKTYNSISKIDKMSRIVFPVLFGTFNLVYWATYLNREPVIKGATSPK   463
E9QE70|GABRA5_Danio_rerio             NST-TVQLKSAEAKPAPDPKKTYNSVSKIDKMSRIVFPVLFGTFNLVYWATYLNREPVIKGAV---   453
Q08E50|GABRA5_Bos_taurus              NAS-SASVKP-EDK-ASENKKTYNSISKIDKMSRIIFPLLFGTFNLVYWATYLNREPVIKGATSPK   462
                                      .......410.......420.......430.......440.......450.......460.....
```

```
                                     *      *     : .. .        :*  *::****.**:************.*********:*********************** *:
Q16445|GABRA6_Homo_sapiens           MASSLPWLCIILWLENALGKLEVEGNFYSENVSRILDNLLEGYDNRLRPGFGGAVTEVKTDIYVTSFGPVSDVEMEYTMDVFFRQTWTDERLKFGGPTEI 100
A0A2R8ZQ75|GABRA6_Pan_paniscus       MASSLPWLCIILWLENALGKLEVEGNFYSENVSRILDNLLEGYDNRLRPGFGGAVTEVKTDIYVTSFGPVSDVEMEYTMDVFFRQTWTDERLKFGGPTEI 100
H2QRY3|GABRA6_Pan_troglodytes        MASSLPWLCIILWLENALGKLEVGGNFYSENVSRILDNLLEGYDNRLRPGFGGAVTEVKTDIYVTSFGPVSDVEMEYTMDVFFRQTWTDERLKFGGPTEI 100
A0A1D5RL15|GABRA6_Macaca_mulatta     MASPLPWLCIILWLESALGKFEDEGNFYSENVSRILDNLLEGYDNRLRPGFGGAVTEVKTDIYVTSFGPVSDVEMEYTMDVFFRQTWTDERLKFGGPTEI 100
M3YP65|GABRA6_Mustela_putorius_furo  MASPLLWLYILLWVENTLGTLEDEESFYSRNISRILDNLLEGYDNRLRPGFGGAVTEVKTDIYVTSFGPVSDVEMEYTMDVFFRQTWTDERLKFGGPTEI 100
P30191|GABRA6_Rattus_norvegicus      MLLLLPWLFSLLWIENAQAQLEDEGNFYSENVSRILDNLLEGYDNRLRPGFGGAVTEVKTDIYVTSFGPVSDVEMEYTMDVFFRQTWTDERLKFKGPAEI 100
P16305|GABRA6_Mus_musculus           MVLLLPWLFIILWLENAQAQLEDEGNFYSENVSRILDNLLEGYDNRLRPGFGGAVTEVKTDIYVTSFGPVSDVEMEYTMDVFFRQTWTDERLKFKGPAEI 100
E2RSN3|GABRA6_Canis_lupus_familiaris MASPLPLLYIILWVENTLGKLEDEGNFYSKNISRILDNLLEGYDNRLRPGFGGAVTEVKTDIYVTSFGPVSDVEMEYTMDVFFRQTWTDERLKFGGPTEI 100
F1QGW0|GABRA6b_Danio_rerio           MAWVRTCLFLSC-VGQVVANPPTETRIYLDNITRILDRLLDGYDNRLRPGFGGPVTEVKTDIFVTSFGPVSDVEMEYTMDVFFRQTWIDDRLKFEGPIEI 99
E1BE96|GABRA6_Bos_taurus             MVSPLPCLYIVLWVENALGKLEDEGNFYSKNISRILDNLLEGYDNRLRPGFGGAVTEVKTDIYVTSFGPVSDVEMEYTMDVFFRQTWTDE---------- 90
                                     1.......10........20........30........40........50........60........70........80........90.......100

                                                      ****:**:***********:*:**************:***:*:*******:*********** :**:********:*****:
Q16445|GABRA6_Homo_sapiens           LSLNNLMVSKIWTPDTFFRNGKKSIAHNMTTPNKLFRIMQNGTILYTMRLTINADCPMRLVNFPMDGHACPLKFGSYAYPKSEIIYTWKKGPLYSVEVPE 200
A0A2R8ZQ75|GABRA6_Pan_paniscus       LSLNNLMVSKIWTPDTFFRNGKKSIAHNMTTPNKLFRIMQNGTILYTMRLTINADCPMRLVNFPMDGHACPLKFGSYAYPKSEIIYTWKKGPLYSVEVPE 200
H2QRY3|GABRA6_Pan_troglodytes        LSLNNLMVSKIWTPDTFFRNGKKSIAHNMTTPNKLFRIMQNGTILYTMRLTINADCPMRLVNFPMDGHACPLKFGSYAYPKSEIIYTWKKGPLYSVEVPE 200
A0A1D5RL15|GABRA6_Macaca_mulatta     LSLNNLMVSKIWTPDTFFRNGKKSIAHNMTTPNKLFRIMQNGTILYTMRLTINADCPMRLVNFPMDGHACPLKFGSYAYPKSEIIYTWKKGPLYSVEVPE 200
M3YP65|GABRA6_Mustela_putorius_furo  LSLNNLMVSKIWTPDTFFRNGKKSIAHNMTTPNKLFRIMQNGTILYTMRLTINADCPMRLVNFPMDGHACPLKFGSYAYPKSEIIYTWKKGPLYSVEVPE 200
P30191|GABRA6_Rattus_norvegicus      LSLNNLMVSKIWTPDTFFRNGKKSIAHNMTTPNKLFRLMHNGTILYTMRLTINADCPMRLVNFPMDGHACPLKFGSYAYPKSEIIYTWKKGPLYSVEVPE 200
P16305|GABRA6_Mus_musculus           LSLNNLMVSKIWTPDTFFRNGKKSIAHNMTTPNKLFRLMQNGTILYTMRLTINADCPMRLVNFPMDGHACPLKFGSYAYPKTEIIYTWKKGPLYSVEVPE 200
E2RSN3|GABRA6_Canis_lupus_familiaris LSLNNLMVSKIWTPDTFFRNGKKSIAHNMTTPNKLFRIMQNGTILYTMRLTINADCPMRLVNFPMDGHACPLKFGSYAYPKSEIIYTWKKGPLYSVEVPE 200
F1QGW0|GABRA6b_Danio_rerio           LRLNNLMVSKIWTPDTFFRNGKRSISHNMTTPNKLFRIMQNGTILYTMRLTINAECPMRLINFPMDGHSCPLKFGSYAYPMSEIVYTWKKGPLFSVEVPQ 199
E1BE96|GABRA6_Bos_taurus             ------------------RNGKKSIAHNMTTPNKLFRIMQNGTILYTMRLTINADCPMKLVNFPMDGHACPLKFGSYAYPKSEIIYTWKKGPLYSVEVPE 172
                                     .......110.......120.......130.......140.......150.......160.......170.......180.......190.......200

                                     ****************** :*******::************:*:** * ********:******:************************************
Q16445|GABRA6_Homo_sapiens           ESSSLLQYDLIGQTVSSETIKSNTGEYVIMTVYFHLQRKMGYFMIQIYTPCIMTVILSQVSFWINKESVPARTVFGITTVLTMTTLSISARHSLPKVSYA 300
A0A2R8ZQ75|GABRA6_Pan_paniscus       ESSSLLQYDLIGQTVSSETIKSNTGEYVIMTVYFHLQRKMGYFMIQIYTPCIMTVILSQVSFWINKESVPARTVFGITTVLTMTTLSISARHSLPKVSYA 300
H2QRY3|GABRA6_Pan_troglodytes        ESSSLLQYDLIGQTVSSETIKSNTGEYVIMTVYFHLQRKMGYFMIQIYTPCIMTVILSQVSFWINKESVPARTVFGITTVLTMTTLSISARHSLPKVSYA 300
A0A1D5RL15|GABRA6_Macaca_mulatta     ESSSLLQYDLIGQTVSSETIKSNTGEYVIMTVYFHLQRKMGYFMIQIYTPCIMTVILSQVSFWINKESVPARTVFGITTVLTMTTLSISARHSLPKVSYA 300
M3YP65|GABRA6_Mustela_putorius_furo  ESSSLLQYDLIGQTVSSETIKSNTGEYVIMTVYFHLQRKMGYFMIQIYTPCIMTVILSQVSFWINKESVPARTVFGITTVLTMTTLSISARHSLPKVSYA 300
P30191|GABRA6_Rattus_norvegicus      ESSSLLQYDLIGQTVSSETIKSNTGEYVIMTVYFHLQRKMGYFMIQIYTPCIMTVILSQVSFWINKESVPARTVFGITTVLTMTTLSISARHSLPKVSYA 300
P16305|GABRA6_Mus_musculus           ESSSLLQYDLIGQTVSSETIKSNTGEYVIMTVYFHLQRKMGYFMIQIYTPCIMTVILSQVSFWINKESVPARTVFGITTVLTMTTLSISARHSLPKVSYA 300
E2RSN3|GABRA6_Canis_lupus_familiaris ESSSLLQYDLIGQTVSSETIKSNTGEYVIMTVYFHLQRKMGYFMIQIYTPCIMTVILSQVSFWINKESVPARTVFGITTVLTMTTLSISARHSLPKVSYA 300
F1QGW0|GABRA6b_Danio_rerio           ESSSLLQYDLIGQTVSSERLKSNTGEYIVMTVYFHLQRKMGFFLIQTYIPCIMTVILAQVSFWIDKESVPARTVFGITTVLTMTTLSISARHSLPKVSYA 299
E1BE96|GABRA6_Bos_taurus             ESSSLLQYDLIGQTVSSETIKSNTGEYVIMTVYFHLQRKMGYFMIQIYTPCIMTVILSQVSFWINKESVPARTVFGITTVLTMTTLSISARHSLPKVSYA 272
                                     .......210.......220.......230.......240.......250.......260.......270.......280.......290.......300

                                     **************************:.**::*  *:*: *   ..: :   :   .* *::. *.   ****:.**:      .:.:  .  ::: :  .
Q16445|GABRA6_Homo_sapiens           TAMDWFIAVCFAFVFSALIEFAAVNYFTNLQTQKAKRKAQFAAPPTVTISKATEPLEAEIVLHP-DSKYHLKKRITSLSLPIVSSSEANKVLTRAPILQS 399
A0A2R8ZQ75|GABRA6_Pan_paniscus       TAMDWFIAVCFAFVFSALIEFAAVNYFTNLQTQKAKRKAQIAAPPTVTISKATEPLEAEIVLHP-DSKYHLKKRITSLSLPIVSSSEANKVLTRAPILQS 399
H2QRY3|GABRA6_Pan_troglodytes        TAMDWFIAVCFAFVFSALIEFAAVNYFTNLQTQKAKRKAQIAAPPSVTISKATEPLEAEIVLHP-DSKYHLKKRITSLSLPIVSSSEANKVLTRAPILQS 399
A0A1D5RL15|GABRA6_Macaca_mulatta     TAMDWFIAVCFAFVFSALIEFAAVNYFTNLQTQKAKRKAQIAAPPTVTISKATEPLEAEIVLHP-DSKYHLKKRITSLSLPIVSSSEASKVLTRAPILQS 399
M3YP65|GABRA6_Mustela_putorius_furo  TAMDWFIAVCFAFVFSALIEFAAVNYFTNLQTQKAKRRAQIAASPPVTISKAREPLEAEIVLHP-DSKYHLKKRITSLTLPIVPSPEASKVLTRAPILQS 399
P30191|GABRA6_Rattus_norvegicus      TAMDWFIAVCFAFVFSALIEFAAVNYFTNLQSQKAERQAQTAAKPPVAKSKTTESLEAEIVVHS-DSKYHLKKRISSLTLPIVPSSEASKVLSRTPILPS 399
P16305|GABRA6_Mus_musculus           TAMDWFIAVCFAFVFSALIEFAAVNYFTNLQSQKAERQAQTAATPPVAKSKASESLQAEIVVHS-DSKYHLKKRISSLTLPIVPSSEASKALSRTPILKS 399
E2RSN3|GABRA6_Canis_lupus_familiaris TAMDWFIAVCFAFVFSALIEFAAVNYFTNLQTQKAKRRAQNAASPPVTISKATEPLEAEIVLHP-DSKYHLKKRITSLTLPIVPSPEASKVLTRAPILQS 399
F1QGW0|GABRA6b_Danio_rerio           TAMDWFIAVCFAFVFSALIEFAAVNYFSTLQANKELRRANIARAAAAAAAARSD---GEGVLNPLDPGGVLKKRMNSLSQR--SATDRFSRPSQSDFHQQ 394
E1BE96|GABRA6_Bos_taurus             TAMDWFIAVCFAFVFSALIEFAAVNYFTNLQTQKAKRKAQIAASPPVTIAKATEPLEAEIVLHP-DSKYHLKKRITSLTLPIVPSPEASKVLTRAPILQS 371
                                     .......310.......320.......330.......340.......350.......360.......370.......380.......390.......400

                                      .. *.        *** **:*******::*..**:***:***:*****   .
Q16445|GABRA6_Homo_sapiens           TPVTPPPLSPAFGGTSKIDQYSRILFPVAFAGFNLVYWVVYLSKDTMEVSSSVE 453
A0A2R8ZQ75|GABRA6_Pan_paniscus       TPVTPPPLSPAFGGTSKIDQYSRILFPVAFAGFNIVYWVVYLSKDTMEVSSSVE 453
H2QRY3|GABRA6_Pan_troglodytes        TPVTPPPLSPAFGGTSKIDQYSRILFPVAFAGFNLVYWVVYLSKDTMEVSSSVE 453
A0A1D5RL15|GABRA6_Macaca_mulatta     TPVTPPPLSPAFGGTSKIDQYSRILFPVAFAGFNLVYWIVYLSKDTMEMSSSAE 453
M3YP65|GABRA6_Mustela_putorius_furo  TPVTPPPLSPAFGGTSKIDQYSRILFPVAFAGFNLVYWIVYLSKDTMEVSSSVE 453
P30191|GABRA6_Rattus_norvegicus      TPVTPPLLLPAIGGTSKIDQYSRILFPVAFAGFNLVYWIVYLSKDTMEVSSTVE 453
P16305|GABRA6_Mus_musculus           TPVSPPLLLPATGGTSKIDQYSRILFPVAFAGFNLVYWIVYLSKDTMEVSSTVE 453
E2RSN3|GABRA6_Canis_lupus_familiaris TPVTPPPLSPAFGGTSKIDQYSRILFPVAFAGFNLVYWVVYLSKDTMEVSSSVE 453
F1QGW0|GABRA6b_Danio_rerio           GSAVPA--NNMLIGTSIIDKYSRILFPLSFGAFNLVYWIVYLTKDTMESREV-- 444
E1BE96|GABRA6_Bos_taurus             TPVTPPPVSLTFGGTSKIDQYSRILFPVAFAGFNLVYWVVYLSKDTMEVSNSVE 425
                                     .......410.......420.......430.......440.......450....
```

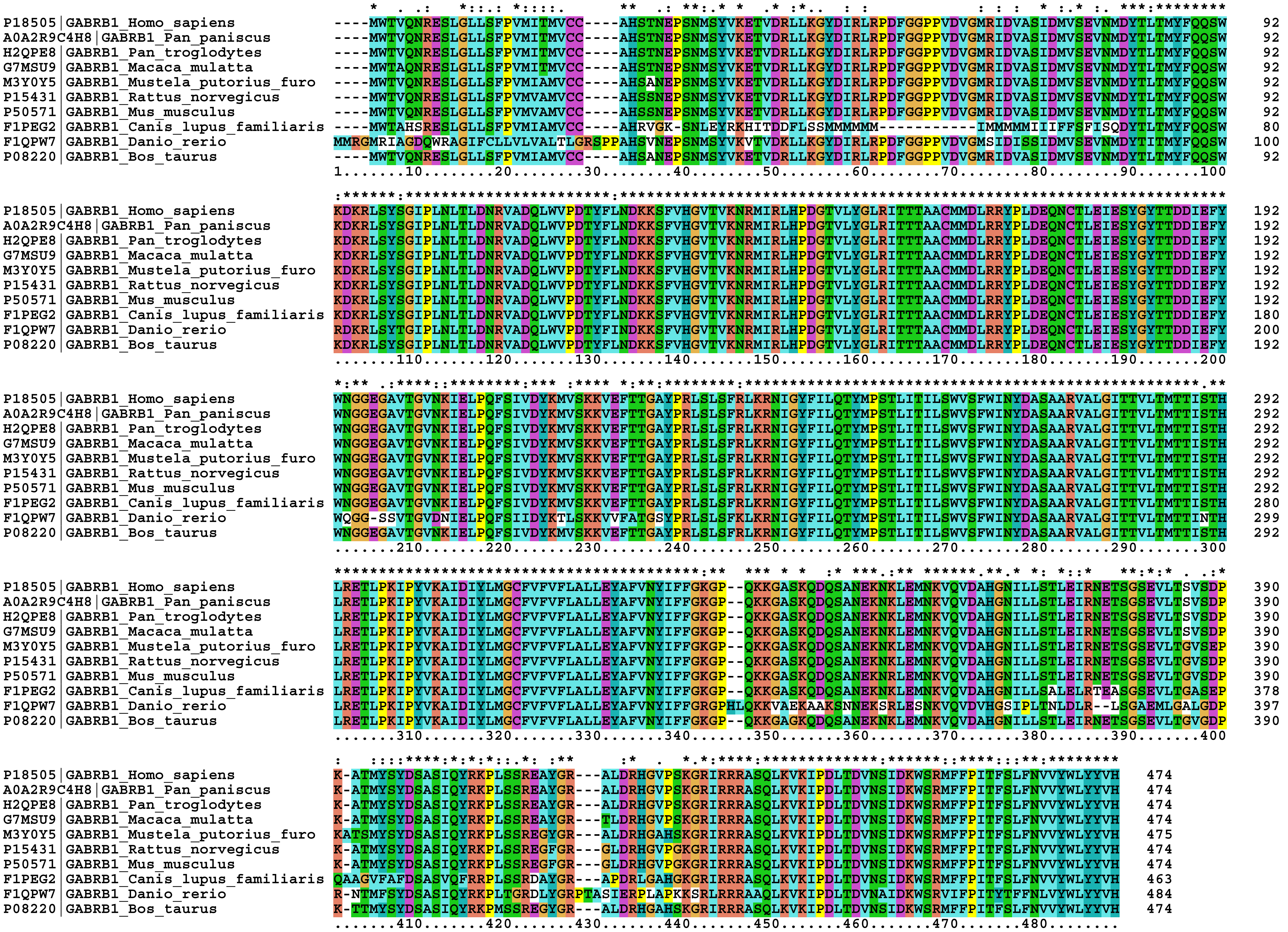
P18505|GABRB1_Homo_sapiens
A0A2R9C4H8|GABRB1_Pan_paniscus
H2QPE8|GABRB1_Pan_troglodytes
G7MSU9|GABRB1_Macaca_mulatta
M3Y0Y5|GABRB1_Mustela_putorius_furo
P15431|GABRB1_Rattus_norvegicus
P50571|GABRB1_Mus_musculus
F1PEG2|GABRB1_Canis_lupus_familiaris
F1QPW7|GABRB1_Danio_rerio
P08220|GABRB1_Bos_taurus

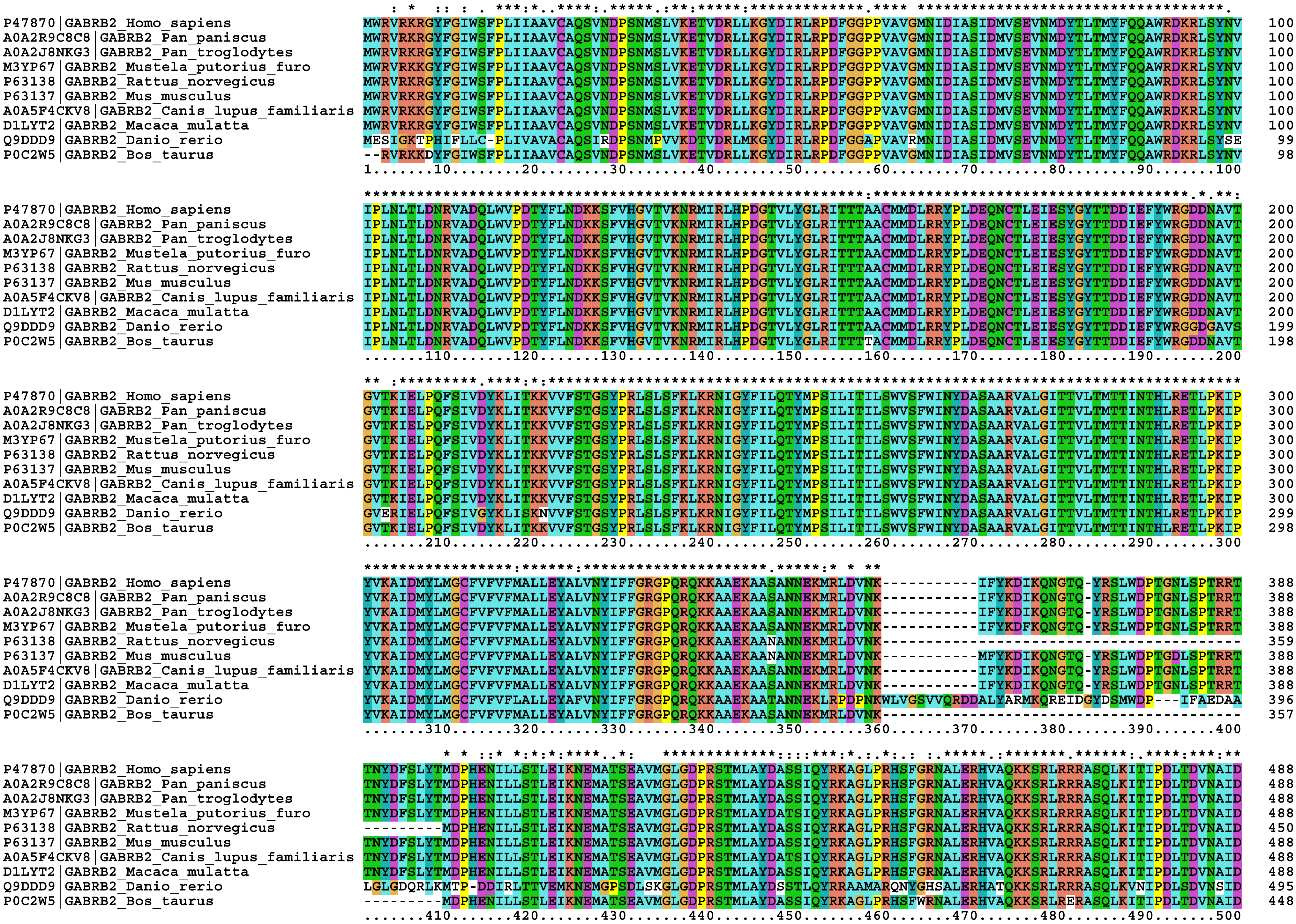

P47870|GABRB2_Homo_sapiens MWRVRKRGYFGIWSFPLIIAAVCAQSVNDPSNMSLVKETVDRLLKGYDIRLRPDFGGPPVAVGMNIDIASIDMVSEVNMDYTLTMYFQQAWRDKRLSYNV 100
A0A2R9C8C8|GABRB2_Pan_paniscus MWRVRKRGYFGIWSFPLIIAAVCAQSVNDPSNMSLVKETVDRLLKGYDIRLRPDFGGPPVAVGMNIDIASIDMVSEVNMDYTLTMYFQQAWRDKRLSYNV 100
A0A2J8NKG3|GABRB2_Pan_troglodytes MWRVRKRGYFGIWSFPLIIAAVCAQSVNDPSNMSLVKETVDRLLKGYDIRLRPDFGGPPVAVGMNIDIASIDMVSEVNMDYTLTMYFQQAWRDKRLSYNV 100
M3YP67|GABRB2_Mustela_putorius_furo MWRVRKKGYFGIWSFPLIIAAVCAQSVNDPSNMSLVKETVDRLLKGYDIRLRPDFGGPPVAVGMNIDIASIDMVSEVNMDYTLTMYFQQAWRDKRLSYNV 100
P63138|GABRB2_Rattus_norvegicus MWRVRKRGYFGIWSFPLIIAAVCAQSVNDPSNMSLVKETVDRLLKGYDIRLRPDFGGPPVAVGMNIDIASIDMVSEVNMDYTLTMYFQQAWRDKRLSYNV 100
P63137|GABRB2_Mus_musculus MWRVRKRGYFGIWSFPLIIAAVCAQSVNDPSNMSLVKETVDRLLKGYDIRLRPDFGGPPVAVGMNIDIASIDMVSEVNMDYTLTMYFQQAWRDKRLSYNV 100
A0A5F4CKV8|GABRB2_Canis_lupus_familiaris MWRVRKKGYFGIWSFPLIIAAVCAQSVNDPSNMSLVKETVDRLLKGYDIRLRPDFGGPPVAVGMNIDIASIDMVSEVNMDYTLTMYFQQAWRDKRLSYNV 100
D1LYT2|GABRB2_Macaca_mulatta MWRVRKRGYFGIWSFPLIIAAVCAQSVNDPSNMSLVKETVDRLLKGYDIRLRPDFGGPPVAVGMNIDIASIDMVSEVNMDYTLTMYFQQAWRDKRLSYNV 100
Q9DDD9|GABRB2_Danio_rerio MESIGKTPHIFLLC-PLIVAVACAQSIRDPSNMPVVKDTVDRLMKGYDIRLRPDFGGAPVAVRMNIDIASIDMVSEVNMDYTLTMYFQQAWRDKRLSYSE 99
P0C2W5|GABRB2_Bos_taurus --RVRKKDYFGIWSFPLIIAAVCAQSVNDPSNMSLVKETVDRLLKGYDIRLRPDFGGPPVAVGMNIDIASIDMVSEVNMDYTLTMYFQQAWRDKRLSYNV 98
1.......10........20........30........40........50........60........70........80........90.......100
P47870|GABRB2_Homo_sapiens IPLNLTLDNRVADQLWVPDTYFLNDKKSFVHGVTVKNRMIRLHPDGTVLYGLRITTTAACMMDLRRYPLDEQNCTLEIESYGYTTDDIEFYWRGDDNAVT 200
A0A2R9C8C8|GABRB2_Pan_paniscus IPLNLTLDNRVADQLWVPDTYFLNDKKSFVHGVTVKNRMIRLHPDGTVLYGLRITTTAACMMDLRRYPLDEQNCTLEIESYGYTTDDIEFYWRGDDNAVT 200
A0A2J8NKG3|GABRB2_Pan_troglodytes IPLNLTLDNRVADQLWVPDTYFLNDKKSFVHGVTVKNRMIRLHPDGTVLYGLRITTTAACMMDLRRYPLDEQNCTLEIESYGYTTDDIEFYWRGDDNAVT 200
M3YP67|GABRB2_Mustela_putorius_furo IPLNLTLDNRVADQLWVPDTYFLNDKKSFVHGVTVKNRMIRLHPDGTVLYGLRITTTAACMMDLRRYPLDEQNCTLEIESYGYTTDDIEFYWRGDDNAVT 200
P63138|GABRB2_Rattus_norvegicus IPLNLTLDNRVADQLWVPDTYFLNDKKSFVHGVTVKNRMIRLHPDGTVLYGLRITTTAACMMDLRRYPLDEQNCTLEIESYGYTTDDIEFYWRGDDNAVT 200
P63137|GABRB2_Mus_musculus IPLNLTLDNRVADQLWVPDTYFLNDKKSFVHGVTVKNRMIRLHPDGTVLYGLRITTTAACMMDLRRYPLDEQNCTLEIESYGYTTDDIEFYWRGDDNAVT 200
A0A5F4CKV8|GABRB2_Canis_lupus_familiaris IPLNLTLDNRVADQLWVPDTYFLNDKKSFVHGVTVKNRMIRLHPDGTVLYGLRITTTAACMMDLRRYPLDEQNCTLEIESYGYTTDDIEFYWRGDDNAVT 200
D1LYT2|GABRB2_Macaca_mulatta IPLNLTLDNRVADQLWVPDTYFLNDKKSFVHGVTVKNRMIRLHPDGTVLYGLRITTTAACMMDLRRYPLDEQNCTLEIESYGYTTDDIEFYWRGDDNAVT 200
Q9DDD9|GABRB2_Danio_rerio IPLNLTLDNRVADQLWVPDTYFLNDKKSFVHGVTVKNRMIRLHPDGTVLYGLRITTTAACMMDLRRYPLDEQNCTLEIESYGYTTDDIEFYWRGGDGAVS 199
P0C2W5|GABRB2_Bos_taurus IPLNLTLDNRVADQLWVPDTYFLNDKKSFVHGVTVKNRMIRLHPDGTVLYGLRITTTTACMMDLRRYPLDEQNCTLEIESYGYTTDDIEFYWRGDDNAVT 198
.......110.......120.......130.......140.......150.......160.......170.......180.......190.......200
P47870|GABRB2_Homo_sapiens GVTKIELPQFSIVDYKLITKKVVFSTGSYPRLSLSFKLKRNIGYFILQTYMPSILITILSWVSFWINYDASAARVALGITTVLTMTTINTHLRETLPKIP 300
A0A2R9C8C8|GABRB2_Pan_paniscus GVTKIELPQFSIVDYKLITKKVVFSTGSYPRLSLSFKLKRNIGYFILQTYMPSILITILSWVSFWINYDASAARVALGITTVLTMTTINTHLRETLPKIP 300
A0A2J8NKG3|GABRB2_Pan_troglodytes GVTKIELPQFSIVDYKLITKKVVFSTGSYPRLSLSFKLKRNIGYFILQTYMPSILITILSWVSFWINYDASAARVALGITTVLTMTTINTHLRETLPKIP 300
M3YP67|GABRB2_Mustela_putorius_furo GVTKIELPQFSIVDYKLITKKVVFSTGSYPRLSLSFKLKRNIGYFILQTYMPSILITILSWVSFWINYDASAARVALGITTVLTMTTINTHLRETLPKIP 300
P63138|GABRB2_Rattus_norvegicus GVTKIELPQFSIVDYKLITKKVVFSTGSYPRLSLSFKLKRNIGYFILQTYMPSILITILSWVSFWINYDASAARVALGITTVLTMTTINTHLRETLPKIP 300
P63137|GABRB2_Mus_musculus GVTKIELPQFSIVDYKLITKKVVFSTGSYPRLSLSFKLKRNIGYFILQTYMPSILITILSWVSFWINYDASAARVALGITTVLTMTTINTHLRETLPKIP 300
A0A5F4CKV8|GABRB2_Canis_lupus_familiaris GVTKIELPQFSIVDYKLITKKVVFSTGSYPRLSLSFKLKRNIGYFILQTYMPSILITILSWVSFWINYDASAARVALGITTVLTMTTINTHLRETLPKIP 300
D1LYT2|GABRB2_Macaca_mulatta GVTKIELPQFSIVDYKLITKKVVFSTGSYPRLSLSFKLKRNIGYFILQTYMPSILITILSWVSFWINYDASAARVALGITTVLTMTTINTHLRETLPKIP 300
Q9DDD9|GABRB2_Danio_rerio GVERIELPQFSIVGYKLISKNVVFSTGSYPRLSLSFKLKRNIGYFILQTYMPSILITILSWVSFWINYDASAARVALGITTVLTMTTINTHLRETLPKIP 299
P0C2W5|GABRB2_Bos_taurus GVTKIELPQFSIVDYKLITKKVVFSTGSYPRLSLSFKLKRNIGYFILQTYMPSILITILSWVSFWINYDASAARVALGITTVLTMTTINTHLRETLPKIP 298
.......210.......220.......230.......240.......250.......260.......270.......280.......290.......300
P47870|GABRB2_Homo_sapiens YVKAIDMYLMGCFVFVFMALLEYALVNYIFFGRGPQRQKKAAEKAASANNEKMRLDVNK-----------IFYKDIKQNGTQ-YRSLWDPTGNLSPTRRT 388
A0A2R9C8C8|GABRB2_Pan_paniscus YVKAIDMYLMGCFVFVFMALLEYALVNYIFFGRGPQRQKKAAEKAASANNEKMRLDVNK-----------IFYKDIKQNGTQ-YRSLWDPTGNLSPTRRT 388
A0A2J8NKG3|GABRB2_Pan_troglodytes YVKAIDMYLMGCFVFVFMALLEYALVNYIFFGRGPQRQKKAAEKAASANNEKMRLDVNK-----------IFYKDIKQNGTQ-YRSLWDPTGNLSPTRRT 388
M3YP67|GABRB2_Mustela_putorius_furo YVKAIDMYLMGCFVFVFMALLEYALVNYIFFGRGPQRQKKAAEKAASANNEKMRLDVNK-----------IFYKDFKQNGTQ-YRSLWDPTGNLSPTRRT 388
P63138|GABRB2_Rattus_norvegicus YVKAIDMYLMGCFVFVFMALLEYALVNYIFFGRGPQRQKKAAEKAANANNEKMRLDVNK----------------------------------------- 359
P63137|GABRB2_Mus_musculus YVKAIDMYLMGCFVFVFMALLEYALVNYIFFGRGPQRQKKAAEKAANANNEKMRLDVNK-----------MFYKDIKQNGTQ-YRSLWDPTGDLSPTRRT 388
A0A5F4CKV8|GABRB2_Canis_lupus_familiaris YVKAIDMYLMGCFVFVFMALLEYALVNYIFFGRGPQRQKKAAEKAASANNEKMRLDVNK-----------IFYKDIKQNGTQ-YRSLWDPTGNLSPTRRT 388
D1LYT2|GABRB2_Macaca_mulatta YVKAIDMYLMGCFVFVFMALLEYALVNYIFFGRGPQRQKKAAEKAASANNEKMRLDVNK-----------IFYKDIKQNGTQ-YRSLWDPTGNLSPTRRT 388
Q9DDD9|GABRB2_Danio_rerio YVKAIDMYLMGCFVFVFLALLEYAFVNYIFFGRGPQRQKKAAEKAATANNEKLRPDPNKWLVGSVVQRDDALYARMKQREIDGYDSMWDP---IFAEDAA 396
P0C2W5|GABRB2_Bos_taurus YVKAIDMYLMGCFVFVFMALLEYALVNYIFFGRGPQRQKKAAEKAASANNEKMRLDVNK----------------------------------------- 357
.......310.......320.......330.......340.......350.......360.......370.......380.......390.......400
P47870|GABRB2_Homo_sapiens TNYDFSLYTMDPHENILLSTLEIKNEMATSEAVMGLGDPRSTMLAYDASSIQYRKAGLPRHSFGRNALERHVAQKKSRLRRRASQLKITIPDLTDVNAID 488
A0A2R9C8C8|GABRB2_Pan_paniscus TNYDFSLYTMDPHENILLSTLEIKNEMATSEAVMGLGDPRSTMLAYDASSIQYRKAGLPRHSFGRNALERHVAQKKSRLRRRASQLKITIPDLTDVNAID 488
A0A2J8NKG3|GABRB2_Pan_troglodytes TNYDFSLYTMDPHENILLSTLEIKNEMATSEAVMGLGDPRSTMLAYDASSIQYRKAGLPRHSFGRNALERHVAQKKSRLRRRASQLKITIPDLTDVNAID 488
M3YP67|GABRB2_Mustela_putorius_furo TNYDFSLYTMDPHENILLSTLEIKNEMATSEAVMGLGDPRSTMLAYDATSIQYRKAGLPRHSFGRNALERHVAQKKSRLRRRASQLKITIPDLTDVNAID 488
P63138|GABRB2_Rattus_norvegicus ---------MDPHENILLSTLEIKNEMATSEAVMGLGDPRSTMLAYDASSIQYRKAGLPRHSFGRNALERHVAQKKSRLRRRASQLKITIPDLTDVNAID 450
P63137|GABRB2_Mus_musculus TNYDFSLYTMDPHENILLSTLEIKNEMATSEAVMGLGDPRSTMLAYDASSIQYRKAGLPRHSFGRNALERHVAQKKSRLRRRASQLKITIPDLTDVNAID 488
A0A5F4CKV8|GABRB2_Canis_lupus_familiaris TNYDFSLYTMDPHENILLSTLEIKNEMATSEAVMGLGDPRSTMLAYDATSIQYRKAGLPRHSFGRNALERHVAQKKSRLRRRASQLKITIPDLTDVNAID 488
D1LYT2|GABRB2_Macaca_mulatta TNYDFSLYTMDPHENILLSTLEIKNEMATSEAVMGLGDPRSTMLAYDASSIQYRKAGLPRHSFGRNALERHVAQKKSRLRRRASQLKITIPDLTDVNAID 488
Q9DDD9|GABRB2_Danio_rerio LGLGDQRLKMTP-DDIRLTTVEMKNEMGPSDLSKGLGDPRSTMLAYDSSTLQYRRAAMARQNYGHSALERHATQKKSRLRRRASQLKVNIPDLSDVNSID 495
P0C2W5|GABRB2_Bos_taurus ---------MDPHENILLSTLEIKNEMATSEAVMGLGDPRSTMLAYDASSIQYRKAGLPRHSFWRNALERHVAQKKSRLRERASQLKITIPDLTDVNAID 448
.......410.......420.......430.......440.......450.......460.......470.......480.......490.......500

```
                                          :***::**.:*************:
P47870|GABRB2_Homo_sapiens                RWSRIFFPVVFSFFNIVYWLYYVN   512
A0A2R9C8C8|GABRB2_Pan_paniscus            RWSRIFFPVVFSFFNIVYWLYYVN   512
A0A2J8NKG3|GABRB2_Pan_troglodytes         RWSRIFFPVVFSFFNIVYWLYYVN   512
M3YP67|GABRB2_Mustela_putorius_furo       RWSRIFFPVVFSFFNIVYWLYYVN   512
P63138|GABRB2_Rattus_norvegicus           RWSRIFFPVVFSFFNIVYWLYYVN   474
P63137|GABRB2_Mus_musculus                RWSRIFFPVVFSFFNIVYWLYYVN   512
A0A5F4CKV8|GABRB2_Canis_lupus_familiaris  RWSRIFFPVVFSFFNIVYWLYYVN   512
D1LYT2|GABRB2_Macaca_mulatta              RWSRIFFPVVFSFFNIVYWLYYVN   512
Q9DDD9|GABRB2_Danio_rerio                 KWSRMIFPTLFSFFNIVYWLYYVH   519
P0C2W5|GABRB2_Bos_taurus                  RWSRIFFPVVFSFFNIVYWLYYVN   472
                                          .......510.......520....
```

```
                                                                                                  .*                   *******************:
P28472|GABRB3_Homo_sapiens              MWGLAGGRLFGIFSAPVLVAVVCCAQSVNDPGNMSFVKETVDKLLKGYDIRLRPDFGGPPVCVGMNIDIASIDMVSEVNMDYTLTMYFQQYWRDKRLAYS  100
A0A2R9BWE5|GABRB3_Pan_paniscus          ----------------------------------------------------------SP-------------------QDYTLTMYFQQYWRDKRLAYS   23
A0A2I3THQ1|GABRB3_Pan_troglodytes       MWGLAGGRLFGIFSAPVLVAVVCCAQSVNDPGNMSFVKETVDKLLKGYDIRLRPDFGGPPVCVGMNIDIASIDMVSEVNMDYTLTMYFQQYWRDKRLAYS  100
F6ZKJ4|GABRB3_Macaca_mulatta            MCSGLLALLLPIWLSWTLGTRGSEPRSVNDPGNMSFVKETVDKLLKGYDIRLRPDFGGPPVCVGMNIDIASIDMVSEVNMDYTLTMYFQQYWRDKRLAYS  100
M3XNI6|GABRB3_Mustela_putorius_furo     MWGFAGGRLFGIFSAPVLVAVVCCAQSVNDPGNMSFVKETVDKLLKGYDIRLRPDFGGPPVCVGMNIDIASIDMVSEVNMDYTLTMYFQQYWRDKRLAYS  100
P63079|GABRB3_Rattus_norvegicus         MWGFAGGRLFGIFSAPVLVAVVCCAQSVNDPGNMSFVKETVDKLLKGYDIRLRPDFGGPPVCVGMNIDIASIDMVSEVNMDYTLTMYFQQYWRDKRLAYS  100
P63080|GABRB3_Mus_musculus              MWGFAGGRLFGIFSAPVLVAVVCCAQSVNDPGNMSFVKETVDKLLKGYDIRLRPDFGGPPVCVGMNIDIASIDMVSEVNMDYTLTMYFQQYWRDKRLAYS  100
J9P3X1|GABRB3_Canis_lupus_familiaris    ----------------------------------------------------------PPVCVGMNIDIASIDMVSEVNMDYTLTMYFQQYWRDKRLAYS   42
A0A0R4ILP2|GABRB3_Danio_rerio           MFGIQKRRLFGVLSLPVIVAVMCCAQSANEPGNMSFVKETVDKLLKGYDIRLRPDFGGAPVAVGMSIDVASIDMVSEVNMDYTLTMYFQQYWRDKRLAYT  100
A5D7U6|GABRB3_Bos_taurus                MWGFAGGRFFGIFSAPVLVAVVCCAQSVNDPGNMSFVKETVDKLLKGYDIRLRPDFGGPPVCVGMNIDIASIDMVSEVNMDYTLTMYFQQYWRDKRLAYS  100
                                        1.......10........20........30........40........50........60........70........80........90.......100

                                        *************************************************************************************************:**:.**
P28472|GABRB3_Homo_sapiens              GIPLNLTLDNRVADQLWVPDTYFLNDKKSFVHGVTVKNRMIRLHPDGTVLYGLRITTTAACMMDLRRYPLDEQNCTLEIESYGYTTDDIEFYWRGGDKAV  200
A0A2R9BWE5|GABRB3_Pan_paniscus          GIPLNLTLDNRVADQLWVPDTYFLNDKKSFVHGVTVKNRMIRLHPDGTVLYGLRITTTAACMMDLRRYPLDEQNCTLEIESYGYTTDDIEFYWRGGDKAV  123
A0A2I3THQ1|GABRB3_Pan_troglodytes       GIPLNLTLDNRVADQLWVPDTYFLNDKKSFVHGVTVKNRMIRLHPDGTVLYGLRITTTAACMMDLRRYPLDEQNCTLEIESYGYTTDDIEFYWRGGDKAV  200
F6ZKJ4|GABRB3_Macaca_mulatta            GIPLNLTLDNRVADQLWVPDTYFLNDKKSFVHGVTVKNRMIRLHPDGTVLYGLRITTTAACMMDLRRYPLDEQNCTLEIESYGYTTDDIEFYWRGGDKAV  200
M3XNI6|GABRB3_Mustela_putorius_furo     GIPLNLTLDNRVADQLWVPDTYFLNDKKSFVHGVTVKNRMIRLHPDGTVLYGLRITTTAACMMDLRRYPLDEQNCTLEIESYGYTTDDIEFYWRGGDKAV  200
P63079|GABRB3_Rattus_norvegicus         GIPLNLTLDNRVADQLWVPDTYFLNDKKSFVHGVTVKNRMIRLHPDGTVLYGLRITTTAACMMDLRRYPLDEQNCTLEIESYGYTTDDIEFYWRGGDKAV  200
P63080|GABRB3_Mus_musculus              GIPLNLTLDNRVADQLWVPDTYFLNDKKSFVHGVTVKNRMIRLHPDGTVLYGLRITTTAACMMDLRRYPLDEQNCTLEIESYGYTTDDIEFYWRGGDKAV  200
J9P3X1|GABRB3_Canis_lupus_familiaris    GIPLNLTLDNRVADQLWVPDTYFLNDKKSFVHGVTVKNRMIRLHPDGTVLYGLRITTTAACMMDLRRYPLDEQNCTLEIESYGYTTDDIEFYWRGGDKAV  142
A0A0R4ILP2|GABRB3_Danio_rerio           GIPLNLTLDNRVADQLWVPDTYFLNDKKSFVHGVTVKNRMIRLHPDGTVLYGLRITTTAACMMDLRRYPLDEQNCTLEIESYGYTTDDIEFYWKGGETAV  200
A5D7U6|GABRB3_Bos_taurus                GIPLNLTLDNRVADQLWVPDTYFLNDKKSFVHGVTVKNRMIRLHPDGTVLYGLRITTTAACMMDLRRYPLDEQNCTLEIESYGYTTDDIEFYWRGGDKAV  200
                                        .......110.......120.......130.......140.......150.......160.......170.......180.......190.......200

                                        ***.**********:::********:***********:**************************************************************
P28472|GABRB3_Homo_sapiens              TGVERIELPQFSIVEHRLVSRNVVFATGAYPRLSLSFRLKRNIGYFILQTYMPSILITILSWVSFWINYDASAARVALGITTVLTMTTINTHLRETLPKI  300
A0A2R9BWE5|GABRB3_Pan_paniscus          TGVERIELPQFSIVEHRLVSRNVVFATGAYPRLSLSFRLKRNIGYFILQTYMPSILITILSWVSFWINYDASAARVALGITTVLTMTTINTHLRETLPKI  223
A0A2I3THQ1|GABRB3_Pan_troglodytes       TGVERIELPQFSIVEHRLVSRNVVFATGAYPRLSLSFRLKRNIGYFILQTYMPSILITILSWVSFWINYDASAARVALGITTVLTMTTINTHLRETLPKI  300
F6ZKJ4|GABRB3_Macaca_mulatta            TGVERIELPQFSIVEHRLVSRNVVFATGAYPRLSLSFRLKRNIGYFILQTYMPSILITILSWVSFWINYDASAARVALGITTVLTMTTINTHLRETLPKI  300
M3XNI6|GABRB3_Mustela_putorius_furo     TGVERIELPQFSIVEHRLVSRNVVFATGAYPRLSLSFRLKRNIGYFILQTYMPSILITILSWVSFWINYDASAARVALGITTVLTMTTINTHLRETLPKI  300
P63079|GABRB3_Rattus_norvegicus         TGVERIELPQFSIVEHRLVSRNVVFATGAYPRLSLSFRLKRNIGYFILQTYMPSILITILSWVSFWINYDASAARVALGITTVLTMTTINTHLRETLPKI  300
P63080|GABRB3_Mus_musculus              TGVERIELPQFSIVEHRLVSRNVVFATGAYPRLSLSFRLKRNIGYFILQTYMPSILITILSWVSFWINYDASAARVALGITTVLTMTTINTHLRETLPKI  300
J9P3X1|GABRB3_Canis_lupus_familiaris    TGVERIELPQFSIVEHRLVSRNVVFATGAYPRLSLSFRLKRNIGYFILQTYMPSILITILSWVSFWINYDASAARVALGITTVLTMTTINTHLRETLPKI  242
A0A0R4ILP2|GABRB3_Danio_rerio           TGVSRIELPQFSIVDYKLVSRNVVFSTGAYPRLSLSFKLKRNIGYFILQTYMPSILITILSWVSFWINYDASAARVALGITTVLTMTTINTHLRETLPKI  300
A5D7U6|GABRB3_Bos_taurus                TGVERIELPQFSIVEHRLVSRNVVFATGAYPRLSLSFRLKRNIGYFILQTYMPSILITILSWVSFWINYDASAARVALGITTVLTMTTINTHLRETLPKI  300
                                        .......210.......220.......230.......240.......250.......260.......270.......280.......290.......300

                                        ************************************* *******  **:***.* : *:                           **::*.***:.::
P28472|GABRB3_Homo_sapiens              PYVKAIDMYLMGCFVFVFLALLEYAFVNYIFFGRGPQRQKKLAEKTAKAKNDRSKSESNR---------------------------VDAHGNILLTSLE  373
A0A2R9BWE5|GABRB3_Pan_paniscus          PYVKAIDMYLMGCFVFVFLALLEYAFVNYIFFGRGPQRQKKLAEKTAKAKNDRSKSESNR---------------------------VDAHGNILLTSLE  296
A0A2I3THQ1|GABRB3_Pan_troglodytes       PYVKAIDMYLMGCFVFVFLALLEYAFVNYIFFGRGPQRQKKLAEKTAKAKNDRSKSESNR---------------------------VDAHGNILLTSLE  373
F6ZKJ4|GABRB3_Macaca_mulatta            PYVKAIDMYLMGCFVFVFLALLEYAFVNYIFFGRGPQRQKKLAEKTAKAKNDRSKSESNR---------------------------VDAHGNILLTSLE  373
M3XNI6|GABRB3_Mustela_putorius_furo     PYVKAIDMYLMGCFVFVFLALLEYAFVNYIFFGRGPQRQKKLAEKTAKAKNDRSKSESNR---------------------------VDAHGSILLTSLE  373
P63079|GABRB3_Rattus_norvegicus         PYVKAIDMYLMGCFVFVFLALLEYAFVNYIFFGRGPQRQKKLAEKTAKAKNDRSKSEINR---------------------------VDAHGNILLAPMD  373
P63080|GABRB3_Mus_musculus              PYVKAIDMYLMGCFVFVFLALLEYAFVNYIFFGRGPQRQKKLAEKTAKAKNDRSKSEINR---------------------------VDAHGNILLAPMD  373
J9P3X1|GABRB3_Canis_lupus_familiaris    PYVKAIDMYLMGCFVFVFLALLEYAFVNYIFFGRGPQRQKKLAEKTAKAKNDRSKSESNR---------------------------VDAHGNILLTSLE  315
A0A0R4ILP2|GABRB3_Danio_rerio           PYVKAIDMYLMGCFVFVFLALLEYAFVNYIFFGRGPQMQKKLAEKAEKANNDRNKYDGNKSVQEGGNCKPSSVKQSNQVQGHRHSRGVDSQGNILLTTLE  400
A5D7U6|GABRB3_Bos_taurus                PYVKAIDMYLMGCFVFVFLALLEYAFVNYIFFGRGPQRQKKLAEKNAKAKNDRSKGDSNR---------------------------VDAHGNILLTSLE  373
                                        .......310.......320.......330.......340.......350.......360.......370.......380.......390.......400

                                        :***:  **::  .:.::*** :  ************      .*.  **.   **.:***************************:** .******:****
P28472|GABRB3_Homo_sapiens              VHNEM--NEVSGGIGDTRNS-AISFDNSGIQYRKQSMPREGHGRFLGDRSLPHKKTHLRRRSSQLKIKIPDLTDVNAIDRWSRIVFPFTFSLFNLVYWLY  470
A0A2R9BWE5|GABRB3_Pan_paniscus          VHNEM--NEVAGGIGDTRNS-AISFDNSGIQYRKQSMPREGHGRFLGDRSLPHKKTHLRRRSSQLKIKIPDLTDVNAIDRWSRIVFPFTFSLFNLVYWLY  393
A0A2I3THQ1|GABRB3_Pan_troglodytes       VHNEM--NEVAGGIGDTRNS-AISFDNSGIQYRKQSMPREGHGRFLGDRSLPHKKTHLRRRSSQLKIKIPDLTDVNAIDRWSRIVFPFTFSLFNLVYWLY  470
F6ZKJ4|GABRB3_Macaca_mulatta            VHNEM--NEVAGGVGDTRNS-AISFDNSGIQYRKQSMPREGHGRFLGDRSLPHKKTHLRRRSSQLKIKIPDLTDVNAIDRWSRIVFPFTFSLFNLVYWLY  470
M3XNI6|GABRB3_Mustela_putorius_furo     VHNEM--NEVTGGVGDTRNS-ARPFDNSGIQYRKQSMPREGHGRHMGDRSIPHKKTHLRRRSSQLKIKIPDLTDVNAIDRWSRIVFPFTFSLFNLVYWLY  470
P63079|GABRB3_Rattus_norvegicus         VHNEM--NEVAGSVGDTRNS-AISFDNSGIQYRKQSMPKEGHGRYMGDRSIPHKKTHLRRRSSQLKIKIPDLTDVNAIDRWSRIVFPFTFSLFNLVYWLY  470
P63080|GABRB3_Mus_musculus              VHNEM--NEVAGSVGDTRNS-AISFDNSGIQYRKQSMPKEGHGRYMGDRSIPHKKTHLRRRSSQLKIKIPDLTDVNAIDRWSRIVFPFTFSLFNLVYWLY  470
J9P3X1|GABRB3_Canis_lupus_familiaris    VHNEM--NEVTGSVGDTRNS-ARPFDNSGIQYRKQSMPREGHGRHMGDRSIPHKKTHLRRRSSQLKIKIPDLTDVNAIDRWSRIVFPFTFSLFNLVYWLY  412
A0A0R4ILP2|GABRB3_Danio_rerio           IHNEVAGNEITTSVSEARNSTSMVFDNSGIQYRKQST-----ARHSMDRNAQSKKPRLRRRSSQLKIKIPDLTDVNAIDRWSRIIFPAVFSLFNLIYWLY  495
A5D7U6|GABRB3_Bos_taurus                VHNEM--NEVAGGVGDTRNS-ARSFDNSGIQYRKQSMPREGHGRHM-DRSVPHKKTHLRRRSSQLKIKIPDLTDVNAIDRWSRIVFPFTFSLFNLVYWLY  469
                                        .......410.......420.......430.......440.......450.......460.......470.......480.......490.......500
```

```
                                              ***
P28472|GABRB3_Homo_sapiens                    YVN   473
A0A2R9BWE5|GABRB3_Pan_paniscus                YVN   396
A0A2I3THQ1|GABRB3_Pan_troglodytes             YVN   473
F6ZKJ4|GABRB3_Macaca_mulatta                  YVN   473
M3XNI6|GABRB3_Mustela_putorius_furo           YVN   473
P63079|GABRB3_Rattus_norvegicus               YVN   473
P63080|GABRB3_Mus_musculus                    YVN   473
J9P3X1|GABRB3_Canis_lupus_familiaris          YVN   415
A0A0R4ILP2|GABRB3_Danio_rerio                 YVN   498
A5D7U6|GABRB3_Bos_taurus                      YVN   472
                                              ...
```

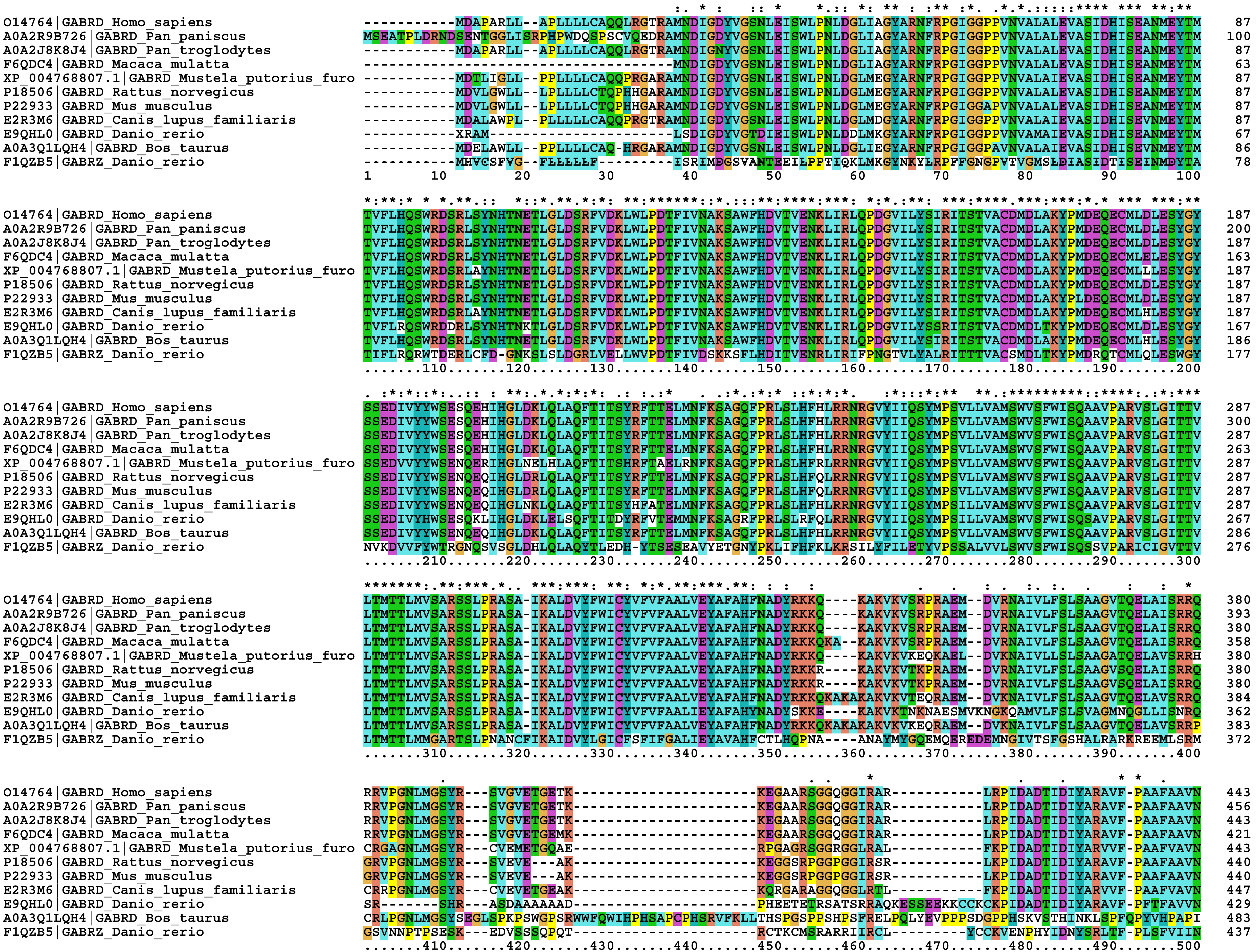
O14764|GABRD_Homo_sapiens
A0A2R9B726|GABRD_Pan_paniscus
A0A2J8K8J4|GABRD_Pan_troglodytes
F6QDC4|GABRD_Macaca_mulatta
XP_004768807.1|GABRD_Mustela_putorius_furo
P18506|GABRD_Rattus_norvegicus
P22933|GABRD_Mus_musculus
E2R3M6|GABRD_Canis_lupus_familiaris
E9QHL0|GABRD_Danio_rerio
A0A3Q1LQH4|GABRD_Bos_taurus
F1QZB5|GABRZ_Danio_rerio

```
                                                   *
O14764|GABRD_Homo_sapiens                        VIYWAAYAM-------------------   452
A0A2R9B726|GABRD_Pan_paniscus                    VIYWAAYAM-------------------   465
A0A2J8K8J4|GABRD_Pan_troglodytes                 VIYWAAYAM-------------------   452
F6QDC4|GABRD_Macaca_mulatta                      VIYWAAYAM-------------------   430
XP_004768807.1|GABRD_Mustela_putorius_furo       VLYWAAYAM-------------------   452
P18506|GABRD_Rattus_norvegicus                   IIYWAAYTM-------------------   449
P22933|GABRD_Mus_musculus                        IIYWAAYTM-------------------   449
E2R3M6|GABRD_Canis_lupus_familiaris              VLYWAAYAM-------------------   456
E9QHL0|GABRD_Danio_rerio                         VIYWVAYTM-------------------   438
A0A3Q1LQH4|GABRD_Bos_taurus                      PTSWRATPTRPSMGKPGVVGSPRPCSGH   511
F1QZB5|GABRZ_Danio_rerio                         LLYWTYYLYF------------------   447
                                                 .......510.......520........
```

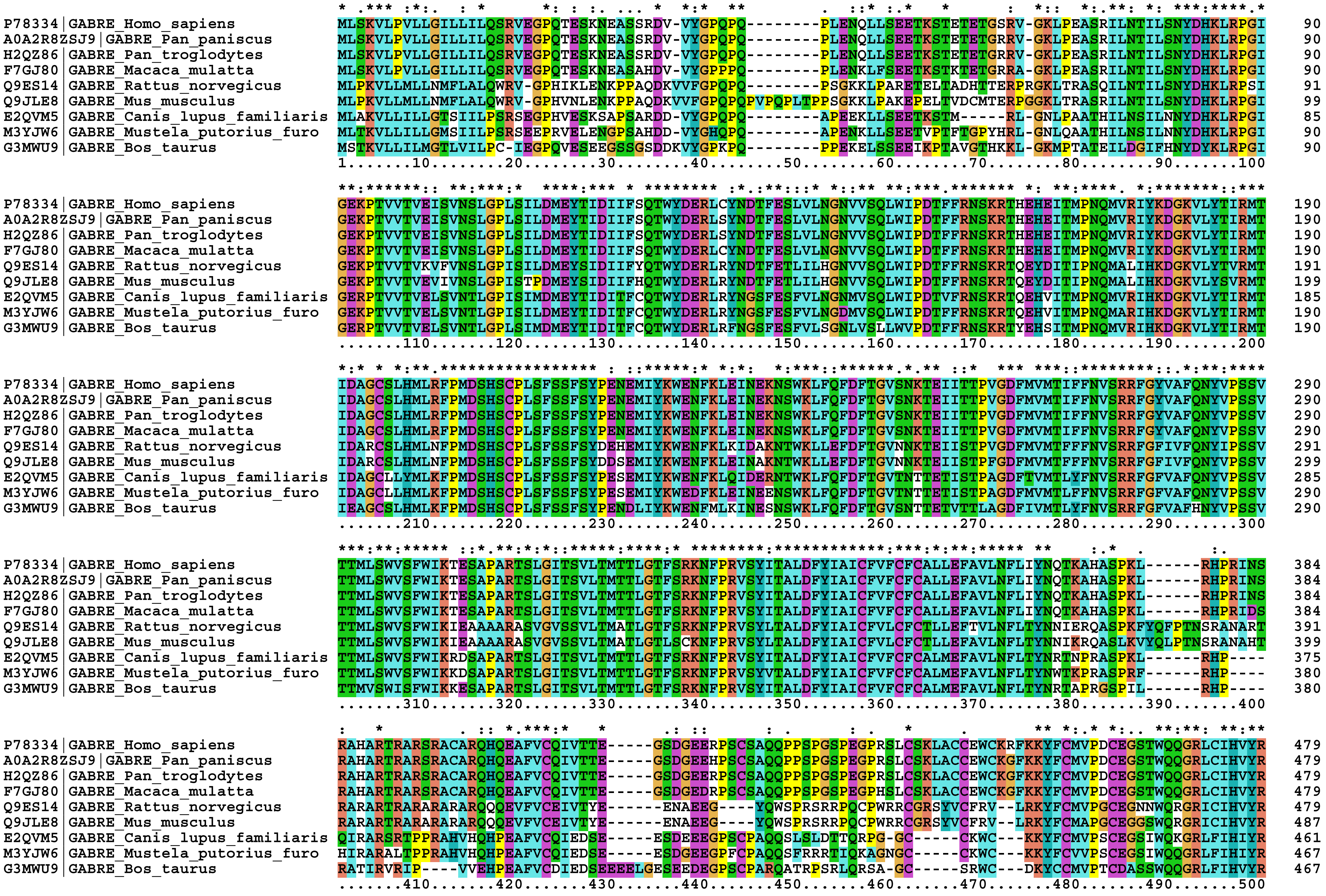

P78334|GABRE_Homo_sapiens MLSKVLPVLLGILLILQSRVEGPQTESKNEASSRDV-VYGPQPQ--------PLENQLLSEETKSTETETGSRV-GKLPEASRILNTILSNYDHKLRPGI 90
A0A2R8ZSJ9|GABRE_Pan_paniscus MLSKVLPVLLGILLILQSRVEGPQTESKNEASSRDV-VYGPQPQ--------PLENQLLSEETKSTETETGRRV-GKLPEASRILNTILSNYDHKLRPGI 90
H2QZ86|GABRE_Pan_troglodytes MLSKVLPVLLGILLILQSRVEGPQTESKNEASSRDV-VYGPQPQ--------PLENQLLSEETKSTETETGRRV-GKLPEASRILNTILSNYDHKLRPGI 90
F7GJ80|GABRE_Macaca_mulatta MLSKVLPVLLGILLILQSRVEGPQTESKNEASAHDV-VYGPPPQ--------PLENKLFSEETKSTKTETGRRA-GKLPEASRILNTILSNYDHKLRPGI 90
Q9ES14|GABRE_Rattus_norvegicus MLPKVLLMLLNMFLALQWRV-GPHIKLENKPPAQDKVVFGPQPQ--------PSGKKLPARETELTADHTTERPRGKLTRASQILNTILSNYDHKLRPSI 91
Q9JLE8|GABRE_Mus_musculus MLPKVLLMLLNMFLALQWRV-GPHVNLENKPPAQDKVVFGPQPQPVPQPLTPPSGKKLPAKEPELTVDCMTERPGGKLTRASRILNTILSNYDHKLRPGI 99
E2QVM5|GABRE_Canis_lupus_familiaris MLAKVLLILLGTSIILPSRSEGPHVESKSAPSARDD-VYGPQPQ--------APEEKLLSEETKSTM-----RL-GNLPAATHILNSILNNYDHKLRPGI 85
M3YJW6|GABRE_Mustela_putorius_furo MLTKVLLILLGMSIILPSRSEEPRVELENGPSAHDD-VYGHQPQ--------APENKLLSEETVPTFTGPYHRL-GNLQAATHILNSILNNYDHKLRPGI 90
G3MWU9|GABRE_Bos_taurus MSTKVLLILMGTLVILPC-IEGPQVESEEGSSGSDDKVYGPKPQ--------PPEKELSSEEIKPTAVGTHKKL-GKMPTATEILDGIFHNYDYKLRPGI 90

P78334|GABRE_Homo_sapiens GEKPTVVTVEISVNSLGPLSILDMEYTIDIIFSQTWYDERLCYNDTFESLVLNGNVVSQLWIPDTFFRNSKRTHEHEITMPNQMVRIYKDGKVLYTIRMT 190
A0A2R8ZSJ9|GABRE_Pan_paniscus GEKPTVVTVEISVNSLGPLSILDMEYTIDIIFSQTWYDERLSYNDTFESLVLNGNVVSQLWIPDTFFRNSKRTHEHEITMPNQMVRIYKDGKVLYTIRMT 190
H2QZ86|GABRE_Pan_troglodytes GEKPTVVTVEISVNSLGPLSILDMEYTIDIIFSQTWYDERLSYNDTFESLVLNGNVVSQLWIPDTFFRNSKRTHEHEITMPNQMVRIYKDGKVLYTIRMT 190
F7GJ80|GABRE_Macaca_mulatta GEKPTVVTVEISVNSLGPLSILDMEYTIDIIFSQTWYDERLCYNDTFESLVLNGNVVSQLWIPDTFFRNSKRTHEHEITMPNQMVRIYKDGKVLYTIRMT 190
Q9ES14|GABRE_Rattus_norvegicus GEKPTVVTVKVFVNSLGPISILDMEYSIDIIFYQTWYDERLRYNDTFETLILHGNVVSQLWIPDTFFRNSKRTQEYDITIPNQMALIHKDGKVLYTVRMT 191
Q9JLE8|GABRE_Mus_musculus GEKPTVVTVEVIVNSLGPISTPDMEYSIDIIFHQTWYDERLRYNDTFETLILHGNVVSQLWIPDTFFRNSKRTQEYDITIPNQMALIHKDGKVLYSVRMT 199
E2QVM5|GABRE_Canis_lupus_familiaris GERPTVVTVELSVNTLGPISIMDMEYTIDITFCQTWYDERLRYNGSFESFVLNGNMVSQLWIPDTVFRNSKRTQEHVITMPNQMVRIHKDGKVLYTIRMT 185
M3YJW6|GABRE_Mustela_putorius_furo GEKPTVVTVELSVNTLGPISILDMEYTIDITFCQTWYDERLRYNGSFESFVLNGDMVSQLWIPDTFFRNSKRTQEHVITMPNQMARIHKDGKVLYTIRMT 190
G3MWU9|GABRE_Bos_taurus GERPTVVTVELSVNTLGPLSIMDMEYTIDITFCQTWYDERLRFNGSFESFVLSGNLVSLLWVPDTFFRNSKRTYEHSITMPNQMVRIHKDGKVLYTIRMT 190

P78334|GABRE_Homo_sapiens IDAGCSLHMLRFPMDSHSCPLSFSSFSYPENEMIYKWENFKLEINEKNSWKLFQFDFTGVSNKTEIITTPVGDFMVMTIFFNVSRRFGYVAFQNYVPSSV 290
A0A2R8ZSJ9|GABRE_Pan_paniscus IDAGCSLHMLRFPMDSHSCPLSFSSFSYPENEMIYKWENFKLEINEKNSWKLFQFDFTGVSNKTEIITTPVGDFMVMTIFFNVSRRFGYVAFQNYVPSSV 290
H2QZ86|GABRE_Pan_troglodytes IDAGCSLHMLRFPMDSHSCPLSFSSFSYPENEMIYKWENFKLEINEKNSWKLFQFDFTGVSNKTEIITTPVGDFMVMTIFFNVSRRFGYVAFQNYVPSSV 290
F7GJ80|GABRE_Macaca_mulatta IDAGCSLHMLRFPMDSHSCPLSFSSFSYPENEMIYKWENFKLEINEKNSWKLFQFDFTGVSNKTEIITTPVGDFMVMTIFFNVSRRFGYVAFQNYVPSSV 290
Q9ES14|GABRE_Rattus_norvegicus IDARCSLHMLNFPMDSHSCPLSFSSFSYDEHEMIYKWENFKLKIDAKNTWKLLEFDFTGVNNKTEIISTPVGDFMVMTFFNVSRRFGFIVFQNYIPSSV 291
Q9JLE8|GABRE_Mus_musculus IDARCSLHMLNFPMDSHSCPLSFSSFSYDDSEMIYKWENFKLEINAKNTWKLLEFDFTGVNNKTEIISTPFGDFMVMTFFFNVSRRFGFIVFQNYVPSSV 299
E2QVM5|GABRE_Canis_lupus_familiaris IDAGCLLYMLKFPMDSHSCPLSFSSFSYPESEMIYKWENFKLQIDERNTWKLFQFDFTGVTNTTETISTPAGDFTVMTLYFNVSRRFGFVAFQNYVPSSV 285
M3YJW6|GABRE_Mustela_putorius_furo IDAGCLLHMLKFPMDSHSCPLSFSSFSYPESEMIYKWEDFKLEINEENSWKLFQFDFTGVTNTTETISTPAGDFMVMTLFFNVSRRFGFVAFQNYVPSSV 290
G3MWU9|GABRE_Bos_taurus IEAGCSLHMLKFPMDSHSCPLSFSSFSYPENDLIYKWENFMLKINESNSWKLFQFDFTGVSNTTETVTTLAGDFIVMTLYFNVSRRFGFVAFHNYVPSSV 290

P78334|GABRE_Homo_sapiens TTMLSWVSFWIKTESAPARTSLGITSVLTMTTLGTFSRKNFPRVSYITALDFYIAICFVFCFCALLEFAVLNFLIYNQTKAHASPKL------RHPRINS 384
A0A2R8ZSJ9|GABRE_Pan_paniscus TTMLSWVSFWIKTESAPARTSLGITSVLTMTTLGTFSRKNFPRVSYITALDFYIAICFVFCFCALLEFAVLNFLIYNQTKAHASPKL------RHPRINS 384
H2QZ86|GABRE_Pan_troglodytes TTMLSWVSFWIKTESAPARTSLGITSVLTMTTLGTFSRKNFPRVSYITALDFYIAICFVFCFCALLEFAVLNFLIYNQTKAHASPKL------RHPRINS 384
F7GJ80|GABRE_Macaca_mulatta TTMLSWVSFWIKTESAPARTSLGITSVLTMTTLGTFSRKNFPRVSYITALDFYIAICFVFCFCALLEFAVLNFLIYNQTKAHASPKL------RHPRIDS 384
Q9ES14|GABRE_Rattus_norvegicus TTMLSWVSFWIKIEAAAARASVGVSSVLTMATLGTFSRKNFPRVSYLTALDFYIAICFVLCFCTLLEFTVLNFLTYNNIERQASPKFYQFPTNSRANART 391
Q9JLE8|GABRE_Mus_musculus TTMLSWVSFWIKIEAAAARASVGVSSVLTMATLGTLSCKNFPRVSYLTALDFYIAICFVLCFCTLLEFAVLNFLTYNNIKRQASLKVYQLPTNSRANAHT 399
E2QVM5|GABRE_Canis_lupus_familiaris TTMLSWVSFWIKRDSAPARTSLGITSVLTMTTLGTFSRKNFPRVSYITALDFYIAICFVFCFCALMEFAVLNFLTYNRTNPRASPKL------RHP---- 375
M3YJW6|GABRE_Mustela_putorius_furo TTMLSWVSFWIKKDSAPARTSLGITSVLTMTTLGTFSRKNFPRVSYVTALDFYIAICFVFCFCALMEFAVLNFLTYNWTKPRASPRF------RHP---- 380
G3MWU9|GABRE_Bos_taurus TTMVSWISFWIKKESAPARTSLGITSVLTMTTLGTFSRKNFPRVSYITALDFYIAICFVFCFCALMEFAVLNFLTYNRTAPRGSPIL------RHP---- 380

P78334|GABRE_Homo_sapiens RAHARTRARSRACARQHQEAFVCQIVTTE-----GSDGEERPSCSAQQPPSPGSPEGPRSLCSKLACCEWCKRFKKYFCMVPDCEGSTWQQGRLCIHVYR 479
A0A2R8ZSJ9|GABRE_Pan_paniscus RAHARTRARSRACARQHQEAFVCQIVTTE-----GSDGEEHPSCSAQQPPSPGSPEGPRSLCSKLACCEWCKGFKKYFCMVPDCEGSTWQQGRLCIHVYR 479
H2QZ86|GABRE_Pan_troglodytes RAHARTRARSRACARQHQEAFVCQIVTTE-----GSDGEERPSCSAQQPPSPGSPEGPRSLCSKLACCEWCKGFKKYFCMVPDCEGSTWQQGRLCIHVYR 479
F7GJ80|GABRE_Macaca_mulatta RAHARTRARSRACARQHQEAFVCQIVTTE-----GSDGEDRPSCSAQQPPSPGSPEGPHSLCSKLACCEWCKGFKKYFCMVPDCEGSTWQQGRLCIHVYR 479
Q9ES14|GABRE_Rattus_norvegicus RARARTRARARARARQQQEVFVCEIVTYE------ENAEEG----YQWSPRSRRPQCPWRRCGRSYVCFRV--LRKYFCMVPGCEGNNWQRGRICIHVYR 479
Q9JLE8|GABRE_Mus_musculus RARARTRARARARARQQQEVFVCEIVTYE------ENAEEG----YQWSPRSRRPQCPWRRCGRSYVCFRV--LRKYFCMAPGCEGGSWQRGRICIHVYR 487
E2QVM5|GABRE_Canis_lupus_familiaris QIRARSRTPPRAHVHQHPEAFVCQIEDSE-----ESDEEEGPSCPAQQSLSLDTTQRPG-GC-----CKWC---KKYFCMVPSCEGSIWQKGRLFIHIYR 461
M3YJW6|GABRE_Mustela_putorius_furo HIRARALTPPRAHVHQHPEAFVCQIEDSE-----ESDEEEGPFCPAQQSFRRRTIQKAGNGC-----CKWC---KKYFCVVPSCEGSIWQQGRLFIHIYR 467
G3MWU9|GABRE_Bos_taurus RATIRVRIP----VVEHPEAFVCDIEDSEEEELGESEEDEGPSCPARQATRPSRLQRSA-GC-----SRWC---DKYCCMVPTCDASSWQQGRLFIHVYR 467

```
                                              *******:**::******:**::****
P78334|GABRE_Homo_sapiens                     LDNYSRVVFPVTFFFFNVLYWLVCLNL   506
A0A2R8ZSJ9|GABRE_Pan_paniscus                 LDNYSRVVFPVSFFFFNVLYWLVCLNL   506
H2QZ86|GABRE_Pan_troglodytes                  LDNYSRVVFPVTFFFFNVLYWLVCLNL   506
F7GJ80|GABRE_Macaca_mulatta                   LDNYSRVVFPVTFFFFNVLYWLVCLNL   506
Q9ES14|GABRE_Rattus_norvegicus                LDNYSRVLFPITFFFFNVVYWVICLNL   506
Q9JLE8|GABRE_Mus_musculus                     LDNYSRVLFPITFFFFNVLYWLICLNL   514
E2QVM5|GABRE_Canis_lupus_familiaris           LDNYSRVIFPVTFFFFNVLYWLVCLNL   488
M3YJW6|GABRE_Mustela_putorius_furo            LDNYSRVVFPVTFFFFNVLYWLVCLNL   494
G3MWU9|GABRE_Bos_taurus                       LDNYSRVIFPVTFFFFNVIYWLVCLNL   494
                                              .......510.......520.......
```

```
                                      .. :    :*    .:     :: ** .  : :* .  *.::** **:.:******:*:::*.*:* ***.********************** **
Q8N1C3|GABRG1_Homo_sapiens            ---MGPLKAFLFSPFLLRSQSRGVRLVFLLLT--LHLGNCVDKADDEDDEDLTVNKTWVLAPKIHEGDITQILNSLLQGYDNKLRPDIGVRPTVIETDVY   95
A0A2R9A1L5|GABRG1_Pan_paniscus        ---MGPLKAFLFSPFLLRSQSRGVRLVFLLLT--LHLGNCVDKADDEDDEDLTVNKTWVLAPKIHEGDITQILNSLLQGYDNKLRPDIGVRPTVIETDVY   95
H2QPE4|GABRG1_Pan_troglodytes         ---MGPLKAFLFSPFLLRSQSRGVRLVFLLLT--LHLGNCVDKADDEDDEDLTVNKTWVLAPKIHEGDITQILNSLLQGYDNKLRPDIGVRPTVIETDVY   95
A0A5F8A0N7|GABRG1_Macaca_mulatta      ---MGPLKAFLFSPFLLRSQSRGVRLVFLLLT--LHLGNCVDKADDEDDEDLTVNKTWVLAPKIHEGDITQILNSLLQGYDNKLRPDIGVRPTVIETDVY   95
M3Y0V1|GABRG1_Mustela_putorius_furo   ---MGSWKAFLFSPFLLWSQSRVVRLMFLLLT--LHLGNCVDKVDDEDDEDLTVNKTWVLAPRIHEGDITQILNSLLQGYDNKLRPDIGVRPTVIETDVY   95
P23574|GABRG1_Rattus_norvegicus       ---MGSGKVFLFSPSLLWSQTRGVRLIFLLLT--LHLGNCIDKADDEDDEDLTMNKTWVLAPKIHEGDITQILNSLLQGYDNKLRPDIGVRPTVIETDVY   95
Q9R0Y8|GABRG1_Mus_musculus            ---MGSGKAFLFSPSLLWSQTRGVRLIFLLLT--LHLGNCVDKADDEDDEDLTMNKTWVLAPKIHEGDITQILNSLLQGYDNKLRPDIGVRPTVIETDVY   95
E2RH22|GABRG1_Canis_lupus_familiaris  MPPMGPWKAPLCSPFLLGSQSRVVRLLFLLLT--LHLGNCVDKVDDEDDEDLTVNKTWVLAPKIHEGDITQILNSLLQGYDNKLRPDIGVRPTVIETDVY   98
A0A0R4IPF9|GABRG1_Danio_rerio         YMLNSPLQE---AP----SHAYGYFVLPLLNNQIMAFGPS--KAEEEDYEDVPINKTWVLSPKVYESDVTLILNKLLQGYDNKLRPDIGVRPTVIETAVY   91
F6Q4V7|GABRG1_Bos_taurus              ---MGSWKALLFSPFLLWKQRRGVRLMFLLLT--VHLGNCVDKADDEDDEDLTVNKTWVLAPKIHEGDITQILNSLLQGYDNKLRPDIGVRPTVIETDVY   95
                                      1.......10.......20.......30.......40.......50.......60.......70.......80.......90.......100

                                      ******************:*****:********:**:*************************************:*.:***:*************:****
Q8N1C3|GABRG1_Homo_sapiens            VNSIGPVDPINMEYTIDIIFAQTWFDSRLKFNSTMKVLMLNSNMVGKIWIPDTFFRNSRKSDAHWITTPNRLLRIWNDGRVLYTLRLTINAECYLQLHNF  195
A0A2R9A1L5|GABRG1_Pan_paniscus        VNSIGPVDPINMEYTIDIIFAQTWFDSRLKFNSTMKVLMLNSNMVGKIWIPDTFFRNSRKSDAHWITTPNRLLRIWNDGRVLYTLRLTINAECYLQLHNF  195
H2QPE4|GABRG1_Pan_troglodytes         VNSIGPVDPINMEYTIDIIFAQTWFDSRLKFNSTMKVLMLNSNMVGKIWIPDTFFRNSRKSDAHWITTPNRLLRIWNDGRVLYTLRLTINAECYLQLHNF  195
A0A5F8A0N7|GABRG1_Macaca_mulatta      VNSIGPVDPINMEYTIDIIFAQTWFDSRLKFNSTMKVLMLNSNMVGKIWIPDTFFRNSRKSDAHWITTPNRLLRIWNDGRVLYTLRLTINAECYLQLHNF  195
M3Y0V1|GABRG1_Mustela_putorius_furo   VNSIGPVDPINMEYTIDIIFAQTWFDSRLKFNSTMKVLMLNSNMVGKIWIPDTFFRNSRKSDAHWITTPNRLLRIWNDGRVLYTLRLTINAECYLQLHNF  195
P23574|GABRG1_Rattus_norvegicus       VNSIGPVDPINMEYTIDIIFAQTWFDSRLKFNSTMKVLMLNSNMVGKIWIPDTFFRNSRKSDAHWITTPNRLLRIWSDGRVLYTLRLTINAECYLQLHNF  195
Q9R0Y8|GABRG1_Mus_musculus            VNSIGPVDPINMEYTIDIIFAQTWFDSRLKFNSTMKVLMLNSNMVGKIWIPDTFFRNSRKSDAHWITTPNRLLRIWSDGRVLYTLRLTINAECYLQLHNF  195
E2RH22|GABRG1_Canis_lupus_familiaris  VNSIGPVDPINMEYTIDIIFAQTWFDSRLKFNSTMKVLMLNSNMVGKIWIPDTFFRNSRKSDAHWITTPNRLLRIWNDGRVLYTLRLTINAECYLQLHNF  198
A0A0R4IPF9|GABRG1_Danio_rerio         VNSIGPVDPINMEYTIDIFFAQTWYDSRLKFNSSMKLLMLNSNMVGKIWIPDTFFRNSRKSDAHWITTPNRLLRLWSNGRVMYTLRLTINAECYLKLHNF  191
F6Q4V7|GABRG1_Bos_taurus              VNSIGPVDPINMEYTIDIIFAQTWFDSRLKFNSTMKVLMLNSNMVGKIWIPDTFFRNSRKSDAHWITTPNRLLRIWNDGRVLYTLRLTINAECYLQLHNF  195
                                      .......110.......120.......130.......140.......150.......160.......170.......180.......190.......200

                                      *********************:*:*:: ****** :**************::::::* **:*::********************** : ***********
Q8N1C3|GABRG1_Homo_sapiens            PMDEHSCPLEFSSYGYPKNEIEYKWKKPSVEVADPKYWRLYQFAFVGLRNSTEITHTISGDYVIMTIFFDLSRRMGYFTIQTYIPCILTVVLSWVSFWIN  295
A0A2R9A1L5|GABRG1_Pan_paniscus        PMDEHSCPLEFSSYGYPKNEIEYKWKKPSVEVADPKYWRLYQFAFVGLRNSTEITHTISGDYVIMTIFFDLSRRMGYFTIQTYIPCILTVVLSWVSFWIN  295
H2QPE4|GABRG1_Pan_troglodytes         PMDEHSCPLEFSSYGYPKNEIEYKWKKPSVEVADPKYWRLYQFAFVGLRNSTEITHTISGDYVIMTIFFDLSRRMGYFTIQTYIPCILTVVLSWVSFWIN  295
A0A5F8A0N7|GABRG1_Macaca_mulatta      PMDEHSCPLEFSSYGYPKNEIEYKWKKPSVEVADPKYWRLYQFAFVGLRNSTEISHTISGDYVIMTIFFDLSRRMGYFTIQTYIPCILTVVLSWVSFWIN  295
M3Y0V1|GABRG1_Mustela_putorius_furo   PMDEHSCPLEFSSYGYPKNEIEYKWKKPSVEVADPKYWRLYQFAFVGLRNSTEISHTISGDYVIMTIFFDLSRRMGYFTIQTYIPCILTVVLSWVSFWIN  295
P23574|GABRG1_Rattus_norvegicus       PMDEHSCPLEFSSYGYPKNEIEYKWKKPSVEVADPKYWRLYQFAFVGLRNSTEISHTISGDYIIMTIFFDLSRRMGYFTIQTYIPCILTVVLSWVSFWIN  295
Q9R0Y8|GABRG1_Mus_musculus            PMDEHSCPLEFSSYGYPKNEIEYKWKKPSVEVADPKYWRLYQFAFVGLRNSTEISHTISGDYIIMTIFFDLSRRMGYFTIQTYIPCILTVVLSWVSFWIN  295
E2RH22|GABRG1_Canis_lupus_familiaris  PMDEHSCPLEFSSYGYPKNEIEYKWKKPSVEVADPKYWRLYQFAFVGLRNSTEISHTISGDYIIMTIFFDLSRRMGYFTIQTYIPCILTVVLSWVSFWIN  298
A0A0R4IPF9|GABRG1_Danio_rerio         PMDEHSCPLEFSSYGYPKNEIQYRWQRRSVEVADQRYWRLYQFAFVGLRNSSDVANTQSGEYVVMTIFFDLSRRMGYFTIQTYIPCSMIVVLSWVSFWIN  291
F6Q4V7|GABRG1_Bos_taurus              PMDEHSCPLEFSSYGYPKNEIEYKWKKPSVEVADPKYWRLYQFAFVGLRNTTEISHTISGDYIIMTIFFDLSRRMGYFTIQTYIPCILTVVLSWVSFWIN  295
                                      .......210.......220.......230.......240.......250.......260.......270.......280.......290.......300

                                      ************************:***********************.****************.: *. *:  : :  :  .  ::.*::*: **.::
Q8N1C3|GABRG1_Homo_sapiens            KDAVPARTSLGITTVLTMTTLSTIARKSLPKVSYVTAMDLFVSVCFIFVFAALMEYGTLHYFTSNQKGKTATKDRKLKNKASMTPGLHPGSTLIPMNNIS  395
A0A2R9A1L5|GABRG1_Pan_paniscus        KDAVPARTSLGITTVLTMTTLSTIARKSLPKVSYVTAMDLFVSVCFIFVFAALMEYGTLHYFTSNQKGKTATKDRKLKNKASMTPGLHPGSTLIPMNNIS  395
H2QPE4|GABRG1_Pan_troglodytes         KDAVPARTSLGITTVLTMTTLSTIARKSLPKVSYVTAMDLFVSVCFIFVFAALMEYGTLHYFTSNQKGKTATKDRKLKNKASMTPGLHPGSTLIPMNNIS  395
A0A5F8A0N7|GABRG1_Macaca_mulatta      KDAVPARTSLGITTVLTMTTLSTIARKSLPKVSYVTAMDLFVSVCFIFVFAALMEYGTLHYFTSNKKGKTATKDRKLKNKASMTPGLHPGSTLIPMNSVS  395
M3Y0V1|GABRG1_Mustela_putorius_furo   KDAVPARTSLGITTVLTMTTLSTIARKSLPKVSYVTAMDLFVSVCFIFVFAALMEYGTLHYFTSNKKVKT-TRDKKQKNKSSMSPGLHPGSTLIPMNNIS  394
P23574|GABRG1_Rattus_norvegicus       KDAVPARTSLGITTVLTMTTLSTIARKSLPKVSYVTAMDLFVSVCFIFVFAALMEYGTLHYFTSNNKGKT-TRDRKLKSKTSVSPGLHAGSTLIPMNNIS  394
Q9R0Y8|GABRG1_Mus_musculus            KDAVPARTSLGITTVLTMTTLSTIARKSLPKVSYVTAMDLFVSVCFIFVFAALMEYGTLHYFTSNNKGKT-TRGRKLKNKTSASPGLHAGSTLIPMNSIS  394
E2RH22|GABRG1_Canis_lupus_familiaris  KDAVPARTSLGITTVLTMTTLSTIARKSLPKVSYVTAMDLFVSVCFIFVFAALMEYGTLHYFTSNKKGKT-TRNRKLKNKSSMSPGLHPGSTLIPMNSIS  397
A0A0R4IPF9|GABRG1_Danio_rerio         KDAVPARTSLGITTVLTMTTLSTISRKSLPKVSYVTAMDLFVSVCFIFTFAALMEYGTLHYFTSNRQNKK-TKSSHAQKPSMVN--IRPGTSLLQMNNIA  388
F6Q4V7|GABRG1_Bos_taurus              KDAVPARTSLGITTVLTMTTLSTIARKSLPKVSYVTAMDLFVSVCFIFVFAALMEYGTLHYFTSNKKVKT-TREKKPKLKTSMSSGLHPGSTLIPMNSLS  394
                                      .......310.......320.......330.......340.......350.......360.......370.......380.......390.......400

                                        :  ::**.*:**:****::*****:***:*:***.*:*::::*********     * :  ::.*. :    :*:
Q8N1C3|GABRG1_Homo_sapiens            VPQ-EDDYGYQCLEGKDCASFFCCFEDCRTGSWREGRIHIRIAKIDSYSRIFF----PTAFALFNLVYWVGYLYL---------   465
A0A2R9A1L5|GABRG1_Pan_paniscus        VPQ-EDDYGYQCLEGKDCASFFCCFEDCRTGSWREGRIHIRIAKIDSYSRIFF----PTAFALFNLVYWVGYLYL---------   465
H2QPE4|GABRG1_Pan_troglodytes         VPQ-EDDYGYQCLEGKDCASFFCCFEDCRTGSWREGRIHIRIAKIDSYSRIFF----PTAFALFNLVYWVGYLYL---------   465
A0A5F8A0N7|GABRG1_Macaca_mulatta      VPQ-EEDYGYQCLEGKDCASFFCCFEDCRTGSWREGRIHIRIAKIDSYSRIFSQQLLPCSTWFIGLAIFTYKIYFISKSQKKFD   478
M3Y0V1|GABRG1_Mustela_putorius_furo   LPHGEEDYGYQCLEGKDCASFFCCFEDCRTGSWREGRIHIRIAKIDSYSRIFF----PTAFALFNLVYWVGYLYL---------   465
P23574|GABRG1_Rattus_norvegicus       MPQGEDDYGYQCLEGKDCATFFCCFEDCRTGSWREGRIHIRIAKIDSYSRIFF----PTAFALFNLVYWVGYLYL---------   465
Q9R0Y8|GABRG1_Mus_musculus            LPQGEDDYGYQCLEGKDCTSFFCCFDDCRTGSWREGRIHIRIAKIDSYSRIFF----PTAFALFNLVYWVGYLYL---------   465
E2RH22|GABRG1_Canis_lupus_familiaris  LPHGEEDYGYQCLEGKDCASFFCCFEDCRTGSWREGRIHIRIAKIDSYSRIFF----PTAFALFNLVYWVGYLYL---------   468
A0A0R4IPF9|GABRG1_Danio_rerio         PYHEDDDYAYECLDGKDCTSFFCCFDDCRSGAWRENRMHVHVSKIDSYSRIFF----PTAFGLFNLVYWIGYLYL---------   459
F6Q4V7|GABRG1_Bos_taurus              LPQGEDDYGYQCLEGKDCASFFCCFEDCRTGSWREGRIHIRIAKIDSYSRIFF----PTAFALFNLVYWVGYLYL---------   465
                                      .......410.......420.......430.......440.......450.......460.......470.......480....
```

```
                                   *:* :  *   .  : *..: *: :* :*:  *  . .  :***   * .:********** *.*** ***.**:**************:*****:
P18507-2|GABRG2_Homo_sapiens               ---MSSPNIWSTGSSVYSTPVFSQKMTVWILLLLSLYPGFTSQKSDDDYEDYASNKTWVLTPKVPEGDVTVILNNLLEGYDNKLRPDIGVKPTLIHTDMY  97
A0A2R9CM76|GABRG2_Pan_paniscus             ---MSSPNIWSTGSSVYSTPVFSQKMTVWILLLLSLYPGFTSQKSDDDYEDYASNKTWVLTPKVPEGDVTVILNNLLEGYDNKLRPDIGVKPTLIHTDMY  97
A0A2I3RPH6|GABRG2_Pan_troglodytes          ---MSSPNIWSTGSSVYSTPVFSQKMTVWILLLLSLYPGFTSQKSDDDYEDYASNKTWVLTPKVPEGDVTVILNNLLEGYDNKLRPDIGVKPTLIHTDMY  97
F7A3C2|GABRG2_Macaca_mulatta               ---MSSPNIWSTGSSVYSTPVFSQKMTVWILLLLSLYPGFTSQKSDDDYEDYASNKTWVLTPKVPEGDVTVILNNLLEGYDNKLRPDIGVKPTLIHTDMY  97
XP_004737727.1|GABRG2_Mustela_putorius_furo ---MSSTNIWSTGSSDYSTPVFSQKMTVWILLLLSLYPGLTSQKSDDDYEDYASNKTWVLTPKVPEGDVTVILNNLLEGYDNKLRPDIGVKPTLIHTDMY  97
NP_001380704.1|GABRG2_Rattus_norvegicus    ---MSSPNTWSTGSTVYS-PVFSQKMTLWILLLLSLYPGFTSQKSDDDYEDYASNKTWVLTPKVPEGDVTVILNNLLEGYDNKLRPDIGVKPTLIHTDMY  96
P22723|GABRG2_Mus_musculus                 ---MSSPNTWSIGSSVYS-PVFSQKMTLWILLLLSLYPGFTSQKSDDDYEDYASNKTWVLTPKVPEGDVTVILNNLLEGYDNKLRPDIGVKPTLIHTDMY  96
F1RDP2|GABRG2_Danio_rerio                  MVMMASLHFSSKCLNAMAIPAVTLKLFLWAFLIAHLPLSSVQLESDD---DEVTNKTWVLTPKVYESDVTHILNSLLDGYDNKLRPDIGVKPTVIHTDMF  97
E2RSQ1|GABRG2_Canis_lupus_familiaris       ---MSSTNIWSTGSSEYSTPVFSQKMTVWILLLLSLYPGLTSQKSDDDYEDYASNKTWVLTPKVPEGDVTVILNNLLEGYDNKLRPDIGVKPTLIHTDMY  97
P22300|GABRG2_Bos_taurus                   ---MSSPNIWSTGSSVYSTPVFSQKMTLWILLLLSLYPGLTRQKSDDDYEDYASNKTWVLTPKVPEGDVTVILNNLLEGYDNKLRPDIGVKPTLIHTDMY  97
                                           1.......10........20........30........40........50........60........70........80........90.......100

                                           *******************.*************:********************************************:*************:*:**
P18507-2|GABRG2_Homo_sapiens               VNSIGPVNAINMEYTIDIFFAQTWYDRRLKFNSTIKVLRLNSNMVGKIWIPDTFFRNSKKADAHWITTPNRMLRIWNDGRVLYTLRLTIDAECQLQLHNF  197
A0A2R9CM76|GABRG2_Pan_paniscus             VNSIGPVNAINMEYTIDIFFAQTWYDRRLKFNSTIKVLRLNSNMVGKIWIPDTFFRNSKKADAHWITTPNRMLRIWNDGRVLYTLRLTIDAECQLQLHNF  197
A0A2I3RPH6|GABRG2_Pan_troglodytes          VNSIGPVNAINMEYTIDIFFAQTWYDRRLKFNSTIKVLRLNSNMVGKIWIPDTFFRNSKKADAHWITTPNRMLRIWNDGRVLYTLRLTIDAECQLQLHNF  197
F7A3C2|GABRG2_Macaca_mulatta               VNSIGPVNAINMEYTIDIFFAQTWYDRRLKFNSTIKVLRLNSNMVGKIWIPDTFFRNSKKADAHWITTPNRMLRIWNDGRVLYTLRLTIDAECQLQLHNF  197
XP_004737727.1|GABRG2_Mustela_putorius_furo VNSIGPVNAINMEYTIDIFFAQTWYDRRLKFNSTIKVLRLNSNMVGKIWIPDTFFRNSKKADAHWITTPNRMLRIWNDGRVLYTLRLTIDAECQLQLHNF  197
NP_001380704.1|GABRG2_Rattus_norvegicus    VNSIGPVNAINMEYTIDIFFAQTWYDRRLKFNSTIKVLRLNSNMVGKIWIPDTFFRNSKKADAHWITTPNRMLRIWNDGRVLYTLRLTIDAECQLQLHNF  196
P22723|GABRG2_Mus_musculus                 VNSIGPVNAINMEYTIDIFFAQTWYDRRLKFNSTIKVLRLNSNMVGKIWIPDTFFRNSKKADAHWITTPNRMLRIWNDGRVLYTLRLTIDAECQLQLHNF  196
F1RDP2|GABRG2_Danio_rerio                  VNSIGPVNAINMEYTIDIFFAQTWYDRRLKFNSTMKVLRLNSNMVGKIWIPDTFFRNSKKADAHWITTPNRMLRIWNDGRILYTLRLTIDAECQLKLNNF  197
E2RSQ1|GABRG2_Canis_lupus_familiaris       VNSIGPVNAINMEYTIDIFFAQTWYDRRLKFNSTIKVLRLNSNMVGKIWIPDTFFRNSKKADAHWITTPNRMLRIWNDGRVLYTLRLTIDAECQLQLHNF  197
P22300|GABRG2_Bos_taurus                   VNSIGPVNAINMEYTIDIFFGQTWYDRRLKFNSTIKVLRLNSNMVGKIWIPDTFFRNSKKADAHWITTPNRMLRIWNDGRVLYTLRLTIDAECQLQLHNF  197
                                           .......110.......120.......130.......140.......150.......160.......170.......180.......190.......200

                                           ****************:*****:********.* ***************:***:*.******::*:*******************************
P18507-2|GABRG2_Homo_sapiens               PMDEHSCPLEFSSYGYPREEIVYQWKRSSVEVGDTRSWRLYQFSFVGLRNTTEVVKTTSGDYVVMSVYFDLSRRMGYFTIQTYIPCTLIVVLSWVSFWIN  297
A0A2R9CM76|GABRG2_Pan_paniscus             PMDEHSCPLEFSSYGYPREEIVYQWKRSSVEVGDTRSWRLYQFSFVGLRNTTEVVKTTSGDYVVMSVYFDLSRRMGYFTIQTYIPCTLIVVLSWVSFWIN  297
A0A2I3RPH6|GABRG2_Pan_troglodytes          PMDEHSCPLEFSSYGYPREEIVYQWKRSSVEVGDTRSWRLYQFSFVGLRNTTEVVKTTSGDYVVMSVYFDLSRRMGYFTIQTYIPCTLIVVLSWVSFWIN  297
F7A3C2|GABRG2_Macaca_mulatta               PMDEHSCPLEFSSYGYPREEIVYQWKRSSVEVGDTRSWRLYQFSFVGLRNTTEVVKTTSGDYVVMSVYFDLSRRMGYFTIQTYIPCTLIVVLSWVSFWIN  297
XP_004737727.1|GABRG2_Mustela_putorius_furo PMDEHSCPLEFSSYGYPREEIVYQWKRSSVEVGDTRSWRLYQFSFVGLRNTTEVVKTTSGDYVVMSVYFDLSRRMGYFTIQTYIPCTLIVVLSWVSFWIN  297
NP_001380704.1|GABRG2_Rattus_norvegicus    PMDEHSCPLEFSSYGYPREEIVYQWKRSSVEVGDTRSWRLYQFSFVGLRNTTEVVKTTSGDYVVMSVYFDLSRRMGYFTIQTYIPCTLIVVLSWVSFWIN  296
P22723|GABRG2_Mus_musculus                 PMDEHSCPLEFSSYGYPREEIVYQWKRSSVEVGDTRSWRLYQFSFVGLRNTTEVVKTTSGDYVVMSVYFDLSRRMGYFTIQTYIPCTLIVVLSWVSFWIN  296
F1RDP2|GABRG2_Danio_rerio                  PMDEHSCPLEFSSYGYPKEEIVYKWKRSSVEVGDIRSWRLYQFSFVGLRNTSEVVRTVSGDYVVLTVFFDLSRRMGYFTIQTYIPCTLIVVLSWVSFWIN  297
E2RSQ1|GABRG2_Canis_lupus_familiaris       PMDEHSCPLEFSSYGYPREEIVYQWKRSSVEVGDTRSWRLYQFSFVGLRNTTEVVKTTSGDYVVMSVYFDLSRRMGYFTIQTYIPCTLIVVLSWVSFWIN  297
P22300|GABRG2_Bos_taurus                   PMDEHSCPLEFSSYGYPREEIVYQWKRSSVEVSDTRSWRLYQFSFVGLRNTTEVVKTTSGDYVVMTVYFDLSRRMGYFTIQTYIPCTLIVVLSWVSFWIN  297
                                           .......210.......220.......230.......240.......250.......260.......270.......280.......290.......300

                                           *****************************************************:**:****************..**********:** * ***:********
P18507-2|GABRG2_Homo_sapiens               KDAVPARTSLGITTVLTMTTLSTIARKSLPKVSYVTAMDLFVSVCFIFVFSALVEYGTLHYFVSNRKPSKDKDKKKKNPLLRMFSFK-APTIDIRPRSAT  396
A0A2R9CM76|GABRG2_Pan_paniscus             KDAVPARTSLGITTVLTMTTLSTIARKSLPKVSYVTAMDLFVSVCFIFVFSALVEYGTLHYFVSNRKPSKDKDKKKKNPLLRMFSFK-APTIDIRPRSAT  396
A0A2I3RPH6|GABRG2_Pan_troglodytes          KDAVPARTSLGITTVLTMTTLSTIARKSLPKVSYVTAMDLFVSVCFIFVFSALVEYGTLHYFVSNRKPSKDKDKKKKNPLLRMFSFK-APTIDIRPRSAT  396
F7A3C2|GABRG2_Macaca_mulatta               KDAVPARTSLGITTVLTMTTLSTIARKSLPKVSYVTAMDLFVSVCFIFVFSALVEYGTLHYFVSNRKPSKDKDKKKKNPLLRMFSFK-APTIDIRPRSAT  396
XP_004737727.1|GABRG2_Mustela_putorius_furo KDAVPARTSLGITTVLTMTTLSTIARKSLPKVSYVTAMDLFVSVCFIFVFSALVEYGTLHYFVSNRKPSKDKDKKKKNPLLRMFSFK-APTIDIRPRSAT  396
NP_001380704.1|GABRG2_Rattus_norvegicus    KDAVPARTSLGITTVLTMTTLSTIARKSLPKVSYVTAMDLFVSVCFIFVFSALVEYGTLHYFVSNRKPSKDKDKKKKNPLLRMFSFK-APTIDIRPRSAT  395
P22723|GABRG2_Mus_musculus                 KDAVPARTSLGITTVLTMTTLSTIARKSLPKVSYVTAMDLFVSVCFIFVFSALVEYGTLHYFVSNRKPSKDKDKKKKNPLLRMFSFK-APTIDIRPRSAT  395
F1RDP2|GABRG2_Danio_rerio                  KDAVPARTSLGITTVLTMTTLSTIARKSLPKVSYVTAMDLFVSVCFIFVFAALIEYGTLHYFVSNRKPSKKSDKKKKNPLLRLFSSKQAPTVDIRPRSAT  397
E2RSQ1|GABRG2_Canis_lupus_familiaris       KDAVPARTSLGITTVLTMTTLSTIARKSLPKVSYVTAMDLFVSVCFIFVFSALVEYGTLHYFVSNRKPSKDKDKKKKNPLLRMFSFK-APTIDIRPRSAT  396
P22300|GABRG2_Bos_taurus                   KDAVPARTSLGITTVLTMTTLSTIARKSLPKVSYVTAMDLFVSVCFIFVFSALVEYGTLHYFVSNRKPSKDKDKKKKNPLLRMFSFK-APTIDIRPRSAT  396
                                           .......310.......320.......330.......340.......350.......360.......370.......380.......390.......400

                                            *******::*****************:**********:*******:*******:***:**:************ ***:***.*****
P18507-2|GABRG2_Homo_sapiens               -IQMNNATHLQERDEEYGYECLDGKDCASFFCCFEDCRTGAWRHGRIHIRIAKMDSYARIFFPTAFCLFNLVYWVSYLYL  475
A0A2R9CM76|GABRG2_Pan_paniscus             -IQMNNATHLQERDEEYGYECLDGKDCASFFCCFEDCRTGAWRHGRIHIRIAKMDSYARIFFPTAFCLFNLVYWVSYLYL  475
A0A2I3RPH6|GABRG2_Pan_troglodytes          -IQMNNATHLQERDEEYGYECLDGKDCASFFCCFEDCRTGAWRHGRIHIRIAKMDSYARIFFPTAFCLFNLVYWVSYLYL  475
F7A3C2|GABRG2_Macaca_mulatta               -IQMNNATHLQERDEEYGYECLDGKDCASFFCCFEDCRTGAWRHGRIHIRIAKMDSYARIFFPTAFCLFNLVYWVSYLYL  475
XP_004737727.1|GABRG2_Mustela_putorius_furo -IQMNNATHLQERDEEYGYECLDGKDCASFFCCFEDCRTGAWRHGRIHIRIAKMDSYARIFFPTAFCLFNLVYWVSYLYL  475
NP_001380704.1|GABRG2_Rattus_norvegicus    -IQMNNATHLQERDEEYGYECLDGKDCASFFCCFEDCRTGAWRHGRIHIRIAKMDSYARIFFPTAFCLFNLVYWVSYLYL  474
P22723|GABRG2_Mus_musculus                 -IQMNNATHLQERDEEYGYECLDGKDCASFFCCFEDCRTGAWRHGRIHIRIAKMDSYARIFFPTAFCLFNLVYWVSYLYL  474
F1RDP2|GABRG2_Danio_rerio                  AIQMNNATQMQERDEEYGYECLDGKDCTSFFCCFEDCRSGAWRHGRLHIRVAKIDSYARIFFPTAFGLFNVVYWFSYLYL  477
E2RSQ1|GABRG2_Canis_lupus_familiaris       -IQMNNATHLQERDEEYGYECLDGKDCASFFCCFEDCRTGAWRHGRIHIRIAKMDSYARIFFPTAFCLFNLVYWVSYLYL  475
P22300|GABRG2_Bos_taurus                   -IQMNNATHLQERDEEYGYECLDGKDCASFFCCFEDCRTGAWRHGRIHIRIAKMDSYARIFFPTAFCLFNLVYWVSYLYL  475
                                           .......410.......420.......430.......440.......450.......460.......470.......480
```

```
                                                                                               :*:.*  .  :
Q99928|GABRG3_Homo_sapiens                  MAPKLLLLLCLFSGLHARSRKV--EEDEYEDSSSNQKWVLAPKSQDTDVTLILNKLLREYDKKLRPDIGIKPTVIDVDIYVNSIGPVSSINMEYQIDIFF   98
A0A2R9BD98|GABRG3_Pan_paniscus              ------------------SRKV--EEDEYEDSSSNQKWVLAPKSQDTDVTLILNKLLREYDKKLRPDIGIKPTVIDVDIYVNSIGPVSSINMEYQIDIFF   80
A0A2I3TTK9|GABRG3_Pan_troglodytes           MAPKLLLLLCLFSGLHARSRKV--EEDEYEDSSSNQKWVLAPKSQDTDVTLILNKLLREYDKKLRPDIGIKPTVIDVDIYVNSIGPVSSINMEYQIDIFF   98
F7HI54|GABRG3_Macaca_mulatta                MAPKLLLLLCLFSGLHARSRKV--EEDEYEDSSSNQKWVLAPKSQDTDVTLILNKLLREYDKKLRPDIGIKPTVIDVDIYVNSIGPVSSINMEYQIDIFF   98
P28473|GABRG3_Rattus_norvegicus             MAAKLLLLLCLFSGLHARSRRV--EEDDSEDSPSNQKWVLAPKSQDTDVTLILNKLLREYDKKLRPDIGIKPTVIDVDIYVNSIGPVSSINMEYQIDIFF   98
P27681|GABRG3_Mus_musculus                  MAAKLLLLLCLFSGLHARSRRV--EEDENEDSPSNQKWVLAPKSQDTDVTLILNKLLREYDKKLRPDIGIKPTVIDVDIYVNSIGPVSSINMEYQIDIFF   98
F1PHI6|GABRG3_Canis_lupus_familiaris        MAPELLLLLCLSWGVHARSRKV--EEDEYEDSSSNQKWVLAPKSQDTDVTLILNKLLREYDKKLRPDIGIKPTVIDVDIYVNSIGPVSSINMEYQIDIFF   98
XP_009300843.1|GABRG3_Danio_rerio           MTTKLFLYFLLLSVFRACSPTFASDDDEYDDVTVNQ--MLAPKTHETDATQILNNLLKEYDKKLRPDIGVKPTVIDVDIYVNSIGPVSSINMEYQIDIIF   98
XP_024838027.1|GABRG3_Bos_taurus            MAPKLLLLLCLLSGLHARTRKV--EEDEYEDSSSNQKWVLAPKSQDTDVTLILNKLLREYDKKLRPDIGIKPTVIDVDIYVNSIGPVSSINMEYQIDIFF   98
XP_044926358.1|GABRG3_Mustela_putorius_furo -------------------------------------------------MVVLKDLQAHNE---------------------------------------   12
                                            1.......10........20........30........40........50........60........70........80........90.......100

                                                                                                                   *.**                                    **::*
Q99928|GABRG3_Homo_sapiens                  AQTWTDSRLRFNSTMKILTLNSNMVGLIWIPDTIFRNSKTAEAHWITTPNQLLRIWNDGKILYTLRLTINAECQLQLHNFPMDEHSCPLIFSSYGYPKEE  198
A0A2R9BD98|GABRG3_Pan_paniscus              AQTWTDSRLRFNSTMKILTLNSNMVGLIWIPDTIFRNSKTAEAHWITTPNQLLRIWNDGKILYTLRLTINAECQLQLHNFPMDEHSCPLIFSSYGYPKEE  180
A0A2I3TTK9|GABRG3_Pan_troglodytes           AQTWTDSRLRFNSTMKILTLNSNMVGLIWIPDTIFRNSKTAEAHWITTPNQLLRIWNDGKILYTLRLTINAECQLQLHNFPMDEHSCPLIFSSYGYPKEE  198
F7HI54|GABRG3_Macaca_mulatta                AQTWTDSRLRFNSTMKILTLNSNMVGLIWIPDTIFRNSKTAEAHWITTPNQLLRIWNDGKILYTLRLTINAECQLQLHNFPMDEHSCPLIFSSYGYPKEE  198
P28473|GABRG3_Rattus_norvegicus             AQTWTDSRLRFNSTMKILTLNSNMVGLIWIPDTIFRNSKTAEAHWITTPNQLLRIWNDGKILYTLRLTINAECQLQLHNFPMDAHACPLTFSSYGYPKEE  198
P27681|GABRG3_Mus_musculus                  AQTWTDSRLRFNSTMKILTLNSNMVGLIWIPDTIFRNSKTAEAHWITTPNQLLRIWNDGKILYTLRLTINAECQLQLHNFPMDAHACPLTFSSYGYPKEE  198
F1PHI6|GABRG3_Canis_lupus_familiaris        AQTWTDSRLRFNSTMKILTLNSNMVGLIWIPDTIFRNSKTAEAHWITTPNQLLRIWNDGKILYTLRLTINAECQLQLHNFPMDEHSCPLIFSSYGYPKEE  198
XP_009300843.1|GABRG3_Danio_rerio           AQTWTDSRLRYNSTMKILTLNSNMVGLIWLPDTIFRNSKSADSHWITTPNQLLRIWNDGKILYTLRLTINAECQLQLHNFPMDEHSCPLIFSSYGYPRDE  198
XP_024838027.1|GABRG3_Bos_taurus            AQTWTDSRLRFNSTMKILTLNSNMVGLIWIPDTIFRNSKTAEAHWITTPNQLLRIWNDGKILYTLRLTINAECQLQLHNFPMDEHSCPLIFSSYGYPKEE  198
XP_044926358.1|GABRG3_Mustela_putorius_furo -------------------------------------------------------WRDG------------------------------------YPKEE   21
                                            .......110.......120.......130.......140.......150.......160.......170.......180.......190.......200

                                            ***:**:***:*********************::: *:********:**:**************************************:*
Q99928|GABRG3_Homo_sapiens                  MIYRWRKNSVEAADQKSWRLYQFDFMGLRNTTEIVTTSAGDYVVMTIYFELSRRMGYFTIQTYIPCILTVVLSWVSFWIKKDATPARTAL----------  288
A0A2R9BD98|GABRG3_Pan_paniscus              MIYRWRKNSVEAADQKSWRLYQFDFMGLRNTTEIVTTSAGDYVVMTIYFELSRRMGYFTIQTYIPCILTVVLSWVSFWIKKDATPARTAL----------  270
A0A2I3TTK9|GABRG3_Pan_troglodytes           MIYRWRKNSVEAADQKSWRLYQFDFMGLRNTTEIVTTSAGDYVVMTIYFELSRRMGYFTIQTYIPCILTVVLSWVSFWIKKDATPARTAL----------  288
F7HI54|GABRG3_Macaca_mulatta                MIYRWRKNSVEAADQKSWRLYQFDFMGLRNTTEIVTTSAGDYVVMTIYFELSRRMGYFTIQTYIPCILTVVLSWVSFWIKKDATPARTAL----------  288
P28473|GABRG3_Rattus_norvegicus             MIYRWRKNSVEAADQKSWRLYQFDFMGLRNTTEIVTTSAGDYVVMTIYFELSRRMGYFTIQTYIPCILTVVLSWVSFWIKKDATPARTTL----------  288
P27681|GABRG3_Mus_musculus                  MIYRWRKNSVEAADQKSWRLYQFDFMGLRNTTEIVTTSAGDYVVMTIYFELSRRMGYFTIQTYIPCILTVVLSWVSFWIKKDATPARTTL----------  288
F1PHI6|GABRG3_Canis_lupus_familiaris        MIYRWRKNSVEAADQKSWRLYQFDFMGLRNTTEIVTTSAGDYVVMTIYFELSRRMGYFTIQTYIPCILTVVLSWVSFWIKKDATPARTAL----------  288
XP_009300843.1|GABRG3_Danio_rerio           MIYKWRRNSVQAADQKSWRLYQFDFMGLRNTTDVIKTTAGDYVVMTVYFDLSRRMGYFTIQTYIPCILTVVLSWVSFWIKKDATPARTALVSCFKSMSCP  298
XP_024838027.1|GABRG3_Bos_taurus            MIYRWRKNSVEAADQKSWRLYQFDFMGLRNTTEIVQTSAGDYVVMTIYFELSRRMGYFTIQTYIPCILTVVLSWVSFWIKKDATPARTAL----------  288
XP_044926358.1|GABRG3_Mustela_putorius_furo MIYRWRKNSVEAADQKSWRLYQFDFMGLRNTTEIVTTSAGDYVVMTIYFELSRRMGYFTIQTYIPCILTVVLSWVSFWIKKDATPARTAL----------  111
                                            .......210.......220.......230.......240.......250.......260.......270.......280.......290.......300

                                                   *************:**.************************************  .*:*.  *..                     ****:**
Q99928|GABRG3_Homo_sapiens                  -------GITTVLTMTTLSTIARKSLPRVSYVTAMDLFVTVCFLFVFAALMEYATLNYYSS-CRKPTTTKKTTSLLHPDSSRWIPERISLQAPSNYSLLD  380
A0A2R9BD98|GABRG3_Pan_paniscus              -------GITTVLTMTTLSTIARKSLPRVSYVTAMDLFVTVCFLFVFAALMEYATLNYYSS-CRKPTTTKKTTSLLHPDSSRWIPERISLQAPSNYSLLD  362
A0A2I3TTK9|GABRG3_Pan_troglodytes           -------GITTVLTMTTLSTIARKSLPRVSYVTAMDLFVTVCFLFVFAALMEYATLNYYSS-CRKPTTTKKTTSLLHPDSSRWIPERISLQAPSNYSLLD  380
F7HI54|GABRG3_Macaca_mulatta                -------GITTVLTMTTLSTIARKSLPRVSYVTAMDLFVTVCFLFVFAALMEYATLNYYSS-CRKPTTTKKTTSLLHSDSSRWIPERISLQAPSNYSLLD  380
P28473|GABRG3_Rattus_norvegicus             -------GITTVLTMTTLSTIARKSLPRVSYVTAMDLFVTVCFLFVFAALMEYATLNYYSS-CRKPTIRKKKTSLLHPDSTRWIPDRISLQAPSNYSLLD  380
P27681|GABRG3_Mus_musculus                  -------GITTVLTMTTLSTIARKSLPRVSYVTAMDLFVTVCFLFVFAALMEYATLNYYSS-CRKPTIRKKKTSLLHPDSTRWIPDRISLQAPSNYSLLD  380
F1PHI6|GABRG3_Canis_lupus_familiaris        -------GITTVLTMTTLSTIARNSLPRVSYVTAMDLFVTVCFLFVFAALMEYATLNYYSS-CRKPTTTKKKTSLLHPDSSRWIHERISLQAPSNYSLLD  380
XP_009300843.1|GABRG3_Danio_rerio           GKCHCCQGITTVLTMTTLSTVARTSLPRVSYVTAMDLFVTVCFLFVFAALMEYATLNYYSYSARRPTCNKTKR--------------------SNYSVLD  378
XP_024838027.1|GABRG3_Bos_taurus            -------GITTVLTMTTLSTIARNSLPRVSYVTAMDLFVTVCFLFVFAALMEYATLNYYSS-CRKPPTTKKKTSLLHADSSRWIHERISLQAPSNYSLLD  380
XP_044926358.1|GABRG3_Mustela_putorius_furo -------GITTVLTMTTLSTIARNSLPRVSYVTAMDLFVTVCFLFVFAALMEYATLNYYSS-CRKPTTTKKKTSLLHPDSSRWIHERISLQAPSNYSLLD  203
                                            .......310.......320.......330.......340.......350.......360.......370.......380.......390.......400

                                            : ***.   *****:****::*:**********************.*:**:*::***: ***:**************:*********
Q99928|GABRG3_Homo_sapiens                  MRPPPTAMITLNNSVYWQEFEDTCVYECLDGKDCQSFFCCYEECKSGSWRKGRIHIDILELDSYSRVFFPTSFLLFNLVYWVGYLYL  467
A0A2R9BD98|GABRG3_Pan_paniscus              MRPPPPAMITLNNSVYWQEFEDTCVYECLDGKDCQSFFCCYEECKSGSWRKGRIHIDILELDSYSRVFFPTSFLLFNLVYWVGYLYL  449
A0A2I3TTK9|GABRG3_Pan_troglodytes           MRPPPPAMITLNNSVYWQEFEDTCVYECLDGKDCQSFFCCYEECKSGSWRKGRIHIDILELDSYSRVFFPTSFLLFNLVYWVGYLYL  467
F7HI54|GABRG3_Macaca_mulatta                MRPPPPAMTTLNNSVYWQEFEDTCVYECLDGKDCQSFFCCYEECKSGSWRKGRIHIDILELDSYSRVFFPTSFLLFNLVYWVGYLYL  467
P28473|GABRG3_Rattus_norvegicus             MRPPPPVMITLNNSMYWQEFEDTCVYECLDGKDCQSFFCCYEECKSGSWRRGRIHIDVSELDSYSRVFFPTSFLLFNLVYWVGYLYL  467
P27681|GABRG3_Mus_musculus                  MRPPPPVMITLNNSMYWQEFEDTCVYECLDGKDCQSFFCCYEECKSGSWRRGRIHIDVSELDSYSRVFFPTSFLLFNLVYWVGYLYL  467
F1PHI6|GABRG3_Canis_lupus_familiaris        MRPPPPAMITLNNSVYWQELEDTCVYECLDGKDCQSFFCCYEECKSGSWRKGRIHIDILELDSYSRVFFPTSFLLFNLVYWVGYLYL  467
XP_009300843.1|GABRG3_Danio_rerio           VGPPPT-VITLNNSMYWQEFDDACVYECLDGKDCQSFFCCYEECKDGAWRKGRVHIDILELDAYSRVFFPTSFLLFNVVYWVGYLYL  464
XP_024838027.1|GABRG3_Bos_taurus            MRPPPPVKVTLNNSVYWQELEDTCVYECLDGKDCQSFFCCYEECKSGSWRKGHIHIDILELDSYSRVFFPTSFLLFNLVYWVGYLYL  467
XP_044926358.1|GABRG3_Mustela_putorius_furo MRPPPPAMITLNNSVYWQEFEDTCVYECLDGKDCQSFFCCYEECKSGSWRKGRIHIDILELDSYSRVFFPTSFLLFNLVYWVGYLYL  290
                                            .......410.......420.......430.......440.......450.......460.......470.......480.......
```

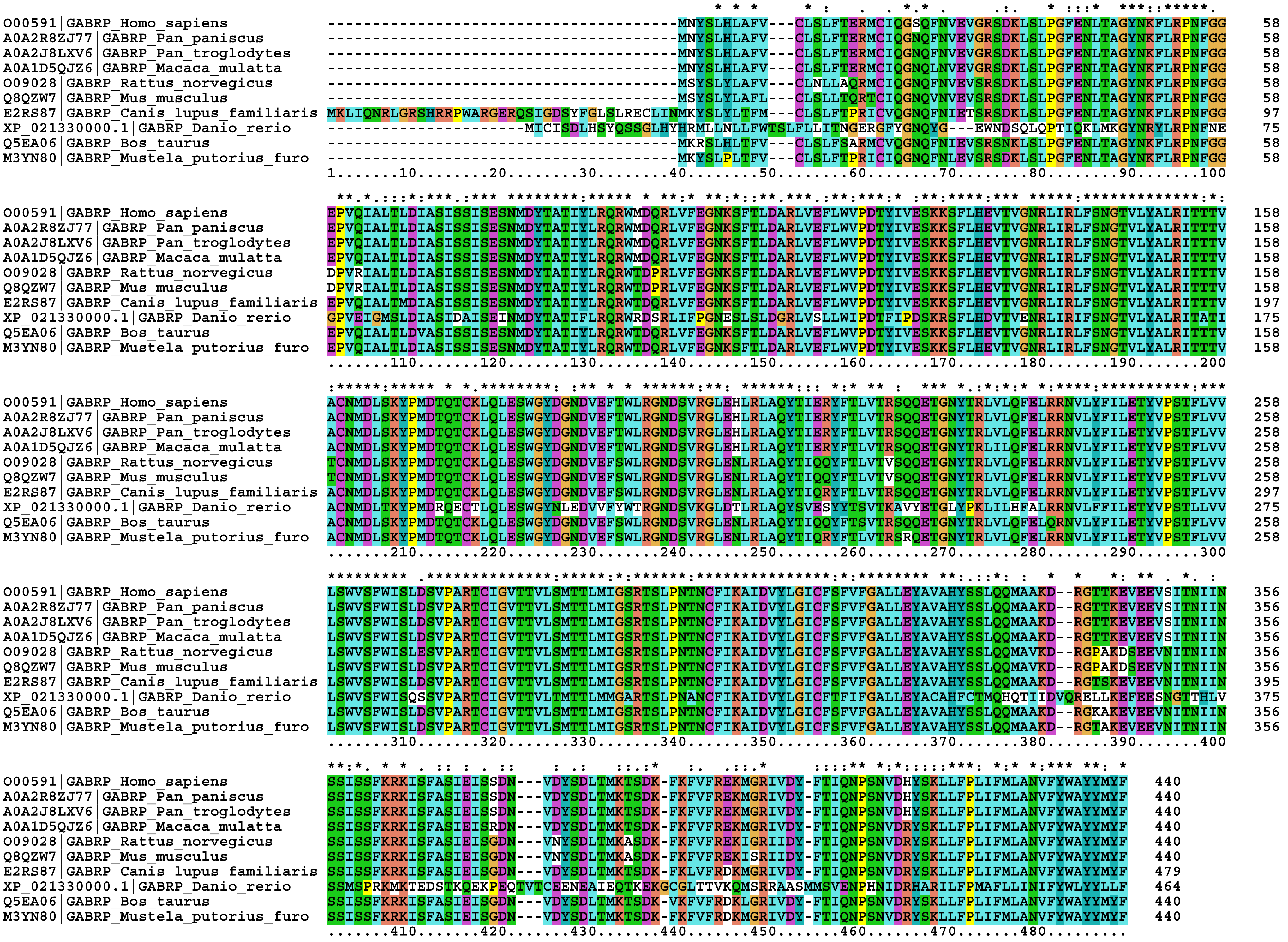

O00591|GABRP_Homo_sapiens ---------------------------------------MNYSLHLAFV---CLSLFTERMCIQGSQFNVEVGRSDKLSLPGFENLTAGYNKFLRPNFGG 58
A0A2R8ZJ77|GABRP_Pan_paniscus ---------------------------------------MNYSLHLAFV---CLSLFTERMCIQGNQFNVEVGRSDKLSLPGFENLTAGYNKFLRPNFGG 58
A0A2J8LXV6|GABRP_Pan_troglodytes ---------------------------------------MNYSLHLAFV---CLSLFTERMCIQGNQFNVEVGRSDKLSLPGFENLTAGYNKFLRPNFGG 58
A0A1D5QJZ6|GABRP_Macaca_mulatta ---------------------------------------MNYSLHLAFV---CLSLFTERMCIQGNQLNVEVGRSDKLSLPGFENLTAGYNKFLRPNFGG 58
O09028|GABRP_Rattus_norvegicus ---------------------------------------MSYSLYLAFV---CLNLLAQRMCIQGNQFNVEVSRSDKLSLPGFENLTAGYNKFLRPNFGG 58
Q8QZW7|GABRP_Mus_musculus ---------------------------------------MSYSLYLAFL---CLSLLTQRTCIQGNQVNVEVSRSDKLSLPGFENLTAGYNKFLRPNFGG 58
E2RS87|GABRP_Canis_lupus_familiaris MKLIQNRLGRSHRRPWARGERQSIGDSYFGLSLRECLINMKYSLYLTFM---CLSLFTPRICVQGNQFNIETSRSDKLSLPGFENLTAGYNKFLRPNFGG 97
XP_021330000.1|GABRP_Danio_rerio ----------------------MICISDLHSYQSSGLHYHRMLLNLLFWTSLFLLITNGERGFYGNQYG---EWNDSQLQPTIQKLMKGYNRYLRPNFNE 75
Q5EA06|GABRP_Bos_taurus ---------------------------------------MKRSLHLTFV---CLSLFSARMCVQGNQFNIEVSRSNKLSLPGFENLTAGYNKFLRPNFGG 58
M3YN80|GABRP_Mustela_putorius_furo ---------------------------------------MKYSLPLTFV---CLSLFTPRICIQGNQFNLEVSRSDKLSLPGFENLTAGYNKFLRPNFGG 58

O00591|GABRP_Homo_sapiens EPVQIALTLDIASISSISESNMDYTATIYLRQRWMDQRLVFEGNKSFTLDARLVEFLWVPDTYIVESKKSFLHEVTVGNRLIRLFSNGTVLYALRITTTV 158
A0A2R8ZJ77|GABRP_Pan_paniscus EPVQIALTLDIASISSISESNMDYTATIYLRQRWMDQRLVFEGNKSFTLDARLVEFLWVPDTYIVESKKSFLHEVTVGNRLIRLFSNGTVLYALRITTTV 158
A0A2J8LXV6|GABRP_Pan_troglodytes EPVQIALTLDIASISSISESNMDYTATIYLRQRWMDQRLVFEGNKSFTLDARLVEFLWVPDTYIVESKKSFLHEVTVGNRLIRLFSNGTVLYALRITTTV 158
A0A1D5QJZ6|GABRP_Macaca_mulatta EPVQIALTLDIASISSISESNMDYTATIYLRQRWMDQRLVFEGNKSFTLDARLVEFLWVPDTYIVESKKSFLHEVTVGNRLIRLFSNGTVLYALRITTTV 158
O09028|GABRP_Rattus_norvegicus DPVRIALTLDIASISSISESNMDYTATIYLRQRWTDPRLVFEGNKSFTLDARLVEFLWVPDTYIVESKKSFLHEVTVGNRLIRLFSNGTVLYALRITTTV 158
Q8QZW7|GABRP_Mus_musculus DPVRIALTLDIASISSISESNMDYTATIYLRQRWTDPRLVFEGNKSFTLDARLVEFLWVPDTYIVESKKSFLHEVTVGNRLIRLFSNGTVLYALRITTTV 158
E2RS87|GABRP_Canis_lupus_familiaris EPVQIALTMDIASISSISESNMDYTATIYLRQRWTDQRLVFEGNKSFTLDARLVEFLWVPDTYIVESKKSFLHEVTVGNRLIRLFSNGTVLYALRITTTV 197
XP_021330000.1|GABRP_Danio_rerio GPVEIGMSLDIASIDAISEINMDYTATIFLRQRWRDSRLIFPGNESLSLDGRLVSLLWIPDTFIPDSKRSFLHDVTVENRLIRIFSNGTVLYALRITATI 175
Q5EA06|GABRP_Bos_taurus EPVQIALTLDVASISSISESNMDYTATIYLRQRWTDQRLVFEGNKSFTLDARLVEFLWVPDTYIVESKKSFLHEVTVGNRLIRLFSNGTVLYALRITTTV 158
M3YN80|GABRP_Mustela_putorius_furo EPVQIALTLDIASISSISESNMDYTATIYLRQRWTDQRLVFEGNKSFTLDARLVEFLWVPDTYIVESKKSFLHEVTVGNRLIRLFSNGTVLYALRITTTV 158

O00591|GABRP_Homo_sapiens ACNMDLSKYPMDTQTCKLQLESWGYDGNDVEFTWLRGNDSVRGLEHLRLAQYTIERYFTLVTRSQQETGNYTRLVLQFELRRNVLYFILETYVPSTFLVV 258
A0A2R8ZJ77|GABRP_Pan_paniscus ACNMDLSKYPMDTQTCKLQLESWGYDGNDVEFTWLRGNDSVRGLEHLRLAQYTIERYFTLVTRSQQETGNYTRLVLQFELRRNVLYFILETYVPSTFLVV 258
A0A2J8LXV6|GABRP_Pan_troglodytes ACNMDLSKYPMDTQTCKLQLESWGYDGNDVEFTWLRGNDSVRGLEHLRLAQYTIERYFTLVTRSQQETGNYTRLVLQFELRRNVLYFILETYVPSTFLVV 258
A0A1D5QJZ6|GABRP_Macaca_mulatta ACNMDLSKYPMDTQTCKLQLESWGYDGNDVEFTWLRGNDSVRGLEHLRLAQYTIERYFTLVTRSQQETGNYTRLILQFELRRNVLYFILETYVPSTFLVV 258
O09028|GABRP_Rattus_norvegicus TCNMDLSKYPMDTQTCKLQLESWGYDGNDVEFSWLRGNDSVRGLENLRLAQYTIQQYFTLVTVSQQETGNYTRLVLQFELRRNVLYFILETYVPSTFLVV 258
Q8QZW7|GABRP_Mus_musculus TCNMDLSKYPMDTQTCKLQLESWGYDGNDVEFSWLRGNDSVRGLENLRLAQYTIQQYFTLVTVSQQETGNYTRLVLQFELRRNVLYFILETYVPSTFLVV 258
E2RS87|GABRP_Canis_lupus_familiaris ACNMDLSKYPMDTQTCKLQLESWGYDGNDVEFSWLRGNDSVRGLENLRLAQYTIQRYFTLVTRSQQETGNYTRLVLQFELRRNVLYFILETYVPSTFLVV 297
XP_021330000.1|GABRP_Danio_rerio ACNMDLTKYPMDRQECTLQLESWGYNLEDVVFYWTRGNDSVKGLDTLRLAQYSVESYYTSVTKAVYETGLYPKLILHFALRRNVLFFILETYVPSTLLVV 275
Q5EA06|GABRP_Bos_taurus ACNMDLSKYPMDTQTCKLQLESWGYDGNDVEFSWLRGNDSVRGLENLRLAQYTIQQYFTSVTRSQQETGNYTRLVLQFELQRNVLYFILETYVPSTFLVV 258
M3YN80|GABRP_Mustela_putorius_furo ACNMDLSKYPMDTQTCKLQLESWGYDGNDVEFSWLRGNDSVRGLENLRLAQYTIQRYFTLVTRSRQETGNYTRLVLQFELRRNVLYFILETYVPSTFLVV 258

O00591|GABRP_Homo_sapiens LSWVSFWISLDSVPARTCIGVTTVLSMTTLMIGSRTSLPNTNCFIKAIDVYLGICFSFVFGALLEYAVAHYSSLQQMAAKD--RGTTKEVEEVSITNIIN 356
A0A2R8ZJ77|GABRP_Pan_paniscus LSWVSFWISLDSVPARTCIGVTTVLSMTTLMIGSRTSLPNTNCFIKAIDVYLGICFSFVFGALLEYAVAHYSSLQQMAAKD--RGTTKEVEEVSITNIIN 356
A0A2J8LXV6|GABRP_Pan_troglodytes LSWVSFWISLDSVPARTCIGVTTVLSMTTLMIGSRTSLPNTNCFIKAIDVYLGICFSFVFGALLEYAVAHYSSLQQMAAKD--RGTTKEVEEVSITNIIN 356
A0A1D5QJZ6|GABRP_Macaca_mulatta LSWVSFWISLDSVPARTCIGVTTVLSMTTLMIGSRTSLPNTNCFIKAIDVYLGICFSFVFGALLEYAVAHYSSLQQMAAKD--RGTTKEVEEVSITNIIN 356
O09028|GABRP_Rattus_norvegicus LSWVSFWISLESVPARTCIGVTTVLSMTTLMIGSRTSLPNTNCFIKAIDVYLGICFSFVFGALLEYAVAHYSSLQQMAVKD--RGPAKDSEEVNITNIIN 356
Q8QZW7|GABRP_Mus_musculus LSWVSFWISLDSVPARTCIGVTTVLSMTTLMIGSRTSLPNTNCFIKAIDVYLGICFSFVFGALLEYAVAHYSSLQQMAVKD--RGPAKDSEEVNITNIIN 356
E2RS87|GABRP_Canis_lupus_familiaris LSWVSFWISLDSVPARTCIGVTTVLSMTTLMIGSRTSLPNTNCFIKAIDVYLGICFSFVFGALLEYAVAHYSSLQQMAAKD--RGTSKEVEEVNITNIIN 395
XP_021330000.1|GABRP_Danio_rerio LSWVSFWISQSSVPARTCIGVTTVLTMTTLMMGARTSLPNANCFIKAIDVYLGICFTFIFGALLEYACAHFCTMQHQTIIDVQRELLKEFEESNGTTHLV 375
Q5EA06|GABRP_Bos_taurus LSWVSFWISLDSVPARTCIGVTTVLSMTTLMIGSRTSLPNTNCFIKAIDVYLGICFSFVFGALLEYAVAHYSSLQQMAAKD--RGKAKEVEEVNITNIIN 356
M3YN80|GABRP_Mustela_putorius_furo LSWVSFWISLDSVPARTCIGVTTVLSMTTLMIGSRTSLPNTNCFIKAIDVYLGICFSFVFGALLEYAVAHYSSLQQMAAKD--RGTAKEVEEVNITNIIN 356

O00591|GABRP_Homo_sapiens SSISSFKRKISFASIEISSDN---VDYSDLTMKTSDK-FKFVFREKMGRIVDY-FTIQNPSNVDHYSKLLFPLIFMLANVFYWAYYMYF 440
A0A2R8ZJ77|GABRP_Pan_paniscus SSISSFKRKISFASIEISSDN---VDYSDLTMKTSDK-FKFVFREKMGRIVDY-FTIQNPSNVDHYSKLLFPLIFMLANVFYWAYYMYF 440
A0A2J8LXV6|GABRP_Pan_troglodytes SSISSFKRKISFASIEISSDN---VDYSDLTMKTSDK-FKFVFREKMGRIVDY-FTIQNPSNVDHYSKLLFPLIFMLANVFYWAYYMYF 440
A0A1D5QJZ6|GABRP_Macaca_mulatta SSISSFKRKISFASIEISRDN---VDYSDLTMKTSDK-FKFVFREKMGRIVDY-FTIQNPSNVDRYSKLLFPLIFMLANVFYWAYYMYF 440
O09028|GABRP_Rattus_norvegicus SSISSFKRKISFASIEISGDN---VNYSDLTMKASDK-FKFVFREKIGRIIDY-FTIQNPSNVDRYSKLLFPLIFMLANVFYWAYYMYF 440
Q8QZW7|GABRP_Mus_musculus SSISSFKRKISFASIEISGDN---VNYSDLTMKASDK-FKFVFREKISRIIDY-FTIQNPSNVDRYSKLLFPLIFMLANVFYWAYYMYF 440
E2RS87|GABRP_Canis_lupus_familiaris SSISSFKRKISFASIEISGDN---VDYSDLTMKTSDK-FKLVFRDKMGRIVDY-FTIQNPSNVDRYSKLLFPLIFMLANVFYWAYYMYF 479
XP_021330000.1|GABRP_Danio_rerio SSMSPRKMKTEDSTKQEKPEQTVTCEENEAIEQTKEKGCGLTTVKQMSRRAASMMSVENPHNIDRHARILFPMAFLLINIFYWLYYLLF 464
Q5EA06|GABRP_Bos_taurus SSISSFKRKISFASIEISGDN---VDYSDLTMKTSDK-VKFVFRDKLGRIVDY-FTIQNPSNVDRYSKLLFPLIFMLANVFYWAYYMYF 440
M3YN80|GABRP_Mustela_putorius_furo SSISSFKRKISFASIEISGDN---VDYSDLTMKTSDK-FKFVFRDKMGRIVDY-FTIQNPSNVDRYSKLLFPLIFMLANVFYWAYYMYF 440

```
                                                          .   .    :   ::.  . * :   ... .:. ...:. :*::*. ** ****:***.** * :*: ::**:.***:
Q9UN88|GABRQ_Homo_sapiens           MGIRGMLRAAVILLLIRTWLAEGNYPSPIPKFHFEFSSAVPEVVLNLFNCKNCANEAVVQKILDRVLSRYDVRLRPNFGGAPVPVRISIYVTSIEQISEM  100
A0A2R9ALH2|GABRQ_Pan_paniscus       MGIRGMLRAAVILLLIRTWLAEGNYPSPIPKFHFEFSSAVPEVVLNLFNCKNCANEAVVQKILDRVLSRYDVRLRPNFGGAPVPVRISIYVTSIEQISEM  100
A0A6D2XHJ7|GABRQ_Pan_troglodytes    MGIRGMLRAAVILLLIRTWLAEGNYPSPIPKFHFEFSSAVPEVVLNLFNCKNCANEAVVQKILDRVLSRYDVRLRPNFGGAPVPVRISIYVTSIEQISEM  100
F7F4H9|GABRQ_Macaca_mulatta         MGIRGMLRAAVILLLIRTWLAEGNYPSPIPKFHFEFSSAVPEVVLNLFNCKNCANEAVVQKILDRVLSRYDVRLRPNFGGAPVPVRISIYVTSIEQISEM  100
M3YJY6|GABRQ_Mustela_putorius_furo  MGIRGMLRAAVLLLLIRTWLAESHDPSPTPEFHFELSPTVPEVVLSLFNCKNCANEAVVHKILDRVLSKYDVRLRPNFGGAPVPVGISMYVSSIEQISEM  100
G3V875|GABRQ_Rattus_norvegicus      -----MLRAAALLLLIRTWLAESNDPSPTPKFHFELSSSMPEVILDLFNCKNCANEAVVQKILDRVLSTYDVRLRPNFGGAPVPVSISIYVSSIEQISEI   95
Q9JLF1|GABRQ_Mus_musculus           MGIRGMLRAAALLLLIRTWLAESNGPSPTPKFHFELSSSTPEVILDLFNCKNCANEAVVQKILDRVLSTYDVRLRPNFGGAPVPVSVSIYVSSIEQISEI  100
F6XRX3|GABRQ_Canis_lupus_familiaris MGIRGILRAAVLLLLIRTWLAEGNEPSPTPKFHFELSPTVPEVILNLFNCKKCANEGVVHKILDRVLSNYDVRLRPNFGGAPVPVGVSMYVSSIEQISEM  100
E1BJH4|GABRQ_Bos_taurus             MSIRGMLRAAVFLLLIRTWLAEGSDLSSTPKFHVELSSTVPEVVLNLFDCKNCANEATVHKILDRVLSNYDSRLRPNFGGTPVPVGVSIYVSSIEQISEV  100
F1Q4Y6|GABRB4_Danio_rerio           --------------------MLGPQEDKLCGIVSALAAL-SFVCFAQSSSTGSTGISVAKTTVDKLLKGYDIRLRPDFGGPPVIVGMSINIASIDSISEV   79
                                    1.......10........20........30........40........50........60........70........80........90.......100

                                    .*******:::*:*:* **** *  ****** *: ::**:** **:*.*.:*:*. *.:**:::******* **:*:****** ::*.::*:*:* *.*
Q9UN88|GABRQ_Homo_sapiens           NMDYTITMFFHQTWKDSRLAYYETTLNLTLDYRMHEKLWVPDCYFLNSKDAFVHDVTVENRVFQLHPDGTVRYGIRLTTTAACSLDLHKFPMDKQACNLV  200
A0A2R9ALH2|GABRQ_Pan_paniscus       NMDYTITMFFHQTWKDSRLAYYETTLNLTLDYRMHEKLWVPDCYFLNSKDAFVHDVTVENRVFQLHPDGTVRYGIRLTTTAACSLDLHKFPMDKQACNLV  200
A0A6D2XHJ7|GABRQ_Pan_troglodytes    NMDYTITMFFHQTWKDSRLAYYETTLNLTLDYRMHEKLWVPDCYFLNSKDAFVHDVTVENRVFQLHPDGTVRYGIRLTTTAACSLDLHKFPMDKQACNLV  200
F7F4H9|GABRQ_Macaca_mulatta         NMDYTITMFFHQTWKDSRLAYYETTLNLTLDYRMHEKLWVPDCYFLNSKDAFVHDVTVENRVFQLHPDGTVRYGIRLTTTAACSLDLHKFPMDKQACNLV  200
M3YJY6|GABRQ_Mustela_putorius_furo  TMDYTITMFFHQTWKDPRLAYHETNLNLTLDYRMLEKLWVPDCYFLNSKDAFVHDITVENRVFQLHPDGTVRYGMRLTTTAACSLDLQKFPLDKQACKLE  200
G3V875|GABRQ_Rattus_norvegicus      NMDYTITMFLHQTWKDTRLAYYETNLNLTLDYRMHEKLWVPDCYFVNSKDAFVHDVTAENRVFQLHPDGTVQYGIRLTTTAACSLDLQKFPMDKQSCKLE  195
Q9JLF1|GABRQ_Mus_musculus           NMDYTITMFLHQTWKDTRLAYYETNLNLTLDYRMHEKLWVPDCYFVNSKDAFVHDVTVENRVFQLHPDGTVRYGIRLTTTAACSLDLQKFPMDKQSCKLE  200
F6XRX3|GABRQ_Canis_lupus_familiaris TMDYTITMFFHQTWKDPRLAYHETDLNLTLDYRMLEKLWVPDCYFLNSKDAFVHDVTVENRVFQLHPDGTVRYGMRLTTTAACSLDLQKFPLDKQTCKLE  200
E1BJH4|GABRQ_Bos_taurus             TMDYTITMFFHQTWKDPRLAYHETNLNLTLDYRLFEKLWVPDCYFLNSKEAFVHDATVENRVFQLHPDGTVRYGIRLTTTAACSMNLEKFPLDKQTCKLE  200
F1Q4Y6|GABRB4_Danio_rerio           NMDYTITMYFQQSWRDKRLAYAEMALNLTLDNRVADQLWLPDTYFLNDKKSFLHGVTVKNRMIRLHPDGTVLYGLRITTTAACMMDLRRYPLDEQNCTLE  179
                                    .......110.......120.......130.......140.......150.......160.......170.......180.......190.......200

                                    :******.:**:: *: ..**:  .: *.:**:: :   : ::*: * **** ** : * ::*::  :::* * *::* *: **:***:** :*****::
Q9UN88|GABRQ_Homo_sapiens           VESYGYTVEDIILFWDDNGNAIHMTEELHIPQFTFLGRTITSKEVYFYTGSYIRLILKFQVQREVNSYLVQVYWPTVLTTITSWISFWMNYDSSAARVTI  300
A0A2R9ALH2|GABRQ_Pan_paniscus       VESYGYTVEDIILFWDDNGNAIHMTEELHIPQFTFLGRTITSKEVYFYTGSYIRLILKFQVQREVNSYLVQVYWPTVLTTITSWISFWMNYDSSAARVTI  300
A0A6D2XHJ7|GABRQ_Pan_troglodytes    VESYGYTVEDIILFWDDNGNAIHMTEELHIPQFTFLGRTITSKEVYFYTGSYIRLILKFQVQREVNSYLVQVYWPTVLTTITSWISFWMNYDSSAARVTI  300
F7F4H9|GABRQ_Macaca_mulatta         VESYGYTVEDIILFWDDNGNAIHMTEELHIPQFTFLGRTITSKEVYFYTGSYIRLILKFQVQREVNSYLVQVYWPTVLTTITSWISFWMNYDSSAARVTI  300
M3YJY6|GABRQ_Mustela_putorius_furo  VESYGYTVEDIVLYWEGDGNAIQGTEQLHIPQFSFLGKTMSSKEMAFYTGSYVRLILRFLVQREITSYLVQIYWPTVLTTVVSWISFWMNYESSAARVTV  300
G3V875|GABRQ_Rattus_norvegicus      VESYGYTVEDIVLSWE-DDNAIHITDELHIPQYTYLGRTITSKEVYFYTGSYMRLIVKFQVQREVRSYLVQVYWPTVLTTILSWISFWMNYGSSAARVTI  294
Q9JLF1|GABRQ_Mus_musculus           VESYGYTVEDIVLSWE-DDNAIHITDGLHIPQYTYLGRTITSKEVYFYTGSYMRLIVKFQVQREVRSYLVQVYWPTVLTTILSWISFWMNYDSSAARVTI  299
F6XRX3|GABRQ_Canis_lupus_familiaris VESYGYTVEDIVLYWEGNGNAIQGTEKLHIPQFSFLGKTMSSKEVFFYTGSYVRLILRFLVQREVTSYLVQIYWPTVLTTVVSWISFWMNYESSAARVTV  300
E1BJH4|GABRQ_Bos_taurus             VESYGYTVDDILLYWEGSGNAVQGTEKLHIPQFSFLGKTISTKEVFFYTGSYVRLILRFLVKREVTSYLVQIYWPSVLTTVVSWISFWMNYESSAARVTV  300
F1Q4Y6|GABRB4_Danio_rerio           IESYGYTTDDIVFFWQGGDNAVTGVDKLELPQFSIVELRLVSREVRFTTGSYPRLSLSFRIKRNIGYFILQTYMPSILITILSWVSFWINYDASAARVAL  279
                                    .......210.......220.......230.......240.......250.......260.......270.......280.......290.......300

                                    *:*::* :.:*::*: : :*:.. :****:*:: *:.****:*:**. :**::::.: .::  :  .:  :.   : **.:  :
Q9UN88|GABRQ_Homo_sapiens           GLTSMLILTTIDSHLRDKLPNISCIKAIDIYILVCLFFVFLSLLEYVYINYLFYSR-GPRRQPRRHRRPRRV-IARYRYQQVVVG---------------  383
A0A2R9ALH2|GABRQ_Pan_paniscus       GLTSMLILTTIDSHLRDKLPNISCIKAIDIYILVCLFFVFLSLLEYVYINYLFYSR-GPRRQPRRRRRPRRV-IAHYRYQQVVVG---------------  383
A0A6D2XHJ7|GABRQ_Pan_troglodytes    GLTSMLILTTIDSHLRDKLPNISCIKAIDIYILVCLFFVFLSLLEYVYINYLFYSR-GPRRQPRRRRRPRRV-IAHYRYQQVVVG---------------  383
F7F4H9|GABRQ_Macaca_mulatta         GLTSMLILTTIDSHLRDKLPNISCIKAIDIYILVCLFFVFLSLLEYVYINYLFYSR-GPRRQPRRRRRPRRV-IAHYRYQQVVVE---------------  383
M3YJY6|GABRQ_Mustela_putorius_furo  GLTSMLILNAINSHLWNKLPQVACIKAIDIYMVVCFFFVFLSLLEYVSINYLFYSRGRSRRTQRRQRRARRV-MARYRYQEVTVQ---------------  384
G3V875|GABRQ_Rattus_norvegicus      GLTSILVLTTIDSHMRDKLPHISCIKAIDIYILVCLFFVFLSLLEYVYINYLFFSQ-APRRNHRRCRKPRRV-VARYRYQEVVVA---------------  377
Q9JLF1|GABRQ_Mus_musculus           GLTSILVLTTIDSHMRDKLPHISCIKAIDIYILVCLFFVFLSLLEYVYINYLFFSQ-VPRRNHRRCRKPRRV-VARYRYQEVVVA---------------  382
F6XRX3|GABRQ_Canis_lupus_familiaris GLTSMLILNAINSHLRDKLPQVACIKAIDIYMVVCFLFVFLSLLEYVYINYLYYSRGGSRRNQRRRRRARRV-MARYRYREVMLQPFANRRSQEEMILSV  399
E1BJH4|GABRQ_Bos_taurus             GLTSMLILNSINSHLRNQFPQFSCIKAIDIYMVVCFFFVFLSLVEYVYINYLFYNRRGSRHSQRQLRRALRV-MERYRYREVIVH---------------  384
F1Q4Y6|GABRB4_Danio_rerio           GVTTVLTMTTINTHLRETLPKIPYVKAIDVYLMGCFVFVFLALLEYAFVNYVFFGR-GPQQQKKINERLNKANNERPRYEEKRLR---------------  363
                                    .......310.......320.......330.......340.......350.......360.......370.......380.......390.......400

                                                                                  . :                *  :                          . * *
Q9UN88|GABRQ_Homo_sapiens           ------------------------------------------NVQDGLINVEDGV----SSLPITPAQAPLASPESLGSLTSTSEQAQLATSESLSPLTS  437
A0A2R9ALH2|GABRQ_Pan_paniscus       ------------------------------------------NVQDGLINVEDGV----SSLPITPAQAPLASPESLGSLTSTSEQAQLATSESLSPLTS  437
A0A6D2XHJ7|GABRQ_Pan_troglodytes    ------------------------------------------NVQDGLINVEDGV----SSLPITPAQAPLASPESLGSLTSTSEQAQLATSESLSPLTS  437
F7F4H9|GABRQ_Macaca_mulatta         ------------------------------------------NVQDGLINVEDGV----SSLPITPAQAPLASPESLGSLTSTSEQAQLATSESLSPLTS  437
M3YJY6|GABRQ_Mustela_putorius_furo  -------------------------------------SRAGDGTEGDQISIEDES----GSLSSRPVQACLASLESLSSLISIPGQAPLATSESLSSLSS  443
G3V875|GABRQ_Rattus_norvegicus      ------------------------------------------NVQDGLINVEDRVEDRAGPLPDSPMQAHLASQESLGSLMFTSEEAQLATSESLSLLSS  435
Q9JLF1|GABRQ_Mus_musculus           ------------------------------------------NVQDGLINVEDRVEDRAGPLPDSPMQAHLASQESLGSLVFTSEQAQLATSESLSLLSS  440
F6XRX3|GABRQ_Canis_lupus_familiaris EEIDTHIATVDEESPLSPLPSPPPVLIHVGLANPDAINVSTISLREDQISIEDES----GSLSSRPARACLASLESLSSLTSIPGQAPLATAESLSSLSS  495
E1BJH4|GABRQ_Bos_taurus             ------------------------------------------TAFNDQVHLEDET----DSPPSSPWWAGLASPESLGSLASLTKEAPLASSESLSSLSS  438
F1Q4Y6|GABRB4_Danio_rerio           --------------------------------------------EQDSISA--------------PFQS--------------------------NTLRS  379
                                    .......410.......420.......430.......440.......450.......460.......470.......480.......490.......500
```

```
                                   :         .  :       .  .      :: :   .: .             :.  .  . :.    :
Q9UN88|GABRQ_Homo_sapiens          LSGQAPLATGESLSDLPSTSEQARHSYG-VRFNGFQADDSIFPTEIRNRVEAHGHGVTHDHEDSNESLSSDERHGHG--PSGKPMLHHGEKGVQEAGWD-   533
A0A2R9ALH2|GABRQ_Pan_paniscus      LSGQAPLATGESLSDLPSTSEQARHSYG-VRFNGFQADDSIIPTEIRNRVEAHGHGVTHDHEDSNESLSSDERHGHG--PSGKPMLHHGEKGVQEAGWD-   533
A0A6D2XHJ7|GABRQ_Pan_troglodytes   LSGQAPLATGESLSDLPSTSEQARHSYG-VRFNGFQADDSIIPTEIRNRVEAHGHGVTHDHEDSNESLSSDERHGHG--PSGKPMLHHGEKGVQEAGWD-   533
F7F4H9|GABRQ_Macaca_mulatta        LSGQAPLATGESLSDLPSTSEQARHSYG-VRFNGFQADDSIIPTEIRNRAEAHGRGVTHDHEDSDESLSSDEHHGHG--PSGKPMLHDGKKGVQEAGWD-   533
M3YJY6|GABRQ_Mustela_putorius_furo LSEQAWTGSRESLSDRPSTSEQALHRNG-FHFSGSETDDGVICPEIHNRADAHGHADTRDPEDPKENLSSDESHGHG--PRGRYLLVYGHRCVQEASYS-   539
G3V875|GABRQ_Rattus_norvegicus     ASSQVQLATGESLGDLPSTSEQTLPDYT-IHFHGFLTNDSILPIKIRSHSDALG------DEDSEESLSSEESYGHGGSPTGRLKLQISQRCVQEASWD-   527
Q9JLF1|GABRQ_Mus_musculus          ASSQTQLATGESLSDLPSTSEQTVPECT-IHFHGFLTNDSIIPIKIHSRSDACD------DEDSEESLSSEESHGHGSSHTGRLKLQISQRCVQEASWD-   532
F6XRX3|GABRQ_Canis_lupus_familiaris LSELASLASRESLSDLPSTPEQAPHRYG-IHVNGFENDDSVTPTEMRNLAEAHDHAEISDPEDPEENFSLDESPVHI--PSGRPLLLYGHRHVREASCSL  592
E1BJH4|GABRQ_Bos_taurus            LSDGASLATTESLSDLPSTSEQVVHDDG-IRVNGIDVDNSVVPTEIRNLAETHD-----PEEDPGESSDSDESEDNG--PSKKRLLVHGQRHVQEATYEL   530
F1Q4Y6|GABRB4_Danio_rerio          YTQRRNLY-------LEEQRKVGVDAYGNILLTTLEMNNEVMPSDVGSSV----------SDSRNSVMSFDSSGVQFRKPMGSR----------------   446
                                   .......510.......520.......530.......540.......550.......560.......570.......580.......590.......600

                                                                                . :   .  :   .  .*  .     .*    :. .: .    :. :*: .
Q9UN88|GABRQ_Homo_sapiens          -------------L-------DDNNDKSDCLAIKEQFKCDTNSTWGLNDDELMAHGQEKD--SSSESEDSCPPSPGCSFTEGFSFDLFNPDYVPKVDKWS   611
A0A2R9ALH2|GABRQ_Pan_paniscus      -------------L-------DDNNDKSDCLAIKEQFKCDTNSTWGLNDDGLMAHGQEKD--SSSESEDSCPPSPGCSFTEGFSFDLFNPDYVPKVDKWS   611
A0A6D2XHJ7|GABRQ_Pan_troglodytes   -------------L-------DDNNDKSDCLAIKEQFKCDTNSTWGLNDDGLMAHGQEKD--SSSESEDSCPPSPGCSFTEGFSFDLFNPDYVPKVDKWS   611
F7F4H9|GABRQ_Macaca_mulatta        -------------L-------DDNSVKSGCLAVEEQLKCDTNSTWGLNEDELMAHGHEKD--NSSESEDSCPPSPGCSFTEGFSFDLFNPDYVPKVDKWS   611
M3YJY6|GABRQ_Mustela_putorius_furo -------------LEEIRSLPDDIRVESGYLDLEKQLRRDLDSTWSLNVDGLPGFDQDKD--SNSESDDSRPPSPRCSFSEVFSSKLFDPDYVPQVDKWS   624
G3V875|GABRQ_Rattus_norvegicus     -------------IDKIESLQDDISIKSSWLGLDEQRKGDADSIWSLTDEELMACDQEKDSSSSSESEESCSPSPGCSFNEGFSFQLFNPNRVPKVDRWS   614
Q9JLF1|GABRQ_Mus_musculus          -------------LDKIEILQDDISITSSWLGLDEQCKGDADSIWSLTDEELMACDQEKD--SSSESEENCSPSPGCSFNEGFSFQLFKPNRVPKVDRWS   617
F6XRX3|GABRQ_Canis_lupus_familiaris DEIRSNLNEIRSNLDEIRSLPDDIRVESGYLDLEKQLRYDL-YNWRPNAKKFMRLNRRKD--SNSESDDSCPPSPGCSFTEGFSSKLFDPDYVPKVDKWS  689
E1BJH4|GABRQ_Bos_taurus            QEICNT-------LRDIHSLPDDVIVESGYPDLEEQLKRKVDSTGSLHSDDFMDFDGDKE--SNSESDNSFPPSPGCSFSKGFSSDLFHPDYIPKVDQCC   621
F1Q4Y6|GABRB4_Danio_rerio          -------------------------------------------------DGFSHHSLDR---SAMRSRANCRLRRRSS---KLKLKIPNLTDVSTIDKWS   491
                                   .......610.......620.......630.......640.......650.......660.......670.......680.......690.......700

                                   *::**::* .**::** *::
Q9UN88|GABRQ_Homo_sapiens          RFLFPLAFGLFNIVYWVYHMY   632
A0A2R9ALH2|GABRQ_Pan_paniscus      RFLFPLAFGLFNIVYWVYHMY   632
A0A6D2XHJ7|GABRQ_Pan_troglodytes   RFLFPLAFGLFNIVYWVYHMY   632
F7F4H9|GABRQ_Macaca_mulatta        RFLFPLAFGLFNIVYWVYHMY   632
M3YJY6|GABRQ_Mustela_putorius_furo RILFPLAFVVFNIVYWAYHLY   645
G3V875|GABRQ_Rattus_norvegicus     RFLFPLSFGLFNVVYWLYHIY   635
Q9JLF1|GABRQ_Mus_musculus          RFLFPLSFGLFNVVYWLYHVY   638
F6XRX3|GABRQ_Canis_lupus_familiaris RILFPLAFVVFNIVYWAYHLN  710
E1BJH4|GABRQ_Bos_taurus            RLLFPLAFVVFNIVYWVYHIY   642
F1Q4Y6|GABRB4_Danio_rerio          RIIFPITFGFFNLIYWLYYVN   512
                                   .......710.......720.
```

```
                                                                   :::    :                                       * :  :..*:**    *.: **::
P24046|GABRR1_Homo_sapiens                    ------------------MLAVPNMRFGIFLLWWGWVLATESRMHWPGREVHEMSKKG-RPQRQRREVHEDAHKQVSPILRRSPDITKSPLTKSEQLLRI   81
A0A2R9BL98|GABRR1_Pan_paniscus                ------------------MLAVPNMRFGIFLLWWGWVLATESRVHWPGREVHEMSKKG-RPQRQRREVHEDAHKQVSPILRRSPDITKSPLTKSEQLLRI   81
H2QTE3|GABRR1_Pan_troglodytes                 ------------------MLAVPNMRFGIFLLWWGWVLATESRVHWPGREVHEMSKKG-RPQRQRREVHEDAHKQVSPILRRSPDITKSPLTKSEQLLRI   81
F6W0N4|GABRR1_Macaca_mulatta                  ------------------MLTVPNMKFGIFLLWWGWVLATESRVHWPGREVHEMSKKGSRPQRQRREVHEDAHKQISPILRRSPDITKSPLTKSEQLLRI   82
P50572|GABRR1_Rattus_norvegicus               ------------------MLAVRNMKFGIFLLWWGWVLAAESTVHWPGREVHEPSKKGSRPQRQRRGAHDDAHKQGSPILRKSSDITKSPLTKSEQLLRI   82
P56475|GABRR1_Mus_musculus                    ------------------MLAVQNMKFGIFLLWWGWVLAAESTAHWPGREVHEPSRKGSRPQRQRRGAHDDAHKQGSPILRRSSDITKSPLTKSEQLLRI   82
M3YD49|GABRR1_Mustela_putorius_furo           ------------------MLAVQNMKVGVFILWWGWVWATESKVH-GRREVHEMSKKG------------------STILKRSPDITKSPLTKSEQLLRI   63
XP_038538749.1|GABRR1_Canis_lupus_familiaris  MGKYNRTRDMRTIWIWKPMLAVQNMKVGVFLLWWGWVLTTEGRQHWQGREAHEMSKKG------------------SPILKRSPDITKSPLTKSEQLLRI   82
Q5TZ16|GABRR1_Danio_rerio                     ------------------MHADV-----CFLLCACFLCT----------GALQSCSKS-RSKRETTRAERDLHKPGSTLLMRSPDVTKAPVTKSEQLLKL   66
Q0II76|GABRR1_Bos_taurus                      ---------------------MPYFS-RLILFLFCLVVLVESR----------------KPKKRRWTGQLETSKP-SHLYKKNPDMTKIRHGKPQPLLRV   61
                                              1.......10........20........30........40........50........60........70........80........90......100

                                              *****:***.*************:****:******************** **:*** .* ****************:***************:*:**** 
P24046|GABRR1_Homo_sapiens                    DDHDFSMRPGFGGPAIPVGVDVQVESLDSISEVDMDFTMTLYLRHYWKDERLSFPSTNNLSMTFDGRLVKKIWVPDMFFVHSKRSFIHDTTTDNVMLRVQ   181
A0A2R9BL98|GABRR1_Pan_paniscus                DDHDFSMRPGFGGPAIPVGVDVQVESLDSISEVDMDFTMTLYLRHYWKDERLSFPSTNNLSMTFDGRLVKKIWVPDMFFVHSKRSFIHDTTTDNVMLRVQ   181
H2QTE3|GABRR1_Pan_troglodytes                 DDHDFSMRPGFGGPAIPVGVDVQVESLDSISEVDMDFTMTLYLRHYWKDERLSFPSTNNLSMTFDGRLVKKIWVPDMFFVHSKRSFIHDTTTDNVMLRVQ   181
F6W0N4|GABRR1_Macaca_mulatta                  DDHDFSMRPGFGGPAIPVGVDVQVESLDSISEVDMDFTMTLYLRHYWKDERLSFPSTNNLSMTFDGRLVKKIWVPDMFFVHSKRSFIHDTTTDNVMLRVQ   182
P50572|GABRR1_Rattus_norvegicus               DDHDFSMRPGFGGPAIPVGVDVQVESLDSISEVDMDFTMTLYLRHYWKDERLSFPSTNNLSMTFDGRLVKKIWVPDMFFVHSKRSFIHDTTTDNVMLRVQ   182
P56475|GABRR1_Mus_musculus                    DDHDFSMRPGFGGPAIPVGVDVQVESLDSISEVDMDFTMTLYLRHYWKDERLSFPSSNNLSMTFDGRLVKKIWVPDMFFVHSKRSFIHDTTTDNVMLRVQ   182
M3YD49|GABRR1_Mustela_putorius_furo           DDHDFSMRPGFGGPAIPVGVDVQVESLDSISEVDMDFTMTLYLRHYWKDERLSFPSTNNLSMTFDGRLVKKIWVPDMFFVHSKRSFIHDTTTDNVMLRVQ   163
XP_038538749.1|GABRR1_Canis_lupus_familiaris  DDHDFSMRPGFGGPAIPVGVDVQVESLDSISEVDMDFTMTLYLRHYWKDERLSFPSTNNLSMTFDGRLVKKIWVPDMFFVHSKRSFIHDTTTDNVMLRVQ   182
Q5TZ16|GABRR1_Danio_rerio                     DDHDFTMRPGFGGPAIPVGVDVQLESLDTISEVDMDFTMTLYLRHYWKDARLSFPSNTNQSMTFDGRLVKKIWVPDIFFVHSKRSFIHDTTTENVMLRVH   166
Q0II76|GABRR1_Bos_taurus                      DDHDFTMRPAFGGPAIPVGVDVQVESLDSISEVDMDFTMTLYLRHYWKDERLAFPSASNKSMTFDGRLVKKIWVPDVFFVHSKRSFIHDTTTDNIMLRVF   161
                                              .......110.......120.......130.......140.......150.......160.......170.......180.......190.......200

                                              *** ****:*:***:*****:*:****:******:******::*******.*:.**:**::*********:****::***********************
P24046|GABRR1_Homo_sapiens                    PDGKVLYSLRVTVTAMCNMDFSRFPLDTQTCSLEIESYAYTEDDLMLYWKKGNDSLKTDERISLSQFLIQEFHTTTKLAFYSSTGWYNRLYINFTLRRHI   281
A0A2R9BL98|GABRR1_Pan_paniscus                PDGKVLYSLRVTVTAMCNMDFSRFPLDTQTCSLEIESYAYTEDDLMLYWKKGNDSLKTDERISLSQFLIQEFHTTTKLAFYSSTGWYNRLYINFTLRRHI   281
H2QTE3|GABRR1_Pan_troglodytes                 PDGKVLYSLRVTVTAMCNMDFSRFPLDTQTCSLEIESYAYTEDDLMLYWKKGNDSLKTDERISLSQFLIQEFHTTTKLAFYSSTGWYNRLYINFTLRRHI   281
F6W0N4|GABRR1_Macaca_mulatta                  PDGKVLYSLRVTVTAMCNMDFSRFPLDTQTCSLEIESYAYTEDDLMLYWKKGNDSLKTDERISLSQFLIQEFHTTTKLAFYSSTGWYNRLYINFTLRRHI   282
P50572|GABRR1_Rattus_norvegicus               PDGKVLYSLRVTVTAMCNMDFSRFPLDTQTCSLEIESYAYTEDDLMLYWKKGNDSLKTDERISLSQFLIQEFHTTTKLAFYSSTGWYNRLYINFTLRRHI   282
P56475|GABRR1_Mus_musculus                    PDGKVLYSLRVTVTAMCNMDFSRFPLDTQTCSLEIESYAYTEDDLMLYWKKGNDSLKTDERISLSQFLIQEFHTTTKLAFYSSTGWYNRLYINFTLRRHI   282
M3YD49|GABRR1_Mustela_putorius_furo           PDGTVLYSLRVTVTAMCNMDFSRFPLDTQTCSLEIESYAYTEDDLMLYWKKGNDSLKTDERISLSQFLIQEFHTTTKLAFYSSTGWYNRLYINFTLRRHI   263
XP_038538749.1|GABRR1_Canis_lupus_familiaris  PDGKVLYSLRVTVTAMCNMDFSRFPLDTQTCSLEIESYAYTEDDLMLYWKKGNDSLKTDERISLSQFLIQEFHTTTKLAFYSSTGWYNRLYINFTLRRHI   282
Q5TZ16|GABRR1_Danio_rerio                     PDGKVLYSLRVTVTSMCNMDLSHFPLDTQTCSLEIESYAYTDDDLMLYWKRGNKSLQTDDKISLSQFLIQEFHTTTKLAFYSSTGWYNRLYINFTLRRHI   266
Q0II76|GABRR1_Bos_taurus                      PDGQVLYSMRITVTAMCNMDFSHFPLDSQTCSLELESYAYTDEDLMLYWKNGDESLKTDEKISLSQFLIQKFHTTSRLAFYSSTGWYNRLYINFTLRRHI   261
                                              .......210.......220.......230.......240.......250.......260.......270.......280.......290.......300

                                              *********************************.****************************:*************:********:*:*****:. ..
P24046|GABRR1_Homo_sapiens                    FFFLLQTYFPATLMVMLSWVSFWIDRRAVPARVPLGITTVLTMSTIITGVNASMPRVSYIKAVDIYLWVSFVFVFLSVLEYAAVNYLTTVQERKEQKLRE   381
A0A2R9BL98|GABRR1_Pan_paniscus                FFFLLQTYFPATLMVMLSWVSFWIDRRAVPARVPLGITTVLTMSTIITGVNASMPRVSYIKAVDVYLWVSFVFVFLSVLEYAAVNYLTTVQERKEQKLRE   381
H2QTE3|GABRR1_Pan_troglodytes                 FFFLLQTYFPATLMVMLSWVSFWIDRRAVPARVPLGITTVLTMSTIITGVNASMPRVSYIKAVDIYLWVSFVFVFLSVLEYAAVNYLTTVQERKEQKLRE   381
F6W0N4|GABRR1_Macaca_mulatta                  FFFLLQTYFPATLMVMLSWVSFWIDRRAVPARVPLGITTVLTMSTIITGVNASMPRVSYIKAVDIYLWVSFVFVFLSVLEYAAVNYLTTVQERKEQKLRE   382
P50572|GABRR1_Rattus_norvegicus               FFFLLQTYFPATLMVMLSWVSFWIDRRAVPARVPLGITTVLTMSTIITGVNASMPRVSYIKAVDIYLWVSFVFVFLSVLEYAAVNYLTTVQERKERKLRE   382
P56475|GABRR1_Mus_musculus                    FFFLLQTYFPATLMVMLSWVSFWIDRRAVPARVPLGITTVLTMSTIITGVNASMPRVSYIKAVDIYLWVSFVFVFLSVLEYAAVNYLTTVQERKERKLRE   382
M3YD49|GABRR1_Mustela_putorius_furo           FFFLLQTYFPATLMVMLSWVSFWIDRRAVPARVPLGITTVLTMSTIITGVNASMPRVSYIKAVDIYLWVSFVFVFLSVLEYAAVNYLTTVQERKERKLRE   363
XP_038538749.1|GABRR1_Canis_lupus_familiaris  FFFLLQTYFPATLMVMLSWVSFWIDRRAVPARVPLGITTVLTMSTIITGVNASMPRVSYIKAVDIYLWVSFVFVFLSVLEYAAVNYLTTVQERKERKLRD   382
Q5TZ16|GABRR1_Danio_rerio                     FFFLLQTYFPATLMVMLSWVSFWIDRRAVPARVPLGITTVLTMSTIITGVNASMPRVSYIKAVDIYLWVSFVFVFLSVIEYAAVNYLSTLQERKERSQNS   366
Q0II76|GABRR1_Bos_taurus                      FFFLLQTYFPATLMVMLSWVSFWIDRRAVPARVSLGITTVLTMSTIITGVNASMPRVSYIKAVDIYLWVSFVFVFLSVLEYAAVNYLTTVQERKERKLQE   361
                                              .......310.......320.......330.......340.......350.......360.......370.......380.......390.......400

                                              : :.* .*:  .   *:...**: :      .*      .*     :  ::::*:*.:  .* .: :        .    :.*   :*********:***.:* :**:
P24046|GABRR1_Homo_sapiens                    K-LPCTSGLPPPRTAMLDGNYSDGEVNDLDNY-----MPE-NGEKPDRMMVQLTLASERSSPQRKSQRSSYVSMRI--DTHAIDKYSRIIFPAAYILFNL   472
A0A2R9BL98|GABRR1_Pan_paniscus                K-LPCTSGLPPPRTVMLDGNYSDGEVNDLDNY-----MPE-NGEKPDRMMVQLTLASERSSPRRKSQRSSYVSMRI--DTHAIDKYSRIIFPAAYILFNL   472
H2QTE3|GABRR1_Pan_troglodytes                 K-LPCTSGLPPPRTVMLDGNYSDGEVNDLDNY-----VPE-NGEKPDRMMVQLTLASERSSPRRKSQRSSYVSMRI--DTHAIDKYSRIIFPAAYILFNL   472
F6W0N4|GABRR1_Macaca_mulatta                  K-LPCTSGLPPPRTVMLDGNYSDGEVNDLDNY-----MPE-NGEKPDRMMVQLTLASERSSPQRKSQRSSYVSMRI--DTHAIDKYSRIIFPAAYILFNL   473
P50572|GABRR1_Rattus_norvegicus               K-ISCTCGLPQPRGVMLDSSYSDGEVNDLGGY-----MPE-NGEKPDRMMVQLTLASERGSPQRKSQRGSYVSMRI--NTHAIDKYSRIIFPAAYILFNL   473
P56475|GABRR1_Mus_musculus                    K-ISCTCGLPQPRGVMLDSSYSDGEVNDLGGY-----MPE-NGEKPDRMMVQLTLASERGSPQRKGQRGSYVSMRI--NTHAIDKYSRIIFPAAYILFNL   473
M3YD49|GABRR1_Mustela_putorius_furo           K-LPCTCGIPQPRGVMLEGSYSDGEVNDLGSY-----IPE-NGEKPDKMMVQLTLASERSSPQRKSQRSSYVSMRI--DTHAIDKYSRIIFPAAYILFNL   454
XP_038538749.1|GABRR1_Canis_lupus_familiaris  K-LPCTCGMPQPRGVMLDGSYSDGEVNDLGNY-----TPE-NGEKPDKMMVQLTLASERSSPQRKSQRSSYVSMRI--DTHAIDKYSRIIFPAAYILFNL   473
Q5TZ16|GABRR1_Danio_rerio                     RQLPCTCGMTHPGQMMMSSSYSEMDMMTTGNYG----MSELSDTKQEQFLVHLVMDNEQGAQRPAVTTSS--TLNI--DTHAIDKYSRVIFPGAYTLFNI   458
Q0II76|GABRR1_Bos_taurus                      K-FPCMCGMLHSRTMMLDGSYSESEANSLAGYPRSHILPE--EERQDKIVVHLALSNESSSSRKKGLLKGQVGLRIFQNTHAIDKYSRLIFPASYIFFNL   458
                                              .......410.......420.......430.......440.......450.......460.......470.......480.......490.......500
```

```
                                                 ****:::
P24046|GABRR1_Homo_sapiens                       IYWSIFS-   479
A0A2R9BL98|GABRR1_Pan_paniscus                   IYWSIFS-   479
H2QTE3|GABRR1_Pan_troglodytes                    IYWSIFS-   479
F6W0N4|GABRR1_Macaca_mulatta                     IYWSIFS-   480
P50572|GABRR1_Rattus_norvegicus                  IYWSIFS-   480
P56475|GABRR1_Mus_musculus                       IYWSIFS-   480
M3YD49|GABRR1_Mustela_putorius_furo              IYWSIFS-   461
XP_038538749.1|GABRR1_Canis_lupus_familiaris     IYWSIFS-   480
Q5TZ16|GABRR1_Danio_rerio                        IYWSIYSQ   466
Q0II76|GABRR1_Bos_taurus                         IYWSVFA-   465
                                                 ........
```

```
                                                              ***  :* :.:: *  : *.*:   .:.:* ** :*  * .  * **  *:** :  *.. **:*::***:****
P28476|GABRR2_Homo_sapiens              -------------------------MPYFTRLILFLFCLMVLVESRK---PKRKRWTGQVEMPKPS-HLYKKNLDVTKIRKGKPQQLLRVDEHDFSMRPA   71
P47742|GABRR2_Rattus_norvegicus         -------------------------MPYFMRLALFLFCLMALVESRK---PRRKRWTGHLETSKPS-HLYKKNLDVTKIRTGKPRPLLRVEDHDFTMRPA   71
P56476|GABRR2_Mus_musculus              -------------------------MPYLMRLALVLFCLMALVESRK---PRRKRWTGLLETSKPS-HLYKKNLDVTKMRPGKPRPLLRVEDHDFTMRPA   71
A0A2R9AXF6|GABRR2_Pan_paniscus          -------------------------MPYFTRLILFLFCLMVLVESRK---PKRKRWTGQVEMPKPS-HLYKKNLDVTKIQKGKPQQLLRVDEHDFSMRPA   71
XP_527448.5|GABRR2_Pan_troglodytes      MVKPGGICSATGYWKAAFCLTDVHKMPYFTRLILFLFCLMVLVESRK---PKRKRWTGQVEMPKPS-HLYKKNLDVTKIQKGKPQRLLRVDEHDFSMRPA   96
XP_001095465.3|GABRR2_Macaca_mulatta    MVKPGAICSATGYWKAAFCLTDIHKMPYFIRLILFLFCLMVLVESRK---PKRKRWTGQVEMPKPS-HLYKKNLDMTKIRKGKPQQLLRVDEHDFSMRPA   96
M3YCV9|GABRR2_Mustela_putorius_furo     TVQPAGACPAAGYWRAVCGIVDVPRMPYFTRLILLLFCLTALVESRK---PKRRRSTGQLEMTKPS-HLYKKNLDMTKIRKGKPQPLLRVEDHDFSMRPA   96
E2R4R0|GABRR2_Canis_lupus_familiaris    -------------------------MPYFTRLILFLFCLTALVESRK---PKRKRWTGQLEMPKPS-HLYKKNLDVTKIRKGKPQPLLRVEDHDFSMRPA   71
F1QX34|GABRR2a_Danio_rerio              ------------------------MMPYARQLLILVLFLGSFGECRNKHATRERRWTGAVETQKHGTSLAKKPPDVTKSRRLKTEQLLKVDDHDFTMRPA   76
Q0II76|GABRR2_Bos_taurus                -------------------------MPYFSRLILFLFCLVVLVESRK---PKKRRWTGQLETSKPS-HLYKKNPDMTKIRHGKPQPLLRVDDHDFTMRPA   71
                                        1.......10........20........30........40........50........60........70........80........90.......100

                                        ***   * * **:.  :.  *.:  ************:****:*.*::*:************************ *****:*********:****:*
P28476|GABRR2_Homo_sapiens              FGGPAIPVGVDVQVESLDSISEVDMDFTMTLYLRHYWKDERLAFSSASNKSMTFDGRLVKKIWVPDVFFVHSKRSFTHDTTTDNIMLRVFPDGHVLYSMR  171
P47742|GABRR2_Rattus_norvegicus         FGGPAIPVGVDVQVESLDSISEVDMDFTMTLYLRHYWRDERLAFPSSSNRSMTFDGRLVKKIWVPDVFFVHSKRSFTHDTTTDNIMLRVFPDGHVLYSMR  171
P56476|GABRR2_Mus_musculus              FGGPAIPVGVDVQVESLDSISEVDMDFTMTLYLRHYWRDERLAFPSSSNKSMTFDGRLVKKIWVPDVFFVHSKRSFIHDTTTDNIMLRVFPDGHVLYSMR  171
A0A2R9AXF6|GABRR2_Pan_paniscus          FGG--WPQGCDVHFCLVS--SRLPQDFTMTLYLRHYWKDERLAFSSASNKSMTFDGRLVKKIWVPDVFFVHSKRSFTHDTTTDNIMLRVFPDGHVLYSMR  167
XP_527448.5|GABRR2_Pan_troglodytes      FGGPAIPVGVDVQVESLDSISEVDMDFTMTLYLRHYWKDERLAFSSASNKSMTFDGRLVKKIWVPDVFFVHSKRSFTHDTTTDNIMLRVFPDGHVLYSMR  196
XP_001095465.3|GABRR2_Macaca_mulatta    FGGPAIPVGVDVQVESLDSISEVDMDFTMTLYLRHYWKDERLAFSSASNKSMTFDGRLVKKIWVPDVFFVHSKRSFTHDTTTDNIMLRVFPDGHVLYSMR  196
M3YCV9|GABRR2_Mustela_putorius_furo     FGGPAIPVGVDVQVESLDSISEVDMDFTMTLYLRHYWKDERLAFPSTSNKSMTFDGRLVKKIWVPDVFFVHSKRSFIHDTTTDNIMLRVFPDGHVLYSMR  196
E2R4R0|GABRR2_Canis_lupus_familiaris    FGGPAIPVGVDVQVESLDSISEVDMDFTMTLYLRHYWKDERLAFPSTSNKSMTFDGRLVKKIWVPDVFFVHSKRSFTHDTTTDNIMLRVFPDGHVLYSMR  171
F1QX34|GABRR2a_Danio_rerio              FGGPAIPVGVDVQVESLDSISEVDMDFTMTLYLRHYWKDERLSFTSSTNKSMTFDGRLVKKIWVPDVFFVHSKRSFIHDTTTENIMLRVFPDGHVLYSLR  176
Q0II76|GABRR2_Bos_taurus                FGGPAIPVGVDVQVESLDSISEVDMDFTMTLYLRHYWKDERLAFPSASNKSMTFDGRLVKKIWVPDVFFVHSKRSFIHDTTTDNIMLRVFPDGQVLYSMR  171
                                        .......110.......120.......130.......140.......150.......160.......170.......180.......190.......200

                                        :**** ******:*********************************. ****************************************************
P28476|GABRR2_Homo_sapiens              ITVTAMCNMDFSHFPLDSQTCSLELESYAYTDEDLMLYWKNGDESLKTDEKISLSQFLIQKFHTTSRLAFYSSTGWYNRLYINFTLRRHIFFFLLQTYFP  271
P47742|GABRR2_Rattus_norvegicus         ITVTAMCNMDFSHFPLDSQTCSLELESYAYTDEDLMLYWKNGDESLKTDEKISLSQFLIQKFHTTSRLAFYSSTGWYNRLYINFTLRRHIFFFLLQTYFP  271
P56476|GABRR2_Mus_musculus              ITVTAMCNMDFSHFPLDSQTCSLELESYAYTDEDLMLYWKNGDESLKTDEKISLSQFLIQKFHTTSRLAFYSSTGWYNRLYINFTLRRHIFFFLLQTYFP  271
A0A2R9AXF6|GABRR2_Pan_paniscus          ITVTAMCNMDFSHFPLDSQTCSLELESYAYTDEDLMLYWKNGDESLKTDEKISLSQFLIQKFHTTSRLAFYSSTGWYNRLYINFTLRRHIFFFLLQTYFP  267
XP_527448.5|GABRR2_Pan_troglodytes      ITVTAMCNMDFSHFPLDSQTCSLELESYAYTDEDLMLYWKNGDESLKTDEKISLSQFLIQKFHTTSRLAFYSSTGWYNRLYINFTLRRHIFFFLLQTYFP  296
XP_001095465.3|GABRR2_Macaca_mulatta    ITVTAMCNMDFSHFPLDSQTCSLELESYAYTDEDLMLYWKNGDESLKTDEKISLSQFLIQKFHTTSRLAFYSSTGWYNRLYINFTLRRHIFFFLLQTYFP  296
M3YCV9|GABRR2_Mustela_putorius_furo     ITVTAMCNMDFSHFPLDSQTCSLELESYAYTDEDLMLYWKNGDESLKTDEKISLSQFLIQKFHTTSRLAFYSSTGWYNRLYINFTLRRHIFFFLLQTYFP  296
E2R4R0|GABRR2_Canis_lupus_familiaris    ITVTAMCNMDFSHFPLDSQTCSLELESYAYTDEDLMLYWKNGDESLKTDEKISLSQFLIQKFHTTSRLAFYSSTGWYNRLYINFTLRRHIFFFLLQTYFP  271
F1QX34|GABRR2a_Danio_rerio              VTVTAACNMDFSRFPLDSQTCSLELESYAYTDEDLMLYWKNGDESLSIDEKISLSQFLIQKFHTTSRLAFYSSTGWYNRLYINFTLRRHIFFFLLQTYFP  276
Q0II76|GABRR2_Bos_taurus                ITVTAMCNMDFSHFPLDSQTCSLELESYAYTDEDLMLYWKNGDESLKTDEKISLSQFLIQKFHTTSRLAFYSSTGWYNRLYINFTLRRHIFFFLLQTYFP  271
                                        .......210.......220.......230.......240.......250.......260.......270.......280.......290.......300

                                        ***************:*********** *****:***************:.*****************************:**::***:::      :*
P28476|GABRR2_Homo_sapiens              ATLMVMLSWVSFWIDRRAVPARVSLGITTVLTMTTIITGVNASMPRVSYVKAVDIYLWVSFVFVFLSVLEYAAVNYLTTVQERKERKLREK------FPC  365
P47742|GABRR2_Rattus_norvegicus         ATLMVMLSWVSFWIDHRAVPARVSLGIMTVLTMSTIITGVNASMPRVSYIRAVDIYLWVSFVFVFLSVLEYAAVNYLTTVQEQKERKLRDK------FPC  365
P56476|GABRR2_Mus_musculus              ATLMVMLSWVSFWIDHRAVPARVSLGIMTVLTMSTIITGVNASMPRVSYIRAVDIYLWVSFVFVFLSVLEYAAVNYLTTLQEQKERKFREK------LPC  365
A0A2R9AXF6|GABRR2_Pan_paniscus          ATLMVMLSWVSFWIDRRAVPARVSLGITTVLTMTTIITGVNASMPRVSYVKAVDIYLWVSFVFVFLSVLEYAAVNYLTTVQERKERKLREK------FPC  361
XP_527448.5|GABRR2_Pan_troglodytes      ATLMVMLSWVSFWIDRRAVPARVSLGITTVLTMTTIITGVNASMPRVSYVKAVDIYLWVSFVFVFLSVLEYAAVNYLTTVQERKERKLREK------FPC  390
XP_001095465.3|GABRR2_Macaca_mulatta    ATLMVMLSWVSFWIDRRAVPARVSLGITTVLTMTTIITGVNASMPRVSYVKAVDIYLWVSFVFVFLSVLEYAAVNYLTTVQERKERKLREK------FPG  390
M3YCV9|GABRR2_Mustela_putorius_furo     ATLMVMLSWVSFWIDRRAVPARVSLGITTVLTMSTIITGVNASMPRVSYIKAVDIYLWVSFVFVFLSVLEYAAVNYLTTVQERKERKLREK------FPC  390
E2R4R0|GABRR2_Canis_lupus_familiaris    ATLMVMLSWVSFWIDRRAVPARVSLGITTVLTMSTIITGVNASMPRVSYIKAVDIYLWVSFVFVFLSVLEYAAVNYLTTVQERKERKLRDK------FPC  365
F1QX34|GABRR2a_Danio_rerio              ATLMVMLSWVSFWIDRRAVPARVSLGITTVLTMSTIITGVNASMPRVSYIKAVDIYLWVSFVFVFLSVLEYAAVNYLTTVQERRERKLRDRAVREQSLPC  376
Q0II76|GABRR2_Bos_taurus                ATLMVMLSWVSFWIDRRAVPARVSLGITTVLTMSTIITGVNASMPRVSYIKAVDIYLWVSFVFVFLSVLEYAAVNYLTTVQERKERKLQEK------FPC  365
                                        .......310.......320.......330.......340.......350.......360.......370.......380.......390.......400

                                         .**  ::** ***:***:::******..: :: **   *: :: ***:.:.: . ::***:      .: *:***********:***. **.*******
P28476|GABRR2_Homo_sapiens              MCGMLHSKTMMLDGSYSESEANSLAGYPRSHILTEE---ERQDKIVVHLGLSGEANAARKKGLLKGQTGFRIFQNTHAIDKYSRLIFPASYIFFNLIYWS  462
P47742|GABRR2_Rattus_norvegicus         TCGMLHSRTMTLDGSYSESEANSLAGYPRSHILPEE---ERQDKIVVHLALNSELTSSRKKGLLKGQMGLYIFQNTHAIDKYSRLIFPAFYIVFNLIYWS  462
P56476|GABRR2_Mus_musculus              MCGMLHSRTMMLDGSYSESEANSLAGYPRSHILPEE---ERPDNIVVHLALNSELTSSRKKGLLKGQMGLYIFQNTHAIDKYSRLIFPAFYIVFNLIYWS  462
A0A2R9AXF6|GABRR2_Pan_paniscus          MCGMLHSKTMMLDGSYSESEANSLAGYPRSHILTEE---ERQDKIVVHLGLSGEANAARKKGLLKGQTGFRIFQNTHAIDKYSRLIFPASYIFFNLIYWS  458
XP_527448.5|GABRR2_Pan_troglodytes      MCGMLHSKTMMLDGSYSESEANSLAGYPRSHILTEE---ERQDKIVVHLGLSGEANAARKKGLLKGQTGFRIFQNTHAIDKYSRLIFPASYIFFNLIYWS  487
XP_001095465.3|GABRR2_Macaca_mulatta    MCGMLHSKTMMLDGSYSESEANSLAGYPRSHILMEE---ERQDKTVVHLGLSGDANAARKKGLLKGQMGFRIFQNTHAIDKYSRLIFPASYIFFNLIYWS  487
M3YCV9|GABRR2_Mustela_putorius_furo     MSGMLPSRTMMLDGSYSESEANSLAGYPRSHILTEE---ERQDKIVVHVALSNESNSSRKKGFLKGHVGLRIFQNTHAIDKYSRLIFPASYIFFNLIYWS  487
E2R4R0|GABRR2_Canis_lupus_familiaris    MCGMLHSRTMMLDGSYSESEANSLAGYPRSHILTEE---ERQDKIVVHLALSNDSNSSRKKGLLRGHMGLRIFQNTHAIDKYSRLIFPASYIFFNLIYWS  462
F1QX34|GABRR2a_Danio_rerio              TCGMSHTRTMMLDGTYSEADTNSLAGYTEAPMVHEEVVPEKQEHMVVHLSVSSESTNTKKKGI----RALRIMQNTHAIDKYSRMIFPGAYIIFNLIYWS  472
Q0II76|GABRR2_Bos_taurus                MCGMLHSRTMMLDGSYSESEANSLAGYPRSHILPEE---ERQDKIVVHLALSNESSSSRKKGLLKGQVGLRIFQNTHAIDKYSRLIFPASYIFFNLIYWS  462
                                        .......410.......420.......430.......440.......450.......460.......470.......480.......490.......500
```

```
                                                    *:.
P28476|GABRR2_Homo_sapiens                          VFS    465
P47742|GABRR2_Rattus_norvegicus                     VFS    465
P56476|GABRR2_Mus_musculus                          VFS    465
A0A2R9AXF6|GABRR2_Pan_paniscus                      VFS    461
XP_527448.5|GABRR2_Pan_troglodytes                  VFS    490
XP_001095465.3|GABRR2_Macaca_mulatta                VFS    490
M3YCV9|GABRR2_Mustela_putorius_furo                 VFS    490
E2R4R0|GABRR2_Canis_lupus_familiaris                VFS    465
F1QX34|GABRR2a_Danio_rerio                          VYC    475
Q0II76|GABRR2_Bos_taurus                            VFA    465
                                                    ...
```

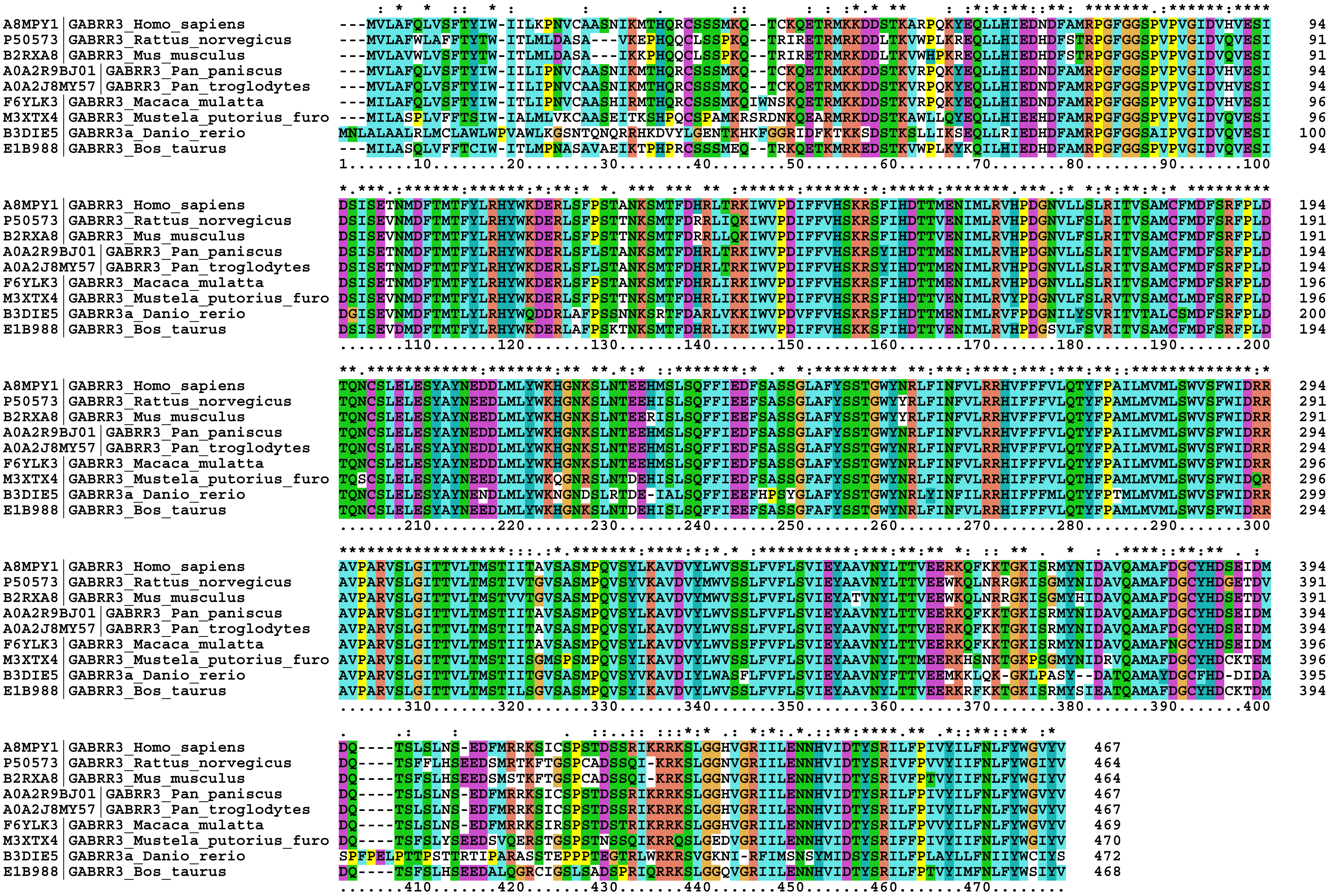
A8MPY1|GABRR3_Homo_sapiens
P50573|GABRR3_Rattus_norvegicus
B2RXA8|GABRR3_Mus_musculus
A0A2R9BJ01|GABRR3_Pan_paniscus
A0A2J8MY57|GABRR3_Pan_troglodytes
F6YLK3|GABRR3_Macaca_mulatta
M3XTX4|GABRR3_Mustela_putorius_furo
B3DIE5|GABRR3a_Danio_rerio
E1B988|GABRR3_Bos_taurus

| # | Name | SMILES |
|---|---|---|
| 1 | ACAMPROSATE | S(=O)(=O)(O)CCCNC(=O)C |
| 2 | ALPRAZOLAM | Clc4cc2c(n1c(nnc1C)CN=C2c3ccccc3)cc4 |
| 3 | AMOBARBITAL | O=C1NC(=O)C(C(=O)N1)(CCC(C)C)CC |
| 4 | AMOXAPINE | Clc4cc2c(Oc3c(N=C2N1CCNCC1)cccc3)cc4 |
| 5 | APALUTAMIDE | S=C3N(c1cnc(c(c1)C(F)(F)F)C#N)C(=O)C4(N3c2cc(F)c(cc2)C(=O)NC)CCC4 |
| 6 | BARBITAL | O=C1NC(=O)C(C(=O)N1)(CC)CC |
| 7 | BENTAZEPAM | s2c1NC(=O)CN=C(c1c3c2CCCC3)c4ccccc4 |
| 8 | BREXANOLONE | O=C([C@@H]4[C@@]3([C@H]([C@H]2[C@@H]([C@@]1([C@H](C[C@H](O)CC1)CC2)C)CC3)CC4)C)C |
| 9 | BROMAZEPAM | Brc3cc2c(NC(=O)CN=C2c1ncccc1)cc3 |
| 10 | BUTALBITAL | O=C1NC(=O)C(C(=O)N1)(CC=C)CC(C)C |
| 11 | BUTOBARBITAL | O=C1NC(=O)C(C(=O)N1)(CCCC)CC |
| 12 | CARISOPRODOL | O=C(OCC(COC(=O)N)(CCC)C)NC(C)C |
| 13 | CHLORDESMETHYLDIAZEPAM | Clc1c(cccc1)C3=NCC(=O)Nc2c3cc(Cl)cc2 |
| 14 | CHLORDIAZEPOXIDE | Clc3cc1c(N=C(NC)CN(=O)=C1c2ccccc2)cc3 |
| 15 | CINOLAZEPAM | Clc3cc2c(N(C(=O)C(O)N=C2c1c(F)cccc1)CCC#N)cc3 |
| 16 | CLOBAZAM | Clc3cc2N(c1ccccc1)C(=O)CC(=O)N(c2cc3)C |
| 17 | CLONAZEPAM | Clc1c(cccc1)C3=NCC(=O)Nc2c3cc([N+](=O)[O-])cc2 |
| 18 | CLORAZEPATE | Clc3cc1c(NC(=O)C(N=C1c2ccccc2)C(=O)O)cc3 |
| 19 | CLOTIAZEPAM | Clc1c(cccc1)C3=NCC(=O)N(c2sc(cc23)CC)C |
| 20 | DESFLURANE | FC(F)(F)C(F)OC(F)F |
| 21 | DIAZEPAM | Clc3cc1c(N(C(=O)CN=C1c2ccccc2)C)cc3 |
| 22 | ENFLURANE | ClC(F)C(F)(F)OC(F)F |
| 23 | ESTAZOLAM | Clc4cc2c(n1c(nnc1)CN=C2c3ccccc3)cc4 |
| 24 | ESZOPICLONE | Clc4cnc(N3C(=O)c1nccnc1[C@@H]3OC(=O)N2CCN(CC2)C)cc4 |
| 25 | ETHANOL | OCC |
| 26 | ETHCHLORVYNOL | Cl¥C=C¥C(O)(C#C)CC |
| 27 | ETIZOLAM | Clc1c(cccc1)C3=NCc4nnc(n4c2sc(cc23)CC)C |
| 28 | ETOMIDATE | O=C(OCC)c1n(cnc1)[C@@H](c2ccccc2)C |
| 29 | FLUDIAZEPAM | Clc3cc2c(N(C(=O)CN=C2c1c(F)cccc1)C)cc3 |
| 30 | FLUMAZENIL | Fc3cc2c(n1cnc(c1CN(C2=O)C)C(=O)OCC)cc3 |
| 31 | FLUNITRAZEPAM | Fc1c(cccc1)C3=NCC(=O)N(c2c3cc([N+](=O)[O-])cc2)C |
| 32 | FLURAZEPAM | Clc3cc2c(N(C(=O)CN=C2c1c(F)cccc1)CCN(CC)CC)cc3 |
| 33 | FLUTAZOLAM | Clc4cc3c(N(C(=O)CN1CCOC13c2c(F)cccc2)CCO)cc4 |
| 34 | FOSPROPOFOL | P(=O)(OCOc1c(cccc1C(C)C)C(C)C)(O)O |
| 35 | GLUTETHIMIDE | O=C1NC(=O)CCC1(c2ccccc2)CC |
| 36 | HALAZEPAM | Clc3cc1c(N(C(=O)CN=C1c2ccccc2)CC(F)(F)F)cc3 |
| 37 | HALOTHANE | BrC(Cl)C(F)(F)F |
| 38 | HEXOBARBITAL | O=C1N(C(=O)C(C(=O)N1)(C2=CCCCC2)C)C |
| 39 | ISOFLURANE | ClC(OC(F)F)C(F)(F)F |
| 40a | IVERMECTIN H2B1a | O=C6O[C@@H]2C[C@@]1(O[C@@H]([C@H](CC1)C)[C@@H](CC)C)O[C@@H](C2)CC=C([C@@H](O[C@@H]4O[C@H]([C@H](O[C@@H]3O[C@H]([C@H](O)[C@@H](OC)C3)C)[C@@H](OC)C4)C)[C@H](C=CC=C5[C@]7(O)[C@H](OC5)[C@H](O)C(=C[C@@H]67)C)C)C |
| 40b | IVERMECTIN H2B1b | O=C6O[C@@H]2C[C@@]1(O[C@@H]([C@H](CC1)C)C(C)C)O[C@@H](C2)CC=C([C@@H](O[C@@H]4O[C@H]([C@H](O[C@@H]3O[C@H]([C@H](O)[C@@H](OC)C3)C)[C@@H](OC)C4)C)[C@H](C=CC=C5[C@]7(O)[C@H](OC5)[C@H](O)C(=C[C@@H]67)C)C)C |
| 41 | KETAZOLAM | Clc4cc2c(N(C(=O)CN1C(=O)C=C(OC12c3ccccc3)C)C)cc4 |
| 42 | LAMOTRIGINE | Clc1c(Cl)cccc1c2nnc(nc2N)N |
| 43 | LORAZEPAM | Clc1c(cccc1)C3=NC(O)C(=O)Nc2c3cc(Cl)cc2 |
| 44 | LORMETAZEPAM | Clc1c(cccc1)C3=NC(O)C(=O)Nc2c3cc(Cl)cc2 |
| 45 | MEDAZEPAM | Clc3cc1c(N(CCN=C1c2ccccc2)C)cc3 |
| 46 | MEDROXYPROGESTERONE ACETATE | O=C(O[C@@]4(C(=O)C)[C@@]3([C@H]([C@H]2[C@@H]([C@@]1(C(=CC(=O)CC1)[C@H](C2)C)C)CC3)CC4)C)C |
| 47 | MEMANTINE | NC12C[C@]3(C[C@@](C1)(CC(C2)C3)C)C |

| | | |
|---|---|---|
| 48 | MEPROBAMATE | O=C(OCC(COC(=O)N)(CCC)C)N |
| 49 | METHARBITAL | O=C1N(C(=O)C(C(=O)N1)(CC)CC)C |
| 50 | METHOHEXITAL | O=C1N(C(=O)C(C(=O)N1)(C(C#CCC)C)CC=C)C |
| 51 | METHOXYFLURANE | ClC(Cl)C(F)(F)OC |
| 52 | METHYPRYLON | O=C1NCC(C(=O)C1(CC)CC)C |
| 53 | MEXAZOLAM | Clc1c(cccc1)C34OCC(N4CC(O)=Nc2c3cc(Cl)cc2)C |
| 54 | MIDAZOLAM | Clc4cc3c(n1c(ncc1CN=C3c2c(F)cccc2)C)cc4 |
| 55 | NIMETAZEPAM | O=[N+]([O-])c3cc1c(N(C(=O)CN=C1c2ccccc2)C)cc3 |
| 56 | NITRAZEPAM | O=[N+]([O-])c3cc1c(NC(=O)CN=C1c2ccccc2)cc3 |
| 57 | NORDAZEPAM | Clc3cc1c(NC(=O)CN=C1c2ccccc2)cc3 |
| 58 | OLANZAPINE | s3c2Nc4c(N=C(N1CCN(CC1)C)c2cc3C)cccc4 |
| 59 | OXAZEPAM | Clc3cc1c(NC(=O)C(O)N=C1c2ccccc2)cc3 |
| 60 | PENTOBARBITAL | O=C1NC(=O)C(C(=O)N1)(C(CCC)C)CC |
| 61 | PHENOBARBITAL | O=C2NC(=O)C(c1ccccc1)(C(=O)N2)CC |
| 62 | PHENYTOIN | O=C1NC(=O)C(N1)(c2ccccc2)c3ccccc3 |
| 63 | PIPERAZINE | N1CCNCC1 |
| 64 | PRAZEPAM | Clc4cc1c(N(C(=O)CN=C1c2ccccc2)CC3CC3)cc4 |
| 65 | PRIMIDONE | O=C2NCNC(=O)C2(c1ccccc1)CC |
| 66 | PROPOFOL | Oc1c(cccc1C(C)C)C(C)C |
| 67 | QUAZEPAM | Clc3cc2c(N(C(=S)CN=C2c1c(F)cccc1)CC(F)(F)F)cc3 |
| 68 | RILMAZAFONE | Clc1c(cccc1)C(=O)c3c(n2nc(nc2CNC(=O)CN)C(=O)N(C)C)ccc(Cl)c3 |
| 69 | SECOBARBITAL | O=C1NC(=O)C(C(=O)N1)(C(CCC)C)CC=C |
| 70 | SEVOFLURANE | FC(F)(F)C(OCF)C(F)(F)F |
| 71 | STIRIPENTOL | O1c2c(OC1)ccc(c2)¥C=C¥C(O)C(C)(C)C |
| 72 | TEMAZEPAM | Clc3cc1c(N(C(=O)C(O)N=C1c2ccccc2)C)cc3 |
| 73 | THIAMYLAL | S=C1NC(=O)C(C(=O)N1)(C(CCC)C)CC=C |
| 74 | THIOCOLCHICOSIDE | S(c4c(=O)cc3c(c2c(OC)c(OC)c(O[C@@H]1O[C@@H]([C@@H](O)[C@H](O)[C@H]1O)CO)cc2CC[C@@H]3NC(=O)C)cc4)C |
| 75 | THIOPENTAL | S=C1NC(=O)C(C(=O)N1)(C(CCC)C)CC |
| 76 | TOPIRAMATE | S(=O)(=O)(OC[C@]13OC(O[C@H]1[C@@H]2OC(O[C@@H]2CO3)(C)C)(C)C)N |
| 77 | TRIAZOLAM | Clc1c(cccc1)C4=NCc2nnc(n2c3c4cc(Cl)cc3)C |
| 78 | TRIAZULENONE | Clc1c(cccc1)C3=NCC4N(c2c3cc(N(=O)=O)cc2)C(=O)/C(/N=4)=C/N5CCN(CC5)C |
| 79 | ZALEPLON | O=C(N(c3cc(c2n1ncc(c1ncc2)C#N)ccc3)CC)C |
| 80 | ZOLPIDEM | O=C(N(C)C)Cc1n3c(nc1c2ccc(cc2)C)ccc(c3)C |
| 81 | ZOPICLONE | Clc4cnc(N3C(=O)c1nccnc1C3OC(=O)N2CCN(CC2)C)cc4 |

**Supplementary Table S3:** All drugs selected in this study and their respective SMILES codes.

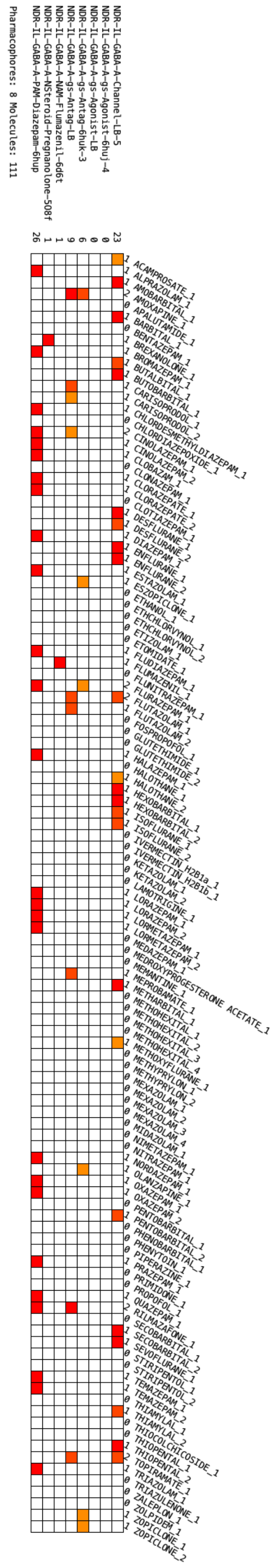

**Supplementary Figure S9:** Results from *in silico* profiling of 81 Drugs using selected GABAA models in the NeuroDeRisk IL Profiler V1.0.

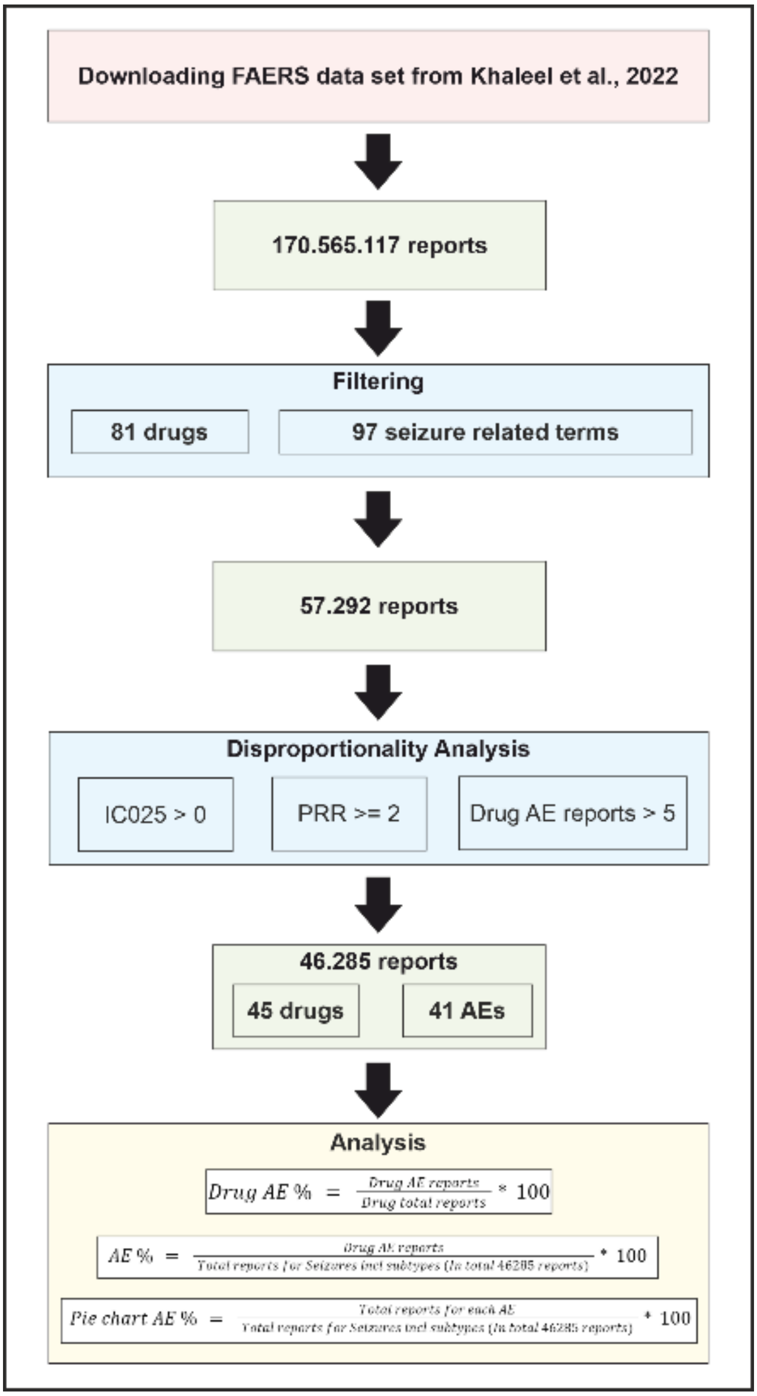


**Supplementary Figure S10. Workflow of the FAERS analysis.** Greenish boxes contain the results of the filtering processes, blue boxes describe the filtering process which was used for the FAERS data set obtained from Khaleel et al., 2022 (Khaleel *et al.*, 2022).

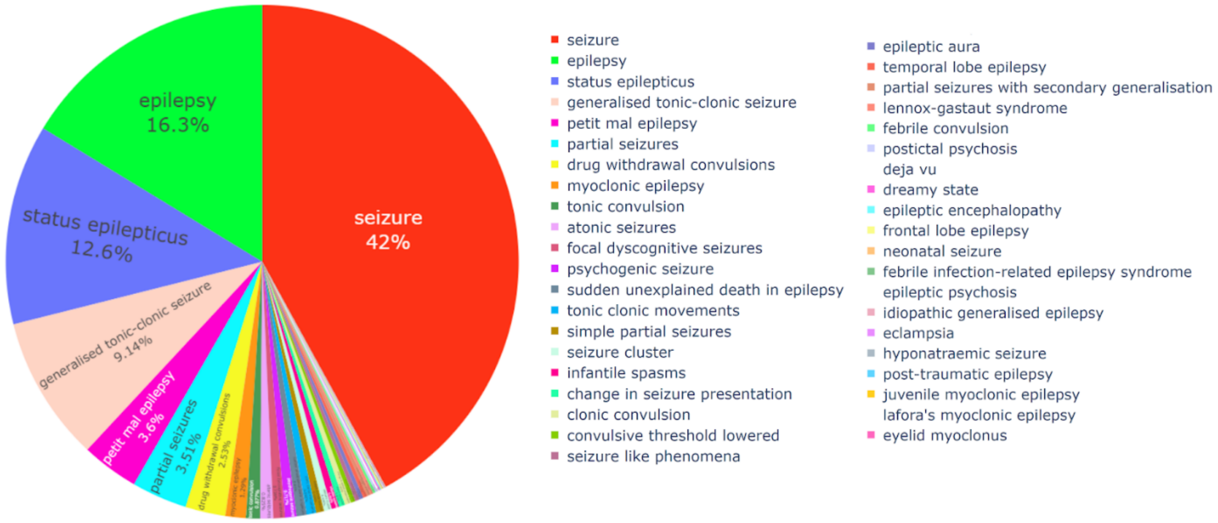


**Supplementary Figure S11:** Distribution of all seizure related reports from our analysis which are in total 46285 reports.

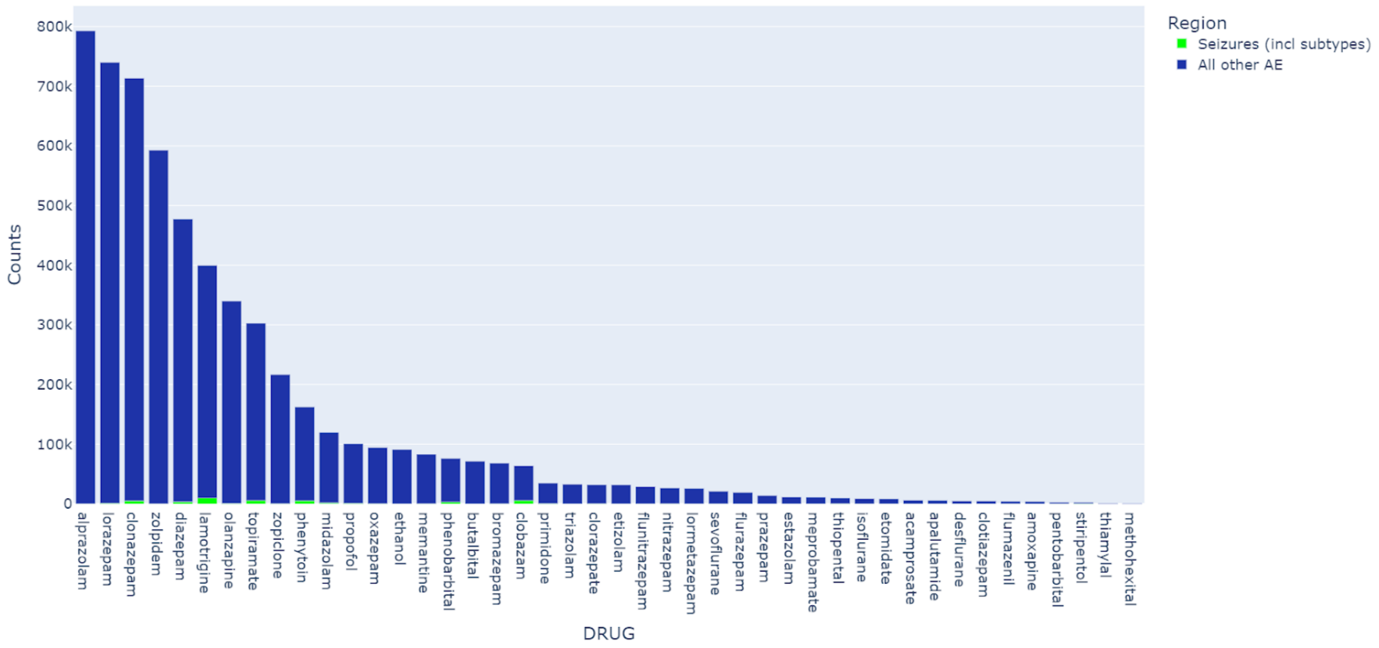


**Supplementary Figure S12.** Total report number for all drugs used, which are displayed in Figure 4. All seizure-category reports are displayed in green, all other adverse event terms as a lump sum in blue.

| Drug | Total reports | AE | AE reports |
|---|---|---|---|
| **ACAMPROSATE** | 587 | SEIZURE | 17 |
| **ALPRAZOLAM** | 30031 | SEIZURE | 410 |
| | | EPILEPSY | 71 |
| | | STATUS EPILEPTICUS | 44 |
| | | DRUG WITHDRAWAL CONVULSIONS | 29 |
| **AMOXAPINE** | 134 | SEIZURE | 9 |
| **BROMAZEPAM** | 3047 | SEIZURE | 26 |
| | | STATUS EPILEPTICUS | 14 |
| | | EPILEPSY | 13 |
| **CARISOPRODOL** | 3493 | SEIZURE | 56 |
| **CHLORDIAZEPOXIDE** | 756 | SEIZURE | 14 |
| **CLOBAZAM** | 2070 | SEIZURE | 325 |
| | | DRUG WITHDRAWAL CONVULSIONS | 76 |
| | | EPILEPSY | 55 |
| | | STATUS EPILEPTICUS | 51 |
| | | PARTIAL SEIZURES | 19 |
| | | FOCAL DYSCOGNITIVE SEIZURES | 12 |
| | | PETIT MAL EPILEPSY | 11 |
| **CLONAZEPAM** | 18338 | SEIZURE | 498 |
| | | STATUS EPILEPTICUS | 66 |
| | | EPILEPSY | 59 |
| | | DRUG WITHDRAWAL CONVULSIONS | 29 |
| | | PARTIAL SEIZURES | 18 |
| | | PETIT MAL EPILEPSY | 18 |
| **CLORAZEPATE DIPOTASSIUM** | 1205 | SEIZURE | 16 |
| **DESFLURANE** | 509 | SEIZURE LIKE PHENOMENA | 12 |
| **DIAZEPAM** | 20293 | SEIZURE | 345 |
| | | STATUS EPILEPTICUS | 88 |
| | | DRUG WITHDRAWAL CONVULSIONS | 69 |
| | | EPILEPSY | 58 |
| | | PARTIAL SEIZURES | 12 |
| | | PETIT MAL EPILEPSY | 9 |
| **ESTAZOLAM** | 263 | SEIZURE | 16 |

| | | | |
|---|---|---|---|
| **ESZOPICLONE** | 7811 | SEIZURE | 22 |
| **ETHANOL** | 13742 | SEIZURE | 164 |
| | | STATUS EPILEPTICUS | 14 |
| | | EPILEPSY | 14 |
| | | PARTIAL SEIZURES | 12 |
| **ETIZOLAM** | 630 | SEIZURE | 13 |
| **FLUMAZENIL** | 248 | SEIZURE | 51 |
| | | DRUG WITHDRAWAL CONVULSIONS | 42 |
| **FLUNITRAZEPAM** | 875 | SEIZURE | 28 |
| **ISOFLURANE** | 502 | STATUS EPILEPTICUS | 33 |
| **IVERMECTIN** | 1764 | SEIZURE | 21 |
| **LAMOTRIGINE** | 26598 | SEIZURE | 1723 |
| | | EPILEPSY | 228 |
| | | STATUS EPILEPTICUS | 189 |
| | | PETIT MAL EPILEPSY | 140 |
| | | PARTIAL SEIZURES | 76 |
| | | FOCAL DYSCOGNITIVE SEIZURES | 54 |
| | | SUDDEN UNEXPLAINED DEATH IN EPILEPSY | 41 |
| | | MYOCLONIC EPILEPSY | 33 |
| **LORAZEPAM** | 16200 | SEIZURE | 291 |
| | | STATUS EPILEPTICUS | 79 |
| | | EPILEPSY | 76 |
| | | DRUG WITHDRAWAL CONVULSIONS | 27 |
| | | PETIT MAL EPILEPSY | 10 |
| | | PARTIAL SEIZURES | 9 |
| **LORMETAZEPAM** | 1347 | EPILEPSY | 14 |
| | | SEIZURE | 8 |
| **MEDROXYPROGESTERONE ACETATE** | 10874 | SEIZURE | 34 |
| | | EPILEPSY | 12 |
| **MEMANTINE** | 6510 | SEIZURE | 190 |
| | | EPILEPSY | 61 |
| | | PETIT MAL EPILEPSY | 16 |
| | | PARTIAL SEIZURES | 12 |
| | | STATUS EPILEPTICUS | 8 |
| **MEPROBAMATE** | 781 | SEIZURE | 10 |

| | | | |
|---|---|---|---|
| **MIDAZOLAM** | 4298 | SEIZURE | 123 |
| | | STATUS EPILEPTICUS | 57 |
| | | EPILEPSY | 21 |
| | | SEIZURE LIKE PHENOMENA | 15 |
| | | FOCAL DYSCOGNITIVE SEIZURES | 13 |
| | | PETIT MAL EPILEPSY | 13 |
| | | PARTIAL SEIZURES | 12 |
| **NITRAZEPAM** | 627 | SEIZURE | 13 |
| **OLANZAPINE** | 28785 | SEIZURE | 554 |
| | | EPILEPSY | 137 |
| | | STATUS EPILEPTICUS | 42 |
| | | PARTIAL SEIZURES | 18 |
| | | PETIT MAL EPILEPSY | 17 |
| **OXAZEPAM** | 4627 | SEIZURE | 53 |
| | | STATUS EPILEPTICUS | 17 |
| | | EPILEPSY | 14 |
| **PHENOBARBITAL** | 2287 | SEIZURE | 143 |
| | | STATUS EPILEPTICUS | 80 |
| | | EPILEPSY | 35 |
| | | PARTIAL SEIZURES | 15 |
| | | MYOCLONIC EPILEPSY | 8 |
| **PHENYTOIN** | 10126 | SEIZURE | 972 |
| | | STATUS EPILEPTICUS | 204 |
| | | EPILEPSY | 100 |
| | | PARTIAL SEIZURES | 59 |
| | | PETIT MAL EPILEPSY | 42 |
| | | FOCAL DYSCOGNITIVE SEIZURES | 23 |
| | | SUDDEN UNEXPLAINED DEATH IN EPILEPSY | 13 |
| **PRIMIDONE** | 711 | SEIZURE | 51 |
| | | EPILEPSY | 17 |
| | | PETIT MAL EPILEPSY | 14 |
| | | MYOCLONIC EPILEPSY | 11 |
| **PROPOFOL** | 7869 | SEIZURE | 165 |
| | | STATUS EPILEPTICUS | 70 |
| | | EPILEPSY | 27 |
| | | SEIZURE LIKE PHENOMENA | 25 |

| | | | |
|---|---|---|---|
| | | MYOCLONIC EPILEPSY | 12 |
| SEVOFLURANE | 1650 | SEIZURE | 23 |
| | 1650 | SEIZURE LIKE PHENOMENA | 11 |
| TEMAZEPAM | 3519 | SEIZURE | 46 |
| THIOPENTAL | 530 | STATUS EPILEPTICUS | 30 |
| | 530 | SEIZURE | 20 |
| TOPIRAMATE | 11307 | SEIZURE | 562 |
| | 11307 | EPILEPSY | 85 |
| | 11307 | STATUS EPILEPTICUS | 82 |
| | 11307 | PETIT MAL EPILEPSY | 59 |
| | 11307 | FOCAL DYSCOGNITIVE SEIZURES | 31 |
| | 11307 | PARTIAL SEIZURES | 30 |
| | 11307 | DRUG WITHDRAWAL CONVULSIONS | 21 |
| | 11307 | MYOCLONIC EPILEPSY | 21 |
| | 11307 | SUDDEN UNEXPLAINED DEATH IN EPILEPSY | 16 |
| | 11307 | SEIZURE LIKE PHENOMENA | 9 |
| TRIAZOLAM | 1431 | SEIZURE | 12 |
| ZOLPIDEM | 18040 | SEIZURE | 248 |
| | | DRUG WITHDRAWAL CONVULSIONS | 39 |
| | | EPILEPSY | 38 |
| | | STATUS EPILEPTICUS | 22 |
| ZOPICLONE | 5399 | SEIZURE | 70 |
| | | EPILEPSY | 28 |
| | | STATUS EPILEPTICUS | 12 |

**Supplementary Table S4:** Showing all total reports and the reports per AE per drug for the selected drugs and AEs.

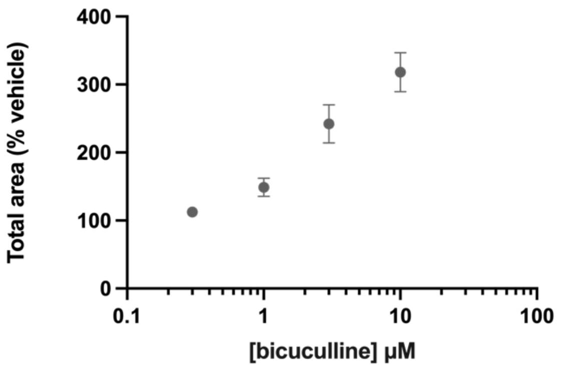


**Supplementary Figure S13:** Mean (± SEM) concentration-response data summary of bicuculline (0.3, 1, 3, and 10 µM) effects on CA1 population spike area in rat hippocampal brain slices. Statistical testing was run on raw concentration data (dose: F(4, 27)=57.10 p<.001; Dunnett's posthoc: bicuculline 1, 3, and 10 µM vs veh, p<.001).

| BEHAVIORAL ALTERATIONS | | |
|---|---|---|
| **Amoxapine (mM)** | **Occurrence** | **%** |
| 0 | 0/8 | 0% |
| 0.003 | 0/8 | 0% |
| 0.01 | 0/8 | 0% |
| 0.03 | 3/8 | 38% |
| 0.1 | 8/8 | 100% |
| 0.3 | 8/8 | 100% |

| MORTALITY | | |
|---|---|---|
| **Amoxapine (mM)** | **Occurrence** | **%** |
| 0 | 0/8 | 0% |
| 0.003 | 0/8 | 0% |
| 0.01 | 0/8 | 0% |
| 0.03 | 0/8 | 0% |
| 0.1 | 0/8 | 0% |
| 0.3 | 2/8 | 25% |

**Supplementary Table S5:** Behavioral alterations (abnormal swimming behaviour, like circling or erratic movements) and mortality of zebrafish larvae.

| | 1.58µM | 5µM | 15.8µM | 50µM |
|---|---|---|---|---|
| Firing Rate | **146** | **173** | **145** | 76 |
| Median Burst Rate | 104 | 127 | **160** | **158** |
| Median Num of Spikes in Burst | **296** | **303** | 183 | 92 |
| Percent Isolated Spikes | 72 | **14** | **9** | **35** |
| ISI CV | 135 | **170** | 129 | 92 |
| Normalized IQR Burst Duration | 73 | **27** | **42** | **49** |
| Median Burst Duration | **145** | **124** | 71 | 57 |
| Mean Interburst Interval | 97 | 79 | **65** | **66** |
| Mean of ISI-distance | 75 | **41** | **36** | **39** |
| Normalized MAD Burst Spike | **59** | **22** | **25** | **38** |
| Median/Mean ISI | 101 | 76 | 70 | 89 |
| Median ISI | **67** | **32** | **37** | 76 |

**Supplementary Table S6:** Heatmap of bicuculline from MEA recordings from rat cortical neurons showing % changes versus vehicle in each parameter. Green color indicates an increase in a parameter, red color indicates a decrease. A value of 100% corresponds to no change from vehicle (pure white color). The intensity of the color in a box increases with the magnitude of the change. Statistically significant values compared to vehicle are indicated in bold.

| Subunit | Species | Sequence ID | Database | Date accessed |
|---|---|---|---|---|
| GABRA1 | Homo sapiens | P14867 | UniProt | 11.04.2022 |
| GABRA1 | Pan paniscus | A0A2R9AU65 | UniProt | 11.04.2022 |
| GABRA1 | Mustela putorius furo | M3YP62 | UniProt | 11.04.2022 |
| GABRA1 | Rattus norvegicus | P62813 | UniProt | 11.04.2022 |
| GABRA1 | Mus musculus | P62812 | UniProt | 11.04.2022 |
| GABRA1 | Canis lupus familiaris | E2RSP8 | UniProt | 11.04.2022 |
| GABRA1 | Pan troglodytes | A0A2I3SDV7 | UniProt | 11.04.2022 |
| GABRA1 | Danio rerio | Q08BJ3 | UniProt | 11.04.2022 |
| GABRA1 | Macaca mulatta | A0A1D5Q406 | UniProt | 11.04.2022 |
| GABRA1 | Bos taurus | P08219 | UniProt | 11.04.2022 |
| GABRA2 | Homo sapiens | P47869 | UniProt | 11.04.2022 |
| GABRA2 | Pan paniscus | XP_003816076.1 | NCBI Protein | 11.04.2022 |
| GABRA2 | Mustela putorius furo | M3Y0V9 | UniProt | 11.04.2022 |
| GABRA2 | Rattus norvegicus | P23576 | UniProt | 11.04.2022 |
| GABRA2 | Mus musculus | P26048 | UniProt | 11.04.2022 |

| | | | | |
|---|---|---|---|---|
| GABRA2 | Canis lupus familiaris | A0A5F4CYJ7 | UniProt | 11.04.2022 |
| GABRA2 | Pan troglodytes | H2QPE5 | UniProt | 11.04.2022 |
| GABRA2a | Danio rerio | E7F635 | UniProt | 11.04.2022 |
| GABRA2 | Macaca mulatta | XP_028703913.1 | NCBI Protein | 11.04.2022 |
| GABRA2 | Bos taurus | P10063 | UniProt | 11.04.2022 |

| | | | | |
|---|---|---|---|---|
| GABRA3 | Homo sapiens | P34903 | UniProt | 11.04.2022 |
| GABRA3 | Pan paniscus | A0A2R9AHX2 | UniProt | 11.04.2022 |
| GABRA3 | Mustela putorius furo | M3YJX4 | UniProt | 11.04.2022 |
| GABRA3 | Rattus norvegicus | P20236 | UniProt | 11.04.2022 |
| GABRA3 | Mus musculus | P26049 | UniProt | 11.04.2022 |
| GABRA3 | Canis lupus familiaris | E2QVJ7 | UniProt | 11.04.2022 |
| GABRA3 | Pan troglodytes | H2QZ88 | UniProt | 11.04.2022 |
| GABRA3 | Danio rerio | A0A2R8QC15 | UniProt | 11.04.2022 |
| GABRA3 | Macaca mulatta | F7G8R5 | UniProt | 11.04.2022 |
| GABRA3 | Bos taurus | P10064 | UniProt | 11.04.2022 |

| | | | | |
|---|---|---|---|---|
| **GABRA4** | **Homo sapiens** | **P48169** | **UniProt** | **11.04.2022** |
| **GABRA4** | **Pan paniscus** | **A0A2R9C6M3** | **UniProt** | **11.04.2022** |
| **GABRA4** | **Mustela putorius furo** | **M3Y0X5** | **UniProt** | **11.04.2022** |
| **GABRA4** | **Rattus norvegicus** | **P28471** | **UniProt** | **11.04.2022** |
| **GABRA4** | **Mus musculus** | **Q9D6F4** | **UniProt** | **11.04.2022** |
| **GABRA4** | **Canis lupus familiaris** | **F1P9P9** | **UniProt** | **11.04.2022** |
| **GABRA4** | **Pan troglodytes** | **H2QPE7** | **UniProt** | **11.04.2022** |
| **GABRA4** | **Danio rerio** | **Q568M9** | **UniProt** | **11.04.2022** |
| **GABRA4** | **Macaca mulatta** | **F6YLN4** | **UniProt** | **11.04.2022** |
| **GABRA4** | **Bos taurus** | **P20237** | **UniProt** | **11.04.2022** |

| | | | | |
|---|---|---|---|---|
| **GABRA5** | **Homo sapiens** | **P31644** | **UniProt** | **11.04.2022** |
| **GABRA5** | **Pan paniscus** | **A0A2R9BFW8** | **UniProt** | **11.04.2022** |
| **GABRA5** | **Mustela putorius furo** | **M3XNJ0** | **UniProt** | **11.04.2022** |
| **GABRA5** | **Rattus norvegicus** | **P19969** | **UniProt** | **11.04.2022** |
| **GABRA5** | **Mus musculus** | **Q8BHJ7** | **UniProt** | **11.04.2022** |
| **GABRA5** | **Canis lupus familiaris** | **E2RG38** | **UniProt** | **11.04.2022** |

| | | | | |
|---|---|---|---|---|
| **GABRA5** | **Pan troglodytes** | **H2Q919** | **UniProt** | **11.04.2022** |
| **GABRA5** | **Danio rerio** | **E9QE70** | **UniProt** | **11.04.2022** |
| **GABRA5** | **Macaca mulatta** | **G7MW76** | **UniProt** | **11.04.2022** |
| **GABRA5** | **Bos taurus** | **Q08E50** | **UniProt** | **11.04.2022** |

| | | | | |
|---|---|---|---|---|
| **GABRA6** | **Homo  sapiens** | **Q16445** | **UniProt** | **11.04.2022** |
| **GABRA6** | **Pan  paniscus** | **A0A2R8ZQ75** | **UniProt** | **11.04.2022** |
| **GABRA6** | **Mustela  putorius furo** | **M3YP65** | **UniProt** | **11.04.2022** |
| **GABRA6** | **Rattus  norvegicus** | **P30191** | **UniProt** | **11.04.2022** |
| **GABRA6** | **Mus  musculus** | **P16305** | **UniProt** | **11.04.2022** |
| **GABRA6** | **Canis  lupus familiaris** | **E2RSN3** | **UniProt** | **11.04.2022** |
| **GABRA6** | **Pan  troglodytes** | **H2QRY3** | **UniProt** | **11.04.2022** |
| **GABRA6b** | **Danio  rerio** | **F1QGW0** | **UniProt** | **11.04.2022** |
| **GABRA6** | **Macaca  mulatta** | **A0A1D5RL15** | **UniProt** | **11.04.2022** |
| **GABRA6** | **Bos taurus** | **E1BE96** | **UniProt** | **11.04.2022** |

| | | | | |
|---|---|---|---|---|
| **GABRB1** | **Homo sapiens** | **P18505** | **UniProt** | **11.04.2022** |

| | | | | |
|---|---|---|---|---|
| GABRB1 | Pan paniscus | A0A2R9C4H8 | UniProt | 11.04.2022 |
| GABRB1 | Mustela putorius furo | M3Y0Y5 | UniProt | 11.04.2022 |
| GABRB1 | Rattus norvegicus | P15431 | UniProt | 11.04.2022 |
| GABRB1 | Mus musculus | P50571 | UniProt | 11.04.2022 |
| GABRB1 | Canis lupus familiaris | F1PEG2 | UniProt | 11.04.2022 |
| GABRB1 | Pan troglodytes | H2QPE8 | UniProt | 11.04.2022 |
| GABRB1 | Danio rerio | F1QPW7 | UniProt | 11.04.2022 |
| GABRB1 | Macaca mulatta | G7MSU9 | UniProt | 11.04.2022 |
| GABRB1 | Bos taurus | P08220 | UniProt | 11.04.2022 |
| GABRB2 | Homo sapiens | P47870 | UniProt | 11.04.2022 |
| GABRB2 | Pan paniscus | A0A2R9C8C8 | UniProt | 11.04.2022 |
| GABRB2 | Mustela putorius furo | M3YP67 | UniProt | 11.04.2022 |
| GABRB2 | Rattus norvegicus | P63138 | UniProt | 11.04.2022 |
| GABRB2 | Mus musculus | P63137 | UniProt | 11.04.2022 |
| GABRB2 | Canis lupus familiaris | A0A5F4CKV8 | UniProt | 11.04.2022 |
| GABRB2 | Pan troglodytes | A0A2J8NKG3 | UniProt | 11.04.2022 |

| | | | | |
|---|---|---|---|---|
| GABRB2 | Danio rerio | Q9DDD9 | UniProt | 11.04.2022 |
| GABRB2 | Macaca mulatta | D1LYT2 | UniProt | 11.04.2022 |
| GABRB2 | Bos taurus | P0C2W5 | UniProt | 11.04.2022 |
| GABRB3 | Homo sapiens | P28472 | UniProt | 11.04.2022 |
| GABRB3 | Pan paniscus | A0A2R9BWE5 | UniProt | 11.04.2022 |
| GABRB3 | Mustela putorius furo | M3XNI6 | UniProt | 11.04.2022 |
| GABRB3 | Rattus norvegicus | P63079 | UniProt | 11.04.2022 |
| GABRB3 | Mus musculus | P63080 | UniProt | 11.04.2022 |
| GABRB3 | Canis lupus familiaris | J9P3X1 | UniProt | 11.04.2022 |
| GABRB3 | Pan troglodytes | A0A2I3THQ1 | UniProt | 11.04.2022 |
| GABRB3 | Danio rerio | A0A0R4ILP2 | UniProt | 11.04.2022 |
| GABRB3 | Macaca mulatta | F6ZKJ4 | UniProt | 11.04.2022 |
| GABRB3 | Bos taurus | A5D7U6 | UniProt | 11.04.2022 |
| GABRD | Homo sapiens | O14764 | UniProt | 11.04.2022 |
| GABRD | Pan paniscus | A0A2R9B726 | UniProt | 11.04.2022 |

| | | | | |
|---|---|---|---|---|
| **GABRD** | **Mustela putorius furo** | **XP_004768807.1** | **NCBI Protein** | **11.04.2022** |
| **GABRD** | **Rattus norvegicus** | **P18506** | **UniProt** | **11.04.2022** |
| **GABRD** | **Mus musculus** | **P22933** | **UniProt** | **11.04.2022** |
| **GABRD** | **Canis lupus familiaris** | **E2R3M6** | **UniProt** | **11.04.2022** |
| **GABRD** | **Pan troglodytes** | **A0A2J8K8J4** | **UniProt** | **11.04.2022** |
| **GABRD** | **Danio rerio** | **E9QHL0** | **UniProt** | **11.04.2022** |
| **GABRD** | **Macaca mulatta** | **F6QDC4** | **UniProt** | **11.04.2022** |
| **GABRD** | **Bos taurus** | **A0A3Q1LQH4** | **UniProt** | **11.04.2022** |

| | | | | |
|---|---|---|---|---|
| **GABRE** | **Homo sapiens** | **P78334** | **UniProt** | **11.04.2022** |
| **GABRE** | **Pan paniscus** | **A0A2R8ZSJ9** | **UniProt** | **11.04.2022** |
| **GABRE** | **Mustela putorius furo** | **M3YJW6** | **UniProt** | **11.04.2022** |
| **GABRE** | **Rattus norvegicus** | **Q9ES14** | **UniProt** | **11.04.2022** |
| **GABRE** | **Mus musculus** | **Q9JLE8** | **UniProt** | **11.04.2022** |
| **GABRE** | **Canis lupus familiaris** | **E2QVM5** | **UniProt** | **11.04.2022** |
| **GABRE** | **Pan troglodytes** | **H2QZ86** | **UniProt** | **11.04.2022** |
| **-** | **Danio rerio** | **-** | **-** | **-** |

| | | | | |
|---|---|---|---|---|
| GABRE | Macaca mulatta | F7GJ80 | UniProt | 11.04.2022 |
| GABRE | Bos taurus | G3MWU9 | UniProt | 11.04.2022 |

| | | | | |
|---|---|---|---|---|
| GABRG1 | Homo sapiens | Q8N1C3 | UniProt | 11.04.2022 |
| GABRG1 | Pan paniscus | A0A2R9A1L5 | UniProt | 11.04.2022 |
| GABRG1 | Mustela putorius furo | M3Y0V1 | UniProt | 11.04.2022 |
| GABRG1 | Rattus norvegicus | P23574 | UniProt | 11.04.2022 |
| GABRG1 | Mus musculus | Q9R0Y8 | UniProt | 11.04.2022 |
| GABRG1 | Canis lupus familiaris | E2RH22 | UniProt | 11.04.2022 |
| GABRG1 | Pan troglodytes | H2QPE4 | UniProt | 11.04.2022 |
| GABRG1 | Danio rerio | A0A0R4IPF9 | UniProt | 11.04.2022 |
| GABRG1 | Macaca mulatta | A0A5F8A0N7 | UniProt | 11.04.2022 |
| GABRG1 | Bos taurus | F6Q4V7 | UniProt | 11.04.2022 |

| | | | | |
|---|---|---|---|---|
| GABRG2 | Homo sapiens | P18507-2 | UniProt | 11.04.2022 |
| GABRG2 | Pan paniscus | A0A2R9CM76 | UniProt | 11.04.2022 |
| GABRG2 | Mustela putorius furo | XP_004737727.1 | NCBI Protein | 11.04.2022 |

| | | | | |
|---|---|---|---|---|
| **GABRG2** | **Rattus norvegicus** | **NP_001380704.1** | **NCBI Protein** | **11.04.2022** |
| **GABRG2** | **Mus musculus** | **P22723** | **UniProt** | **11.04.2022** |
| **GABRG2** | **Canis lupus familiaris** | **E2RSQ1** | **UniProt** | **11.04.2022** |
| **GABRG2** | **Pan troglodytes** | **A0A2I3RPH6** | **UniProt** | **11.04.2022** |
| **GABRG2** | **Danio rerio** | **F1RDP2** | **UniProt** | **11.04.2022** |
| **GABRG2** | **Macaca mulatta** | **F7A3C2** | **UniProt** | **11.04.2022** |
| **GABRG2** | **Bos taurus** | **P22300** | **UniProt** | **11.04.2022** |

| | | | | |
|---|---|---|---|---|
| **GABRG3** | **Homo sapiens** | **Q99928** | **UniProt** | **11.04.2022** |
| **GABRG3** | **Pan paniscus** | **A0A2R9BD98** | **UniProt** | **11.04.2022** |
| **GABRG3** | **Mustela putorius furo** | **XP_044926358.1** | **NCBI Protein** | **11.04.2022** |
| **GABRG3** | **Rattus norvegicus** | **P28473** | **UniProt** | **11.04.2022** |
| **GABRG3** | **Mus musculus** | **P27681** | **UniProt** | **11.04.2022** |
| **GABRG3** | **Canis lupus familiaris** | **F1PHI6** | **UniProt** | **11.04.2022** |
| **GABRG3** | **Pan troglodytes** | **A0A2I3TTK9** | **UniProt** | **11.04.2022** |
| **GABRG3** | **Danio rerio** | **XP_009300843.1** | **NCBI Protein** | **11.04.2022** |

| | | | | |
|---|---|---|---|---|
| GABRG3 | Macaca mulatta | F7HI54 | UniProt | 11.04.2022 |
| GABRG3 | Bos taurus | XP_024838027.1 | NCBI Protein | 11.04.2022 |
| GABRP | Homo sapiens | O00591 | UniProt | 11.04.2022 |
| GABRP | Pan paniscus | A0A2R8ZJ77 | UniProt | 11.04.2022 |
| GABRP | Mustela putorius furo | M3YN80 | UniProt | 11.04.2022 |
| GABRP | Rattus norvegicus | O09028 | UniProt | 11.04.2022 |
| GABRP | Mus musculus | Q8QZW7 | UniProt | 11.04.2022 |
| GABRP | Canis lupus familiaris | E2RS87 | UniProt | 11.04.2022 |
| GABRP | Pan troglodytes | A0A2J8LXV6 | UniProt | 11.04.2022 |
| GABRP | Danio rerio | XP_021330000.1 | NCBI Protein | 11.04.2022 |
| GABRP | Macaca mulatta | A0A1D5QJZ6 | UniProt | 11.04.2022 |
| GABRP | Bos taurus | Q5EA06 | UniProt | 11.04.2022 |
| GABRQ | Homo sapiens | Q9UN88 | UniProt | 11.04.2022 |
| GABRQ | Pan paniscus | A0A2R9ALH2 | UniProt | 11.04.2022 |
| GABRQ | Mustela putorius furo | M3YJY6 | UniProt | 11.04.2022 |

| | | | | |
|---|---|---|---|---|
| GABRQ | Rattus norvegicus | G3V875 | UniProt | 11.04.2022 |
| GABRQ | Mus musculus | Q9JLF1 | UniProt | 11.04.2022 |
| GABRQ | Canis lupus familiaris | F6XRX3 | UniProt | 11.04.2022 |
| GABRQ | Pan troglodytes | A0A6D2XHJ7 | UniProt | 11.04.2022 |
| GABRB4 | Danio rerio | F1Q4Y6 | UniProt | 11.04.2022 |
| GABRQ | Macaca mulatta | F7F4H9 | UniProt | 11.04.2022 |
| GABRQ | Bos taurus | E1BJH4 | UniProt | 11.04.2022 |
| GABRR1 | Homo sapiens | P24046 | UniProt | 11.04.2022 |
| GABRR1 | Pan paniscus | A0A2R9BL98 | UniProt | 11.04.2022 |
| GABRR1 | Mustela putorius furo | M3YD49 | UniProt | 11.04.2022 |
| GABRR1 | Rattus norvegicus | P50572 | UniProt | 11.04.2022 |
| GABRR1 | Mus musculus | P56475 | UniProt | 11.04.2022 |
| GABRR1 | Canis lupus familiaris | XP_038538749.1 | NCBI Protein | 11.04.2022 |
| GABRR1 | Pan troglodytes | H2QTE3 | UniProt | 11.04.2022 |
| GABRR1 | Danio rerio | Q5TZ16 | UniProt | 11.04.2022 |

| | | | | |
|---|---|---|---|---|
| **GABRR1** | **Macaca mulatta** | **F6W0N4** | **UniProt** | **11.04.2022** |
| **GABRR1** | **Bos taurus** | **Q0II76** | **UniProt** | **11.04.2022** |
| **GABRR2** | **Homo sapiens** | **P28476** | **UniProt** | **11.04.2022** |
| **GABRR2** | **Pan paniscus** | **A0A2R9AXF6** | **UniProt** | **11.04.2022** |
| **GABRR2** | **Mustela putorius furo** | **M3YCV9** | **UniProt** | **11.04.2022** |
| **GABRR2** | **Rattus norvegicus** | **P47742** | **UniProt** | **11.04.2022** |
| **GABRR2** | **Mus musculus** | **P56476** | **UniProt** | **11.04.2022** |
| **GABRR2** | **Canis lupus familiaris** | **E2R4R0** | **UniProt** | **11.04.2022** |
| **GABRR2** | **Pan troglodytes** | **XP_527448.5** | **NCBI Protein** | **11.04.2022** |
| **GABRR2a** | **Danio rerio** | **F1QX34** | **UniProt** | **11.04.2022** |
| **GABRR2** | **Macaca mulatta** | **XP_001095465.3** | **NCBI Protein** | **11.04.2022** |
| **GABRR2** | **Bos taurus** | **Q0II76** | **UniProt** | **11.04.2022** |
| **GABRR3** | **Homo sapiens** | **A8MPY1** | **UniProt** | **11.04.2022** |
| **GABRR3** | **Pan paniscus** | **A0A2R9BJ01** | **UniProt** | **11.04.2022** |
| **GABRR3** | **Mustela putorius furo** | **M3XTX4** | **UniProt** | **11.04.2022** |

| | | | | |
|---|---|---|---|---|
| **GABRR3** | **Rattus norvegicus** | **P50573** | **UniProt** | **11.04.2022** |
| **GABRR3** | **Mus musculus** | **B2RXA8** | **UniProt** | **11.04.2022** |
| **GABRR3** | **Canis lupus familiaris** | **-** | **-** | **-** |
| **GABRR3** | **Pan troglodytes** | **A0A2J8MY57** | **UniProt** | **11.04.2022** |
| **GABRR3a** | **Danio rerio** | **B3DIE5** | **UniProt** | **11.04.2022** |
| **GABRR3** | **Macaca mulatta** | **F6YLK3** | **UniProt** | **11.04.2022** |
| **GABRR3** | **Bos taurus** | **E1B988** | **UniProt** | **11.04.2022** |

**Supplementary Table S7**: All sources (subunit, sequence ID, database) of protein sequences used for the alignments of all species in different coloured groups (all alpha subunits, all beta subunits, etc).

## References

Bocquet N, Nury H, Baaden M, Le Poupon C, Changeux J-P, Delarue M*, et al.* (2009). X-ray structure of a pentameric ligand-gated ion channel in an apparently open conformation. *Nature* **457**(7225)**:** 111-114.

Chen Q, Wells MM, Arjunan P, Tillman TS, Cohen AE, Xu Y*, et al.* (2018). Structural basis of neurosteroid anesthetic action on GABAA receptors. *Nature communications* **9**(1)**:** 3972.

Fourati Z, Howard RJ, Heusser SA, Hu H, Ruza RR, Sauguet L*, et al.* (2018). Structural Basis for a Bimodal Allosteric Mechanism of General Anesthetic Modulation in Pentameric Ligand-Gated Ion Channels. *Cell Reports* **23**(4)**:** 993-1004.

Hénault CM, Govaerts C, Spurny R, Brams M, Estrada-Mondragon A, Lynch J*, et al.* (2019). A lipid site shapes the agonist response of a pentameric ligand-gated ion channel. *Nature chemical biology* **15**(12)**:** 1156-1164.

Huang X, Chen H, Shaffer PL (2017). Crystal Structures of Human GlyRα3 Bound to Ivermectin. *Structure (London, England : 1993)* **25**(6)**:** 945-950.e942.

Kasaragod VB, Mortensen M, Hardwick SW, Wahid AA, Dorovykh V, Chirgadze DY*, et al.* (2022). Mechanisms of inhibition and activation of extrasynaptic αβ GABAA receptors. *Nature* **602**(7897)**:** 529-533.

Khaleel MA, Khan AH, Ghadzi SM, Adnan AS, Abdallah QM (2022). A Standardized Dataset of a Spontaneous Adverse Event Reporting System. In: *Healthcare* Vol. 10.

Kim JJ, Gharpure A, Teng J, Zhuang Y, Howard RJ, Zhu S*, et al.* (2020). Shared structural mechanisms of general anaesthetics and benzodiazepines. *Nature* **585**(7824)**:** 303-308.

Laverty D, Thomas P, Field M, Andersen OJ, Gold MG, Biggin PC*, et al.* (2017). Crystal structures of a GABAA-receptor chimera reveal new endogenous neurosteroid-binding sites. *Nature structural & molecular biology*.

Liu S, Xu L, Guan F, Liu Y-T, Cui Y, Zhang Q*, et al.* (2018). Cryo-EM structure of the human α5β3 GABAA receptor. *Cell Research* **28**(9)**:** 958-961.

Masiulis S, Desai R, Uchański T, Serna Martin I, Laverty D, Karia D*, et al.* (2019). GABAA receptor signalling mechanisms revealed by structural pharmacology. *Nature*.

Miller PS, Aricescu AR (2014). Crystal structure of a human GABAA receptor. *Nature* **512**(7514)**:** 270-275.

Miller PS, Scott S, Masiulis S, De Colibus L, Pardon E (2017). Structural basis for GABAA receptor potentiation by neurosteroids. **24**(11)**:** 986-992.

Nury H, Van Renterghem C, Weng Y, Tran A, Baaden M, Dufresne V*, et al.* (2011). X-ray structures of general anaesthetics bound to a pentameric ligand-gated ion channel. *Nature* **469**(7330)**:** 428-431.

Nys M, Wijckmans E, Farinha A, Yoluk O, Andersson M, Brams M*, et al.* (2016). Allosteric binding site in a Cys-loop receptor ligand-binding domain unveiled in the crystal structure of ELIC in complex with chlorpromazine. *Proceedings of the National Academy of Sciences of the United States of America* **113**(43)**:** E6696-e6703.

Pan J, Chen Q, Willenbring D, Mowrey D, Kong X-P, Cohen A*, et al.* (2012). Structure of the Pentameric Ligand-Gated Ion Channel GLIC Bound with Anesthetic Ketamine. *Structure (London, England : 1993)* **20**(9)**:** 1463-1469.

Phulera S, Zhu H (2018). Cryo-EM structure of the benzodiazepine-sensitive alpha1beta1gamma2S tri-heteromeric GABAA receptor in complex with GABA. **7**.

Puthenkalam R, Hieckel M, Simeone X, Suwattanasophon C, Feldbauer RV, Ecker GF*, et al.* (2016). Structural Studies of GABA-A receptor binding sites: Which experimental structure tells us what? *Frontiers in Molecular Neuroscience* **9**.

Sauguet L, Howard RJ, Malherbe L, Lee US, Corringer P-J, Adron Harris R*, et al.* (2013). Structural basis for potentiation by alcohols and anaesthetics in a ligand-gated ion channel. *Nature communications* **4**(1)**:** 1697.

Sente A, Desai R, Naydenova K, Malinauskas T, Jounaidi Y, Miehling J*, et al.* (2022). Differential assembly diversifies GABAA receptor structures and signalling. *Nature* **604**(7904)**:** 190-194.

Spurny R, Billen B, Howard RJ, Brams M, Debaveye S, Price KL*, et al.* (2013). Multisite Binding of a General Anesthetic to the Prokaryotic Pentameric <em>Erwinia chrysanthemi</em> Ligand-gated Ion Channel (ELIC) *. *Journal of Biological Chemistry* **288**(12)**:** 8355-8364.

Spurny R, Debaveye S, Farinha A, Veys K, Vos AM, Gossas T*, et al.* (2015). Molecular blueprint of allosteric binding sites in a homologue of the agonist-binding domain of the α7 nicotinic acetylcholine receptor. *Proceedings of the National Academy of Sciences* **112**(19)**:** E2543-E2552.

Uchański T, Masiulis S, Fischer B, Kalichuk V, López-Sánchez U, Zarkadas E*, et al.* (2021). Megabodies expand the nanobody toolkit for protein structure determination by single-particle cryo-EM. *Nature Methods* **18**(1)**:** 60-68.

Ulens C, Spurny R, Thompson Andrew J, Alqazzaz M, Debaveye S, Han L*, et al.* (2014). The Prokaryote Ligand-Gated Ion Channel ELIC Captured in a Pore Blocker-Bound Conformation by the Alzheimer’s Disease Drug Memantine. *Structure (London, England : 1993)* **22**(10)**:** 1399-1407.

Zhu S, Noviello CM, Teng J, Walsh RM, Kim JJ, Hibbs RE (2018). Structure of a human synaptic GABAA receptor. *Nature* **559**(7712)**:** 67-72.

Zhu S, Sridhar A, Teng J, Howard RJ, Lindahl E, Hibbs RE (2022). Structural and dynamic mechanisms of GABAA receptor modulators with opposing activities. *Nature communications* **13**(1)**:** 4582.

Zimmermann I, Marabelli A, Bertozzi C, Sivilotti LG, Dutzler R (2012). Inhibition of the Prokaryotic Pentameric Ligand-Gated Ion Channel ELIC by Divalent Cations. *PLOS Biology* **10**(11)**:** e1001429.
